# Warfarin dosing algorithms: A systematic review

**DOI:** 10.1111/bcp.14608

**Published:** 2020-11-18

**Authors:** Innocent G. Asiimwe, Eunice J. Zhang, Rostam Osanlou, Andrea L. Jorgensen, Munir Pirmohamed

**Affiliations:** ^1^ The Wolfson Centre for Personalized Medicine, MRC Centre for Drug Safety Science, Department of Pharmacology and Therapeutics, Institute of Systems, Molecular and Integrative Biology University of Liverpool United Kingdom; ^2^ Department of Biostatistics, Institute of Population Health Sciences University of Liverpool United Kingdom

**Keywords:** clinical factors, demographic factors, dosing algorithms, genetic factors, warfarin

## Abstract

**Aims:**

Numerous algorithms have been developed to guide warfarin dosing and improve clinical outcomes. We reviewed the algorithms available for various populations and the covariates, performances and risk of bias of these algorithms.

**Methods:**

We systematically searched MEDLINE up to 20 May 2020 and selected studies describing the development, external validation or clinical utility of a multivariable warfarin dosing algorithm. Two investigators conducted data extraction and quality assessment.

**Results:**

Of 10 035 screened records, 266 articles were included in the review, describing the development of 433 dosing algorithms, 481 external validations and 52 clinical utility assessments. Most developed algorithms were for dose initiation (86%), developed by multiple linear regression (65%) and mostly applicable to Asians (49%) or Whites (43%). The most common demographic/clinical/environmental covariates were age (included in 401 algorithms), concomitant medications (270 algorithms) and weight (229 algorithms) while *CYP2C9* (329 algorithms), *VKORC1* (319 algorithms) and *CYP4F2* (92 algorithms) variants were the most common genetic covariates. Only 26% and 7% algorithms were externally validated and evaluated for clinical utility, respectively, with <2% of algorithm developments and external validations being rated as having a low risk of bias.

**Conclusion:**

Most warfarin dosing algorithms have been developed in Asians and Whites and may not be applicable to under‐served populations. Few algorithms have been externally validated, assessed for clinical utility, and/or have a low risk of bias which makes them unreliable for clinical use. Algorithm development and assessment should follow current methodological recommendations to improve reliability and applicability, and under‐represented populations should be prioritized.

## 1 INTRODUCTION

Warfarin remains the most commonly prescribed oral anticoagulant for the management of thromboembolic disorders.^1^ However, dosing remains challenging due to warfarin's narrow therapeutic index and highly variable clinical response. These dosing challenges usually result in a high frequency of adverse effects (thrombosis and bleeding) as well as an increased burden to the patient (e.g. more frequent monitoring), which could impact quality of life and lead to treatment discontinuation of an otherwise highly efficacious drug.^2^ To better predict an individual's warfarin dose requirements, numerous dose‐prediction algorithms based on demographic, clinical, environment and genetic factors have been developed.^3^, ^4^ Algorithms incorporating only demographic, clinical and environmental factors have been designated as *clinical* and those additionally incorporating genetic factors as *pharmacogenetic*.^5^

The availability of numerous published dosing algorithms provides a wealth of information but makes navigating the literature to identify which algorithms to use or recommend, and in which patients/populations difficult. This task becomes more complicated if it is not clear if the identified algorithms have low risks of bias, have been externally validated and/or have been evaluated for clinical utility. The Clinical Pharmacogenetics Implementation Consortium (CPIC) has recommended the use of 4 dosing algorithms.^1^ However, these may not be applicable to non‐Caucasians or for some categories of patients such as those with international normalized ratio (INR) targets outside of the 2–3 range.^1^, ^6^, ^7^

Previous reviews have attempted to describe available dosing algorithms but most, if not all, have had the main limitation of focusing on a limited number of algorithms. For example Saffian *et al*.^8^ evaluated 16 algorithms but these were only from studies that had: (i) evaluated the algorithm's predictive ability in the form of a high resolution scatterplot (observed *vs* predicted maintenance doses); (ii) used a validation dataset; and (iii) included at least 5 patients requiring warfarin at a dose >7 mg/d. Other reviews including those by Verhoef *et al*.^9^ (32 algorithms) and Shendre *et al*.^10^ (50 eligible studies) have been narrative in nature and consequently neither reported a detailed search strategy nor assessed the risk of bias of included studies. Additionally, both these narrative reviews included only pharmacogenetic algorithms and the Shendre *et al*.^10^ review additionally excluded studies that did not involve European or African ancestry populations. To methodologically assess and describe the knowledge base accumulated so far, we have undertaken this systematic review, which includes both clinical and pharmacogenetic algorithms with no population‐based exclusion criteria. Our aim was to accurately and comprehensively summarize which algorithms are available for which populations and the covariates (demographic, clinical, environmental, genetic), performances and the risk of bias of these algorithms.

## 2 METHODS

### 2.1 Search strategy and selection criteria

A predefined protocol (PROSPERO: CRD42019147995), based on the principles set in the CHARMS (CHecklist for critical Appraisal and data extraction for systematic Reviews of prediction Modelling Studies) checklist,^11^ and PROBAST (Prediction model Risk Of Bias Assessment Tool), a tool meant to assess the risk of bias and applicability of prediction model studies^12^ was followed. This report adheres to the Preferred Reporting Items for Systematic Reviews and Meta‐Analyses (PRISMA) statement (Table S1). MEDLINE records (from 1946 to 22 August 2019) were searched using medical subject headings (MeSH terms) and text words related to “warfarin”, “algorithm” and “dosing” (Table S2). A second MEDLINE search was conducted on 20 May 2020 to identify records published after our first search. Lists of references from the identified studies were hand‐searched to identify further eligible studies. Non‐English language studies were excluded.

Observational (e.g. cohort studies) and interventional (e.g. randomised controlled trials) studies that developed, validated or assessed the impact/clinical utility of warfarin dosing algorithms in any warfarin‐treated population were included. For development studies, those that modelled at least 2 predictor variables (not counting dose and INR readings for dose‐revision algorithms) and either: (i) explicitly stated in their aims that they were developing a dosing algorithm; or (ii) reported dosing equations, nomograms, charts, tables, computer programs etc. that can be used to provide a daily or weekly dose, were included. In all cases, the included predictors had to have been reported. Unless enough details pertaining to algorithm development were available in an external validation or clinical utility assessment study or elsewhere (corresponding development study, other studies), external validation or clinical utility assessment studies were excluded. Additionally, for a clinical utility assessment study to be included, a comparison between a dosing algorithm with an alternative strategy (such as existing clinical practice) was a prerequisite. For the purposes of this review, clinical utility^13^ was defined as the demonstration that a dosing algorithm improved the quality of anticoagulation (based on the time spent in the therapeutic INR range) or lead to better clinical endpoints (such as fewer bleeding episodes). Not to be confused with the outcome to be predicted in the individual studies (i.e. the stable warfarin dose), the primary outcome of interest in this review was the warfarin dose‐prediction algorithm developed, and whether it was externally validated or evaluated for clinical utility in the included studies.

### 2.2 Data extraction and quality assessment

One reviewer (I.G.A.) screened titles and abstracts of the retrieved bibliographic records for eligibility. For all stages, a second reviewer (R.O.) independently checked a random 10% of the records to check for consistency. Disagreements were resolved by consensus and because the first reviewer was consistent with regard to following agreed upon criteria, only the first reviewer continued reviewing the remaining records. A data extraction form was adapted from the CHARMS^11^ and PROBAST^12^ tools, piloted in a subset of randomly selected included papers and used to extract relevant information related to participants, predictors, outcome, analysis and results. When a single publication reported both development and external validation studies (and/or clinical utility assessments), or multiple algorithms, each study/algorithm was assessed separately.^12^ The exception was studies that reported the warfarindosing.com platform—although this platform incorporates multiple algorithms, it was not possible to separate the individual algorithms and so it was considered as 2, the clinical and pharmacogenetic Gage algorithms.^14^ Algorithm updating/extension studies in which new predictors were added to existing algorithms were considered as new algorithm development studies.^12^

To assess the methodological quality of each included development or external validation study, the 2 reviewers used the PROBAST tool.^12^ Although this tool focuses on prediction models that consider binary or time‐to‐event outcomes and studies that use generalized linear modelling, its authors encourage its use in studies that consider other outcomes and other machine learning techniques such as those explored in this review.^12^ It should, however, be tailored to these other outcomes/techniques as we did in Tables S3 and S4 and Figure S1. For reasons detailed in Table S3, emphasis was placed on the assessment of the risk of bias in the analysis domain. We did not assess the methodological quality (and performance) of clinical utility (impact) assessment studies since these have been previously explored in several systematic reviews and meta‐analyses.^15^, ^16^, ^17^, ^18^, ^19^, ^20^, ^21^, ^22^

### 2.3 Data synthesis

This systematic review was qualitative in nature and no attempt to quantitatively synthesise studies by way of meta‐analysis was conducted. Consequently, heterogeneity measures and publication bias were not explored. The descriptive results (i.e. proportions and measures of central tendency and dispersion) are presented in structured tables, graphically and as a narrative summary. Where appropriate, the results were stratified according to the type of algorithm (clinical *vs* pharmacogenetic) and ethnic populations for which they were developed. For the purposes of stratifying by population, we used the 4 categories (White, Asian, Black and Mixed/Other) reported by 1 of the largest warfarin‐related studies to date, the International Warfarin Pharmacogenetics Consortium (IWPC) study.^23^ Where these race categories were unreported, country was used as a proxy (for example populations from China were categorized as Asian while populations from northern Europe as White). Regarding which algorithm would be relevant to a given population, we arbitrarily chose a 5% cut‐off, i.e. an algorithm that recruited at least 5% of a given population would be applicable to that population. These descriptive analyses were conducted in R (version 3.6.1).^24^ No sensitivity analyses were conducted.

### 2.4 Nomenclature of targets and ligands

Key protein targets and ligands in this article are hyperlinked to corresponding entries in http://www.guidetopharmacology.org, the common portal for data from the IUPHAR/BPS Guide to PHARMACOLOGY.

## 3 RESULTS

**FIGURE 1:**
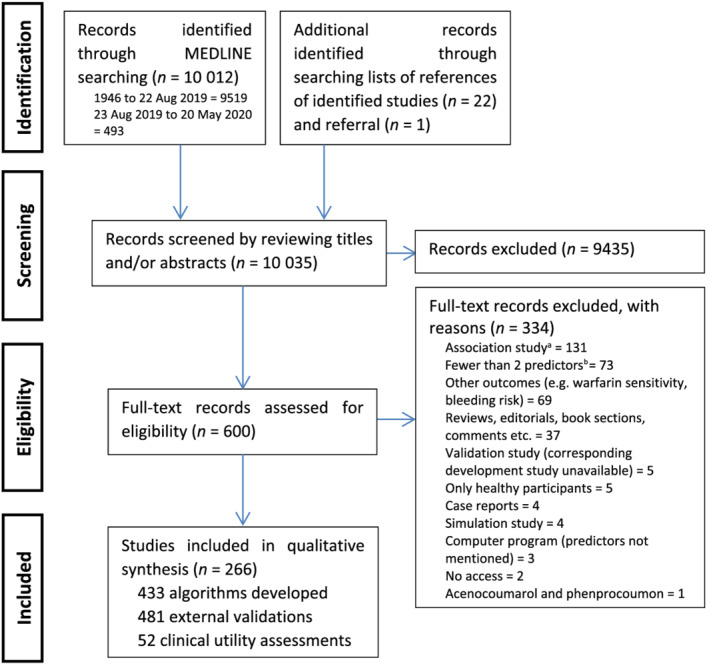
PRISMA flow chart of included studies. ^a^Includes studies that neither stated in their aims that they were developing/validating a dosing algorithm nor reported dosing equations, nomograms, charts, tables, or other tools that can be used to provide a daily or weekly dose. ^b^Prior doses and international normalized ratios not counted

We aimed to summarize which algorithms are available for which populations, and the covariates, performances and risk of bias of these algorithms. Figure 1 illustrates the literature search and selection process; 10 035 records were identified of which 9435 were excluded based on the title and abstract. Of 600 full text records assessed for eligibility, 266 met the eligibility criteria and were included in the qualitative synthesis. Of these, 205, 123 and 32 articles, respectively, described algorithm development, external validation and clinical utility assessment. Some articles described both algorithm development and external validation (*n* = 74), algorithm development and clinical utility assessment (*n* = 10), external validation and clinical utility assessment (*n* = 15) while 5 articles reported all 3 components (Figure S2). The included articles described the development of 433 dosing algorithms, 481 external validations and 52 clinical utility assessments, whose characteristics are summarised in Table 1 and detailed in Tables S5 and S6 (algorithm development), Table S7 (external validations), and Table S8 (clinical utility assessments).

**TABLE 1:** Summary characteristics of algorithm developments, external validations, and clinical utility assessments

Characteristic	Algorithm development (*n* = 433)	External validations (*n* = 481)	Clinical utility assessments (*n* = 52)
Publication year, *n* (%)			
2000 and before	7 (1.6)	‐	‐
2001 to 2005	12 (2.8)	3 (0.6)	2 (3.8)
2006 to 2010	75 (17.3)	81 (16.8)	12 (23.1)
2011 to 2015	175 (40.4)	224 (46.6)	18 (34.6)
`2016 to 2020	164 (37.9)	173 (36.0)	17 (32.7)
Sample size, median (range)	229 (18–10,673)	125 (28–2,181)	234 (10–2,343)
Participants (included), *n* (%)			
≥5% white	186 (43.0)	205 (42.6)	36 (69.2)
≥5% Asian	210 (48.5)	277 (57.6)	17 (32.7)
≥5% black	121 (27.9)	115 (23.9)	16 (30.8)
≥5% mixed/other	77 (17.8)	62 (12.9)	2 (3.8)
Adults	422 (97.5)	455 (94.6)	49 (94.2)
Children	11 (2.5)	26 (5.4)	3 (5.8)
Location, *n* (%)			
Africa^a^	2 (0.5)	2 (0.4)	‐
Asia^b^	175 (40.4)	208 (43.2)	14 (26.9)
Europe	34 (7.9) 121	55 (11.4)	11 (21.2)
North America	136 (31.4)	121 (25.2)	25 (48.1)
South America	15 (3.5)	21 (4.4)	‐
Middle East	30 (6.9)	25 (5.2)	2 (3.8)
Oceania	‐	8 (1.7)	‐
Multiple	41 (9.5)	41 (8.5)	‐
Covariates included, *n* (%)			
Clinical^c^ only	87 (20.1)	49 (10.2)	11 (21.2)
Genetic only^d^	2 (0.5)	‐	‐
Clinical^c^ and genetic	344 (79.4)	432 (89.8)	41 (78.8)
Application time, *n* (%)			
Dose initiation	373 (86.1)	443 (92.1)	40 (76.9)
Dose revision	41 (9.5)	31 (6.4)	10 (19.2)
Both initiation and revision^e^	19 (4.4)	7 (1.5)	2 (3.8)
Modelling techniques, *n* (%)			
Artificial neural network	32 (7.4)	2 (0.4)	1 (1.9)
Multiple linear regression	280 (64.7)	458 (95.2)	47 (90.4)
Nonlinear mixed effects^f^	14 (3.2)	7 (1.5)	3 (5.8)
Support vector regression	27 (6.2)	2 (0.4)	‐
Other^g^	66 (15.2)	9 (1.9)	‐
Unclear	10 (2.3)	3 (0.6)	1 (1.9)
Algorithm presentation, *n* (%)			
Computer program^h^	10 (2.3)	4 (0.8)	4 (7.7)
Nomogram/table	9 (2.1)	3 (0.6)	‐
Regression formula	239 (55.2)	453 (94.2)	47 (90.4)
None	175 (40.4)	21 (4.4)	1 (1.9)

^a^ Excludes Egypt, which is under Middle East.

^b^ Mostly China (131 algorithm developments, 120 external validations and 11 clinical utility assessments). This was followed by South Korea (16 algorithm developments, 59 external validations and 1 clinical utility assessment) and Japan (10 algorithm developments and 14 external validations).

^c^ Clinical includes clinical, demographic, and environmental variables.

^d^ Clinical factors also considered during the modelling.

^e^ All incorporate pharmacokinetic and/or pharmacodynamic techniques.

^f^ Used to fit pharmacokinetic/pharmacodynamic‐based algorithms.

^g^ See Table S6 for details.

^h^ Or online tool.

**FIGURE 2:**
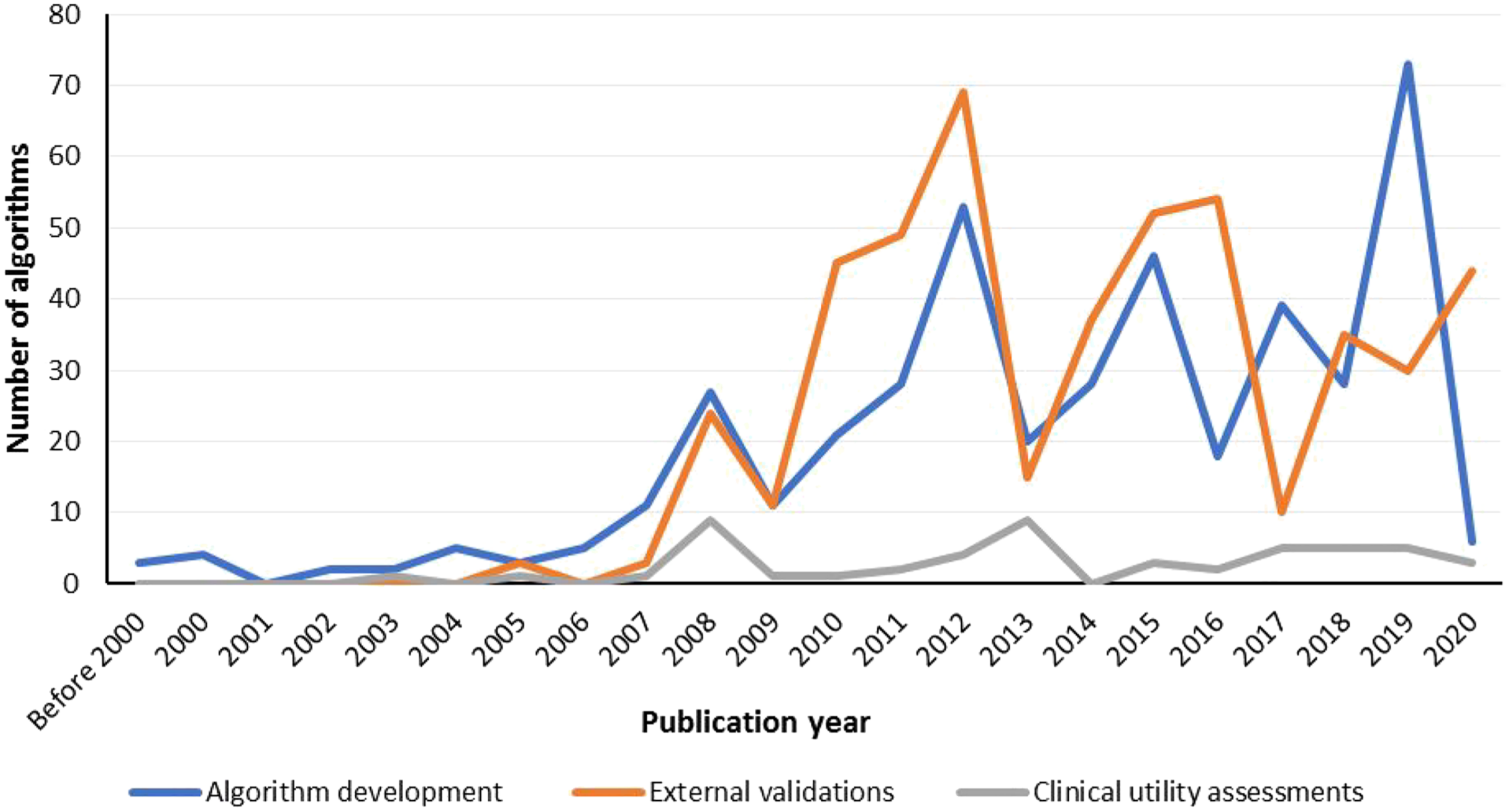
Algorithm development/evaluation by publication year

Out of all date ranges investigated, the period during which most algorithm developments/evaluations were published was 2011–2015 in which 175 (41%), 224 (47%) and 18 (35%) algorithms were developed, externally validated and assessed for clinical utility, respectively (Table 1, Figure 2). The median sample sizes for these studies were 229 (range 18–10 673), 125 (28–2181) and 234 (10–2343), respectively. Children were studied less often—algorithm developments, external validations and clinical utility assessments in children were 11 (3%), 26 (5%) and 3 (6%), respectively. Asia had the highest number of algorithm developments (*n* = 175, 40%) and external validations (*n* = 208, 43%), while North America had the highest rate of clinical utility assessments (*n* = 25, 48%). Most of the developed algorithms included both clinical and genetic covariates (*n* = 344, 79%), were mostly for dose‐initiation (*n* = 373, 86%), were developed using multiple linear regression (*n* = 280, 65%) and presented a regression formula that could be used to compute a weekly or daily dose (*n* = 239, 55%). Of the developed algorithms, 111 (26%) and 30 (7%) algorithms were respectively externally validated or assessed for clinical utility at least once (Table S9). The 5 most externally validated algorithms were all dose‐initiation pharmacogenetic algorithms and included those by the IWPC^23^ (*n* = 72 external validations), Gage^14^ (*n* = 46), Sconce^25^ (*n* = 32), Wadelius^26^ (*n* = 20) and Huang^27^ (*n* = 19) while the 4 most clinically assessed algorithms were the Gage pharmacogenetic algorithm^14^ (*n* = 8 clinical utility assessments), IWPC pharmacogenetic algorithm^23^ (*n* = 7), Gage clinical algorithm^14^ (*n* = 5) and Lenzini dose revision pharmacogenetic algorithm^28^ (*n* = 4). Consequently, most external validations were conducted on pharmacogenetic (*n* = 432 external validations, 90%) and dose initiation (*n* = 443, 92%) algorithms, algorithms developed using multiple linear regression (*n* = 458, 95%) and those that presented a regression formula (*n* = 453, 94%). A similar trend was observed for the clinical utility assessments (Table 1).

### 3.1 Study populations

Of the 433 developed algorithms, 186 (43%), 210 (49%), 121 (28%) and 77 (18%) used datasets that included at least 5% White, 5% Asian, 5% Black and 5% Mixed/Other participants, respectively (Table 1). For the studies including at least 5% White participants, the median percentage of White participants was 90% (range 33–100%; Table S10). Corresponding values for Asian, Black and Mixed/Other cohorts were 100% (7–100%; Table S11), 14% (5–100%; Table S12) and 71% (5–100%; Table S13), respectively. Other characteristics stratified by populations are detailed in Tables S10–S13. For the developed algorithms that included at least 5% White participants in their datasets, 54 (29%) and 20 (11%) algorithms were respectively externally validated and assessed for clinical utility at least once (Table S14). The corresponding values were 55 (26%) and 10 (5%) for Asians (Table S15), 34 (28%) and 10 (8%) for Blacks (Table S16), and 14 (18%) and 2 (3%) for Mixed/Other (Table S16). It is important to note that these algorithms were not always validated in populations for whom they were developed.

### 3.2 Predictors

**FIGURE 3:**
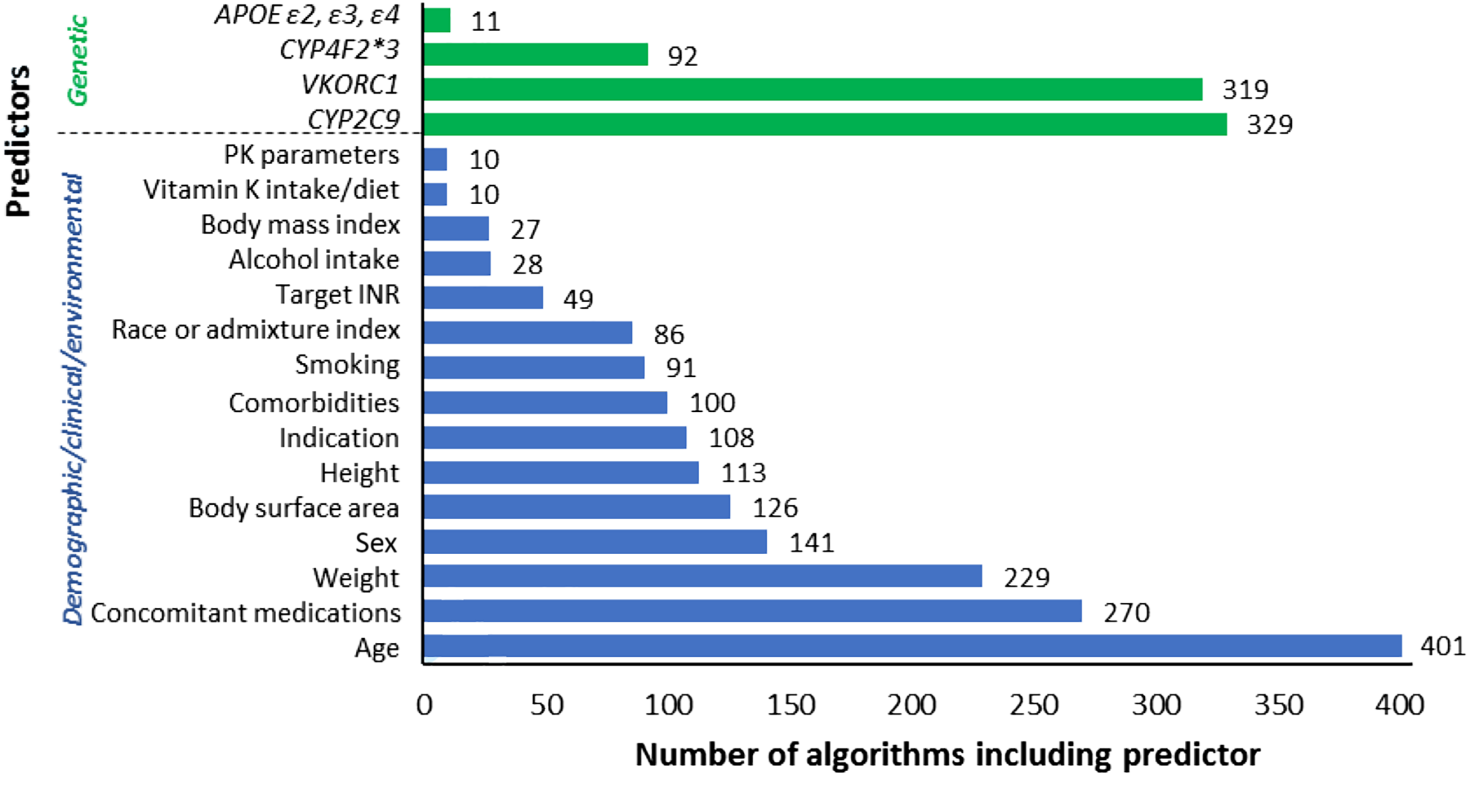
Predictors included in at least 10 algorithms. *APOE*, apolipoprotein E; *CYP2C9*, cytochrome P450, family 2, subfamily C, polypeptide 9; *CYP4F2*, cytochrome P450, family 4, subfamily F, polypeptide 2; PK parameters, pharmacokinetic parameters (S‐warfarin clearance and/or distribution volume); INR, international normalized ratio; *VKORC1*, vitamin K epoxide reductase complex subunit 1

During algorithm development, all 433 algorithms explored demographic, clinical, and environmental predictors and for these the median number of predictors included in the final algorithms was 5 (range 0–23). Conversely, only 346 algorithms explored genetic factors with the median number of genetic predictors included in the final algorithms being 3 (range 1–205). The predictors included in at least 10 algorithms are shown in Figure 3. Age (included in 401 algorithms), concomitant medications (270 algorithms, amiodarone in 201 algorithms), weight (229 algorithms) and sex (141 algorithms) were the 4 most common demographic/clinical/environmental predictors. Comorbidities were included in 100 algorithms and these included renal disease (42 algorithms), hepatic disease (40 algorithms), hypertension (27 algorithms) and diabetes mellitus (26 algorithms). The genes most frequently included in the pharmacogenetic algorithms were *CYP2C9* (329 algorithms), *VKORC1* (319 algorithms), *CYP4F2* (92 algorithms) and *APOE* (11 algorithms). *CYP2C9* variants included CYP2C9*2 (in 206 algorithms), CYP2C9*3 (316 algorithms) and other variants (64 algorithms), while *VKORC1* variants included *VKORC1‐1639G > A* (270 algorithms), *VKORC1 1173C > T*, (75 algorithms), *VKORC1* 3730G > A (20 algorithms) and a number of others (34 algorithms). Figure S3 shows numbers stratified by the 4 population categories. Age remained the most included demographic/clinical/environmental predictor in each of the 4 categories, while *CYP2C9* remained the most common predictor in Whites, Blacks and the Mixed/Other population categories, but was overtaken by *VKORC1* in Asians. The Asian algorithms that included the *CYP2C9* gene (*n* = 153) mostly focused on the CYP2C9*3 variant (*n* = 149, 97%) as opposed to the CYP2C9*2 (*n* = 44, 29%) or other *CYP2C9* (*n* = 7, 5%) variants. The corresponding proportions for inclusion of CYP2C9*3 vs CYP2C9*2 vs other *CYP2C9* variants, respectively, were 99 *vs* 92 *vs* 19% (Whites, *n* = 153 algorithms), 99 *vs* 96 *vs* 36% (Blacks, *n* = 99) and 88 *vs* 95 *vs* 30% (Mixed/Other, *n* = 64).

### 3.3 Predictive performance

A fit accuracy measure (the coefficient of determination, *R* ^2^) was the most commonly reported performance measure during both algorithm development (323 [75% of 433]) and external validation (261 [54% of 481]; Table 2). The *R* ^2^ value represents the proportion of total interpatient variability in warfarin dose requirements that can be jointly accounted for by the variables included in an algorithm. For algorithm development, the median variability in warfarin dose explained by included predictors was 43% (range 2–96%). This was higher (median 45%, range 8–96%) when only pharmacogenetic algorithms were considered and lower (median 20%, range 2–83%) when only clinical factors were considered. The *CYP2C9* and *VKORC1* genes, respectively, accounted for a median of 7% (<1–50%) and median of 25% (1–59%) of the variability in warfarin dose requirements. The *R* ^2^ performance stratified by race is shown in Table S17.

**TABLE 2:** Performance measures

Measures		Algorithm development (*n* = 433)		External validations (*n* = 481)	
		*N* ^a^	Median (range)	*N* ^a^	Median (range)
Fit accuracy	*R* ^2^ ^b^ (%)				
	All	323	43 (2–96^c^)	261	39 (<1–86)
	Pharmacogenetic	273	45 (8–96)	232	41 (<1–86)
	Clinical^d^	178	20 (2–83)	29	24 (<1–69)
	*CYP2C9*	98	7 (<1–50)	‐	‐
	*VKORC1*	114	25 (1–59)	‐	‐
	Correlation coefficient				
	All	19	0.65 (0.31–0.82)	101	0.60 (0.03–0.86)
	Pharmacogenetic	15	0.65 (0.52–0.79)	97	0.60 (0.03–0.86)
	Clinical	4	0.56 (0.31–0.82)	4	0.32 (0.07–0.54)
Precision/predictive accuracy	Mean absolute error (mg/d)^e^ ^,^ ^f^				
	All	137	1.23 (0.11–2.89)	222	1.20 (0.37–3.70)
	Pharmacogenetic	105	1.26 (0.11–1.96)	185	1.18 (0.57–3.30)
	Clinical	32	1.10 (0.21–2.89)	37	1.34 (0.37–3.70)
	Mean square error (mg^2^/d^2^)				
	All	54	0.02 (0.01–0.74)	4	0.67 (0.60–0.74)
	Pharmacogenetic	30	0.02 (0.01–0.10)	‐	‐
	Clinical	24	0.02 (0.01–0.74)	4	0.67 (0.60–0.74)
	Root mean square error (mg/d)				
	All	14	0.80 (0.10–3.09)	68	1.44 (0.19–4.29)
	Pharmacogenetic	6	0.34 (0.10–1.44)	58	1.37 (0.19–4.29)
	Clinical	8	1.87 (0.66–3.09)	10	1.77 (0.66–2.33)
	Mean absolute percentage error (%)^f^				
	All	7	21 (13–54)	37	32 (20–53)
	Pharmacogenetic	6	25 (18–54)	34	32 (21–53)
	Clinical	1	19 (13–21)	3	34 (20–36)
	*Unbiased* mean absolute percentage				
	Error (%)				
	All (clinical)	1	34	3	37 (36–38)
	Root mean square percentage error (%)				
	All (pharmacogenetic)	1	42	5	53 (37–99)
Bias	Mean prediction error (mg/d)^f^				
	All	17	0.01 (−0.28–0.60)	144	−0.20 (−3.94–1.80)
	Pharmacogenetic	9	−0.10 (−0.28–0.48)	140	−0.20 (−3.94–1.80)
	Clinical	8	0.04 (0.01–0.60)	4	−0.59 (−1.01–0.27)
	Mean percentage prediction error (%)^f^				
	All (pharmacogenetic)	3	4 (3–6)	26	22 (2–76)
	Logarithm of the accuracy ratio‐derived (%)				
	All (clinical)	1	<1	3	8 (4–13)
Clinical relevance	Patients with predicted dose within 20% of actual (%)				
	All	132	48 (10–98)	245	43 (0–80)
	Pharmacogenetic	95	50 (30–98)	231	42 (0–80)
	Clinical	37	47 (10–87)	14	48 (26–63)
	Patients with predicted dose within 1 mg/d of actual (%)				
	All	14	63 (34–92)	47	42 (17–83)
	Pharmacogenetic	12	63 (34–92)	34	42 (17–83)
	Clinical	2	62 (36–87)	13	42 (22–70)

^a^ *N* represents the number of algorithms for which the respective measures were explored and reported. For algorithm development, both development and internal validation cohorts were included, if both reported, although the algorithm was still counted as 1. Results in figures were included if a numerical value was extractable.

^b^ Also called the coefficient of determination. For the development cohort, adjusted values used, when reported.

^c^ The highest *R* ^2^ reported in Pavani^29^ as 94%/96%.

^d^ From clinical algorithms. For algorithm development, this also includes pharmacogenetic algorithms that reported *R* ^2^ contributions of clinical factors only.

^e^ Includes 9 studies reporting median absolute error.

^f^ In some studies (e.g. Botton,^30^ You,^31^ Tan,^32^ Biss,^33^ Zhou,^34^ Lin,^35^ Xie^36^) these performance measures were unclear or inconsistent with their definitions (if available) and/or reported values, in which case a best guess was made. For example, a negative mean absolute error was likely to be a mean prediction error.

A consideration of the race‐specific proportions in each stratified analysis (Tables S10–S13) should be made when interpreting the race‐stratified performances. For example, for 24 studies that included at least 5% Black patients, the proportion of warfarin dose variability that can be attributed to *VKORC1* is 23%. However, these 24 studies on average included a median of only 13% (range 5–100%) Black patients. When only the 3 studies that included only Black patients are considered, the median *VKORC1* partial *R* ^2^ becomes 9% (range 7–10%). These partial *R* ^2^ values should also be cautiously interpreted since different computation approaches yield different results (Figure S1).

Regarding the precision (predictive accuracy) and bias measures, the most reported measures were the mean absolute and mean prediction errors, respectively, being reported 137 (32%) and 17 (4%) times (algorithm development) and 222 (46%) and 144 (31%) times (external validations). The median mean absolute errors for the algorithm development and external validations were respectively 1.23 and 1.20 mg/d, with Asians (0.70 and 0.96 mg/d) and Whites (1.29 and 1.30 mg/d) having lower values when compared to Blacks (1.55 and 1.39 mg/d). The preferred precision and bias measures (Table S4) were underreported, each being reported only once and thrice for algorithm development and external validation, respectively. Clinical relevance and other fit accuracy, precision and bias measures are detailed in Tables 2 and S17.

Because most studies reported *R* ^2^ (a fit accuracy measure), we carried out a *posthoc* correlation analysis and included the studies that reported both the *R* ^2^ and a precision accuracy measure to determine whether *R* ^2^ could be a good proxy of predictive accuracy. For this purpose, we used the mean absolute error as the predictive accuracy measure because it was the most reported (its limitations as a predictive accuracy measure (Table S4) not withstanding). A total of 216 algorithm developments and external validations reported both these 2 measures, with lower mean absolute errors being moderately associated with higher *R* ^2^ values (Pearson's product–moment correlation coefficient −0.390 [95% confidence intervals −0.494 to −0.274], Figure S4).

Tables 2 and S17 include the performance measures stratified according to whether algorithms were clinical or pharmacogenetic (direct comparisons are available for some algorithm pairs in Tables S5 and S7). To directly compare the performances of algorithms stratified according to the modelling technique and time of application (dose initiation or dose revision), we summarized the studies that, using the same dataset, included at least 2 algorithms that differed in these 2 characteristics. As expected, dose revision algorithms generally performed better than dose initiation algorithms (Table S18). Multiple linear regression performed comparable to or even better than many other machine learning techniques (Table S19). Although pharmacokinetic/pharmacodynamic algorithms (fitted using nonlinear mixed effect modelling) performed better than other algorithms, this is mainly attributable to their dose revision aspects (i.e. when used for dose initiation, performance was comparable). However, the numbers of direct comparisons were few, and the performance metrics used were probably suboptimal (Table S4).

### 3.4 Risk of bias

We focused on the assessment of the risk of bias in the analysis domain (Tables S3, S4, S6, S7 and S20). During algorithm development, most developments had the number of participants per candidate predictor variable ≥20 (*n* = 203, 47%), did not provide information on the handling of continuous and categorical predictors (*n* = 291, 67%), probably included all enrolled participants in the analysis (*n* = 229, 53%) and did not provide information on the handling of participants with missing data (*n* = 233, 54%; Table S20). Additionally, many algorithm developments relied on univariable (*n* = 204, 47%) and multivariable (*n* = 208, 48%) analysis during predictor selection, did not appropriately evaluate algorithm performance (*n* = 232, 54%), did not account for model overfitting and optimism in algorithm performance (*n* = 300, 69%), and did not provide enough information to assess whether predictors and their assigned weights in the final algorithms corresponded to the results reported in the multivariable analysis (*n* = 220, 51%). Consequently, only 1 (<1%) algorithm was rated as having a low risk of bias (unclear *n* = 26, 6%; high *n* = 406, 94%).

By contrast, most external validations included at least 100 participants with stable dose (*n* = 329, 68%), all probably appropriately handled continuous and categorical predictors (*n* = 481, 100%), mostly analysed all enrolled participants (*n* = 309, 64%) although many did not provide enough information on the handling of participants with missing data (*n* = 293, 61%). Most (*n* = 273, 57%) reported the precision measures we considered appropriate for this review, although only 8 (<2%) were rated as having a low risk of bias (unclear *n* = 97, 20%; high *n* = 376, 78%).

Although we did not focus on the risk of bias in the participant, predictors and outcome domains, the key risk of bias concerns in these domains are reported in Tables S6 (algorithm development) and S7 (external validation). Of note, despite large variability, most algorithm developments (*n* = 386, 89%) and external validations (*n* = 433, 90%) provided stable dose definitions and/or referenced previous publications in which the same were provided. Lastly, for algorithm development, we also explored whether the outcome of stable dose was transformed during analysis since outcome transformation may affect the bias of the algorithm (Asiimwe, unpublished data). Most algorithm developments (*n* = 228, 53%) did not incorporate any form of transformation, while 102 (24%) and 85 (20%) algorithm developments, respectively, incorporated logarithmic and square root transformations for reasons detailed in Table S21 (for 18 [4%] algorithms, information was not available). The most common reason provided was to normalize dose and limit heteroscedasticity (*n* = 120, 64%) with only 3 (2%) studies reporting clinical considerations as justification.

## 4 DISCUSSION

To facilitate a literature search of warfarin dosing algorithms by clinicians, guideline developers and/or policymakers, we have provided a comprehensive summary of existing algorithms (*n* = 433), external validations (*n* = 481) and clinical utility assessments (*n* = 52) as well as described the populations for which they were developed. Using a threshold of at least 5% inclusion in a dataset, most algorithms were developed in Asians (49% of developed algorithms) and Whites (43%). Whereas 28% of the development datasets included Blacks, this group was under‐represented, the median percentage of Black participants in those datasets being only 14% (Asians and Whites, by contrast, comprised 100 and 90% [medians] of their corresponding algorithm development datasets). Similar trends were observed in the external validations and clinical utility assessments, with these results echoing previous reports of underrepresentation of minority groups.^6^, ^10^ The IWPC^23^ population categories were reported in the main results. As a result, Hispanic Caucasians were grouped with European Caucasians, sub‐Saharan Blacks with African Americans, Indians with Han‐Chinese, multiple sub‐populations under Mixed/Other etc.—groupings that simplify results' presentation but may be inaccurate in the context of personalized medicine. We therefore included locations (countries) from where the trial populations were recruited to serve as proxies for these sub‐populations, and further disparities were revealed. For example, despite 28% of the algorithm developments, 24% of the external validations and 31% of the clinical utility assessments including at least 5% Blacks, <1% algorithm developments, <1% external validations and none of the clinical utility assessments were conducted in sub‐Saharan Africa, results that re‐affirm our previous report that very few studies are conducted in Africa.^37^

Nonstatistical methods including clinical reasoning and literature support are recommended when deciding which candidate predictors to omit, combine or include during multivariable modelling.^12^ To make it easier for those developing new or updating existing algorithms, we also summarised the clinical, demographic, environmental, and genetic factors that are commonly included in warfarin dosing algorithms. The 4 most common demographic/clinical/environmental predictors were age, concomitant medications such as amiodarone, weight and sex being included in 93, 62, 53 and 32% of the 433 algorithms, respectively. By contrast, *CYP2C9*, *VKORC1*, and *CYP4F2* variants were respectively included in 95, 92 and 27% of the 346 pharmacogenetic algorithms. All these are well‐established predictors whose mechanisms of action have been previously extensively reported.^7^, ^38^ In line with the ethnicity‐specific differences in minor allele frequencies of the various genetic variants, population‐specific differences were observed. For instance, CYP2C9*2 is almost absent in some Asian populations^39^ and it was included in only 29% of Asian algorithms that included the *CYP2C9* gene (*n* = 153; compared to being included in 92% of 153 White algorithms, 96% of 99 Black algorithms and 95% of 64 Mixed/Other algorithms). Other *CYP2C9* variants (such as *5, *6, *8 and *11) were mostly included in Black (36% of 99) and Mixed/Other (30% of 64) algorithms and less frequently in Asian (5% of 153) and White (19% of 153) algorithms. Despite a higher inclusion of these other *CYP2C9* variants in studies employing at least 5% Blacks, 36% may still be a low figure given the importance of these African‐specific variants.^37^ When undertaking multivariable modelling, other population‐ and/or clinical setting‐specific considerations such as availability and cost of predictors should also always be considered.^12^

Our third objective was to evaluate the performances of these algorithms. As reported previously,^40^ the coefficient of determination (*R* ^2^) was the most common performance measure (reported in 75% of algorithm developments and 54% of external validations). Based on *R* ^2^, the median contribution of clinical factors (20%) and *VKORC1* (25%) was similar to previous estimates^7^, ^38^ although *CYP2C9*’s contribution (7%) was lower (previously estimated at 12^38^ and 15%^7^). Among the first of 2 key cautions is, like for all the other performance measures, these summary estimates were descriptive in nature since we did not conduct a formal quantitative synthesis, which with the preferred measures (Table S4) and methods (such as individual participant data meta‐analysis^41^) is possible. Because of the descriptive nature of the study, different algorithms using the same or overlapping datasets was also of little concern. The second cautionary warning is that *R* ^2^ is a fit accuracy and not a prediction accuracy measure, the former of which is of less relevance when evaluating the value of prediction algorithms.^42^, ^43^, ^44^ For example, fit accuracy measures will mostly focus on the correct relative ordering of the dose predictions while predictive accuracy measures will also require that these predictions be close to the doses actually required by the patients. During a *posthoc* correlation analysis aimed at determining if *R* ^2^ could be a good proxy of 1 of the predictive accuracy measures (the mean absolute error, MAE), a moderate correlation coefficient (−0.39) was observed, which further questions the use of fit‐accuracy measures given that predictive accuracy measures are available. Among the predictive accuracy distance measures (Table S4), the MAE is preferred to the [root] mean squared error mainly because it is less sensitive to outliers^45^—and this would be an additional reason to prioritize it over *R* ^2^, which is also highly sensitive to outliers. For example, the exclusion of only 1 outlier (<0.1% of 1010 participants) in the IWPC internal validation cohort improved the performance of the pharmacogenetic algorithm from an *R* ^2^ of 33 to 43%^23^ while in Langley's study (*n* = 75), the *R* ^2^ increased from 9 to 31% when 2 outliers were excluded.^46^ To reiterate and for the reasons above, all the reported *R* ^2^ values, especially those that approach 100%, should be interpreted cautiously. Despite the mean absolute error being preferable to the above‐mentioned measures, it remains a distance measure whose limitations we detail in Table S4. In the same table, we describe an unbiased predictive accuracy ratio measure (derived from the logarithm of the ratio of the predicted dose to the actual dose^43^, ^47^), which we consider to be most appropriate. Unfortunately, and excluding our own study (Asiimwe, unpublished data), this measure was not used. We conducted some comparisons in performance based on populations, modelling techniques and the time an algorithm is applied but these results should again be cautiously interpreted because few studies reported direct comparisons and the performance measures used were likely to be inappropriate. For example, it may be misleading to conclude that Asians are better dosed compared to Whites or Blacks based on mean absolute error (or any distance measure that ignores the actual dose required by a patient) since as explained in Table S4, an MAE of 1 mg/d in a small value (e.g. 2 mg/d) may be clinically more important than a larger error (e.g. 2 mg/d) in an even larger value (e.g. 5 mg/d). With these precautions in mind, multiple linear regression (the most commonly used technique) seemed to perform comparable or even better than other supervised machine learning techniques as previously observed.^23^ A further understanding of these other techniques, and a more thorough comparison with multiple linear regression, is nevertheless recommended. For instance, artificial neural networks can capture very complex relationships,^48^ while pharmacokinetic/pharmacodynamic‐based techniques model both temporal and quantitative aspects of warfarin response and do not exclude unstable patients,^40^ which may be beneficial to warfarin dosing.

Lastly, we assessed the risk of bias of existing algorithms with focus on the analysis domain. Less than 2% of both algorithm developments and external validations had a low risk of bias. Only our study was ranked as having a low risk of bias during algorithm development and although this could be because of self‐evaluation, we mainly attribute it to following the TRIPOD (Transparent Reporting of a multivariable prediction model for Individual Prognosis Or Diagnosis) guidelines,^49^ which if followed correctly would result in a low risk of bias rating with the risk of bias assessment tool that we used. It is of concern that although the TRIPOD guidelines were published in 2015, none of the other 163 studies reported from 2016 onwards refer to its use. In the context of not adhering to current methodological recommendations, warfarin dosing algorithms may not be unique.^50^, ^51^ The consequences of most of the design flaws have been previously described in detail.^52^ One key issue that has received less attention is data transformation (done in 44% of the algorithm developments). As discussed by Keene,^53^ we also discourage data‐driven decisions to transform or not and recommend that the logarithmic transformation be preferred because it produces a proportional/multiplicative scale that is clinically relevant and easy to interpret.^53^, ^54^ A slightly more complex method to fit nontransformed dose using a proportional/multiplicative scale is to estimate the parameters of a linear algorithm using nonlinear (log–log) modelling^43^, ^47^ as we previously did (Asiimwe, unpublished data).

In agreement with the CPIC guidelines,^1^ we recommend the IWPC^23^ and Gage^14^ clinical and pharmacogenetic algorithms since these have been the most externally validated and clinically assessed algorithms. Specifically, clinical utility assessments have concluded that they are better than fixed dose‐initiation approaches.^15^, ^16^, ^17^, ^18^, ^19^, ^20^, ^21^, ^22^ Recent debate has mainly focused on whether pharmacogenetic‐guided dosing strategies are better than clinical‐guided strategies with randomized controlled trials such as Kimmel *et al*.,^55^ Pirmohamed *et al*.^56^ and Gage *et al*.^57^ providing conflicting results—we neither quantitatively synthesized (both benefit and safety) nor assessed the risk of bias of the clinical utility studies since we felt these have been previously explored in more detail in several systematic reviews and meta‐analyses.^15^, ^16^, ^17^, ^18^, ^19^, ^20^, ^21^, ^22^

In addition to the CPIC guidance, before using these or other algorithms, clinicians, guideline developers and/or policymakers are reminded to ensure their applicability to their respective populations. For example, the above pharmacogenetic algorithms, despite including Black patients, may not be appropriate for Blacks because they exclude important Black‐specific genetic variants. Clinicians are also reminded that other numerous dosing algorithms that were not assessed in this review exist and may be more appropriate depending on the clinical setting. For example, we excluded many algorithms that rely only on current dose and measured INR levels to make this review more manageable, while the Food and Drug Administration dosing table was excluded because the methods used to derive this table are not publicly available. Nevertheless, these algorithms are less likely to perform better than those that incorporate predictors additional to existing dose/measured INR while the Food and Drug Administration table has been assessed in several studies^58^, ^59^, ^60^, ^61^, ^62^, ^63^, ^64^ and its performance is not better than the pharmacogenetic algorithms we have reported here.

In addition to using heterogeneous and nonspecific racial categories, presenting mainly descriptive results, and excluding algorithms that rely only on current dose and measured INR levels, our study had other limitations. Specifically, we did not include non‐English articles, which could have affected geographical representation. For example, we excluded 9 Chinese studies during title/abstract screening and non‐English articles are less likely to be indexed in MEDLINE.^65^ Although we tried identifying other studies through reference list searching, using only the MEDLINE database also limited the number of studies that we could include in this review. Lastly, we relied on single‐reviewer extraction; a second reviewer, nevertheless, confirmed consistency based on a random selection of 10% of the included papers.

For further research, novel/existing algorithms may need to be developed or updated and externally validated following the recommended guidelines such as TRIPOD.^49^ More attention needs to be paid to under‐represented populations such as minority ethnic groups and children (only 3% developed algorithms) to reduce health disparities. Moreover, although newer directly acting oral anticoagulants have been developed, warfarin is likely to remain the preferred choice for some of these groups.^66^

In conclusion, this systematic review provides a comprehensive summary of the algorithms available for different populations and their associated covariates (demographic, clinical, environmental and/or genetic), performances and risk of bias from these algorithms. Most of these algorithms have been developed for Asians and Whites, most have neither been externally validated nor assessed for clinical utility and either have a high or unclear risk of bias, which makes their reliability for clinical use uncertain. Future research should focus on developing prediction algorithms for under‐represented populations and externally validating and assessing the clinical utility of these and already existing algorithms. Algorithm development and assessment should follow current methodological recommendations to improve reliability and applicability.

## COMPETING INTERESTS

M.P. receives other research funding from various organisations including the EU Commission. He has also received partnership funding for the following: MRC Clinical Pharmacology Training Scheme (co‐funded by MRC and Roche, UCB, Eli Lilly and Novartis); a PhD studentship jointly funded by EPSRC and Astra Zeneca; and grant funding from Vistagen Therapeutics. He has also unrestricted educational grant support for the UK Pharmacogenetics and Stratified Medicine Network from Bristol‐Myers Squibb and UCB. He has developed an HLA genotyping panel with MC Diagnostics, but does not benefit financially from this. None of these of additional funding sources have been used for the current paper.

## CONTRIBUTORS

I.G.A., E.J.Z., A.L.J. and M.P. conceived and designed the study. I.G.A. and R.O. performed data search, screening and analysis. A.L.J. and M.P. supervised the study. I.G.A. wrote the manuscript. A.L.J. and M.P. interpreted the results and made manuscript revisions. All authors read and approved the final manuscript.

## Supporting information


**TABLE S1** Preferred Reporting Items for Systematic Reviews and Meta‐Analyses: The PRISMA Statement^1^
**TABLE S2** MEDLINE search strategy
**TABLE S3** Tailoring PROBAST (Prediction model Risk Of Bias ASsessment Tool)^2^ to the systematic review
**TABLE S4** Relevant performance measures
**TABLE S5** Clinical and pharmacogenetic algorithms
**TABLE S6** Quality assessment (algorithm development)
**TABLE S7** External validations
**TABLE S8** Clinical utility assessments^a^
**TABLE S9** Algorithms that have been externally validated or assessed for clinical utility at least once^a^
**TABLE S10** Summary characteristics of algorithm developments, external validations and clinical utility assessments for studies that included at least 5% White participants
**TABLE S11** Summary characteristics of algorithm developments, external validations and clinical utility assessments for studies that included at least 5% Asian participants
**TABLE S12** Summary characteristics of algorithm developments, external validations and clinical utility assessments for studies that included at least 5% Black participants
**TABLE S13** Summary characteristics of algorithm developments, external validations and clinical utility assessments for studies that included at least 5% Mixed/Other participants
**TABLE S14** Algorithms that have been externally validated or assessed for clinical utility at least once^a^ (studies that included at least 5% Whites)
**TABLE S15** Algorithms that have been externally validated or assessed for clinical utility at least once^a^ (studies that included at least 5% Asians)
**TABLE S16** Algorithms that have been externally validated or assessed for clinical utility at least once^a^ (studies that included at least 5% Blacks or 5% Mixed/Other participants)
**TABLE S17** Performance measures stratified by race
**TABLE S18** Dose initiation (a priori) *vs* dose revision (a posteriori) algorithms
**TABLE S19** A comparison of various modelling techniques
**TABLE S20** Summary results of the risk of bias assessment (analysis domain)
**TABLE S21** Reasons for logarithmic or square‐root transformations (n = 187).
**FIGURE S1** Computing *R* ^2^ and partial *R* ^2^ using set representation.
**FIGURE S2** A breakdown of the research articles included in the systematic review.
**FIGURE S3** Most commonly included predictors (Figure 2) stratified according to the 4 population categories.
**FIGURE S4** Correlation between a fit accuracy measure (*R* ^2^) and a predictive accuracy measure (mean absolute error).

## Data Availability

All relevant material is provided in the supplementary material.
