# Supplementary material for: Warfarin dosing algorithms: A systematic review
Source: Br J Clin Pharmacol. 2020 Nov 18;87(4):1717–29. doi: 10.1111/bcp.14608 (PMC8056736; doi:10.1111/bcp.14608)
Supplement: Supplementary file 1 — TABLE S1 Preferred Reporting Items for Systematic Reviews and Meta‐Analyses: The PRISMA Statement1 TABLE S2 MEDLINE search strategy TABLE S3 Tailoring PROBAST (Prediction model Risk Of Bias ASsessment Tool)2 to the systematic review TABLE S4 Relevant performance measures TABLE S5 Clinical and pharmacogenetic algorithms TABLE S6 Quality assessment (algorithm development) TABLE S7 External validations TABLE S8 Clinical utility assessmentsa TABLE S9 Algorithms that have been externally validated or assessed for clinical utility at least oncea TABLE S10 Summary characteristics of algorithm developments, external validations and clinical utility assessments for studies that included at least 5% White participants TABLE S11 Summary characteristics of algorithm developments, external validations and clinical utility assessments for studies that included at least 5% Asian participants TABLE S12 Summary characteristics of algorithm developments, external validations and clinical utility assessments for studies that included at least 5% Black participants TABLE S13 Summary characteristics of algorithm developments, external validations and clinical utility assessments for studies that included at least 5% Mixed/Other participants TABLE S14 Algorithms that have been externally validated or assessed for clinical utility at least oncea (studies that included at least 5% Whites) TABLE S15 Algorithms that have been externally validated or assessed for clinical utility at least oncea (studies that included at least 5% Asians) TABLE S16 Algorithms that have been externally validated or assessed for clinical utility at least oncea (studies that included at least 5% Blacks or 5% Mixed/Other participants) TABLE S17 Performance measures stratified by race TABLE S18 Dose initiation (a priori) vs dose revision (a posteriori) algorithms TABLE S19 A comparison of various modelling techniques TABLE S20 Summary results of the risk of bias assessment (analysis domain) TABLE S21 Reasons for log [file BCP-87-1717-s001.docx]

**Warfarin dosing algorithms: a systematic review**

Innocent G. Asiimwe^1^, Eunice J. Zhang^1^, Rostam Osanlou^1^, Andrea L. Jorgensen^2,3^, and Munir Pirmohamed^1,3^

^1^The Wolfson Centre for Personalized Medicine, MRC Centre for Drug Safety Science, Department of Pharmacology and Therapeutics, Institute of Systems, Molecular and Integrative Biology, University of Liverpool. ^2^Department of Biostatistics, Institute of Population Health Sciences, University of Liverpool. ^3^These authors contributed equally.

**Table of contents**

**Supplementary Tables……………………………………………………………………………….………….. 2**

**Supplementary Figures………………………………………………………………….………….………..… 107**

**Supplementary References……………………………………………………….……………………….…. 111**

**Supplementary Tables**

**Table S1*.* Preferred Reporting Items for Systematic Reviews and Meta-Analyses: The PRISMA Statement^1^**

| **Section and topic** | **Item No** | **Checklist item** | **Section and topic** |
| --- | --- | --- | --- |
| TITLE | | |  |
| Title | 1 | Identify the report as a systematic review, meta-analysis, or both. | Title page |
| ABSTRACT | | |  |
| Structured summary | 2 | Provide a structured summary including, as applicable: background; objectives; data sources; study eligibility criteria, participants, and interventions; study appraisal and synthesis methods; results; limitations; conclusions and implications of key findings; systematic review registration number. | Abstract |
| INTRODUCTION | | |  |
| Rationale | 3 | Describe the rationale for the review in the context of what is already known. | Introduction, paragraphs 1, 2 and 3 |
| Objectives | 4 | Provide an explicit statement of questions being addressed with reference to participants, interventions, comparisons, outcomes, and study design (PICOS). | Introduction, paragraph 3 |
| METHODS | | |  |
| Protocol and registration | 5 | Indicate if a review protocol exists, if and where it can be accessed (e.g., Web address), and, if available, provide registration information including registration number. | Methods (Search strategy and selection criteria) |
| Eligibility criteria | 6 | Specify study characteristics (e.g., PICOS, length of follow-up) and report characteristics (e.g., years considered, language, publication status) used as criteria for eligibility, giving rationale. | Methods (Search strategy and selection criteria) |
| Information sources | 7 | Describe all information sources (e.g., databases with dates of coverage, contact with study authors to identify additional studies) in the search and date last searched. | Methods (Search strategy and selection criteria) |
| Search | 8 | Present full electronic search strategy for at least one database, including any limits used, such that it could be repeated. | Table S2 |
| Study selection | 9 | State the process for selecting studies (i.e., screening, eligibility, included in systematic review, and, if applicable, included in the meta-analysis). | Methods (Search strategy and selection criteria) |
| Data collection process | 10 | Describe method of data extraction from reports (e.g., piloted forms, independently, in duplicate) and any processes for obtaining and confirming data from investigators. | Methods (Data extraction and quality assessment) |
| Data items | 11 | List and define all variables for which data were sought (e.g., PICOS, funding sources) and any assumptions and simplifications made. | Methods (Data extraction and quality assessment) |
| Risk of bias in individual studies | 12 | Describe methods used for assessing risk of bias of individual studies (including specification of whether this was done at the study or outcome level), and how this information is to be used in any data synthesis. | Methods (Data extraction and quality assessment) |
| Summary measures | 13 | State the principal summary measures (e.g., risk ratio, difference in means). | Methods (Search strategy and selection criteria) |

**Table S1. Continued**

| **Section and topic** | **Item No** | **Checklist item** | **Section and topic** |
| --- | --- | --- | --- |
| Synthesis of results | 14 | Describe the methods of handling data and combining results of studies, if done, including measures of consistency (e.g., I^2^) for each meta-analysis. | Methods (Data synthesis) |
| Risk of bias across studies | 15 | Specify any assessment of risk of bias that may affect the cumulative evidence (e.g., publication bias, selective reporting within studies). | Methods (Data synthesis) |
| Additional analyses | 16 | Describe methods of additional analyses (e.g., sensitivity or subgroup analyses, meta-regression), if done, indicating which were pre-specified. | Methods (Sensitivity and subgroup analyses) |
| RESULTS | | |  |
| Study selection | 17 | Give numbers of studies screened, assessed for eligibility, and included in the review, with reasons for exclusions at each stage, ideally with a flow diagram. ` | Figure 1 |
| Study characteristics | 18 | For each study, present characteristics for which data were extracted (e.g., study size, PICOS, follow-up period) and provide the citations. | Tables S5–S8 |
| Risk of bias within studies | 19 | Present data on risk of bias of each study and, if available, any outcome level assessment (see item 12). | Tables S6, S7 and S20 |
| Results of individual studies | 20 | For all outcomes considered (benefits or harms), present, for each study: (a) simple summary data for each intervention group (b) effect estimates and confidence intervals, ideally with a forest plot. | Tables S5–S8 |
| Synthesis of results | 21 | Present results of each meta-analysis done, including confidence intervals and measures of consistency. | Not done |
| Risk of bias across studies | 22 | Present results of any assessment of risk of bias across studies (see Item 15). | Not done |
| Additional analysis | 23 | Give results of additional analyses, if done (e.g., sensitivity or subgroup analyses, meta-regression [see Item 16]). | Table S10-S17 |
| DISCUSSION | | |  |
| Summary of evidence | 24 | Summarize the main findings including the strength of evidence for each main outcome; consider their relevance to key groups (e.g., healthcare providers, users, and policy makers). | Discussion |
| Limitations | 25 | Discuss limitations at study and outcome level (e.g., risk of bias), and at review-level (e.g., incomplete retrieval of identified research, reporting bias). | Discussion |
| Conclusions | 26 | Provide a general interpretation of the results in the context of other evidence, and implications for future research. | Discussion |
| FUNDING | | |  |
| Funding | 27 | Describe sources of funding for the systematic review and other support (e.g., supply of data); role of funders for the systematic review. | Funding section. |

**Table S2*.* MEDLINE search strategy**

| **Number** | **Search Term** |
| --- | --- |
| 1 | Warfarin.mp |
| 2 | s-warfarin.mp |
| 3 | r-warfarin.mp |
| 4 | Coumarin.mp |
| 5 | Coumadin.mp |
| 6 | algorithm$.mp |
| 7 | regression$.mp |
| 8 | model$.mp |
| 9 | prediction$.mp |
| 10 | dose response$.mp |
| 11 | dose predict$.mp |
| 12 | dose calculation$.mp |
| 13 | dosing$.mp |
| 14 | Bayes$.mp |
| 15 | drug response$.mp |
| 16 | validat$.mp |
| 17 | prognostic$.mp |
| 18 | Multivaria$.mp |
| 19 | 1 or 2 or 3 or 4 or 5 |
| 20 | 6 or 7 or 8 or 9 or 10 or 11 or 12 or 13 or 14 or 15 or 16 or 17 or 18 |
| 21 | 19 and 20 |

**Table S3. Tailoring PROBAST (Prediction model Risk Of Bias ASsessment Tool)^2^ to the systematic review**

| **Domain^a^** | **Signalling question** | **Concerns** | **Assessment** | **Comments** |
| --- | --- | --- | --- | --- |
| Participants | 1.1. Were appropriate data sources used, e.g., cohort, RCT, or nested case–control study data? | Problems may arise if a study inappropriately includes or excludes participant groups from entering the study. | Yes, for all studies | During screening, only cohort studies, randomised controlled trials and routine care registry studies were included. |
|  | 1.2 Were all inclusions and exclusions of participants appropriate? |  | Yes, for all studies | - This signalling question is answered with respect to an intended target population and our review was not restricted to a particular group of patients. It primarily aimed to summarize the algorithms which are available for all kinds of patient populations. Health care providers, guideline developers, and/or policymakers, who will make use of the results of this review are however reminded to cross-check that the algorithms available for their populations were developed/externally validated in participants with characteristics similar to their target populations.  - Some PKPD models make use of healthy populations. PKPD models were only included if they [also] used warfarin-treated patients receiving a stable dose. |
|  | ROB rating |  | Low | ‘Yes’ answered for all signalling questions. |
| Predictors | 2.1 Were predictors defined and assessed in a similar way for all  participants? | A high risk of bias when predictor definitions and measurements are flawed, inconsistent or influenced by knowledge of the outcome. | Yes/probably yes (unless otherwise stated), for all studies | - Because of the temporal nature of stable dose (i.e. it is achieved days after a patient is initiated on warfarin dose), the predictors usually considered including ‘existing’ doses/INRs for dose-revision models are “blinded to the outcome.”  - Others like gender or genetic factors are stable over time making the time component to be of less concern.  - Even when measured after (e.g. in some development studies), most predictors and the outcome variable, are objective measures that do not require subjective interpretation.  - Where it was felt that the subjective interpretation of a predictor had potential to change the overall ROB rating of a study, or when a study included a predictor that cannot be known at the time when the model is supposed to be used (e.g. INR readings [not baseline] for dose-initiation studies), we explicitly pointed it out and changed the ROB rating. |
|  | 2.2 Were predictor assessments made without knowledge of outcome data? |  |  |  |
|  | 2.3 Are all predictors available at the time the model is intended to be used? |  |  |  |
|  | ROB rating |  | Low (unless otherwise stated) | ‘Yes/probably yes’ answered for all signalling questions (unless otherwise stated) |
| Outcome | 3.1 Was the outcome determined appropriately? | Bias can occur when patients are wrongly classified as having a stable warfarin dose, when the stable warfarin dose is wrongly or inconsistently determined or when its determination is influenced by knowledge of predictors. | Yes/probably yes (unless otherwise stated), for all studies | - There is no generally agreed warfarin dose definition. However, most definitions require stable levels of anticoagulation (i.e. INRs within a given target INR range – an objective measure) over a period during which the warfarin dose is unchanged/does not vary by more than a specified proportion ^3^.  - Although there is a ROB related to selective outcome reporting when a prespecified/standard definition is not used, we considered any study that relied on ≥2 INRs within range to have a low ROB.  - Many studies do not provide an explicit definition but report that patients were stable (based on a specified INR range). Such studies were highlighted, although they were still ranked to have a low ROB.  - The relationship between outcome and predictor is discussed under the ‘predictors’ domain.  - Consequently, all items were marked ‘yes/probably yes’ by default. Any concerns that we felt would change the overall ROB rating of a study (e.g. when it was clear only 1 INR reading was used to determine the stable dose, when stable dose was based on self-reports, when unstable patients were [un]intentionally included in the stable dose analyses etc.) were explicitly pointed out and the ROB rating change to high. |
|  | 3.2 Was a prespecified or standard outcome definition used? |  |  |  |
|  | 3.3 Were predictors excluded from the outcome definition? |  |  |  |
|  | 3.4 Was the outcome defined and determined in a similar way for all  participants? |  |  |  |
|  | 3.5 Was the outcome determined without knowledge of predictor  information? |  |  |  |
|  | 3.6 Was the time interval between predictor assessment and outcome determination appropriate? |  |  |  |
|  | ROB rating |  | Low (unless otherwise stated) | ‘Yes/probably yes’ answered for all signalling questions (unless otherwise stated) |

**Table S3. Continued**

| **Domain^a^** | **Signalling question** | **Concerns** | **Assessment** | **Comments** |
| --- | --- | --- | --- | --- |
| Analysis | 4.1 Were there a reasonable number of participants with the outcome? | Inappropriate or flawed statistical analysis methods can  distort results. | As per PROBAST | - Since our outcome was stable dose and not an event, it was more appropriate to use the total number of study participants per candidate predictor variable (PCV) than the number of events per candidate predictor variable (EPV).  - Candidate predictors were the total predictors considered for statistical-based selection processes (including univariate analyses).  - Some machine learning methods require PCVs in excess of 200^4^ while others such as LASSO^5^ require much less.  - PKPD models commonly employ repeated measurements from the same individual during the dose-titration phase and can therefore require only a small number of participants^6^. However, our focus was on the ability of the PKPD model to predict one endpoint (the stable dose). The PCV was calculated in relation to this one endpoint and therefore the thresholds stated in the PROBAST guideline were used. |
|  | 4.2 Were continuous and categorical predictors handled appropriately? |  | As per PROBAST | -Except that continuous predictors which were not converted into ≥2  categories also had to have been examined for nonlinearity for a ‘Yes/Probably Yes’ rating.  - A consideration that some ML techniques such as artificial neural nets model non-linear relationships was made. |
|  | 4.3 Were all enrolled participants included in the analysis? |  | As per PROBAST | - Note that this is different from signalling question 1.2. However, patients who were excluded during enrolment because of uninterpretable (unclear) findings, outliers or missing predictor data were ranked ‘no/probably no’ for this signalling question.  - ROB may be lower with a very low percentage of excluded participants^7^. However, an acceptable percentage is hard to define as it depends on the exact participants excluded e.g. the exclusion of only one outlier (<0.1% of 1010 participants in the IWPC internal validation cohort) improved the performance of the pharmacogenetic algorithm from a MAE of 8.8 mg/wk and R^2^ of 33% to 8.5 mg/wk and 43% respectively^3^. Therefore, we did not take into consideration the percentage of excluded participants during the ROB assessment. If results of sensitivity analysis reporting the exclusion of patients during analysis are reported, this section was answered ‘Y’ or ‘PY’, otherwise it was answered ‘N’ or ‘PN’. |
|  | 4.4 Were participants with missing data handled appropriately? |  | As per PROBAST | Except that if the study had no explicit mention of methods to handle missing data, it was ranked as ‘No information’. |
|  | 4.5 Was selection of predictors based on univariable analysis avoided? |  | As per PROBAST |  |
|  | 4.6 Were complexities in the data (e.g., censoring, competing risks,  sampling of control participants) accounted for appropriately? |  | Not applicable | Due to the continuous nature of the outcome (all patients included had a stable dose); this is more relevant to binary (or more) events and time-to-event outcomes. |
|  | 4.7 Were relevant model performance measures evaluated  appropriately? |  | As per PROBAST | Theoretical arguments support the precision/predictive accuracy and bias measures derived from the logarithm of the accuracy ratio to be the most appropriate measures (Table S4). To our knowledge, these are a recent introduction to warfarin dosing (Asiimwe, unpublished data) and consensus on their adequacy is yet to be reached. Other precision measures may also be clinically useful despite some limitations, but this will be explored in future work. For this review, we therefore considered the five precision accuracy measures in Table S4 to be appropriate (we acknowledge that some of these may both be inadequate and clinically inappropriate). |
|  | 4.8 Were model overfitting and optimism in model performance  accounted for? |  | As per PROBAST | Some machine learning methods such as LASSO account for overfitting and model optimism during predictor selection. |
|  | 4.9 Do predictors and their assigned weights in the final model correspond to the results from the reported multivariable analysis? |  | As per PROBAST |  |
|  | ROB rating |  | As per PROBAST |  |

^a^Regarding applicability for the three domains (participants, predictors and outcome), this systematic review was broad in terms of included participants (warfarin-treated populations of any ethnicity included), clinical settings (no exclusion criteria with respect to clinical settings or daily practice) and outcome definition (no specific definition required), and hence all studies were deemed to have low applicability concerns (unless otherwise stated e.g. studies that also included some unstable patients). Abbreviations: INR, international normalized ratio; IWPC, international warfarin pharmacogenetics consortium; LASSO, least absolute shrinkage and selection operator; MAE, mean absolute error; PCV, participants per candidate predictor; PKPD, pharmacokinetic pharmacodynamic; R^2^, coefficient of determination; ROB, risk of bias.

**Table S4. Relevant performance measures**

| **Performance measure^a^** | **Description or formula** | **Comments** |
| --- | --- | --- |
| ***Precision/predictive accuracy^b^ (the closer the predicted dose is to the actual dose)^8^*** | | |
| Mean absolute error (MAE) | mean(|predicted dose – actual dose|) | - The MAE is preferred to the MSE and RMSE mainly because it is less sensitive to outliers^9^.  - All are distance measures^10^. A problem with distance measures is that an error such as 1 mg/d may be clinically more important in a small value (e.g. 2 mg/d) than a similar (or even larger) error in a large value (e.g. 10 mg/d)^11^.  - Some studies report the median, rather than mean, absolute error. If the errors are normally distributed (an assumption of linear regression), the median and mean errors should coincide. Since departures from regression assumptions are of little concern^12^ and we anticipated a small proportion of these studies, we considered the median absolute error, when reported, to approximate the mean absolute error. In the detailed tables, the word ‘median’ was added to the former.  - Regression-based methods such as ordinary least squares regression and quantile regression that depend on distance-based measures are likely to produced biased models (high tendency to over-predict)^13^. Transformation of the outcome by computing the logarithm, square-root or square can respectively remove, decrease, or increase this bias (Asiimwe, unpublished data). |
| Mean squared error (MSE) | mean((predicted dose – actual dose)^2) |
| Root mean square error (RMSE) | √MSE |
| Mean absolute percentage error (MAPE) | mean($\frac{\left\vert predicted dose-actual dose \right\vert\times100}{actual dose}$) | - A ratio measure^10^ that makes an absolute error of 1 mg/d in a 2 mg/d patient equivalent to an error of 5 mg/d in a 10 mg/d patient (all errors of 50%).  - However, it is asymmetric in nature (e.g. a low dose patient (1 mg/d) predicted to take 5 mg/d will have an APE of 400% $\left( \frac{\left\vert1-5 \right\vert\times100}{1} \right)$ while a high dose patient (5 mg/wk) predicted to require 1 mg/wk will have an APE of 80% $\left( \frac{\left\vert5-1 \right\vert\times100}{5} \right)$). Consequently, MAPE rewards models that systematically under-predict^13,14^. |
| Unbiased MAPE (derived from the logarithm of the accuracy ratio ^10,13^) (Asiimwe, unpublished data) | (exp(mean(absolute(log(predicted dose/actual dose)))) – 1) $\times$ 100 | - Both a ratio and difference measure^13^, unbiased/symmetric in nature and is easy to interpret clinically.  - Preferred precision (predictive accuracy) measure.  - Non-linear least squares can be used to estimate the parameters of a linear model based on the logarithm of the accuracy ratio^10,13^. |
| ***Bias^b^ (the degree to which the typical prediction is either too high or too low) ^8^*** | | |
| Mean predictive error (MPE) | mean(predicted dose – actual dose) | - Is a distance measure so similar problems to MAE.  - Like other bias measures (see below), MPE cannot be used to assess precision since, for instance, a highly negative error may cancel out a highly positive error producing a MPE that is close to zero. The model will be unbiased, but its individual predictions will be far from the true values (poor predictive accuracy). |
| Bias (derived from the logarithm of the accuracy ratio ^10,13^) (Asiimwe, unpublished data) | (exp(mean(log(predicted dose/actual dose))) – 1) × 100 | - Unbiased/symmetric in nature and easy to interpret clinically.  - Preferred bias measure. |
| ***Fit accuracy (how well the fitted equation fits the data from which it was generated^10^)*** | | |
| Coefficient of determination (R^2^) and related correlation measures such as Pearson’s and Spearman’s rank correlation coefficients. | See Kvalseth ^15^ for eight different R^2^ formulae. | - See Figure S1 on why partial or univariate R^2^ (although reported for some studies) should be cautiously interpreted.  - Also a distance measure^10^.  - Assesses the degree of association between actual and predicted doses^8^. Can be misleading in terms of the actual closeness of predictions to true values – not a good measure for predictive accuracy^8,10,16,17^.  - During reporting, focus was placed on the correlation and squared correlation coefficients. Other fit accuracy measures including the visual inspection of the studentized residual plots and Bland-Altman analysis were only included in the supplementary material. |
| ***Clinical relevance*** | | |
| Percentage of patients with predicted doses with 1 mg/d of the actual dose | sum(as.numeric(c(absolute(actual daily dose – predicted dose) < 1)))*100/number of patients. | - Like for MAE, an error of 1 mg/d may be clinically more important in patients who require lower doses (e.g. Asians) than those who require higher doses (e.g. Blacks). Consequently, in low dose patients, performance (i.e. those within 1 mg/day) may be over-estimated.  - A 1 mg/d error is based on a starting dose of 5 mg/d. However, Asians may require lower starting doses such as 3 mg/d. For these an error of 0.6 mg/d may be similar (i.e. within 20% of the population average starting dose).  - Whether 1 mg/d or 0.6 mg/d (or another value) is used, basing on a population average contradicts the goal of personalized medicine i.e. it is known that even if the average population will require 5 mg/d, many patients will require doses higher or lower than this and using 5 mg/d (or 3 mg/d) as a baseline for this would be inaccurate. |
| Percentage of patients with ideal dose | Ideal dose was defined as the predicted dose within 20% of the actual dose^3^. | - Is a ratio measure and is therefore preferable to the percentage of patients with predicted doses with 1 mg/d of the actual dose.  - Other thresholds e.g. within 15% are also reported, although less commonly (our focus was on the 20% threshold)  - However, it ignores patients outside ideal dose i.e. it does not consider that a patient within 21% of ideal dose is preferable to a patient within 80% of ideal dose. |

**Table S4. Continued.**

| **Performance measure^a^** | **Description or formula** | **Comments** |
| --- | --- | --- |
| ***Clinical safety (patients at high risk of under- or over-anticoagulation)*** | | |
| Quantiles | Using 25^th^/75^th^ (e.g. Li^18^) or 30^th^/70^th^ (e.g. Li^19^) or other quantiles to create risk-groups. | - Can wrongly categorize patients depending on the dose distribution (skewed or not) or extent of homogeneity of a population. For example, Li^19^ used the 25^th^ and 75^th^ quantiles to categorize 15,694 patients (96% Han Chinese) into low dose (≤2.5 mg/day), intermediate dose (2.5 to 3 mg/day) and high dose (≥3 mg/day). This contradicts clinical practice in China, where patients receiving the fixed doses of either 2.5 or 3 mg/day are considered not to be at risk.  -It is also illogical for patients at risk of under- or over-dosing to also receive ideal dose e.g. in Liu et al.^20^ up to 24% and 39% of patients classified as low- and high-dose respectively were also classified as patients receiving ideal dose.  - Also ignores the fact that patients receiving very high doses (e.g. ~5 mg in Asians) will not be at risk if a model that consistently predicts very high doses (e.g. a fixed dose of 5 mg) is used. |
| Bracketing a fixed dose (distance-based) | IWPC^3^ used thresholds of 3 mg/d and 7 mg/d which bracket a fixed dose of 5 mg/d. Other thresholds include 25 mg/wk and 45 mg/wk (bracket a fixed dose of 35 mg/wk or 5 mg/d) (e.g. Zambon^21^), 2 mg/d and 4 mg/d (bracket 3 mg/d) (e.g. Xu^22^), 1.5 mg/d and 4.5 mg/d (bracket 3 mg/d) (e.g. Takeuchi^23^) and 1 mg/d and 4 mg/d (bracket 2.5 mg/d) (e.g. Cha^24^). | - Using a specific fixed dose to define risk uses a ‘one-size fits all’ approach and ignores the aims of personalized medicine. E.g. whereas doses below 3mg/d will present risk to a patient requiring 5 mg/d, doses below 3 mg/d are indeed desirable for patients who are warfarin sensitive. Consequently, the risk profile of any patient is dynamic and will depend on the dosing strategy used.  - As an example, Cho^25^ used a fixed dose of 5 mg/d to create risk categories (Korean patients receiving ≤ 3 mg/d and ≥ 7 mg/d were considered to be at high risk). The use of a wrong fixed dose created misleading categories since Korean patients typically receive doses around 2.5-3 mg/d and therefore not all patients receiving ≤ 3 mg/d should have been considered to be at risk. The IWPC study^3^ which also relied on the 5 mg/d fixed dose is likely to have wrongly categorized most Asians as being high risk patients (which is true with 35 mg/wk, but not with the lower doses commonly used in Asia). Although, lower fixed doses in Asians would be more appropriate, using risk categories based on them would still ignore the fact that some Asians require very high doses and that such Asians would not be at risk if a model that is right for them (e.g. consistently predicts high doses) is used. |
| Bracketing a predicted dose (distance-based) | For example, Gage^26^ considered patients with dosing errors >2 mg/d to be a risk of significant overdose while Lin^27^ considered those with errors ≤-2mg/d or ≥ 2 mg/d to be severely under- or over-predicted. | - Whereas this approach circumvents the issue with using a specific fixed dose (above), it is a distance-based measure which ignores the fact that an overprediction of 3 mg/d in a patient who requires 1 mg/d will be clinically less important that the same overprediction in a patient who requires 10 mg/d (see precision measures such as MAE). |
| Bracketing predicted dose (ratio-based) | For example, Lin^27^ considered those with relative errors ≤-50% or ≥ 100% to be severely under- or over-predicted while we (Asiimwe, unpublished data) recently defined risk as having too high (≥40%) or too low (≤40%) an actual dose as compared to the predicted dose. | - This circumvents the issues with both using a specific fixed dose and distance-based measures – and is therefore preferable in our opinion.  - However, the cut-off points are subject to debate. For instance, Lin^27^ do not explain why they chose 50% and 100% as cut-off points and why they used a high cut-off point for over-prediction.  - For our case, the 40% threshold was used to be consistent with the majority of other studies that relied on a difference of 2 mg/d based on a fixed dose of 5 mg/d.  - The clinical safety measures were not assessed in this systematic review. However, to facilitate future research, the different risk thresholds used were reported as part of the quality assessment of algorithm development. |
| ***Others*** | | |
| As diagnostic tests | See PROBAST^7^ Box 4. | - Include measures such as calibration, discrimination, sensitivity and specificity.  - Not applicable to this review which focuses on the final dose a patient receives, not (for example) whether the patient receives a high or low dose. |
| Visual predictive checks | See Hamberg^28^. | - Graphically assesses if algorithm simulations can “*reproduce both the central trend (the median curve) and the variability (outer percentiles) in the observed data when plotted against an independent variable (e.g., time, dose or age)*,"^28^.  - More relevant to repeated measurements from the same individual during dose-titration. |

^a^These measures require only predicted and actual doses so can be computed for all types of algorithms including the pharmacokinetic/ pharmacodynamic models that commonly employ visual predictive checks. ^b^The prediction error (predicted dose minus/divided by actual dose) measures bias while the absolute or squared values of the prediction error measure precision^8^. Both values are usually accompanied by 95% confidence-intervals although these are nor reported in this review. Measures such as number needed to genotype which compare two models were not considered^3^.

**Table S5. Clinical and pharmacogenetic algorithms**

| **#** | **Algorithm** | **Sub-type** | **Country** | **Indication** | **Target INR** | **N** | **Ethnicity (%)** | | | | **Male %** | **Age, yrs (mean ± SD or median, IQR)** | **Clinical parameters** | **Genetic parameters** | **Equation^a^** | **R2^b^** | | | | **MAE (mg/d)** | **Other** | **ROB** | **# EV** | **# CUA** |
| --- | --- | --- | --- | --- | --- | --- | --- | --- | --- | --- | --- | --- | --- | --- | --- | --- | --- | --- | --- | --- | --- | --- | --- | --- |
|  |  |  |  |  |  |  | **W** | **A** | **B** | **M** |  |  |  |  |  | **All** | **Cl** | **VKORC1** | **CYP2C9** |  |  |  |  |  |
| 1 | Tabrizi 2002^29^ | Ini-pgx | USA | AF, DVT, HV, CAD, other | 1.8–3.5 | 153 | 78 | - | 22 | - | 56 | 59 ± 15 | Age, weight | CYP2C9*2, *3 | Ln dose (mg/wk) = 3.90 – 0.11 (age) + 0.41 (weight × 10^-2) – 0.23 (CYP2C9*2) – 0.44 (CYP2C9*3) | 26 | 14 | - | - | - | - | High | - | - |
| 2 | Gage 2004^26^ | Ini-pgx | USA | AF, DVT, PE, stroke, HV, other | Multiple | 369 | 71 | - | 29 | - | 56 | 69 ± 13 | Age, BSA, gender, amio, sim, target INR, race | CYP2C9*2, *3 | Ln dose (mg/d) = 0.385 − 0.0083 (age) + 0.498 (BSA) – 0.208 (CYP2C9*2) − 0.350 (CYP2C9*3) − 0.341 (amio) + 0.378 (target INR) − 0.125 (sim) − 0.113 (race) − 0.075 (female) | 37 | - | - | 10 | 1.2 | 51% within 1 mg/d | High | 5 | 1 |
| 3 | Hillman 2004^30^ | Ini-pgx | USA | AF, DVT, HV, other | - | 453 | Y | - | - | - | 57 | 71 ± 11 | Age, BSA, HV, DM | CYP2C9*2, *3 | Ln dose (mg/wk) = 1.654 + 0.429 (CYP2C9*2 or *3) − 0.274 (CYP2C9*3) + 0.221 (HV) − 0.035 (age) + 2.886 (BSA) + 0.936 (DM) + 0.0002 (age^2) − 0.604 (BSA^2) − 0.014 (age × DM) – 0.305 (BSA × CYP2C9*2 or *3) + 0.129 (DM × CYP2C9*2 or *3) | 34 (log dose) | - | - | - | - | - | High | 3 | 1 |
| 4 | Kamali 2004^31^ | Ini-pgx | UK | AF, DVT, PE, HV, other | 2.0–3.0 | 119 | Y | - | - | - | 55 | 70 ± 12 | Age | CYP2C9*2, *3 | Dose (mg/d) = 8.05 – 0.06 (Age) – 1.12 (CYP2C9*3) | 20 | 17 | - | 6 | - | - | High | - | - |
| 5 | Herman 2005^32^ | Ini-pgx | Slovenia | AF, DVT, HT, other | 2.0–3.5 | 187 | Y | - | - | - | 50 | 72 ± 9 | Age, weight, enzyme inducers | CYP2C9*2, *3 | Ln dose (mg/d)) = 2.161 – 0.507 (*1*x) – 1.018 (*x*x) –0.0173 (age) + 0.577 (inducers) + 0.00884 (lean body weight) | 37 | 10 | - | 27 | - | - | High | - | - |
| 6 | Sconce 2005^33^ | Ini-pgx | UK | AF, DVT, PE, other | 2.0–3.0 | 297 | 100 | - | - | - | 54 | 68 (range 23–90) | Age, height | CYP2C9*2, *3; VKORC1-1639G>A | √dose (mg/d) = 0.628 – 0.0135 (age) – 0.240 (CYP2C9*2) – 0.370 (CYP2C9*3) – 0.241 (VKORC1 -1639 G>A) + 0.0162 (height). | 54 | - | 15 | 18 | - | - | High | 32 | 1 |
| 7 | Wadelius 2005^34^ | Ini-pgx | Sweden | AF, HV, DVT, PE, CMP, TIA | - | 201 | 99 | 1 | - | - | 67 | mean 67 (range 28–88) | Age, weight, HV, CYP2C9 interacting drugs | CYP2C9*2*3; VKORC1 1173C>T | Dose (mg/wk) = 15.5 – 23.2 (VKORC1 1173 TT) – 11.5 (VKORC1 1173 CT) + 34.4 (CYP2C9*1*1) + 29.7 (CYP2C9*1 *2) + 23.9 (CYP2C9*1*3) + 13.7 (CYP2C9*2*2) + 14.3 (CYP2C9*2*3) – 0.316 (age) + 20.6 (CYP2C9 inducers) + 2.16 (no CYP2C9 interactions) + 0.222 (weight) – (4.65 × if no HV) (from Lubitz 2010^35^). | 56 | - | 29 | 11 | - | - | High | 2 | - |
| 8 | Aquilante 2006^36^ | Ini-pgx | USA | AF, DVT, PE, CVA/TIA, HV, CMP | 2.0–4.0 | 350 | 92 | <1 | 7 | - | 87 | 69 ± 11 | Age, weight, smoking, mean and goal INR, vit K intake, enzyme inhibitors and inducers | CYP2C9*2, *3, *5; VKORC1-1639G>A; Factor VII I/D; Factor X I/D | Dose = 19.9 − 23.3 (VKORC1 -1639 AA) − 13.2 (VKORC1 -1639 GA) + 0.21 (weight) − 8.9 (CYP2C9 heterozygotes) − 10.9 (CYP2C9 homozygotes) − 0.23 (age) − 8.0 (CYP2C9 inhibitors) + 6.6 (smoking status) + 9.9 (mean INR) + 10.9 (CYP2C9 inducers) − 6.2 (goal INR) + 4.2 (Factor X I/D) + 3.8 (Factor X I/I) + 3.1 (Factor VII D/D) + 0.03 (calculated vitamin K intake). | 51 | 31 | 52 | 14 | - | - | High | - | - |

**Table S5. Continued**

| **#** | **Algorithm** | **Sub-type** | **Country** | **Indication** | **Target INR** | **N** | **Ethnicity (%)** | | | | **Male %** | **Age, yrs (mean ± SD or median, IQR)** | **Clinical parameters** | **Genetic parameters** | **Equation^a^** | **R2^b^** | | | | **MAE (mg/d)** | **Other** | **ROB** | **# EV** | **# CUA** |
| --- | --- | --- | --- | --- | --- | --- | --- | --- | --- | --- | --- | --- | --- | --- | --- | --- | --- | --- | --- | --- | --- | --- | --- | --- |
|  |  |  |  |  |  |  | **W** | **A** | **B** | **M** |  |  |  |  |  | **All** | **Cl** | **VKORC1** | **CYP2C9** |  |  |  |  |  |
| 9 | Carlquist 2006^37^ | Ini-pgx | USA | AF, DVT | 2.0–3.0 | 213 | Y | - | - | - | 49 | 71 ± 12 | Age, gender, weight | CYP2C9*2, *3; VKORC1 1173C>T | Dose (mg/wk) = 1.64 + exp [3.984 –0.197 (CYP2C9*1*2) – 0.360 (CYP2C9 *1*3) – 0.947 (CYP2C9*2*3) – 0.265 (CYP2C9 *2*2) – 1.892 (CYP2C9*3*3) – 0.304 (VKORC1 CT) – 0.569 (VKORC1 TT) – 0.009 (age) + 0.094 (males) + 0.003 (weight)] (from Anderson 2007^38^) | 45 | 12 | 15 | 18 | - | - | High | 16 | 1 |
| 10 | Herman 2006^39^ | Ini-pgx | Slovenia | AF, DVT, HV, other | 2.0–3.5 | 165 | 100 | - | - | - | 50 (of 188) | 72 ± 9 (for 188) | Age, BSA | CYP2C9*2, *3; VKORC1 1173C>T, 3730G>A | Log dose (mg/d) = 0.690 – 0.315 (VKORC1 1173 TT) – 0.124 (VKORC1 1173 CT) – 0.194 (CYP2C9*1*x) – 0.383 (CYP2C9*x*x) – 0.005 (age) + 0.188 (BSA) + 0.107 (VKORC1 3730 AA) | 60 | 7 | 35 | 18 | - | - | High | 4 | - |
| 11 | Takahashi 2006^40^ | Ini-pgx | Japan | AF, DVT, HV | 1.5–3.0 | 179 | 64 | 36 | - | - | 57 (of 329) | 63 ± 12 (for 329) | Age, weight | CYP2C9*2, *3, *11; VKORC1 1173C>T | Dose (mg/d) = 6.656 – 0.035 (age) + 0.031 (weight) – 1.706 (CYP2C9*1*x) – 2.815 (CYP2C9*x*x) – 1.316 (VKORC1 CT) – 2.941 (VKORC1 TT) | 57 | - | - | - | - | - | High | 9 | - |
| 12 | Tham 2006^41^ | Ini-pgx | Singapore | AF, HV, TE, other | 2.0–3.0 | 107 | - | 100 | - | - | 59 | 57 ± 13 | Age, weight | CYP2C9*3, VKORC1 381 C>T (to infer the VKORC1 haplotype) | Dose (mg/d) = 10[exp (0.838 – 0.005 (age) + 0.003 (weight) – 0.189 (CYP2C9 *3) – 0.283 (VKORC1 CC) – 0.119 (VKORC1 TC )] | 60 | - | 35 | 13 | - | IVC (n=108): MPE = -0.23 mg/d, within 1 to 0.5 mg/d = 52% | High | 7 | 1 |
| 13 | Anderson 2007^38^ | Ini-pgx | USA | AF, DVT, PE, orthopaedic, other | 2.0–3.0 | 175 | 95 | - | - | - | 53 (of 200) | range 18–86 (for 200) | Age, gender, weight | CYP2C9*2, *3; VKORC1 1173C>T | No explicit equation | 47 | 15 | 32 | | - | - | High | 2 | - |
| 14 | Caldwell 2007^42^ | Ini-pgx | USA | AF, HV, TE, other | 2.0–3.5 | 431 | 100 | - | - | - | 56 | 72 (range 28–94) | Age, BSA, gender, HV, DM | CYP2C9*2, *3; VKORC1 1542G>C | No explicit equation (presents nomogram simplified to patient’s CYP2C9 and VKORC1 genotype, age, and gender) | 56 | - | - | - | - | - | High | - | - |
| 15 | Miao 2007^43^ | Ini-pgx | China | AF, DVT, PE, HV | 1.5–3.0 | 178 | - | 100 | - | - | 42 | 55 ± 13 | Age, Weight | CYP2C9*3, VKORC1 -1639G>A | Dose (mg/d) = 6.22 – 0.011 (Age) + 0.017 (Weight) – 0.775 (CYP2C9*3) – 3.397 (VKORC1 GA) – 4.803 (VKORC1 AA) | 63 | 15 | 49 | 2 | - | - | High | 15 | - |
| 16 | Rieder 2007^44^ | Ini-pgx | USA | - | - | 186 | 100 | - | - | - | - | - | Age, gender, losartan, amio | CYP2C9*2, *3; VKORC1 1173C>T; 5 GGCX SNPs | Ln dose (mg/d) = 2.43 – 0.008 (age) – 0.098 (gender) – 0.187 (amio) + 0.013 (losartan) – 0.306 (CYP2C9) – 0.327 (VKORC1) + 0.078 x 4046 (rs1254897) + 0.033 (rs699664) – 0.172 (rs11676382) + 0.013 (rs2028898) + 0.023 (rs10691423) – 0.063 (rs13406935) | 21 | 8 | - | - | - | - | High | - | - |
| 17 | Rieder 2007^44^ | Ini-pgx | USA | - | - | 186 | 100 | - | - | - | - | - | Age, gender, losartan, amio | CYP2C9*2, *3; VKORC1 1173C>T; GGCX haplotype alleles | Ln dose (mg/d) = 2.54 – 0.009 (age) – 0.049 (gender) – 0.247 (amio) – 0.047 (losartan) – 0.280 (CYP2C9) – 0.320 (VKORC1) – 0.160 (Hap2) + 0.047 (Hap4) – 0.058 (Hap10) – 0.158 (Hap11) + 0.066 (HapRare). | - | - | - | - | - | - | High | - | - |

**Table S5. Continued**

| **#** | **Algorithm** | **Sub-type** | **Country** | **Indication** | **Target INR** | **N** | **Ethnicity (%)** | | | | **Male %** | **Age, yrs (mean ± SD or median, IQR)** | **Clinical parameters** | **Genetic parameters** | **Equation^a^** | **R2^b^** | | | | **MAE (mg/d)** | **Other** | **ROB** | **# EV** | **# CUA** |
| --- | --- | --- | --- | --- | --- | --- | --- | --- | --- | --- | --- | --- | --- | --- | --- | --- | --- | --- | --- | --- | --- | --- | --- | --- |
|  |  |  |  |  |  |  | **W** | **A** | **B** | **M** |  |  |  |  |  | **All** | **Cl** | **VKORC1** | **CYP2C9** |  |  |  |  |  |
| 18 | Zhu 2007^45^ | Ini-pgx | USA | AF | 2.0–3.0 | 65 | 100 | - | - | - | 54 | mean 69 (range 41-92) | Age, gender, weight | CYP2C9*2, *3; VKORC1-1639 G>A | Ln dose (mg/d) = 1.35 – 0.008 (age) + 0.116 (male) + 0.004 (weight) – 0.376 (VKORC1 AA) + 0.271 (VKORC1 GG) – 0.307 (CYP2C9*2) – 0.318 (CYP2C9*3) | 61 | - | 27 | 22 |  | ‘SE’ of the dose estimate = 0.39 mg/d | High | 16 | - |
| 19 | Caldwell 2008^46^ | Ini-pgx | USA | AF, DVT, PE, HV, other | 2.0–3.5 | 1051 | 100 | - | - | - | Cohorts from Gage 2004, Hillman 2004, Aquilante 2006, and Caldwell 2007 | | Age, gender, BSA, HV | CYP2C9*2, *3; VKORC1 -1639 G>A; CYP4F2*3 | For CYP2C9*1*1, *1*2 and *1*3, Log dose (mg/wk) = 4.004 –0.186 (CYP2C9) – 0.340 (VKORC1) + 0.069 (VKORC1 Het) + 0.111 (CYP4F2) + 0.061 (male) + 0.165 (HV) – 0.009 (age) + 0.320 (BSA) Geometric mean doses for CYP2C9 *2*2, *2*3 and *3*3 are 24.9, 14.8 and 8.1 mg/wk (From Burmester 2011^47^) | 56 | 17 | - | - | - | - | High | 1 | 1 |
| 20 | Gage 2008^48^ | Ini-pgx | USA | AF, DVT, PE, HV | 1.5–3.2 for most | 1015 | 83 | - | 15 | 2 | 64 | 65 ± 14 | Age, BSA, amio, target INR, smoking, race, DVT, PE | CYP2C9*2, *3; VKORC1 -1639 G>A | Ln dose (mg/d) = 0.9751 – 0.3238 (VKORC1 GA) + 0.4317 (BSA) – 0.00745 (age) – 0.4008 (CYP2C9*3) – 0.2066 (CYP2C9*2) + 0.2029 (target INR) – 0.2538 (amio) + 0.0922 (smokes) – 0.0901 (Blacks) + 0.0664 (DVT/PE) | 53 (White 57, Black 31) | 22 | 25 | 11 | - | - | High | 46 | 8 |
| 21 | Haug 2008^49^ | Ini-pgx | Norway | AMI | 2.8–4.2 | 105 | 100 | - | - | - |  | - | Age, aspirin | CYP2C9*2, *3; VKORC1 -1639G>A | Dose (mg/wk) = 72.297 – 18.946 (VKORC1 AA) – 11.930 (VKORC1 GA) – 8.540 (aspirin) –7.275 (CYP2C9*1*x) – 15.459 (CYP2C9*x*x) – 0.321 (age) | 47 | 4 | 25 | 7 | - | - | High | 1 | - |
| 22 | Kimmel 2008^50^ | Ini-pgx | USA | AF, DVT, PE, CMP, stroke/ TIA, other | 2.0–3.0 | 232 | 52 | - | 48 | - | 68 | 59 ± 15 | Age, BMI, warfarin potentiators and employment status | CYP2C9*2, *3, VKORC1 1173C>T and APOE ε2, ε3, ε4 | √dose (mg/wk) = 8.912 – 1.097 (VKORC1) – 0.497 (CYP2C9) – 0.023 (age) – 0.180 (# warfarin potentiators) – 0.820 (disabled) – 0.301 (retired) –0.857 (unemployed) – 0.687 (BMI <25) – 0.689 (BMI 25–30) + 0.517 (any ε4 × race) + 0.152 (any ε4, Whites) + 0.670 (any ε4, Blacks) | White 57, Black 55 | - | - | - | - | - | High | - | - |
| 23 |  | Ini-pgx | USA | AF, DVT, PE, dilated CMP, stroke/ TIA, other | 2.0–3.0 | 232 | 52 | - | 48 | - | 68 | 59 ± 15 | Age, BMI, warfarin potentiators and employment status | CYP2C9*2, *3, VKORC1 1173C>T and APOE ε2, ε3, ε4 | √dose (mg/wk) = 8.935 – 1.112 (VKORC1) – 0.487 (CYP2C9) – 0.021 (age) – 0.180 (# warfarin potentiators) – 0.872 (disabled) – 0.407 (retired) – 0.970 (unemployed) – 0.685 (BMI <25) – 0.652 (BMI 25–30) + 0.567 (APOE three-level × race) – 0.051 (APOE three-level, Whites) + 0.516 (APOE three-level, Blacks) | White 57, Black 54 | - | - | - | - | - | High | - | - |
| 24 | Meckley 2008^51^ | Ini-pgx | USA | AF, dilated CMP, DVT, PE, HV | 1.8–3.7 | 122 | 100 | - | - | - | 65 (of 172) | 60 ± 16 (for 172) | Age, weight, amio, CHF, malignancy | CYP2C9 *2, *3, VKORC1 haplotypes A and B (defined by -1639 G>A et al.) | Dose (mg/d) = 5.67 – 1.04 (CYP2C9*2) – 1.14 (CYP2C9*3) + 1.85 (VKORC1 BB) – 2.33 (VKORC1 AA) – 0.05 (age) + 0.02 (weight, kg) – 1.14 (amio) – 0.90 (CHF) + 1.07 (malignancy). | 50 | 16 | 27 | 12 | - | - | High | - | - |

**Table S5. Continued**

| **#** | **Algorithm** | **Sub-type** | **Country** | **Indication** | **Target INR** | **N** | **Ethnicity (%)^a^** | | | | **Male %** | **Age, yrs (mean ± SD or median, IQR)** | **Clinical parameters** | **Genetic parameters** | **Equation^b^** | **R2^c^** | | | | **MAE (mg/d)** | **Other** | **ROB** | **# EV** | **# CUA** |
| --- | --- | --- | --- | --- | --- | --- | --- | --- | --- | --- | --- | --- | --- | --- | --- | --- | --- | --- | --- | --- | --- | --- | --- | --- |
|  |  |  |  |  |  |  | **W** | **A** | **B** | **M** |  |  |  |  |  | **All** | **Cl** | **VKORC1** | **CYP2C9** |  |  |  |  |  |
| 25 | Oner Ozgon 2008^52^ | Ini-pgx | Turkey | HV, AF, DVT, PE, CVA, TIA, other | 1.5–3.0 | 205 | - | - | - | 100 | 46 | 58 ± 16 | Age, indication | CYP2C9*2, *3, *4; VKORC1 -1639G>A | Ln dose (mg/wk) = 4.1 − 0.2 (VKORC1) − 0.2 (CYP2C9) − 0.007 (age) + 0.2 (non-indication of DVT/PE) | 34 | 12 | 17 | 13 |  |  | High | 1 | - |
| 26 | Perini 2008^53^ | Ini-pgx | Brazil | AF, HV, TE, other | 2.0–3.5 | 390 | 50 | - | 20 | 30 | 48 | 54 (range 18–91) | Age, weight, HV, TE, amio, sim | CYP2C9*2, *3, *5, *11; VKORC1-1639A | √dose (mg/wk) = 3.8548 − 0.0103 (age) + 0.0159 (weight) + 0.4284 (HV) + 0.3983 (TE) − 0.4387 (sim) − 0.7903 (amio) − 0.6179 (*1*x) − 1.0726 (*x*x) − 0.8516 (VKORC1 GA) − 1.7856 (VKORC1 AA) | 51 (White 51, Black 52) | - | 24 | 7 | 0.99 | M%PE (bias) = 6%, MAPE (precision) = 24% | High | 7 | - |
| 27 | Sasaki 2009^54^ (bases on PKPD model) | Ini-pgx | Japan | HV, IHF | 1.5–3.8 | 45 | - | 100 | - | - | 60 | 63 ± 10 | Apparent S-warfarin clearance, half maximal effecti-ve concentrati-on, initial INR | CYP2C9*3; VKORC1 1173 C>T (used to determine PK PD parameters) | Dose (mg/d) = 11.2 (apparent S-warfarin clearance) + 0.91 (half maximal effective concentration) + 2.36 (INR) – 9.67 | 94 | - | - | - | - | MPE= 0.01 mg/d, RMSE = 0.44 mg/d | High | - | - |
| 28 | Schelleman 2008^55^ | Ini-pgx | USA | AF, DVT, PE, stroke/TIA, CMP, other | 2.0–3.0 | 259 | 57 | - | 43 | - | 66 | 60 ± 15 | Age, BMI, gender, race, warfarin potentiators, alcohol, DVT history | CYP2C9*2, *3; VKORC1 1173C>T; Factor 7 −401G>T (rs510355) | Ln dose (mg/d) = 2.81 − 0.08 (Black) − 0.01 (age) − 0.39 (VKORC1 CT) − 0.82 (VKORC1 TT) − 0.11 (Factor 7 GT) − 0.19 (Factor 7 TT) − 0.16 (CYP2C9 any*2) − 0.30 (CYP2C9 any*3) − 0.18 (BMI <25) − 0.23 (BMI 25 to 30) – 0.08 (# warfarin potentiators) − 0.10 (female) + 0.06 (most alcoholic drinks on one occasion) − 0.03 (Black x most alcoholic drinks) + 0.58 (DVT) − 0.33 (Black x DVT) | 37 | - | - | - | - | LOOCV R2 = 31%, within 1 mg/d = 42% | High | - | - |
| 29 |  | Ini-pgx | USA | AF, DVT, PE, stroke/TIA, CMP, other | 2.0–3.0 | 147 | 100 | - | - | - | 71 | 63 ± 14 | Age, BMI, gender, warfarin potentiators, alcohol, DVT history | CYP2C9*2, *3; VKORC1 1173C>T | Ln dose (mg/d) = 2.74 − 0.01 (age) − 0.40 (VKORC1 CT) − 0.82 (VKORC1 TT) − 0.21 (CYP2C9 any*2) − 0.32 (CYP2C9 any*3) − 0.18 (BMI <25) − 0.19 (BMI 25 to 30) − 0.08 (#potentiators) + 0.03 (most alcoholic drinks on one occasion) + 0.21 (DVT history) − 0.13 (female) | 43 | - | - | - | - | LOOCV R2 = 37%, within 1 mg/d = 47% | High | 1 | - |
| 30 |  | Ini-pgx | USA | AF, DVT, PE, stroke/TIA, CMP, other | 2.0–3.0 | 112 | - | - | 100 | - | 59 | 57 ± 15 | Age, BMI, warfarin potentiators | VKORC1 -1173CT, Factor VII -401 GT, APOE ε2, ε3, ε4 | Ln dose (mg/d) = 2.66 − 0.01 (age) − 0.36 (VKORC1 CT or TT) − 0.19 (Factor 7 GT) − 0.25 (Factor 7 TT) − 0.28 (APOE ε2ε2 or ε2ε3) − 0.21 (APOE ε3ε3 or ε2ε4) − 0.19 (BMI <25) − 0.25 (BMI 25 to 30) − 0.09 (# warfarin potentiators) | 28 | - | - | - | - | LOOCV R2 = 23%, within 1 mg/d = 34% | High | - | - |
| 31 | Wen 2008^56^ | Ini-pgx | Taiwan | AF, DVT, HV, stroke | 1.7–3.0 | 108 | - | 100 | - | - | 58 | 64 ± 14 | Age, BSA | CYP2C9*3; VKORC1 -1639G>A | Dose (mg/d) = −0.432 + 0.769 (genotype-based initial dose) – 0.015 (age) + 1.125 (BSA). Genotype-based initial dose (mg/d): GG*1*1 = 5; GG*1*3, GG*3*3, AG*1*1 = 3.75; AG*1*3, AG*3*3, AA*1*1 = 2.5; AA*1*3, AA*3*3 = 1.25) | 48 | 15 | 29 | 5 | - | - | High | 7 | 1 |

**Table S5. Continued**

| **#** | **Algorithm** | **Sub-type** | **Country** | **Indication** | **Target INR** | **N** | **Ethnicity (%)^a^** | | | | **Male %** | **Age, yrs (mean ± SD or median, IQR)** | **Clinical parameters** | **Genetic parameters** | **Equation^b^** | **R2^c^** | | | | **MAE (mg/d)** | **Other** | **ROB** | **# EV** | **# CUA** |
| --- | --- | --- | --- | --- | --- | --- | --- | --- | --- | --- | --- | --- | --- | --- | --- | --- | --- | --- | --- | --- | --- | --- | --- | --- |
|  |  |  |  |  |  |  | **W** | **A** | **B** | **M** |  |  |  |  |  | **All** | **Cl** | **VKORC1** | **CYP2C9** |  |  |  |  |  |
| 32 | Wen 2008^56^ | Ini-pgx | Taiwan | AF, DVT, HV, stroke | 1.7–3.0 | 75 | - | 100 | - | - | Subset of 108 above (from one hospital only) | | Age, BSA, HTN | CYP2C9*3; VKORC1 -1639G>A | Dose (mg/d) = −0.443 + 0.798 (genotype-based initial dose) – 0.018 (age) + 1.4 (BSA) – 0.269 (HTN) | 62 | 24 | 36 | 1 | - | - | High | 1 | - |
| 33 | Wu 2008^57^ | Ini-pgx | USA | AF, DVT, PE, HV, stroke, other | 2.0–3.0 | 92 | 48 | 16 | 34 | 2 | 70 | 57 ± 13 | Age, race, CYP2C9 inhibitor, gender, weight, height, smoking, amio, sulfamethoxazole | CYP2C9*2, *3; VKORC1-1639G>A, 2255C>T | Ln dose (mg/d) = 1.10837 – 0.03597 (CYP2C9*2) – 0.21622 (CYP2C9*3) – 0.00218 (age) – 0.08091 (Asian) – 0.00365 (Black) – 0.03838 (Hispanic) – 0.11285 (inhibitors) + 0.06889 (other ethnicity) + 0.08583 (gender) + 0.00162 (weight) – 0.00257 (height) + 0.03087 (smoking) + 0.02287 (VKORC1 -1639) – 0.07326 (VKORC1 2255CT) – 0.32939 (VKORC1 2255TT) | 59 | - | 41 | 5 | - | - | High | 7 | - |
| 34 | Huang 2009^58^ | Ini-pgx | China | AF, DVT, HV | 1.8–3.0 | 266 | - | 100 | - | - | 46 | 52 ± 15 | Age, BSA | CYP2C9*3; VKORC1 1173C>T | Ln dose (mg/d) = 0.727 – 0.007 (age) + 0.384 (BSA) + 0.403 (VKORC1 TC) + 0.554 (VKORC1 CC) – 0.482 (CYP2C9*1*3) –1.583 (CYP2C9*3*3) | 54 | 18 | 32 | 33 | - | - | High | 19 | 2 |
| 35 | IWPC 2009^3^ | Ini-pgx | Nine | AF, DVT, PE, HV, stroke, other | 2.0–3.0 | 4043 | 55 | 30 | 9 | 6 | - | In categories | Age, height, weight, race, enzyme inducers (carbamazepine, phenytoin, rifampicin, or rifampicin), amio | CYP2C9*2, *3; VKORC1-1639A | √dose (mg/wk) = 5.6044 – 0.2614 (age) + 0.0087 (height) + 0.0128 (weight) – 0.8677 (VKORC1 AG) – 1.6974 (VKORC1 AA) – 0.4854 (VKORC1 unknown) – 0.5211 (CYP2C9*1*2) – 0.9357 (CYP2C9 *1*3) – 1.0616 (CYP2C9*2*2) – 1.9206 (CYP2C9*2*3) – 2.3312 (CYP2C9*3*3) – 0.2188 (CYP2C9 unknown) – 0.1092 (Asian) – 0.2760 (Black) – 0.1032 (Mixed/Missing race) + 1.1816 (enzyme inducer) – 0.5503 (amio) | 47 | 27 | 13 | 7 | 1.19 | IVC (n = 1009), R2 = 43%, MAE = 1.21, 45% patients with ideal dose; plus one outlier  MAE = 1.26, R2 = 33% | High | 72 | 7 |
| 36 | Kim 2009^59^ | Ini-pgx | Korea | HV | 1.7–2.8 | 239 | - | 100 | - | - | 43 (of 265) | 54 ± 11 (for 265) | Age, weight, CHF/CMP, INR-increasing drug, aspirin, INR decreasing dietary supplements | CYP2C9*3, *13, *14; VKORC1 1173C>T | √dose (mg/d) = 1.85694 – 0.39677 (CYP2C9) + 0.41738 (VKORC1) – 0.00487 (age) + 0.00683 (weightg) – 0.14930 (CHF/CMP) –0.24163 (INR-increasing drug) – 0.17099 (aspirin) + 0.07370 (INR-decreasing dietary supplements) | 56 | - | 20 | 23 |  | IVC (n=26), r = 0.79 | High | 5 | - |
| 37 | Ohno 2009^60^ | Ini-pgx | Japan | AF, DVT, PE, others | 1.5–3.0 | 125 | - | 100 | - | - | 60 | 73 ± 12 | Age, BSA | CYP2C9*3; VKORC1 -1639G>A | Dose (mg/d) = 2.263 + 4.248 (VKORC1 GG) + 1.067 (VKORC1 GA) – 2.416 (CYP2C9*3*3) – 0.864 (CYP2C9*1*3) + 1.308 (BSA) – 0.025 (age) | 55 | - | 30 | 5 | 0.63 (from Sasano 2019^61^) | 49% with ideal dose, MPE = -0.01 mg/d, RMSE = 0.84mg/d, RMSPE = 42% (Sasano, 2019) | High | 12 | - |

**Table S5. Continued**

| **#** | **Algorithm** | **Sub-type** | **Country** | **Indication** | **Target INR** | **N** | **Ethnicity (%)^a^** | | | | **Male %** | **Age, yrs (mean ± SD or median, IQR)** | **Clinical parameters** | **Genetic parameters** | **Equation^b^** | **R2^c^** | | | | **MAE (mg/d)** | **Other** | **ROB** | **# EV** | **# CUA** |
| --- | --- | --- | --- | --- | --- | --- | --- | --- | --- | --- | --- | --- | --- | --- | --- | --- | --- | --- | --- | --- | --- | --- | --- | --- |
|  |  |  |  |  |  |  | **W** | **A** | **B** | **M** |  |  |  |  |  | **All** | **Cl** | **VKORC1** | **CYP2C9** |  |  |  |  |  |
| 38 | Sandanaraj 2009^62^ | Ini-pgx | Singapore | DVT, AF, HV, TIA, stroke | 2.0–3.0 | 107 | - | 100 | - | - | 61 | 64 (range 31–87) | Age, weight | CYP2C9*2, *3; VKORC1 diplo-type (H) status (from 8 SNPs) | Dose (mg/wk = 33.15 + 15.27 (H1-H7) + 13.59 (H1-H*b) + 11.85 (H7-H*a) + 8.45 (H*a-H*b)－2.6 (H1-H*a)－6.93 (CYP2C9*2/*3]－0.32 (age) + 0.11 (weight) | 74 | 55 | 59 | 7 | - | - | High | - | - |
| 39 | Suarez-Kurtz 2009^63^ | Ini-pgx | Brazil | AF, TE, HV, other | 2.0–3.5 | 260 | 50 | - | 20 | 30 | 48 (of 390) | 54 (range 18–91) (for 390) | Age, INR per dose, weight, HV, TE, amio, sim | CYP2C9*2, *3, *5, *11; VKORC1 -1639G>A | √dose (mg/wk) = 5.5691 – 0.0054 (age) – 2.9909 (INR/dose) + 0.0157 (weight) + 0.5726 (HV) + 0.4333 (TE) – 0.4442 (sim) – 0.7748 (amio) – 0.5115 (CYP2C9*1*x) – 1.1043 (CYP2C9*x*x) – 0.8352 (VKORC1 GA) ¬ 1.6841 (VKORC1 AA) | 60 | 15 | 20 | 6 | 0.93 | IVC (n=130), R2 = 59%, MAE = 0.89 mg/d | High | - | - |
| 40 | Wadelius 2009^64^ | Ini-pgx | Sweden | AF, DVT, PE, HV, other | 2.0–3.0 | 1324 | Y | - | - | - | 63 (of 1496) | 66 (57–74) (for 1496) | Age, gender, INR-increasing drugs | CYP2C9*2, *3; VKORC1 -1639G>A | √Dose (mg/wk) = 9.46832 – 0.90112 (VKORC1 GA) – 2.01863 (VKORC1 AA) – 0.50836 (CYP2C9*1*2) – 0.97546 (CYP2C9*1*3) – 1.10204 (CYP2C9*2*2) – 1.74761 (CYP2C9*2*3) – 3.40061 (CYP2C9*3*3) – 0.03686 (age) – 0.27698 (female) – 0.06992 (# INR-increasing drugs) | 59 | - | 30 | 12 | - | - | High | 20 | - |
| 41 | Yoshizawa 2009^65^ | Ini-pgx | Japan | AF, DVT, PE, HV, other | 1.5–3.0 | 93 | - | 100 | - | - | 67 (of 259) | 70 (range 22-89) [for 259] | Age, gender, height, weight, serum albumin | CYP2C9*3; VKORC1 -1639G>A | Dose (mg/d) = -52.85597 + 0.23961 (height) + 0.95716 (weight) + 0.32730 (age) – 21.76605 (male) + 1.33624 (albumin) + 1.35883 (⁎1⁎1 GA) + 2.11405 (⁎1⁎1 GG) – 0.97512 (⁎1⁎3 AA) + 0.47364 (⁎1⁎3 GA) – 0.00409 (weight x height) – 0.06054 (weight x albumin) – 0.00116 (weight x age) + 0.04188 (weight x male) + 0.01795 (height x albumin) – 0.00170 (height x age) + 0.09418 (height x male) – 0.00973 (albumin x age) + 0.57980 (albumin x male) + 0.03176 (age x male) | 33 | - | - | - | - | - | High | 1 | - |
| 42 | Cen 2010^66^ | Ini-pgx | China | HV | 1.5–3.0 | 222 | - | 100 | - | - | 47 | 45 ± 12 | Age, weight, amio | CYP2C9*3, VKORC1 -1639G>A, CYP4F2*3 | Ln dose (mg/d) = 0.57094 – 0.21148 (CYP2C9) – 0.18611 (VKORC1) + 0.03992 (CYP4F2) – 0.00115 (age) + 0.00152 (weight) – 0.23347 (amio) (from Xu 2018^67^) | 56 | - | 40 | 26 | - | - | High | 1 | 1 |
| 43 | Cha 2010^24^ | Ini-pgx | Japan | AF, heart failure, MI, stroke, DVT, PE, HV) | 1.5–3.0 | 440 | - | 100 | - | - | 66 | 68 ± 11 | Age, BSA, amio | CYP2C9 rs10509680; VKORC1 -1639 G>A; CYP4F2*3 | Dose (mg/d) = 1.823954 – 0.023583 (age) + 1.118196 (BSA) + 0.697966 (CYP2C9) + 1.386091 (VKORC1) + 0.22656 (CYP4F2) – 0.755881 (amio) | 43 | 15 | 26 | 2 | - | - | High | 1 | - |
| 44 | Du 2010 (from Zhao 2014^68^) | Ini-pgx | China | - | 1.5–3.0 | - | - | Y | - | - | - | - | Age, weight | CYP2C9, VKORC1 | No explicit equation | 55 |  |  |  |  |  | Unclear | 1 | - |

**Table S5. Continued**

| **#** | **Algorithm** | **Sub-type** | **Country** | **Indication** | **Target INR** | **N** | **Ethnicity (%)^a^** | | | | **Male %** | **Age, yrs (mean ± SD or median, IQR)** | **Clinical parameters** | **Genetic parameters** | **Equation^b^** | **R2^c^** | | | | **MAE (mg/d)** | **Other** | **ROB** | **# EV** | **# CUA** |
| --- | --- | --- | --- | --- | --- | --- | --- | --- | --- | --- | --- | --- | --- | --- | --- | --- | --- | --- | --- | --- | --- | --- | --- | --- |
|  |  |  |  |  |  |  | **W** | **A** | **B** | **M** |  |  |  |  |  | **All** | **Cl** | **VKORC1** | **CYP2C9** |  |  |  |  |  |
| 45 | Harada 2010^69^ | Ini-pgx | Japan | HV | 2.0–3.0 | 97 | - | 100 | - | - | 53 | 63 ± 9 | Age, BSA, WBC count, allopurinol | CYP2C9*3, VKORC1-1639G>A, CYP4F2*3 | Dose (mg/d) = 6.800 + 2.013 (BSA) – 0.04306 (age) – 2.870 (VKORC1 AA) – 2.233 (VKORC1 GA) – 0.912*CYP2C9 (*1*3) – 0.171 (WBC) – 0.596 (allopurinol) + 0.674 (CYP4F2*3*3) (only final algorithm shown) | 49 | - | - | - | - | - | High | - | - |
| 46 | King 2010^70^ (updates Gage 2008) | Ini-pgx | USA | AF, DVT, PE | Multiple | 985 | 78 | <1 | 18 | 3 | 51 | 60 ± 15 | Age, BSA, amio, target INR, smoking, race, DVT, PE | CYP2C9*2, *3; VKORC1-1639A; GGCX rs11676382 | Whites: multiply Gage 2008 predicted dose by 1.01 (CC), 0.95 (GC) or 0.89 (CC). Asians/Blacks: multiply Gage 2008 dose by 1.00 (CC), 0.94 (GC) or 0.88 (GG). | 53 | - | - | - | - | - | High | See Gage 2008 | |
| 47 | McMillin 2010^71^ | Ini-pgx | USA | Elective total hip or knee arthroplasty | 1.8–2.9 | 115 | 90 | - | - | - | 39 (of 229) | 59 ± 13 (for 229) | Age, gender, weight, hip surgery | CYP2C9*2, *3; VKORC1 -1639G>A | Dose (mg/d) = 8.755 – 0.055 (age) + 0.0058 (weight) – 1.163 (# variants) – 0.275 (hip surgery) – 0.257 (female) | - | - | - | - | - | - | High | 1 | - |
| 48 | Namazi 2010^72^ | Ini-pgx | Iran | AF, DVT, PE, HV, CAD or IHD | 2.0–3.0 | 55 | - | 100 | - | - | 44 | 53 ± 11 | Age, gender, height | CYP2C9*2, *3; VKORC1 -1639 G>A | Dose (mg/wk) = – 11.4 + 0.576 (height) – 7.21 (sex) – 0.244 (age) – 9.506 (CYP2C9) – 14.047 (VKROC1) | 41 | - | 20 | 17 | - | - | High | - | - |
| 49 | Nowak-Gottl 2010^73^  (***children***) | Ini-pgx | Germany | DVT, PE, stroke, other | 1.4–3.2 (for 59) | 34 | 100 | - | - | - | 46 (for 59) | 15 years (range 1–19 years) [for 59] | Age | CYP2C9*2, *3; VKORC1 -1639 G>A | √dose (mg/d) = 0.49 – 0.013 (age) − 0.08 (VKORC1 AA) + 0.01 (VKORC1 AG) − 0.02 (CYP2C9) (full model including phenprocoumon patients) | 34 (warfarin only) | 31 | 3 | <1 | - | - | High | 3 | - |
| 50 | Ozer 2010^74^ | Ini-pgx | Turkey | AF, DVT, HV | 2.0–3.5 | 100 | - | - | - | 100 | 39 | 49 ± 11 | Age, BSA | CYP2C9*2, *3; VKORC1 -1639G>A | Dose (mg/d) = − 14.33 − 0.009 (age) + 6.228 (BSA) + 1.661 (VKORC1) + 1.079 (CYP2C9*2) + 1.555 (CYP2C9*3) | 60 | 32 | 34 | 8 | - | - | High | - | - |
| 51 | Pautas 2010^75^ | Ini-pgx | France | DVT, PE, ATE, AF, other | 2.0–3.0 | 283 | 100 | - | - | - | 23 (of 300) | 87 ± 6 (for 300) | Age | CYP2C9*2, *3; VKORC1 -1639G>A; CYP4F2*3; EPHX1 rs2292566 | Dose (mg/d) = 7.26279 − 0.03316 (age) − 0.46609 (# CYP2C9 variant alleles) − 0.91457 (# VKORC1 variant alleles) − 0.38623 (# EPHX1 variant alleles) − 0.24814 (# CYP4F2 variant alleles) | 27 | 2 | 19 | 3 | - | - | High | - | - |
| 52 | Perini 2010^76^ | Ini-pgx | Brazil | AF, TE, HV, other | 2.0–3.5 | 370 | 50 | - | 20 | 30 | See Perini 2008 | | Age, weight, HV, TE, amio, sim | CYP2C9*2, *3, *5, *11; VKORC1 -1639G>A; CYP4F2*3 | √dose (mg/wk) = 5.406367 − 0.008738 (age)) + 0.016586 (weight) + 0.554908 (HV) + 0.241684 (TE) − 0.461530 (sim) − 0.779712 (amio) − 0.621912 (CYP2C9 *1*x) − 1.323429 (CYP2C9*x*x) − 0.998489 (VKORC1 GA) − 1.883283 (VKORC1 AA) + 0.060891 (CYP4F2*1*3) + 0.414532 (CYP4F2*3*3) | 56 | 17 | 25 | 9 | 0.94 | M%PE (bias) = 4% | High | - | - |
| 53 | Roper 2010^77^ | Ini-pgx | USA | AF, PE, DVT, CVA, HV, other | 2.0–3.0 | 121 | 100 | - | - | - | 61 (of 125) | 70 ± 13 (for 125) | Age, height | CYP2C9*2, *3; VKORC1 -1639G>A | Dose (mg/wk) = 46.20 − 8.93 (# variants) − 0.461 (age) + 0.173(height) | 64 | - | - | - | 1.00 | 56% with ideal dose, MAPE = 25% | High | 1 | - |
| 54 | Sagreiya 2010^78^ (updates IWPC 2009) | Ini-pgx | USA | - | 2.0–3.0 | 95 | 75 | 17 | 8 | - | 58 (of 104) | 67 (53–75) (for 104) | Age, height, weight, race, enzyme inducers, amio | CYP2C9*2, *3; VKORC1-1639A, CYP4F2*3 | No explicit equation | 51 | - | - | - | - | - | High | - | - |

**Table S5. Continued**

| **#** | **Algorithm** | **Sub-type** | **Country** | **Indication** | **Target INR** | **N** | **Ethnicity (%)^a^** | | | | **Male %** | **Age, yrs (mean ± SD or median, IQR)** | **Clinical parameters** | **Genetic parameters** | **Equation^b^** | **R2^c^** | | | | **MAE (mg/d)** | **Other** | **ROB** | **# EV** | **# CUA** |
| --- | --- | --- | --- | --- | --- | --- | --- | --- | --- | --- | --- | --- | --- | --- | --- | --- | --- | --- | --- | --- | --- | --- | --- | --- |
|  |  |  |  |  |  |  | **W** | **A** | **B** | **M** |  |  |  |  |  | **All** | **Cl** | **VKORC1** | **CYP2C9** |  |  |  |  |  |
| 55 | Sangviroon 2010^79^ | Ini-pgx | Thailand | HV, other | 1.5–3.5 | 89 | - | 100 | - | - | 46 | 49 ± 13 | Age | CYP2C9*3; VKORC1 haplotype AB | Ln dose (mg/wk) = 1.846＋0.412 (VKORC1 AB) + 0.559 (VKORC1 BB) + 1.512 (CYP2C9*1*1) + 1.136 (CYP2C9 *1*3) − 0.007 (age) | 54 | - | - | - | - | 36% with doses within 15% of actual | High | 1 | - |
| 56 | Takeuchi 2010^23^ (updates IWPC 2009) | Ini-pgx | Japan | AF, HV, DVT, PE, other | 1.6–2.6 | 200 | - | 100 | - | - | 68 | 67 ± 10 | Age, height, weight, amio, alcohol, smoking | CYP2C9*2; VKORC1 -1639G>A | √dose (mg/wk) = 3.798 – 0.261 (age in decades) + 0.009 (height) + 0.013 (weight) – 0.830 (VKORC1 AG) – 1.697 (VKORC1 AA) – 0.936 (CYP2C9*1*3) – 0.550 (amio) + 0.2331 (current drinker) – 0.2217 (non-drinker) + 0.3240 (current smoker) – 0.0465 (non-smoker) | 33 | - | - | - | - | - | High | - | - |
| 57 | Wells 2010^80^ | Ini-pgx | Canada | DVT, PE, other | 1.8–3.2 | 246 | 94 | - | - | - | 55 | 61 ± 14 | Age, BMI, height, exercise, angiotensin II receptor antagonists, β-blockers | CYP2C9*2, *3; VKORC1-1639A; CYP4F2*3 | Dose (mg/d) =10.42 – 0.048 (age) + 0.041 (BMI) + 0.05 (height) – 0.73 (less exercise) – 1.12 (CYP2C9*1*2) – 2.09 (CYP2C9*2*2) – 1.51 (CYP2C9*1*3) –1.44 (VKORC1 GA) – 2.86 (VKORC1 AA) – 1.33 (CYP4F2*1*1) – 1.24 (CPY4F2*1*3) – 1.46 (angiotensin II receptor antagonist) – 0.84 (β-blockers) | 58 | - | - | - | - | - | High | - | - |
| 58 | Zhang 2010 (from Zhao 2014^68^ and abstract) | Ini-pgx | China | AF, DVT, PE, HV, other | 2.0–3.0 | 101 | - | 100 | - | - | 53 | 51 ± 14 | Age, weight | CYP2C9*3 + 1075C>A; VKORC1-1639G>A | Ln dose (mg/d) = 0.346 + 0.017 (weight) – 0.376 (CYP2C9) + 0.148 (VKORC1) – 0.002 (age) | 67 | - | - | - | - | - | Unclear | 1 | - |
| 59 | Avery 2011^81^ (modified IWPC – included pharmacokinetic aspects) | Ini-pgx | UK | AF, DVT, HV, other | 2.0–3.0 | 671 | 100 | - | - | - | 55 | 68 ± 13 | Age, height, weight, amio | CYP2C9*2, *3; VKORC1 -1639G>A | 3-day dose = MD+x (day 1), MD+2x/3 (day 2), MD+x/3 (day 3) where MD = dose estimated by modified IWPC 2009^3^ equation (no ethnicity and warfarin metabolism inducers), x is given by formula reported in Avery 2011^81^. | - | - | - | - | - | r = 0.75 (for 316) | High | 2 | 1 |
| 60 | Botton 2011^82^ | Ini-pgx | Brazil | AF, TE, HV, CVA, thrombophilia, other | 2.0–3.5 | 279 | 100 | - | - | - | 56 | 63 ± 14 | Age, weight, amio, carbamazepine, β-blockers, amlodipine, diuretics | CYP2C9*2, *3; VKORC1 haplotype; CYP4F2*3; F2 -494T | Log10 dose (mg/wk) = 1.540 – 0.003 (age) + 0.004 (weight) – 0.077 (amio) + 0.198 x (carbamazepine) – 0.047 (β-blockers) - 0.065 (amlodipine) – 0.037 (diuretics) – 0.129 (CYP2C9*1*x) – 0.280 (CYP2C9*x*x) + 0.101 (two copies of haplotype GCG) + 0.049 (one haplotype GCA) + 0.087 (two haplotype GCA) – 0.105 (one haplotype ATG) – 0.187 (two haplotype ATG) + 0.041 (CYP4F2*1*3) + 0.102 (CYP4F2*3*3) – 0.120 (F2 494TT) | 63 | - | - | - | 0.99 | - | High | 1 | - |

**Table S5. Continued**

| **#** | **Algorithm** | **Sub-type** | **Country** | **Indication** | **Target INR** | **N** | **Ethnicity (%)^a^** | | | | **Male %** | **Age, yrs (mean ± SD or median, IQR)** | **Clinical parameters** | **Genetic parameters** | **Equation^b^** | **R2^c^** | | | | **MAE (mg/d)** | **Other** | **ROB** | **# EV** | **# CUA** |
| --- | --- | --- | --- | --- | --- | --- | --- | --- | --- | --- | --- | --- | --- | --- | --- | --- | --- | --- | --- | --- | --- | --- | --- | --- |
|  |  |  |  |  |  |  | **W** | **A** | **B** | **M** |  |  |  |  |  | **All** | **Cl** | **VKORC1** | **CYP2C9** |  |  |  |  |  |
| 61 | Botton 2011^82^ | Ini-pgx | Brazil | AF, TE, HV, CVA, thrombophilia, other | 2.0–3.5 | 279 | 100 | - | - | - | 56 | 63 ± 14 | Age, weight, amio, carbamazepine, β-blockers, amlodipine, diuretics | CYP2C9 *2, *3; VKORC1 -1639G>A | Log10 dose (mg/wk) = 1.608 –0.003 (age) + 0.004 (weight) – 0.067 (amio) + 0.201 (carbamazepine) – 0.037 (β-blockers) – 0.072 (amlodipine) – 0.040 (diuretics) – 0.123 (CYP2C9*1*x) – 0.290 (CYP2C9 *x*x) + 0.133 (VKORC1 GA) + 0.254 (VKORC1 AA) | 58 | - | - | - | 1.01 | - | High | - | - |
| 62 | Cavallari 2011^83^ | Ini-pgx | USA | DVT, PE, AF, stroke, HV, other | 2.0–3.5 | 50 | 100 | - | - | - | 36 | 57 ± 15 | Age, BSA, DVT, PE | CYP2C9*2, *3; VKORC1 -1639G>A | No explicit equation | 56 | 17 | 30 | 9 | - | - | High | - | - |
| 63 | Cho 2011^25^ | Ini-pgx | Korea | AF | 2.0–3.0 | 130 | - | 100 | - | - | 66 | 66 ± 13 | Age, BSA, statins | CYP2C9*3, VKORC1 1173C>T | Ln dose (mg/d) = 0.269 + 0.182 (VKORC1) – 0.112 (CYP2C9) – 0.005 (age) + 0.348 (BSA) – 0.082 (statins) | 60 | 40 | 18 | 2 | - | - | High | 6 | - |
| 64 | Choi 2011^84^ | Ini-pgx | Korea | AF, HV, DVT, PE, cerebral infarction | Average stable INR = 2.1 | 564 | - | 100 | - | - | 54 | 63 ± 12 | Age, BSA, gender, INR | CYP2C9*3; VKORC1 1173C>T; CYP4F2*3; GGCX 8016G>A | Dose (mg/d) = 1.73 – 0.03 (age) + 0.20 (gender) + 0.34 (INR) + 1.77 (BSA) + 0.76 (VKORC1 TC) + 1.41 (VKORC1 CC) – 1.18 (CYP2C9*3) – 0.19 (GGCX GA) – 0.36 (GGCX AA) + 0.17 (CYP4F2*1*3) + 0.58 (CYP4F2*3*3) | 35 | - | - | - | - | - | High | 3 | - |
| 65 | Cosgun 2011^85^ | Ini-pgx | USA | - | 2.0–3.0 | 290 | - | - | 100 | - | - | - | Age, weight, height, CHF, CKD, amio, ethnicity | CYP2C9*2*3; VKORC1 1173 C>T; ApoE rs429358, rs7412; CYP4 F2 rs2774030 + 200 SNPs | No explicit equation (random forest regression) | 66 | - | - | - | - | - | High | - | - |
| 66 |  | Ini-pgx | USA | - | 2.0–3.0 | 290 | - | - | 100 | - | - | - |  |  | No explicit equation (boosted regression tree) | 57 | - | - | - | - | - | High | - | - |
| 67 |  | Ini-pgx | USA |  | 2.0–3.0 | 290 | - | - | 100 | - | - | - |  |  | No explicit equation (support vector regression) | 58 | - | - | - | - | - | High | - | - |
| 68 | Gong 2011^86^ | Ini-pgx | Canada | AF, DVT, PE, other | 2.0–3.0 | 167 | 95 | 2 | 2 | 1 | 44 | 60 ± 17 | Age, weight, gender, amio | CYP2C9*2, *3; VKORC1-1639A; CYP4F2*3 | Dose (mg/d) = Genetics-based dose grid (in table) – 1.46 + 0.06 (weight) – 0.05 (age) – 0.90 (female) – 1.97 (amio) + 0.33 (# CYP4F2*3 alleles) | 42 | - | - | - | 1.49 | - | High | - | - |
| 69 | Moon 2011^87^ | Ini-pgx | Korea | TE (cerebral infarction, DVT) | 1.7–3.0 | 58 | - | 100 | - | - | 57 | 66 ± 15 | Age, years, HTN | CYP2C9*3; VKORC1 1173C>T | Log dose (mg/d) = 1.811 + 0.063 (VKORC1 CC/CT) – 0.012 (age) – 0.055 (CYP2C9*1*3) – 0.237 (HTN) | 61 | - | 31 | 4 | - | - | High | - | - |
| 70 | Moreau 2011^88^ | Rev-pgx | France | ATE, DVT, PE | 2.0–3.0 | 115 | 100 | - | - | - | 26 | 86 ± 6 | Age, indication, pretreatment INR (INR0) | CYP2C9*2, *3; VKORC1 -1639G>A | Dose (mg/d) = 11.3 − 0.0566 (age) − 0.607 (ATE) − 2.73 (INR0) − 0.529 (CYP2C9) − 0.890 (VKORC1) | 31 | - | - | - | - | 74% within 1 mg/d | High | - | - |
| 71 | Perera 2011^89^ | Ini-pgx | USA | AF, DVT, PE, HV | - | 330 | - | - | 100 | - | 28 | mean 57 | Age, weight, DVT/PE | CYP2C9*2, *3, *5, *8, *11, rs7089580; VKORC1 -1639G>A, -8191G>A; rs12777823 | Log2 dose (mg/wk) = 5.906398 – 0.014849 (age) + 0.004526 (weight) + 0.157924 (DVT/PE) – 0.254234 (CYP2C9* alleles) – 0.097194 (VKORC1 -8191G>A) + 0.060185 (rs7089580) – 0.37008 (VKORC1 -1639G>A) + 0.103657 (rs12777823) (from Hernandez 2014^90^) | 40 | 22 | 9 | 9 | - | - | High | 2 | - |

**Table S5. Continued**

| **#** | **Algorithm** | **Sub-type** | **Country** | **Indication** | **Target INR** | **N** | **Ethnicity (%)^a^** | | | | **Male %** | **Age, yrs (mean ± SD or median, IQR)** | **Clinical parameters** | **Genetic parameters** | **Equation^b^** | **R2^c^** | | | | **MAE (mg/d)** | **Other** | **ROB** | **# EV** | **# CUA** |
| --- | --- | --- | --- | --- | --- | --- | --- | --- | --- | --- | --- | --- | --- | --- | --- | --- | --- | --- | --- | --- | --- | --- | --- | --- |
|  |  |  |  |  |  |  | **W** | **A** | **B** | **M** |  |  |  |  |  | **All** | **Cl** | **VKORC1** | **CYP2C9** |  |  |  |  |  |
| 72 | Sarapakdi 2011 (from Chumnumwat 2018^91^) | Ini-pgx | Thailand | HV | 2.0–3.0 | 197 | - | 100 | - | - | - | - | Age, weight | CYP2C9, VKORC1 | No explicit equation | 61 | - | - | - | - | - | Unclear | 1 | - |
| 73 | Shahin 2011^92^ | Ini-pgx | Egypt | AF, DVT, PE, HV, CVA, CMP, other | 1.5–3.5 | 195 | - | - | - | 100 | 45 (of 207) | 47 ± 15 (for 207) | Age, PE, smoking | CYP2C9*2, *3, *4, *5, *8; VKORC1 -1639G>A, APOE ε2 | √dose (mg/wk) = 8.06 − 0.63 (VKORC1) − 0.30 (age in decades) − 0.41 (CYP2C9) + 1.44 (PE) − 0.60 (APOE) + 0.58 (smoking) | 31 | 13 | 10 | 5 |  |  | High | - | - |
| 74 | Shrif 2011^93^ | Ini-pgx | Sudan | AF, DVT, PE, HV, other | 2.0–3.5 | 157 | - | - | - | 100 | 44 (of 203) | 39 ± 14 (for 203) | Weight, HV/other, concurrent medications | CYP2C9 *2, *5, *6, *11; VKORC1 1542G>C, 3730G>A, rs7199949 | Log dose (mg/d) = 0.143 + 0.002 (weight) + 0.12 (VKORC1 1542G>C) − 0.13 (rs7199949) + 0.14 (VKORC1 3730G>A) + 0.13 (CYP2C9) + 0.07 (concurrent medication) + 0.08 (HV/other) | 34 |  | 27 | 5 |  | - | High | - | - |
| 75 | Singh 2011^94^ | Ini-pgx | Singapore | AF, DVT, HV, PE, other | 2.0–3.0 | 76 | - | 100 | - | - | 57 (of 124) | 61 (range 29–87) (for 124) | Age, weight | CYP2C9*2, *3; VKORC1 diplotype; CYP4F2*3 | Low-dose diplotype patients’ dose (mg/wk) = 27.74 − 5.39 (CYP2C9 (*2*3 + *3*3) − 0.25 (age) + 0.12 (weight) + 3.48 (CYP4F2 *1*3 + *3*3) | 38 | - | - | 9 |  | - | High | - | - |
| 76 | Suriapranata 2011^95^ | Ini-pgx | Indonesia | AF, HV, ATE or CHF | 1.5–3.0 | 85 | - | 100 | - | - | 55 | 57 ± 11 | Age, weight, height | CYP2C9 rs17847036; VKORC1 -1639G>A | No explicit equation | 15 | 6 | - | - | - | - | High | - | - |
| 77 | Yang 2011^96^ | Ini-pgx | China | AF, DVT, PE, HV | 1.5–3.0 | 178 | - | 100 | - | - | 42 | 55 ± 13 | Age, weight | CYP2C9*3; VKORC1 1173C > T | Dose (mg/d) = 0.982 – 0.015 (age) + 0.019 (weight) – 0.594 (CYP2C9) + 1.541 (VKORC1) | 51 | - | 36 | - | - | - | High | - | - |
| 78 | You 2011^97^ | Ini-pgx | Hong Kong | AF, HV, DVT | 2.0–3.5 | 80 | - | 100 | - | - | 49 | 59 ± 13 | Age, weight, vitamin K intake | CYP2C9*3; VKORC1 1173C>T | √dose (mg/d) =1.314 + 0.378 (VKORC1 CT) + 0.862 (VKORC1 CC) – 0.123 (50≤age<60) – 0.264 (60≤age<70) – 0.285 (age≥70) – 0.431 (CYP2C9*1*3) + 0.007 (weight) + 0.066 (√vitK) | 68 | 16 | 47 | 5 | - | IVC (n=20), r = 0.60, MPE/ MAE = 0.48 mg/d, ideal dose = 50% | High | 6 | - |
| 79 | Zambon 2011^21^ | Ini-pgx | Italy | AF, DVT, other | 2.0–3.0 | 274 | 100 | - | - | - | 65 | 74 (range 39–92) | Age, BSA | CYP2C9*2, *3; VKORC1-1639G>A; CYP4F2*3 | √dose (mg/wk) = 7.39764 – 0.02734 (age) + 1.06287 (BSA) – 1.04468 (VKORC1 AG) – 2.12117 (VKORC1 AA) – 0.78983 (CYP2C9*1*2) – 1.17138 (CYP2C9*1*3) – 1.81292 (CYP2C9*x*x) – 0.46723 (CYP4F2*1*3) – 0.71528 (CYP4F2*1*1) | 65 | 13 | 30 | 50 | 0.97 | IVC (n=97), R2 = 56%, MAE = 1 mg/d, 52% with ideal dose | High | 3 | 1 |
| 80 | Zhang 2011^98^ | Ini-pgx | USA | AF, DVT, CVA, PE, other | 1.8–3.2 | 122 | 95 | - | 5 | - | 100 | 72 ± 10 | Weight, aspirin | CYP2C9*2, *3; VKORC1 -1639  G>A; CYP4F2 *3; POR -173 C>A, -208C>T, rs2868177 | √dose (mg/wk) = 5.559 + 0.004 (weight) – 0.871 (CYP2C9*2) – 1.070 (CYP2C9*3) –0.819 (VKORC1 AG) + 0.379 (CYP4F2*3) + 0.355 (POR rs2868177) – 0.704 (POR -173CA) – 0.510 (POR -208CT) – 0.003 (aspirin) | 48 | - | - | - | - | - | High | - | - |

**Table S5. Continued**

| **#** | **Algorithm** | **Sub-type** | **Country** | **Indication** | **Target INR** | **N** | **Ethnicity (%)^a^** | | | | **Male %** | **Age, yrs (mean ± SD or median, IQR)** | **Clinical parameters** | **Genetic parameters** | **Equation^b^** | **R2^c^** | | | | **MAE (mg/d)** | **Other** | **ROB** | **# EV** | **# CUA** |
| --- | --- | --- | --- | --- | --- | --- | --- | --- | --- | --- | --- | --- | --- | --- | --- | --- | --- | --- | --- | --- | --- | --- | --- | --- |
|  |  |  |  |  |  |  | **W** | **A** | **B** | **M** |  |  |  |  |  | **All** | **Cl** | **VKORC1** | **CYP2C9** |  |  |  |  |  |
| 81 | Zhang 2011^98^ | Ini-pgx | USA | AF, DVT, CVA, PE, other | 1.8–3.2 | 122 | 95 | - | 5 | - | 100 | 72 ± 10 | Weight | CYP2C9*2, *3; VKORC1 -1639G>A; CYP4F2*3; POR -173C>A, -208C>T, rs2868177 | √dose (mg/wk) = 5.567 + 0.004 (weight) – 0.941 (CYP2C9*2) – 1.087 (CYP2C9*3) – 0.831 (VKORC1 AG) + 0.312 (CYP4F2*3) + 0.357 (POR rs2868177) – 0.680 (POR -173CA) – 0.556 (POR -208CT) | 46 | - | - | - | - | - | High | - | - |
| 82 |  | Ini-pgx | USA | AF, DVT, CVA, PE, other | 1.8–3.2 | 122 | 95 | - | 5 | - | 100 | 72 ± 10 | Weight | CYP2C9*2, *3; VKORC1 -1639G>A | √dose (mg/wk) = 5.650 + 0.005 (weight) – 0.869 (CYP2C9*2) – 1.091 (CYP2C9*3) – 0.751 (VKORC1 AG) | 40 | - | - | - | - | - | High | - | - |
| 83 | Anderson 2012^99^ (modifies IWPC based on Gage 2008) | Ini-Pgx | Nine | AF, DVT, PE, HV, stroke, other | 2.0–3.0 | See IWPC 2009 and Gage 2008 | | | | | | | Age, height, weight, race, enzyme inducers (carbamazepine, phenytoin, rifampicin, or rifampicin), amio | CYP2C9*2, *3; VKORC1-1639A | √dose (mg/wk) = 5.5922 – 0.2523 (age) + 0.0089 (height) + 0.0124 (weight) – 0.8410 (VKORC1 AG) – 1.6901 (VKORC1 AA) – 0.4199 (VKORC1 unknown) – 0.5202 (CYP2C9*1*2) – 0.9356 (CYP2C9 *1*3) – 0.9789 (CYP2C9*2*2) – 0.8313 (CYP2C9*2*3) – 2.1565 (CYP2C9*3*3) – 0.1486 (CYP2C9 unknown) – 0.0821 (Asian) – 0.2953 (Black) – 0.1661 (Mixed/Missing race) + 1.1889 (enzyme inducer) – 0.6427 (amio) – 0.3468 (amio unknown) | - | - | - | - | - | - | High | 1 | - |
| 84 | Biss 2012^100^  (***children***) | Ini-pgx | UK | FP, HV, dilated CMP, DVT, PE, aneurysm, stroke, other | 2.0–3.5 | 120 | 76 | 13 | 5 | 6 | 68 | 11 (range 1–18) | Height, indication | CYP2C9*2, *3; VKORC1 -1639G>A | √dose (mg/d) = −0.009 + 0.11 (height) + 0.357 (# VKORC1 G alleles) – 0.478 (# CYP2C9* 3 alleles) – 0.277 (#CYP2C9*2 alleles) + 0.186 (none-Fontan procedure) | 72 | 33 | 27 | 13 | - | - | High | 5 | - |
| 85 | Bress 2012^101^ | Ini-pgx | USA | DVT, PE, AF, HV, stroke, TIA | 2.0–3.5 | 258 | - | - | 100 | - | 26 (of 260) | 55 ± 16 (for 260) | Age, BSA, HTN | CYP2C9*2, *3, *5, *6, *8, *11; VKORC1 -1639G>A | Log dose (mg/wk) = 3.52 – 0.006 (age) + 0.38 (BSA) – 0.15 (HTN) – 0.23 (CYP2C9) – 0.24 (VKORC1) | 37 | 23 | - | - | - | - | High | 1 | - |
| 86 |  | Ini-pgx | USA | DVT, PE, AF, HV, stroke, TIA | 2.0–3.5 | 258 | - | - | 100 | - | 26 (of 260) | 55 ± 16 (for 260) | Age, BSA, HTN | Above plus CYP4F2*3 | Log dose (mg/wk) = 3.52 – 0.006 (age) + 0.38 (BSA) – 0.15 (HTN) – 0.23 (CYP2C9) – 0.24 (VKORC1) – 0.0002 (CYP4F2) | 36 | 23 | - | - | - | - | High | - | - |
| 87 |  | Ini-pgx | USA | DVT, PE, AF, HV, stroke, TIA | 2.0–3.5 | 258 | - | - | 100 | - | 26 (of 260) | 55 ± 16 (for 260) | Age, BSA, HTN | Above plus NQO1*2 | Log dose (mg/wk) = 3.50 – 0.006 (age) + 0.38 (BSA) – 0.15 (HTN) – 0.23 (CYP2C9) – 0.25 (VKORC1) – 0.004 (CYP4F2) + 0.05 (NQO1*2) | 36 | 23 | - | - | - | - | High | - | - |
| 88 |  | Ini-pgx | USA | DVT, PE, AF, HV, stroke, TIA | 2.0–3.5 | 258 | - | - | 100 | - | 26 (of 260) | 55 ± 16 (for 260) | Age, BSA, HTN, ancestry |  | Log dose (mg/wk) = 3.48 – 0.006 (age) + 0.39 (BSA) – 0.15 (HTN) – 0.22 (CYP2C9) – 0.24 (VKORC1) + 0.002 (CYP4F2) + 0.05 (NQO1*2) + 0.003 (West African ancestry) | 35 | 23 (-ancestry) | - | - | - | - | High | - | - |

**Table S5. Continued**

| **#** | **Algorithm** | **Sub-type** | **Country** | **Indication** | **Target INR** | **N** | **Ethnicity (%)^a^** | | | | **Male %** | **Age, yrs (mean ± SD or median, IQR)** | **Clinical parameters** | **Genetic parameters** | **Equation^b^** | **R2^c^** | | | | **MAE (mg/d)** | **Other** | **ROB** | **# EV** | **# CUA** |
| --- | --- | --- | --- | --- | --- | --- | --- | --- | --- | --- | --- | --- | --- | --- | --- | --- | --- | --- | --- | --- | --- | --- | --- | --- |
|  |  |  |  |  |  |  | **W** | **A** | **B** | **M** |  |  |  |  |  | **All** | **Cl** | **VKORC1** | **CYP2C9** |  |  |  |  |  |
| 89 | Bress 2012^101^ | Ini-pgx | USA | DVT, PE, AF, HV, stroke, TIA | 2.0–3.5 | 50 | 100 | - | - | - | 38 (of 53) | 58 ± 15 (for 53) | Age, BSA, AF | CYP2C9*2, *3; VKORC1 -1639G>A | Log dose (mg/wk) = 3.24 – 0.007 (age) + 0.55 (BSA) – 0.39 (AF) – 0.39 (CYP2C9) – 0.34 (VKORC1) | 58 | 36 | - | - | - | - | High | - | - |
| 90 |  | Ini-pgx | USA | DVT, PE, AF, HV, stroke, TIA | 2.0–3.5 | 50 | 100 | - | - | - | 38 (of 53) | 58 ± 15 (for 53) | Age, BSA, AF | Above plus CYP4F2*3 | Log dose (mg/wk) = 3.32 – 0.008 (age) + 0.48 (BSA) – 0.34 (AF) – 0.35 (CYP2C9) – 0.33 (VKORC1) + 0.20 (CYP4F2) | 63 | 36 | - | - | - | - | High | - | - |
| 91 |  | Ini-pgx | USA | DVT, PE, AF, HV, stroke, TIA | 2.0–3.5 | 50 | 100 | - | - | - | 38 (of 53) | 58 ± 15 (for 53) | Age, BSA, AF | Above plus NQO1*2 | Log dose (mg/wk) = 3.12 – 0.008 (age) + 0.53 (BSA) – 0.37 (AF) – 0.29 (CYP2C9) – 0.36 (VKORC1) + 0.17 (CYP4F2) + 0.29 (NQO1*2) | 68 | 36 | - | - | - | - | High | - | - |
| 92 |  | Ini-pgx | USA | DVT, PE, AF, HV, stroke, TIA | 2.0–3.5 | 50 | 100 | - | - | - | 38 (of 53) | 58 ± 15 (for 53) | Age, BSA, AF, native American ancestry | CYP2C9*2, *3; VKORC1 -1639G>A; CYP4F2*3; NQO1*2 | Log dose (mg/wk) = 3.10 – 0.008 (age) + 0.553 (BSA) – 0.398 (AF) – 0.276 (CYP2C9) – 0.373 (VKORC1) + 0.181 (CYP4F2) + 0.354 (NQO1*2) – 0.19 (ancestry) | 70 | 36 (-ancestry) | - | - | - | - | High | - | - |
| 93 | Cini 2012^102^ | Ini-pgx | Italy | DVT, HV, PE, and other | 2.0–3.5 | 55 | 98 | 2 | - | - | 58 | 69 ± 16 | Age, height, weight, gender, smoking, dietary intake, VTE, DM | CYP2C9*2, *3; VKORC1 -1639G>A, 3730G>A | √dose (mg/d) = 00.833 – 0.255 (male) – 0.007 (age) + 0.011 (height) + 0.005 (weight) + 0.385 (smoking) + 0.211 (vegetable intake) + 0.119 (VTE) + 0.328 (DM) – 0.458 (# CYP2C9 variants) – 0.571 (# VKORC1 -1639 A alleles) + 0.025 (# VKORC1 3730 A alleles) | - | - | - | - | - | - | High | 1 | - |
| 94 | Chan 2012^103^ | Ini-pgx | Singapore | AF, HV, TE, stroke, RHD | 2.0–3.0 | 248 | - | 100 | - | - | 56 (of 275) | mean 56 (for 275) | Age, weight, ethnicity | CYP2C9*3, VKORC1 (381) haplotype, CYP4F2*3 | No explicit equation | 61 | 33 | 30 | 7 |  | 48% with ideal dose | High | - | - |
| 95 | El Din 2012^104^ | Ini-pgx | Egypt | AF, HV, other | 2.0–3.0 | 46 | - | - | - | 100 | 44 | 41 ± 14 | - | CYP2C9*2, *3; VKORC1 1173C>T | Dose (mg/wk) = 35.27 + 14.053 (VKORC1 non-TT) – 13.982 (CYP2C9 *1*2) – 15.585 (CYP2C9*1*3) – 20.361 (CYP2C9*2*2) | 60 | - | 32 | 16 | - | - | High | - | - |
| 96 | Kurnik 2012^105^ (updates IWPC 2009) | Ini-pgx | Israel | - | 2.0–3.5 | 210 | 100 | - | - | - | 48 | 61 ± 16 | Age, height, weight, race, enzyme inducers, amio | CYP2C9*2, *3; VKORC1-1639A, D36Y; CYP4F2*3 | No explicit equation | 47 | - | - | - | 1.80 | - | High | - | - |
| 97 | Lee 2012^106^ | Ini-pgx | Korea | HV | 2.0–3.0 | 191 | - | 100 | - | - | 33 | 58 ± 10 | Age | CYP2C9*2, *3; VKORC1 1173 C>T; CYP4F2*3 | Dose (mg/d) = 11.305 – 2.082 (# VKORC1 T alleles) – 1.615 (# CYP2C9 variant alleles) – 0.037 (age) + 0.983 (CYP4F2 *3*3) | 38 | 4 | 27 | 5 | - | - | High | - | 1 |
| 98 | Liang 2012^107^ | Ini-pgx | China | AF, HV, DVT/PE | 2.0–3.0 | 115 | - | 100 | - | - | 62 | 65 ± 13 | Age, BSA | CYP2C9*3; VKORC1-1639G>A; CYP4F2*3 | No explicit equation | 42 | - | 12 | 11 | 0.64 | - | High | - | - |
| 99 | Lou 2012 (from Zhao 2014^68^) | Ini-pgx | China | - | 1.5–3.0 | 488 | - | 100 | - | - | - | - | Age, weight, height, digoxin, amio | CYP2C9, VKORC1, CYP4F2 | No explicit equation | 65 | - | - | - | - | - | Unclear | 2 | - |

**Table S5. Continued**

| **#** | **Algorithm** | **Sub-type** | **Country** | **Indication** | **Target INR** | **N** | **Ethnicity (%)^a^** | | | | **Male %** | **Age, yrs (mean ± SD or median, IQR)** | **Clinical parameters** | **Genetic parameters** | **Equation^b^** | **R2^c^** | | | | **MAE (mg/d)** | **Other** | **ROB** | **# EV** | **# CUA** |
| --- | --- | --- | --- | --- | --- | --- | --- | --- | --- | --- | --- | --- | --- | --- | --- | --- | --- | --- | --- | --- | --- | --- | --- | --- |
|  |  |  |  |  |  |  | **W** | **A** | **B** | **M** |  |  |  |  |  | **All** | **Cl** | **VKORC1** | **CYP2C9** |  |  |  |  |  |
| 100 | Moreau 2012^108^ (***children***) | Ini-pgx | France | - | 1.5–4.0 (from Hamberg 2013^109^) | 83 | Y | - | - | - | 55 | 8 ± 6 | Height, target INR | CYP2C9*2, *3; VKORC1 -1639G>A | Dose (mg/wk) = −10.77 + 0.28 (height) – 5.4 (# VKORC1 A alleles) + 7.83 (target INR = 2.5) + 11.52 (target INR = 3.3) − 3.29 (# CYP2CP variant alleles) (Hamberg 2013^109^; Marek 2016^110^) | 70 | 53 | 18 | 2 | - | 87% with doses within 1 mg/d | High | 3 | - |
| 101 | Pathare 2012^111^ | Ini-pgx | Oman | AF, ATE, HV, PE, other | 2.0–3.0 | 142 | - | - | - | 100 | 57 | 52 ± 18 | Age, weight, gender, indication (AF or DVT/PE) | CYP2C9*2, *3, *8; VKORC1 -1639G>A | Ln dose (mg/d) = 0.69 – 0.001 (age) + 0.0008 (weight) + 0.034 (male) + 0.0123 (DVT/PE) + 0.102 (AF) – 0.036 (CYP2C9 *1*2) – 0.088 (*1*3) – 0.117 (*1*8) – 0.240 (*2*3) – 0.482 (*3/*3) – 0.164 (VKORC1 GA) – 0.404 (VKORC1 AA) | 62 |  | 45 | 17 | - | MPE = 0.26 mg/d  IVC (n=70), MPE = -0.26 mg/d | High | - | - |
| 102 | Pavani 2012^112^ | Ini-pgx | India | HV, DVT, AF | - | 125 | - | Y | - | - | - | - | Age, BMI, gender, vitamin K intake | CYP2C9*2, *3; VKORC1  -1639 G>A, 3730G>A, 6009C>T | Dose (mg/wk) = 34.9588747 – 0.1850797953 (age) + 8.107223532 (male) + 0.1390121899 (BMI) + 6.697769856 (CYP2C9*2) – 8.275810018 (CYP2C9*3) – 10.72676854 (VKORC1 3730G>A) + 8.873462677 (VKORC1 6009C>T) + 11.14958922 (VKORC1 -1639G>A) – 4.824633754 (vit K intake) | - | - | - | - | - | r = 0.64 | High | - | - |
| 103 | Pavani 2012^113^ | Ini-pgx | India | HV, DVT, AF | 2.0–3.5 | 160 | - | Y | - | - | 53 | 39 ± 15 | Age, gender, BMI | CYP2C9*2, *3 *8; VKORC1 -1639G>A, 3730G>A, 6009C>T; CYP4F2*3; GGCX 8016G>A | Males: dose (mg/wk) = 7.970140851 – 0.1013885349 (age) + 1.449999606 (BMI) + 8.054730665 (CYP2C9*2) + 1.726919455 (CYP2C9*3) – 4.437335987 (VKORC1 3730G>A) – 2.771903482 (VKORC1 6009C>T) + 1.511628517 (VKORC1 -1639G>A) + 1.570215716 (CYP4F2*3) + 2.409742997 (GGCX) + 11.05198035 (CYP2C9*8)  Females: dose (mg/wk) = 44.53497515 – 0.05440552061 (age) – 0.2938201651 (BMI) – 1.576151039 (CYP2C9*2) – 5.950436495 (CYP2C9*3) + 2.983528309 (VKORC1 3730G>A) + 8.699010214 (VKORC1 6009C>T) – 11.00733747 (VKORC1 -1639G>A) – 2.282918521 (CYP4F2 V433M) – 4.097105716 (GGCX) – (2.96671589 × CYP2C9*8) [considered as one model] | 61 | 13 | - | - | - | - | High | - | - |
| 104 | Ramirez 2012^114^ | Ini-pgx | USA | DVT, PE, AF, stroke, orthopaedic, other | 2.0–3.0 | 1167 | 88 | - | 12 | - | 55 | 66 (95% CI intervals 35–87) | Age, gender, BSA, smoking, AF, DVT, PE, amio, race | CYP2C9*2, *3, *6, *8; VKORC1 -1639G>A | No explicit equation | 50 (White 50, Black 30) | White 23, Black 24 | - | - | 1.40 (White 1.33, Black 1.84) | - | High | 1 | - |
| 105 | Ramirez 2012^114^ | Ini-pgx | USA | DVT, PE, AF, stroke, orthopedic, other | 2.0–3.0 | 1167 | 88 | - | 12 | - | 55 | 66 (95% CI intervals 35–87) | Age, gender, BSA, smoking, AF, DVT, PE, amio, race | CYP2C9*2, *3, *6, *8; VKORC1 -1639G>A; CALU rs339097; CYP4F2*3 | Log dose (mg/wk) = 5.9487517 – 0.0073436353 (White) – 0.025161445 (age) + 0.058138499 (male) + 1.1848957 (BSA) + 0.068020571 (smoker) + 0.058578086 (DVT/PE) – 0.10646416 (AF) – 0.8142 521 (amio) – 0.64877338 (# CYP2C9*2 alleles) – 1.0601067 (CYP2C9*3) – 1.9737831 (CYP2C9*6) – 1.0622944 (CYP2C9*8) + 0.24749973 (CYP4F2*3) – 0.31996754 (CALU) – 0.87262446 (VKORC1) | 52 (White 53, Black 41) | White 23, Black 24 | - | - | 1.37 (White1.30, Black 1.77) | - | High | - | - |
| 106 | Tan 2012^115^ | Ini-pgx | China | HV | 1.7–3.0 | 321 | - | 100 | - | - | 41 | 46 ± 11 | Age, BSA, INR-increasing drugs, smoking, stroke, HTN | CYP2C9*3, VKORC1 -1639 G>A | √dose (mg/d) = 2.140 − 0.370 (VKORC1) − 0.332 (CYP2C9*3) + 0.324 (BSA) − 0.004 (age) − 0.231 (# increasing INR drugs) + 0.105 (smoker) − 0.135 × (preoperative stroke history) − 0.108 (HTN) | 55 | - | - | - | - | IVC (n=320), r = 0.638, MPE = -0.07 mg/d, 63% ideal dose | High | 6 | - |

**Table S5. Continued**

| **#** | **Algorithm** | **Sub-type** | **Country** | **Indication** | **Target INR** | **N** | **Ethnicity (%)^a^** | | | | **Male %** | **Age, yrs (mean ± SD or median, IQR)** | **Clinical parameters** | **Genetic parameters** | **Equation^b^** | **R2^c^** | | | | **MAE (mg/d)** | **Other** | **ROB** | **# EV** | **# CUA** |
| --- | --- | --- | --- | --- | --- | --- | --- | --- | --- | --- | --- | --- | --- | --- | --- | --- | --- | --- | --- | --- | --- | --- | --- | --- |
|  |  |  |  |  |  |  | **W** | **A** | **B** | **M** |  |  |  |  |  | **All** | **Cl** | **VKORC1** | **CYP2C9** |  |  |  |  |  |
| 107 | Tatarunas 2012^116^ | Ini-pgx | Lithuania | HV | 2.0–3.5 | 189 (185 in abstract) | 100 | - | - | - | 57 | 66 (range 18–85) | Age, weight, liver function, amio, ibuprofen /diclofenac, diuretic, β-blockers, cephalosporin, omeprazole | CYP2C9*3, VKORC1 -1639 G>A | Dose (mg/d) = 4.653 − 0.046 (age) + 0.028 (weight) − 1.029 (liver function) + 2.301 (cephalosporin) − 1.941 (amio) − 1.574 (ibubrofen/diclofenac) − 1.539 (omeprazole) − 1.151 (loop diuretic) − 0.738 (β-blocker) + 1.430 (VKORC1) − 1.014 (CYP2C9) | 43 | 32 | - | - | - | - | High | - | - |
| 108 | Teh 2012^117^ | Ini-pgx | Malaysia | VT, AF, HV | 2.0–4.0 | 86 | - | 100 | - | - | 54 | 59 ± 11 | Age, weight, height | CYP2C9*3; VKORC1 -1639G>A, 1173C>T | Dose (mg/d) = 7.728 − 0.993 (VKORC1 -1639G>A) − 0.564 (VKORC1 1173C>T) − 0.811 (CYP2C9*3) − 0.037 (age) − 0.008 (weight) + 2.180 (height) | 37 | 9 | 27 | <1 | - | - | High | 1 | - |
| 109 | Wei 2012^118^ | Ini-pgx | China | AF | 1.5–3.0 | 260 | - | 100 | - | - | 49 | 67 ± 13 | Age, weight, previous TE, β-blockers, amio | CYP2C9*3; VKORC1 1173C>T; CYP4F2*3 | Dose (mg/d) = 3.47 – 0.022 (age) + 0.017 (weight) + 0.189 (TE) – 0.283 (β-blocker) – 0.471 (amio) – 0.586 (CYP2C9*1/*3) – 0.296 (VKORC1 CT) – 0.648 (VKORC1 TT) + 0.219 (CYP4F2*3*3) | 52 | 29 | 32 | 21 | - | IVC (n=65): r = 0.658 | High | 5 | - |
| 110 | Xu 2012^22^ | Ini-pgx | China | AF, DVT, PE, HV, CMP, other | 1.6–3.0 | 207 | - | 100 | - | - | 54 | 57 ± 16 | Age, BSA | CYP2C9*3; VKORC1 -1639 G>A | √dose (mg/d) = 1.24 – 0.004 (age) + 0.37 (BSA) – 1.00 (CYP2C9*3*3) – 0.31 (CYP2C9*1*3) + 0.20 (VKORC1 AG) + 0.62 (VKORC1 GG) | 38 | - | - | - | - | IVC (n=103): MAE = 0.73 mg/d, R2 = 31%. Full cohort (n= 310): ideal dose = 51% | High | 1 | - |
| 111 | Zhang 2012^119^ | Ini-pgx | China | DVT, PE | 2.0–3.0 | 297 | - | 100 | - | - | 50 | 64 (18–87) | Age, weight | CYP2C9*3; VKORC1 1173  C>T/3730G>A | Dose (mg/d) = 3.747 – 0.030 (age) + 0.022 (weight) + 1.756 (VKORC1 1173C>T) – 0.682 (CYP2C9*3) | 37 | 15 | 20 | 3 | - | IVC (n=31): 65% within 1mg/d | High | 1 | - |
| 112 | Zhong 2012^120^ | Ini-pgx | China | HV | 1.8–3.0 | 591 | - | 100 | - | - | 57 (of 845) | 48 (39–56) (for 845) | Age, BSA, amio, fluconazole, diltiazem | CYP2C9*3; VKORC1 -1639 G>A; CYP4F2*3 | √dose (mg/d) = 1.68143 – 0.0029 (age) + 0.30784 (BSA) – 0.2633 (VKORC1) – 0.19114 (CYP2C9*3) + 0.14735 (CYP4F2*3) – 0.1797 (amio) –0.4138 (fluconazole) – 0.1888 (diltiazem) | 44 | 5 | 24 | 4 | - | IVC (n=254): r = 0.630 | High | 3 | - |
| 113 | Zhu 2012^121^ | Ini-pgx | China | DVT, PE | 2.0–3.0 | 322 | - | 100 | - | - | 49 | 65 (range 19–88) | Age, weight, WBC | CYP2C9*3; VKORC1 1173C>T | Dose (mg/d) = 8.388 – 1.975 (VKORC1 1173C>T) –0.034 (age) – 0.021 (weight) –0.690 (CYP2C9*3) – 0.046 (WBC) | 38 | - | 23 | - | - | - | High | - | - |
| 114 | Daneshjou 2013^122^ | Ini-pgx | USA | - | 2.0–3.0 | 188 | 100 | - | - | - | - | 59 ± 16 | Age, weight, race, amio, losartan | CYP2C9*3; VKOR C1 -1639G>A; 49 SNPs in 7 genes | No explicit equation | 48 | - | - | - | - | - | High | - | - |
| 115 |  | Ini-pgx | USA (IWPC sites) | - | 2.0–3.0 | 233 | 100 | - | - | - | - | - | Age, height, weight, race | CYP2C9 *2, *3; VKORC1 -1639 G>A/1173 C>T | No explicit equation | - | - | - | - | - | - | High | - | - |
| 116 |  | Ini-pgx | USA (IWPC sites) | - | 2.0–3.0 | 302 | - | - | 100 | - |  | 57 ± 15 | Age, height, weight, race, aspirin, amio | CYP2C9*2, *3; VKORC1 -1639 G>A/1173 C>T; 122 SNPs/7 genes | √dose (mg/wk) = IWPC 2000 equation –0.2935 (Aggregate number of minor alleles in the metabolic pathway) | 27 | - | - | - | - | - | High | - | - |

**Table S5. Continued**

| **#** | **Algorithm** | **Sub-type** | **Country** | **Indication** | **Target INR** | **N** | **Ethnicity (%)^a^** | | | | **Male %** | **Age, yrs (mean ± SD or median, IQR)** | **Clinical parameters** | **Genetic parameters** | **Equation^b^** | **R2^c^** | | | | **MAE (mg/d)** | **Other** | **ROB** | **# EV** | **# CUA** |
| --- | --- | --- | --- | --- | --- | --- | --- | --- | --- | --- | --- | --- | --- | --- | --- | --- | --- | --- | --- | --- | --- | --- | --- | --- |
|  |  |  |  |  |  |  | **W** | **A** | **B** | **M** |  |  |  |  |  | **All** | **Cl** | **VKORC1** | **CYP2C9** |  |  |  |  |  |
| 117 | Ekladious 2013^123^ | Ini-pgx | Egypt | AF, DVT, HV, other | 2.0–3.0 | 50 | - | - | - | 100 | 43 | 39 ± 12 | Age | VKORC1 1173C>T | Dose (mg/wk) = 65.226 – 0.422 (age) – 9.474 (VKORC1 TT) | 21 | 13 | 7 | - | - | - | High | 1 | - |
| 118 | Kabagambe 2013^124^ | Ini-pgx | USA | - | 2.0–3.0 | 172 | 62 | - | 38 | - | 48 | 62 ± 14 | Age, gender, BMI, physical activity, energy | CYP2C9*2, *3; VKORC1 -1639 G>A | √dose (mg/d) = 2.28866 – 0.00967 (age) + 0.05395 (male) + 0.01353 (BMI) + 0.19224 (physical activity) – 0.31997 (VKORC1 CT/TT) + 0.24312 (CYP2C9*1*1) + 0.00002598 (energy) | 33 | - | - | - | - | - | High | - | - |
| 119 |  | Ini-pgx | USA | - | 2.0–3.0 | 172 | 62 | - | 38 | - | 48 | 62 ± 14 | Age, gender, BMI, physical activity, energy, Vitamin K | CYP2C9*2, *3; VKORC1 -1639 G>A | √dose (mg/d) = 2.25771 – 0.01050 (age) + 0.05851 (male) + 0.01391 (BMI) + 0.19744 (activity) – 0.29956 (VKORC1 CT/TT) + 0.21245 (CYP2C9) + 0.00002234 (energy) + 0.00050834 (vit K) | 34 | - | - | - | - | - | High | - | - |
| 120 | Mazzaccara 2013^125^ | Ini-pgx | Italy | HV, AF, dilated CMP, DVT, PE | 2.0–3.0 | 266 | 100 | - | - | - | 55 | 67 ± 11 | Age, gender | CYP2C9*2, *3, CYP4F2*3, VKORC1 -1639 G>A, 1173 C>T, 3730G>A | No explicit equation (i.e. no intercept) | 58 | 11 | 31 | 15 | - | - | High | - | - |
| 121 | Natarajan 2013^126^ | Ini-pgx | India | AF, DVT, PE, HV, stroke, other | 2.0–3.5 or lower | 103 | - | 100 | - | - | 54 | 52 ± 14 | Age, weight | VKORC1 1639G>A (or 1173C>T) | Dose (mg/d) = 3.291 – 0.048 (weight) – 0.034 (age) – 1.15 (VKORC1) | 21 | - | - | - | - | - | High | - | - |
| 122 | Nguyen 2013^127^ (***children***) | Ini-pgx | USA | Congenital and acquired heart diseases | 1.5–4.0 | 37 | 73 | 8 | 19 | - | 70 | 10 ± 6 | Age, target INR | CYP2C9*2, *3; VKORC1 1173C>T | Dose (mg/kg/d) = − 0.09 − 0.0006 (age) + 0.11 (VKORC1 CC) + 0.043 (VKORC1 CT) + 0.045 (CYP2C9*1*1) + 0.039 (CYP2C9 *1*2) + 0.073 (target INR) | 82 | 30 | 47 | 5 | - | - | High | 2 | - |
| 123 | Ozer 2013^128^ | Ini-pgx | Turkey | AF, DVT, PE, HV, cardiac surgery | 2.0–3.0 | 107 | - | - | - | 100 | 50 | 54 ± 14 | Age | CYP2C9*2*3; VKORC1 -1639G>A; CYP4F2*3 | Dose (mg/d) = 8.308 − 1.499 (# VKORC1 variant alleles) − 1.330 (# CYP2C9 variant alleles) − 0.028 (age) + 0.505 (# CYP4F2 variant alleles) | 39 | 3 | 15 | 19 | - | - | High | - | - |
| 124 | Park 2013^129^ | Ini-pgx | Korea | Stroke, arrythmia, HV, other | 1.7–3.5 | 204 | - | 100 | - | - | 63 | 66 ± 11 | Age, BMI | CYP2C9*3; VKORC1 3730G>A | Ln dose (mg/d) = 1.142 − 0.01 (age) + 0.017 (BMI) + 0.499 (VKORC1) + 0.370 (CYP2C9) | 25 | - | - | - | - | - | High | - | - |
| 125 | Perera 2013^130^ (updates IWPC) | Ini-pgx | USA | AF, DVT/PE, HV, stroke, CMP, other | 2.0–3.0 | 504 | - | - | 100 | - | 40 (of 533) | 58 ± 15 (for 533) | Age, height, weight, race, inducers, amio | CYP2C9*2, *3; VKORC1 -1639 G>A; rs12777823 | Dose (mg/week) = IWPC 2009 weekly dose (mg) − 6.92 (rs12777823 AG) − 9.34 (rs12777823 AA) | 27 | - | - | - | - | - | High | 1 | - |
| 126 | Shahin 2013^131^ (updates Shahin 2011) | Ini-pgx | Egypt | AF, DVT/PE, HV, CVA, CMP, others | 1.5–3.5 | 195 | - | - | - | 100 | 45 (of 207) | 47 ± 15 (for 207) | Age, PE, smoking | CYP2C9 *2, *3, *4, *5, *8; VKORC1 -1639 G>A, Asp36 Tyr; APOE ε2 | √dose (mg/wk) = 8.19 − 0.62 (# VKORC1 -1639G>A variants) − 0.32 (age in decades) − 0.56 (# CYP2C9 variants) + 1.21 (PE) − 0.59 (APOE) + 0.96 (VKORC1 Asp36Tyr) + 0.58 (smoker) | 37 | 14 | 14 | 7 | - | - | High | - | - |
| 127 | Tan 2013^132^ | Ini-pgx | China | HV | 1.7–3.0 | 317 | - | 100 | - | - | 30 | 45 ± 11 | Age, BSA | CYP2C9 *3; VKORC1 -1639 G>A; CYP4F2*3 | √dose (mg/d) = 2.027 − 0.313 (# of VKORC1 variant) − 0.365 (# CYP2C9 variants) + 0.107 (CYP4F2 CT/TT) + 0.309 (BSA) − 0.004 (age) | 49 | 4 | 25 | 16 | - | - | High | - | - |

**Table S5. Continued**

| **#** | **Algorithm** | **Sub-type** | **Country** | **Indication** | **Target INR** | **N** | **Ethnicity (%)^a^** | | | | **Male %** | **Age, yrs (mean ± SD or median, IQR)** | **Clinical parameters** | **Genetic parameters** | **Equation^b^** | **R2^c^** | | | | **MAE (mg/d)** | **Other** | **ROB** | **# EV** | **# CUA** |
| --- | --- | --- | --- | --- | --- | --- | --- | --- | --- | --- | --- | --- | --- | --- | --- | --- | --- | --- | --- | --- | --- | --- | --- | --- |
|  |  |  |  |  |  |  | **W** | **A** | **B** | **M** |  |  |  |  |  | **All** | **Cl** | **VKORC1** | **CYP2C9** |  |  |  |  |  |
| 128 | Bazan 2014^133^ | Ini-pgx | Egypt | AF, ATE, HV | 2.0–3.5 | 63 | - | - | - | 100 | 49 | 46 ± 13 | Age, smoking | CYP2C9*3; VKORC1 -1639G>A | Dose = 15.147 − 2.628 ((# VKORC1 variants) − 2.786 (# CYP2C9 variants) + 4.614 (ex-smoker) − 0.113 (age) | 43 | 16 | 20 | 8 | - | - | High | - | - |
| 129 | Bosch 2014^134^ | Ini-pgx | Puerto Rico | AF, DVT, PE | 2.0–3.5 | 121 | 90 | - | - | - | 100 | 68 ± 9 (for 138) | Age, BSA, admixture index (AI), target INR, statin, amio, smoking, DM, vit K intake | CYP2C9*2, *3, *4, *6; VKORC1 -1639G>A | Ln dose (mg/d) = 2.20 – 0.0106 (age) + 0.122 (BSA) – 0.190 (CYP2C9 variant alleles) – 0.229 (VKORC1 AA) – 0.636 (VKORC1 GA) – 0.0742 (Taino) – 0.118 (African) – 0.120 (Mixed) + 0.216 (target INR) – 0.0448 (statin) – 0.233 (amio) –0.126 (smoker) + 0.135 (DM) + 0.09837 (vit K) | 48 | 13 (-admixture) | 27 | 8 | - | MSE = 0.10 | High | - | - |
| 130 | Chen 2014^135^ | Ini-pgx | China | HV | 1.6–2.5 | 551 | - | 100 | - | - | 56 | 51 (43–60) | Age, BSA, target INR, DM, amio, digoxin | CYP2C9*3; VKORC1 3730G>A, CYP4F2*3 | Dose (mg/d) = 0.135 + 1.7816 (VKORC1) – 1.2146 (CYP2C9) + 1.2886 (BSA) – 0.0196 (age) + 0.7086 (target INR) + 0.1596 (CYP4F2) + 0.3736 (DM) – 0.5816 (amio) – 0.2526 (digoxin) | 45 | 10 | 27 | 7 | - | + EV cohort (n=787): r =0.648, ideal dose =57% | High | 4 | - |
| 131 | Daneshjou 2014^136^ | Ini-pgx | USA | DVT, PE | - | 476 | - | - | 100 | - | 37 | mean 57 | Age, weight, amio, VTE [aspirin + ancestry in ‘discovery’ cohort  model] | CYP2C9*2, *3, *5, *6, *8, *11; VKORC1 -1639G>A; FPGS rs7856096 | No explicit equation | - | - | - | - | - | - | High | - | - |
| 132 | Grossi 2014^137^ | Ini-pgx | Italy | AF, HV, DVT, PE, CMP, stroke, other | 2.0–4.0 | 377 | 100 | - | - | - | 49 | 76 (70–80) | Gender, height, BMI, DVT, PE, CMP, stroke, diuretics, amio, statins, others | CYP2C9*2, *3, *6, *11; VKORC1 -1639 G>A, 1173C>T, 1542G>C | No explicit equation | 48 |  |  |  | 0.82 | 70% with ideal dose | High | - | - |
| 133 | Issac 2014^138^ | Ini-pgx | Egypt | - | 2.0–3.0 | 84 | - | - | - | 100 | 49 | 41 ± 13 | Age | VKORC1 1173C>T, MDR1 3435C>T | Dose (mg/wk) = 64.909 – 0.282 (age) – 13.390 (VKORC1 TT) – 7.164 (MDR1 CC) | 21 | 5 | 13 | - | - | - | High | - | - |
| 134 |  | Ini-pgx | Egypt | - | 2.0–3.0 | 84 | - | - | - | 100 | 49 | 41 ± 13 | Age | MDR1 3435C>T, EPHX1 H139R and PZ A-13G | Dose (mg/wk) = 52.928 – 0.289 (age) + 9.709 (combined genotype TT/RH or RR/AA) | 8 | 5 | - | - | - | - | High | - | - |
| 135 | Krishna Kumar 2014^139^ | Ini-pgx | India | RHD (HV, AF, stenosis), IHD, DVT, dilated CMP, stroke, other | 2.0–3.5 | 240 | - | 100 | - | - | 37 | 43 ± 11 | Age, weight, HV | CYP2C9*2, *3; VKORC1 -1639 G>A, 3730G>A, 1173C>T, 2255 C>T; CYP4F2*3; GGCX rs11676382 | Log10 dose (mg/d) = 0.656 –0.187 (VKORC1 -1639G>A) + 0.003 (weight) − 0.196 (CYP2C9*3) − 0.144 (CYP2C9*2) + 0.083 (VKORC1 3730G>A) − 0.003 (age) + 0.033 (CYP4F2*3) + 0.037 (HV) − 0.074 (VKORC1 1173C>T) − 0.097 (VKORC1 2255C>T) − 0.130 (GGCX) | 60 | - | 32 | 12 | - | 61% within 1 mg/d | High | - | - |

**Table S5. Continued**

| **#** | **Algorithm** | **Sub-type** | **Country** | **Indication** | **Target INR** | **N** | **Ethnicity (%)^a^** | | | | **Male %** | **Age, yrs (mean ± SD or median, IQR)** | **Clinical parameters** | **Genetic parameters** | **Equation^b^** | **R2^c^** | | | | **MAE (mg/d)** | **Other** | **ROB** | **# EV** | **# CUA** |
| --- | --- | --- | --- | --- | --- | --- | --- | --- | --- | --- | --- | --- | --- | --- | --- | --- | --- | --- | --- | --- | --- | --- | --- | --- |
|  |  |  |  |  |  |  | **W** | **A** | **B** | **M** |  |  |  |  |  | **All** | **Cl** | **VKORC1** | **CYP2C9** |  |  |  |  |  |
| 136 | Lou 2014 (from Jiang 2018^140^ and Xie 2020^141^) | Ini-pgx | China | HV, AF, PE | 1.5–3.0 | 323 | - | Y | - | - | - | - | Age, weight, height, amio, digoxin | CYP2C9*3; VKORC1 -1639G>A, CYP4F2*3 | Dose of warfarin (mg/d) = 1.087 + 2.226 (VKORC1 AG) + 3.844 (VKORC1 GG) − 1.284 (CYP2C9*1*3) − 2.182 (CYP2C9*3 *3) + 0.221 (CYP4F2*1*3) + 0.336 (CYP4F2*3*3) − 0.018 (age) + 0.015 (weight) + 0.013 (height) − 0.777 (amio) − 0.379 (digoxin) | 65 | - | - | - | - | - | Unclear | 1 | 1 |
| 137 | Pavani 2014^142^ | Ini-pgx | India | HV, DVT, AF | 2.0–3.5 | 125 | - | 100 | - | - | 53 | 38 ± 15 | Age, BMI, gender, TSH | CYP2C9*2, *3; VKORC1 3730G>A, 6009C>T, -1639G>A; CYP4F2*3 | Dose (mg/wk) = 17.92 − 0.00317 x1^2 − 0.0155 x1 x2 + 0.0338 x1 x3 + 0.0278 x1 x4 − 0.0609 x1 x5 − 0.0851 x1 x6 − 0.0404 x2^2 − 0.516 x2 x3 + 1.32 x2 x4 + 0.00540 x2 x5 + 0.555 x2 x6 − 1.05 x3^2 + 0.891 x3 x4 + 18.19 x3 x5 − 2.96 x3 x6 + 4.60 x4^2 − 7.13 x4 x5 − 1.13 x4 x6 + 13.86 x5^2 + 13.86 x5 x6 − 4.60 x6^2 + 0.528 x1 + 1.54 x2 + 3.82 x3 − 35.52 x4 − 23.81 x5 − 0.738 x6 + 3.51 x7 + 3.37 x8 + 0.87 x9 − 0.002 x10^2 − 0.31 x10  x1 = age; x2 = BMI; x3 = CYP2C9*3; x4 = VKORC1 3730G>A; x5 = VKORC1 6009C>T; x6 = CYP4F2; x7 = gender; x8 = CYP2C9*2; x9 = VKORC1 -1639; x10 = TSH | - | - | - | - | - | r = 0.62 | High | - | - |
| 138 |  | Ini-pgx | India | HV, DVT, AF | 2.0–3.5 | 125 | - | 100 | - | - | 53 | 38 ± 15 | Age, BMI, gender, vitamin K intake, TSH | CYP2C9*2, *3; VKORC1 3730 G>A, 6009C>T, -1639G>A; CYP4F2*3 | Dose (mg/wk) = 38.27 − 0.13 (age) + 0.0435 (BMI) − 5.64 (CYP2C9*3) + 0.0746 (VKORC1 3730G>A) + 1.45 (VKORC1 6009C>T) + 0.0325 (CYP4F2) + 3.97 (male) + 7.89 (CYP2C9*2) − 3.49 (VKORC1 -1639G>A) − 8.37 (vit K) − 0.31 (TSH) | - | - | - | - | - | r = 0.52 | High | - | - |
| 139 | Saleh 2014^143^ | Ini-pgx | 9 (IWPC sites) | AF, DVT, PE, HV, stroke, other | 2.0–3.0 | 3415 | 63 | 19 | 10 | 9 | 58 | Age categorized | Age, race, height, weight, amio, inducers | CYP2C9*2, *3; VKORC1-1639G>A | No explicit equation | 48 |  |  |  | 1.2 | IVC (n=856) R2 = 43%, MAE = 1.29 ideal dose = 48% | High | - | - |
| 140 | Shaw 2014^144^ (***children***) | Ini-pgx | Canada | FP, HV, DVT, PE, other | Multiple | 77 | 66 | 17 | - | 17 | 56 (of 93) | 5 (range <1–18) (for 93) | Weight, indication (FP) | CYP2C9 *2, *3; VKORC1 -1639G>A | √dose (mg/d) = 1.711 + 0.014 (weight) – 0.257 (VKORC1) – 0.127 (CYP2C9*2) − 0.463 (CYP2C9*3) – 0.161 (FP) | 76 | 55 | 12 | 9 | - | 92% within 1 mg/d | High | 1 | - |
| 141 | Tatarunas 2014^145^ | Ini-pgx | Lithuania | HV | 2.0–3.5 | 189 | 100 | - | - | - | 62 | 68 (range 27–87) | Age, weight, target INR, TSH, amio, CCB, benzodiazepine | CYP2C9 *2, *3; VKORC1 -1639G>A, 3730G>A | Dose (mg/d) = 5.801 –0.051 (age) + 0.033 (weight) – 0.410 (INR) – 0.627 (TSH) – 0.971 (amio) – 1.268 (benzodiazepines) – 1.368 (CCB) – 0.562 (CYP2C9*2) –1.502 (CYP2C9*3) – 0.991 (VKORC1 -1639G>A) + 0.414 (VKORC1 3730G>A) | 50 | 26 | - | - | - | - | High | - | - |
| 142 | Vear 2014^146^  (***children***) | Ini-pgx | USA | DVT, HV, stroke, TIA, other | 1.5–3.5 | 100 | 88 | - | 8 | 4 | 46 | 12 (range 1–20) | Age | CYP2C9 *2, *3; VKORC1 -1639G>A | Log dose (mg/d) = 1.098 + 0.027 (age) – 1.124 (VKORC1 AA) − 0.733 (VKORC1 GA) + 0.345 (CYP2C9*1*1) + 0.031 (age × VKORC1 AA) + 0.037 (age × VKORC1 GA) | 53 | 31 | 13 | 6 | - | - | High | 1 | - |
| 143 | Ye 2014^147^ | Ini-pgx | China | AF, HV, PE, DVT, stroke | 1.8–3.0 | 101 | - | 100 | - | - | 61 | 63 (range 18–84) | Age, weight | VKORC1-1639G>A | Dose = -0.883 + 2.538 (VKORC1 -1639G>A + 0.023 (weight) – 0.015 (age) | 70 | - | - | - | - | - | High | - | - |

**Table S5. Continued**

| **#** | **Algorithm** | **Sub-type** | **Country** | **Indication** | **Target INR** | **N** | **Ethnicity (%)^a^** | | | | **Male %** | **Age, yrs (mean ± SD or median, IQR)** | **Clinical parameters** | **Genetic parameters** | **Equation^b^** | **R2^c^** | | | | **MAE (mg/d)** | **Other** | **ROB** | **# EV** | **# CUA** |
| --- | --- | --- | --- | --- | --- | --- | --- | --- | --- | --- | --- | --- | --- | --- | --- | --- | --- | --- | --- | --- | --- | --- | --- | --- |
|  |  |  |  |  |  |  | **W** | **A** | **B** | **M** |  |  |  |  |  | **All** | **Cl** | **VKORC1** | **CYP2C9** |  |  |  |  |  |
| 144 | An 2015^148^ | Ini-pgx | Korea | HV | 2.0–3.0 | 191 | - | 100 | - | - | 33 | 59 ± 10 | Age | CYP2C9*3, VKORC1 1173 C>T; CYP4F2*3; UGT1A1 rs887829 | Dose (mg/d) = 9.141 – 2.316 (VKORC1 per T allele) – 1.849 (CYP2C9 per *3 allele) + 0.910 (UGT1A1) – 0.035 (age) + 0.800 (CYP4F2*3*3) | 45 | 3 | 28 | 7 | - |  | High | - | - |
| 145 | Chung 2015^149^ | Ini-pgx | Korea | HV | 2.0–3.0 | 206 | - | 100 | - | - | 33 | 61 ± 10 | Age | CYP2C9*3; VKORC1 1173 C>T, CYP4F2*3, NQO1 rs10157 | Dose (mg/d) = 11.641 − 2.229 (VKORC1 per T allele) − 1.748 (CYP2C9 per *3 allele) − 0.039 (age) + 0.881 (CYP4F2 *3*3) + 0.423 (NQO1 CT/TT) | 44 | 5 | 29 | 7 | - | - | High | - | - |
| 146 | Drozda 2015^150^ (updates Gage 2008) | Ini-pgx | USA | AF, DVT, PE, stroke, HV, other | NR | 274 | - | - | 100 | - | 28 | 55 ± 16 | Age, BSA, amio, target INR, smoking, race, DVT, PE | CYP2C9*2, *3, *5, *6, *8, *11; VKORC1 -1639 G>A; rs12777823 | No explicit equation | - | - | - | - | - | - | High | - | - |
| 147 | Ghozlan 2015^151^ | Ini-pgx | Egypt | Acute coronary syndrome | 2.0–3.0 | 80 | - | - | - | 100 | 70 | 54 ± 8 | Age, height | CYP2C9*2, *3; VKORC1 -1639G>A | Dose (mg/d) = 3.889 + 1.657 (VKORC1) + 0.922 (CYP2C9) + 0.079 (age) + 0.061 (height) | 31 | 13 | 13 | 5 | - | - | High | - | - |
| 148 | Ichihara 2015^152^ | Ini-pgx | Japan | AF, HV, DVT, PE, other | 1.5–3.0 | 137 | - | 100 | - | - | 77 | 70 ± 9 | Age, weight, eCrCl, target INR | VKORC1 1639G>A | Dose (mg/d) = -0.652 − 0.026 (age) + 0.044 (weight) + 1.861 (VKORC1 GG/GA) + 0.016 (eCrCl) + 0.747 (target INR) | 45 | 23 | 23 | - | - | - | High | - | - |
| 149 |  | Ini-pgx | Japan | AF, HV, DVT, PE, other | 1.5–3.0 | 137 | - | 100 | - | - | 77 | 70 ± 9 | Age, weight, eGFR, target INR | VKORC1 1639G>A | Dose (mg/d) = 0.108 − 0.029 (age) + 0.044 (weight) + 1.829 (VKORC1 GG/GA) + 0.016 (eGFR) + 0.589 (target INR) | 43 | 20 | 23 | - | - | - | High | - | - |
| 150 | Jeong 2015^153^ | Ini-pgx | Korea | HV | 2.0–3.0 | 201 | - | 100 | - | - | 33 | Same cohort as Chung 2015 | Age | CYP2C9*3; VKORC1 1173 C>T; CYP4F2*3; GATA4 rs8678 58/ rs10090884, rs2645400/ rs4841588 | Dose (mg/d) = 7.620 − 8.681 (VKORC1 per T allele) − 2.803 (CYP2C9 per *3 allele − 4.186 (age) + 3.248 (GATA4 rs867858 /rs10090884, 0=GG/AA, 1=others) + 2.798 (CYP4F2*3*3) + 2.060 (GATA4 rs2645 400/rs4841588, 0=GG/GT, TT, 1=others) | 41 | 4 | 25 | 4 | - | - | High | - | - |
| 151 | Karaca 2015^154^ | Ini-pgx | Turkey | AF, HV, CVA, DVT, PE, other | NA | 97 | - | - | - | 100 | 49 | 61 ± 13 | Age, gender, concomitant drugs, HV | VKORC1 -1639G>A | Ln dose (mg/d) = 2.292 + 0.1504 (sex) − 0.09932 (age) + 0.3679 (HV) − 0.3227 (VKORC1) + 0.1397 (concomitant drugs) | 46 | - | 29 | - | 1.21 | 48% with ideal dose | High | 1 | - |
| 152 | Lee 2015^155^ | Ini-pgx | Korea | HV | 2.0–3.0 | 201 | - | 100 | - | - | 33 | Same cohort as Chung 2015 | Age, diuretics | CYP2C9*3, VKORC1 1173 C>T; CYP4F2 *3; MYC rs4645974, rs4645943 | Dose (mg/d) = 9.562 − 2.035 (VKORC1 per T allele) − 1.431 (CYP2C9 per *3 allele) − 0.696 (age ≥59) + 0.771 (CYP4F2 *3*3) − 0.954 (MYC rs4645974 CT/TT) + 0.668 (MYC rs4645943 CT/TT) − 0.460 (diuretics) | 44 | 4 | 27 | 5 | - | - | High | - | - |
| 153 | Li 2015^18^ | Ini-pgx | China | HV | 1.7–3.0 | 1036 | - | 100 | - | - | - | Age categorized | Age, height, weight, gender, stroke, HV, smoking, INR-increasing drugs | CYP2C9*3; VKORC1 -1639G>A | No explicit equation (linear regression) | - | - | - | - | - | IVC (n=259): MAE = 0.63, 58% with ideal dose | High | 1 | - |

**Table S5. Continued**

| **#** | **Algorithm** | **Sub-type** | **Country** | **Indication** | **Target INR** | **N** | **Ethnicity (%)^a^** | | | | **Male %** | **Age, yrs (mean ± SD or median, IQR)** | **Clinical parameters** | **Genetic parameters** | **Equation^b^** | **R2^c^** | | | | **MAE (mg/d)** | **Other** | **ROB** | **# EV** | **# CUA** |
| --- | --- | --- | --- | --- | --- | --- | --- | --- | --- | --- | --- | --- | --- | --- | --- | --- | --- | --- | --- | --- | --- | --- | --- | --- |
|  |  |  |  |  |  |  | **W** | **A** | **B** | **M** |  |  |  |  |  | **All** | **Cl** | **VKORC1** | **CYP2C9** |  |  |  |  |  |
| 154 | Li 2015^18^ | Ini-pgx | China | HV | 1.7–3.0 | 1036 | - | 100 | - | - | - | Age categorized | Age, height, weight, gender, stroke, HV, smoking, INR-increasing drugs | CYP2C9*3; VKORC1 -1639G>A | No explicit equation (support vector regression) | - | - | - | - | - | IVC (n=259): MAE = 0.65, 56% with ideal dose | High | 1 | - |
| 155 |  | Ini-pgx | China | HV | 1.7–3.0 | 1036 | - | 100 | - | - | - | Age categorized |  | CYP2C9*3; VKORC1 -1639G>A | No explicit equation (artificial neural network) | - | - | - | - | - | IVC (n=259): MAE = 0.67, 53% with ideal dose | High | 1 | - |
| 156 |  | Ini-pgx | China | HV | 1.7–3.0 | 1036 | - | 100 | - | - | - | Age categorized |  | CYP2C9*3; VKORC1 -1639G>A | No explicit equation (regression tree) | - | - | - | - | - | IVC (n=259): MAE = 0.68, 54% with ideal dose | High | 1 | - |
| 157 |  | Ini-pgx | China | HV | 1.7–3.0 | 1036 | - | 100 | - | - | - | Age categorized |  | CYP2C9*3; VKORC1 -1639G>A | No explicit equation (random forest regression) | - | - | - | - | - | IVC (n=259): MAE = 0.64, 57% with ideal dose | High | 1 | - |
| 158 |  | Ini-pgx | China | HV | 1.7–3.0 | 1036 | - | 100 | - | - | - | Age categorized |  | CYP2C9*3; VKORC1 -1639G>A | No explicit equation (boosted regression tree) | - | - | - | - | - | IVC (n=259): MAE = 0.65, 56% with ideal dose | High | 1 | - |
| 159 |  | Ini-pgx | China | HV | 1.7–3.0 | 1036 | - | 100 | - | - | - | Age categorized |  | CYP2C9*3; VKORC1 -1639G>A | No explicit equation (multivariate adaptive regression spines) | - | - | - | - | - | IVC (n=259): MAE = 0.63, 58% with ideal dose | High | 1 | - |
| 160 | Li 2015^156^ | Ini-pgx | China | HV | 1.7–3.0 | 1297 | - | 100 | - | - | - | Age categorized | Age, gender, weight, HV, INR increasing drug, stroke, smokin g | CYP2C9*3; VKORC1 -1639G>A | No explicit equation | 31 | - | - | - | 0.64 | 57% with ideal dose | High | - | - |
| 161 |  | Ini-pgx | China | HV | 1.7–3.0 | 1297 | - | 100 | - | - | - | Age categorized |  | CYP2C9*3: VKORC1 -1639G>A interaction | No explicit equation | 31 | - | - | - | 0.64 | 57% with ideal dose | High | - | - |

**Table S5. Continued**

| **#** | **Algorithm** | **Sub-type** | **Country** | **Indication** | **Target INR** | **N** | **Ethnicity (%)^a^** | | | | **Male %** | **Age, yrs (mean ± SD or median, IQR)** | **Clinical parameters** | **Genetic parameters** | **Equation^b^** | **R2^c^** | | | | **MAE (mg/d)** | **Other** | **ROB** | **# EV** | **# CUA** |
| --- | --- | --- | --- | --- | --- | --- | --- | --- | --- | --- | --- | --- | --- | --- | --- | --- | --- | --- | --- | --- | --- | --- | --- | --- |
|  |  |  |  |  |  |  | **W** | **A** | **B** | **M** |  |  |  |  |  | **All** | **Cl** | **VKORC1** | **CYP2C9** |  |  |  |  |  |
| 162 | Li 2015^156^ | Ini-pgx | IWPC White cohort | See IWPC 2009 | 2.0–3.0 | 2155 | 100 | - | - | - | - | Age categorized | Age, height, weight, enzyme inducer, amio | CYP2C9*2*3; VKORC1 -1639G>A | No explicit equation (CYP2C9 classified as: *1/*1 and rest) | 22 | - | - | - | 1.33 | 47% with ideal dose | High | - | - |
| 163 |  | Ini-pgx |  | See IWPC 2009 | 2.0–3.0 | 2155 | 100 | - | - | - | - |  |  |  | CYP2C9 classified as: *1/*1, *1/*2, *1/*3, *2/*2, *2/*3, and *3/*3) | 20 | - | - | - | 1.36 | 46% with ideal dose | High | - | - |
| 164 |  | Ini-pgx |  | See IWPC 2009 | 2.0–3.0 | 2155 | 100 | - | - | - | - |  |  |  | CYP2C9 classified as *1/*1, *2 (ie, *1/*2, *2/*2), and *3 (ie, *1/*3, *2/*3, *3/*3)) | 21 | - | - | - | 1.35 | 47% with ideal dose | High | - | - |
| 165 |  | Ini-pgx |  | See IWPC 2009 | 2.0–3.0 | 2155 | 100 | - | - | - | - |  |  |  | CYP2C9 classified as: *1/*1, *2 (ie,*1/*2, *2/*2, *2/*3), and *3 (ie, *1/*3, *3/*3)) | 21 | - | - | - | 1.36 | 47% with ideal dose | High | - | - |
| 166 |  | Ini-pgx | IWPC White cohort | See IWPC 2009 | 2.0–3.0 | 2155 | 100 | - | - | - | - | Age categorized | Age, height, weight, enzyme inducer, amio | CYP2C9*2*3: VKORC1 -1639 G>A interaction | No explicit equation (CYP2C9 classified as: *1/*1 and rest) | 22 | - | - | - | 1.33 | 47% with ideal dose | High | - | - |
| 167 |  | Ini-pgx |  | See IWPC 2009 | 2.0–3.0 | 2155 | 100 | - | - | - | - |  |  |  | CYP2C9 classified as: *1/*1, *1/*2, *1/*3, *2/*2, *2/*3, and *3/*3) | 21 | - | - | - | 1.36 | 46% with ideal dose | High | - | - |
| 168 |  | Ini-pgx |  | See IWPC 2009 | 2.0–3.0 | 2155 | 100 | - | - | - | - |  |  |  | CYP2C9 classified as *1/*1, *2 (ie, *1/*2, *2/*2), and *3 (ie, *1/*3, *2/*3, *3/*3)) | 22 | - | - | - | 1.34 | 47% with ideal dose | High | - | - |
| 169 |  | Ini-pgx |  | See IWPC 2009 | 2.0–3.0 | 2155 | 100 | - | - | - | - |  |  |  | CYP2C9 classified as: *1/*1, *2 (ie,*1/*2, *2/*2, *2/*3), and *3 (ie, *1/*3, *3/*3)) | 21 | - | - | - | 1.35 | 46% with ideal dose | High | - | - |
| 170 | Limdi 2015^157^ (updates Gage 2008) | Ini-pgx | USA | DVT, PE, stroke/TIA, AF, MI, peripheral arterial disease, other | 2.0–3.0 | 1357 | 56 | - | 44 | - | 51 | 61 ± 16 | Age, BSA, race, smoking, DVT/PE, amio | CYP2C9*2, *3; VKORC1 -1639G>A | Ln dose (mg/d) = 1.4564 − 0.0992 (Black) − 0.0068 (age) + 0.4219 (BSA) ­− 0.0022 (current smoker) + 0.0512 (DVT/PE) − 0.2245 (amio) − 0.1929 (CYP2C9*2) − 0.4183 (CYP2C9*3) − 0.3016 (VKORC1) | 46 | 16 | 22 | | - | - | High | - | - |
| 171 | Limdi 2015^157^ | Ini-pgx | USA |  | 2.0–3.0 | 1357 | 56 | - | 44 | - | 51 | 61 ± 16 | Age, BSA, race, amio, CKD (based on eGFR) | CYP2C9*2, *3, *5, *6, *11; VKORC1 -1639G>A; rs12777823; CYP4F2*3 | Ln dose (mg/d) = 1.5456 − 0.0686 (Black) − 0.0069 (age) + 0.4127 (BSA) − 0.0860 (CKDII) − 0.2230 (amio) − 0.2042 (CYP2C9 *2) − 0.4282 (CYP2C9*3) − 0.3098 (VKORC1) + 0.0448 (CYP4F2) − 0.0789 (rs12777823) − 0.1796 (CYP2C9*5/6/11) | 48 | 17 | 24 | | - | - | High | - | - |
| 172 | Limdi 2015^157^ (updates Gage 2008) | Ini-pgx | USA | DVT, PE, stroke/TIA, AF, MI, peripheral arterial disease, other | 2.0–3.0 | 762 | 100 | - | - | - | 58 | 64 ± 15 | Age, BSA, smoking, DVT/PE, amio | CYP2C9*2, *3; VKORC1 -1639G>A; rs12777823 | Ln dose (mg/d) = 1.4749 − 0.0064 (age) + 0.4143 (BSA) − 0.0046 (current smoker) + 0.0574 (DVT/PE) − 0.1906 (amio) − 0.2313 (CYP2C9*2) − 0.4221 (CYP2C9*3) − 0.3330 (VKORC1) | 51 | 15 | 34 | | - | - | High | 1 | - |
| 173 | Limdi 2015^157^ | Ini-pgx | USA | DVT, PE, stroke/TIA, AF, MI, peripheral arterial disease, other | 2.0–3.0 | 762 | 100 | - | - | - | 58 | 64 ± 15 | Age, BSA, race, amio, CKD | CYP2C9*2, *3; VKORC1 -1639G>A; rs12777823; CYP4F2*3 | Ln dose (mg/d) = 1.5413 − 0.0062 (age) + 0.4006 (BSA) − 0.1156 (CKDII) − 0.1926 (amio) − 0.2312 (CYP2C9*2) − 0.4162 (CYP2C9*3) − 0.3416 (VKORC1) + 0.0573 (CYP4F2) − 0.0229 (rs12777823) | 54 | 16 | 35 | | - | - | High | - | - |
| 174 |  | Ini-pgx | USA | DVT, PE, stroke/TIA, AF, MI, peripheral arterial disease, other | 2.0–3.0 | 595 | - | - | 100 | - | 44 | 57 ± 16 | Age, BSA, smoking, DVT/PE, amio | CYP2C9*2, *3; VKORC1 -1639G>A | Ln dose (mg/d) = 1.3163 − 0.0070 (age) + 0.4411 (BSA) − 0.0078 (current smoker) + 0.0374 (DVT/PE) − 0.3146 (amio) − 0.0389 (CYP2C9*2) − 0.4246 (CYP2C9*3) − 0.2058 (VKORC1) | 29 | 22 | 7 | | - | - | High | 1 | - |

**Table S5. Continued**

| **#** | **Algorithm** | **Sub-type** | **Country** | **Indication** | **Target INR** | **N** | **Ethnicity (%)^a^** | | | | **Male %** | **Age, yrs (mean ± SD or median, IQR)** | **Clinical parameters** | **Genetic parameters** | **Equation^b^** | **R2^c^** | | | | **MAE (mg/d)** | **Other** | **ROB** | **# EV** | **# CUA** |
| --- | --- | --- | --- | --- | --- | --- | --- | --- | --- | --- | --- | --- | --- | --- | --- | --- | --- | --- | --- | --- | --- | --- | --- | --- |
|  |  |  |  |  |  |  | **W** | **A** | **B** | **M** |  |  |  |  |  | **All** | **Cl** | **VKORC1** | **CYP2C9** |  |  |  |  |  |
| 175 | Limdi 2015^157^ | Ini-pgx | USA | DVT, PE, stroke/TIA, AF, MI, peripheral arterial disease, other | 2.0–3.0 | 595 | - | - | 100 | - | 44 | 57 ± 16 | Age, BSA, race, amio, CKD | CYP2C9*2, *3, *5, *6, *11; VKORC1 -1639 A; rs12777823; CYP4F2*3 | Ln dose (mg/d) = 1.4355 − 0.0071 (age) + 0.4375 (BSA) − 0.0668 (CKDII) − 0.2977 (amio) − 0.0294 (CYP2C9*2) − 0.4753 (CYP2C9*3) − 0.2231 (VKORC1) + 0.0122 (CYP4F2) − 0.1307 (rs12777823) − 0.1304 (CYP2C9*5/6/11) | 34 | 23 | 10 | - | - | High | - | - |  |
| 176 | Liu 2015^20^ | Ini-pgx | IWPC sites | AF, DVT, PE, stroke, other | 2.0–3.0 | 3838 | 57 | 24 | 14 | 5 | 58 (of 4798) | Age categorized | Age, race, weight, height, smoking, amio, enzyme inducer (minus race and height in Black/ White-only cohorts and minus race, height and enzyme inducer in Asian-only cohort) | CYP2C9*2, *3; VKORC1-1639A | No explicit equation (linear regression) | - | - | - | - | - | IVC (n=960) (MAE/ideal dose) = 1.33/44% (1.43/43% for Whites [n = 544],  0.88/46% for Asians [n = 231], 1.74/43% for Blacks [n = 133]) | High | - | - |
| 177 |  | Ini-pgx | IWPC sites | AF, DVT, PE, stroke, other | 2.0–3.0 | 3838 | 57 | 24 | 14 | 5 | 58 (of 4798) | Age categorized |  | CYP2C9*2, *3; VKORC1-1639A | No explicit equation (support vector regression) | - | - | - | - | - | IVC (n=960) (MAE/ideal dose) = 1.28/46% (1.37/45% for Whites [n = 544],  0.88/46% for Asians [n = 231], 1.77/42% for Blacks [n = 133]) | High | - | - |
| 178 |  | Ini-pgx | IWPC sites | AF, DVT, PE, stroke, other | 2.0–3.0 | 3838 | 57 | 24 | 14 | 5 | 58 (of 4798) | Age categorized |  | CYP2C9*2, *3; VKORC1-1639A | No explicit equation (artificial neural network) | - | - | - | - | - | IVC (n=960) (MAE/ideal dose) = 1.40/41% (1.51/40% for Whites [n = 544],  0.93/43% for Asians [n = 231], 1.89/ 40% for Blacks [n = 133]) | High | - | - |
| 179 |  | Ini-pgx | IWPC sites | AF, DVT, PE, stroke, other | 2.0–3.0 | 3838 | 57 | 24 | 14 | 5 | 58 (of 4798) | Age categorized |  | CYP2C9*2, *3; VKORC1-1639A | No explicit equation (regression tree) | - | - | - | - | - | IVC (n=960) (MAE/ideal dose) = 1.37/ 43% (1.51/41% for Whites [n = 544],0.94/42% for Asians [n = 231], 1.98/ 37% for Blacks [n = 133]) | High | - | - |

**Table S5. Continued**

| **#** | **Algorithm** | **Sub-type** | **Country** | **Indication** | **Target INR** | **N** | **Ethnicity (%)^a^** | | | | **Male %** | **Age, yrs (mean ± SD or median, IQR)** | **Clinical parameters** | **Genetic parameters** | **Equation^b^** | **R2^c^** | | | | **MAE (mg/d)** | **Other** | **ROB** | **# EV** | **# CUA** |
| --- | --- | --- | --- | --- | --- | --- | --- | --- | --- | --- | --- | --- | --- | --- | --- | --- | --- | --- | --- | --- | --- | --- | --- | --- |
|  |  |  |  |  |  |  | **W** | **A** | **B** | **M** |  |  |  |  |  | **All** | **Cl** | **VKORC1** | **CYP2C9** |  |  |  |  |  |
| 180 | Liu 2015^20^ | Ini-pgx | IWPC sites | AF, DVT, PE, stroke, other | 2.0–3.0 | 3838 | 57 | 24 | 14 | 5 | 58 (of 4798) | Age categorized | Age, race, weight, height, smoking, amio, enzyme inducer (minus race and height in Black/White-only cohorts and minus race, height and enzyme inducer in Asian-only cohort) | CYP2C9*2, *3; VKORC1-1639A | No explicit equation (random forest regression) | - | - | - | - | - | IVC (n=960) (MAE/ideal dose) = 1.29/45% (1.39/44% for Whites [n = 544],  0.89/45% for Asians [n = 231], 1.81/42% for Blacks [n = 133]) | High | - | - |
| 181 |  | Ini-pgx | IWPC sites | AF, DVT, PE, stroke, other | 2.0–3.0 | 3838 | 57 | 24 | 14 | 5 | 58 (of 4798) | Age categorized |  | CYP2C9*2, *3; VKORC1-1639A | No explicit equation (boosted regression tree) | - | - | - | - | - | IVC (n=960) (MAE/ideal dose) = 1.28/46% (1.37/45% for Whites [n = 544],  0.89/45% for Asians [n = 231], 1.79/42% for Blacks [n = 133]) | High | - | - |
| 182 |  | Ini-pgx | IWPC sites | AF, DVT, PE, stroke, other | 2.0–3.0 | 3838 | 57 | 24 | 14 | 5 | 58 (of 4798) | Age categorized |  | CYP2C9*2, *3; VKORC1-1639A | No explicit equation (multivariate adaptive regression splines) | - | - | - | - | - | IVC (n=960) (MAE/ideal dose) = 1.26/46% (1.34/46% for Whites [n = 544],  0.87/47% for Asians [n = 231], 1.76/42% for Blacks [n = 133]) | High | - | - |
| 183 |  | Ini-pgx | IWPC sites | AF, DVT, PE, stroke, other | 2.0–3.0 | 3838 | 57 | 24 | 14 | 5 | 58 (of 4798) | Age categorized |  | CYP2C9*2, *3; VKORC1-1639A | No explicit equation (Lasso regression) | - | - | - | - | - | IVC (n=960) (MAE/ideal dose) = 1.33/ 44% (1.43/ 43% for Whites [n = 544], 0.88/46% for Asians [n = 231], 1.74/ 43% for Blacks [n = 133]) | High | - | - |

**Table S5. Continued**

| **#** | **Algorithm** | **Sub-type** | **Country** | **Indication** | **Target INR** | **N** | **Ethnicity (%)^a^** | | | | **Male %** | **Age, yrs (mean ± SD or median, IQR)** | **Clinical parameters** | **Genetic parameters** | **Equation^b^** | **R2^c^** | | | | **MAE (mg/d)** | **Other** | **ROB** | **# EV** | **# CUA** |
| --- | --- | --- | --- | --- | --- | --- | --- | --- | --- | --- | --- | --- | --- | --- | --- | --- | --- | --- | --- | --- | --- | --- | --- | --- |
|  |  |  |  |  |  |  | **W** | **A** | **B** | **M** |  |  |  |  |  | **All** | **Cl** | **VKORC1** | **CYP2C9** |  |  |  |  |  |
| 184 | Liu 2015^20^ | Ini-pgx | IWPC sites | AF, DVT, PE, stroke, other | 2.0–3.0 | 3838 | 57 | 24 | 14 | 5 | 58 (of 4798) | Age in decades | Age, race, weight, height, smoking, amio, enzyme inducer (minus race and height in Black/White-only cohorts and minus race, height and enzyme inducer in Asian-only cohort) | CYP2C9*2, *3; VKORC1-1639A | No explicit equation (Bayesian additive regression trees) | - | - | - | - | - | IVC (n=960) (MAE/ideal dose) = 1.27/46% (1.35/46% for Whites [n = 544],  0.87/47% for Asians [n = 231], 1.76/42% for Blacks [n = 133]) | High | - | - |
| 185 | Moon 2015^158^ | Ini-pgx | Korea | HV | 2.0–3.0 | 201 | - | 100 | - | - | Same cohort as Chung 2015 | | Age | CYP2C9*3, VKORC1 1173C>T; CYP4F2*3; PXR rs2472682; CAR/HNF4a rs2501873/rs3212198 | Dose (mg/d) = 15.924 − 9.590 (VKORC1 per T allele) − 4.140 (CYP2C9 per *3 allele) − 3.740 (age ≥59) + 2.935 (CYP4F2 *3*3) – 2.138 (PXR rs2472682 CC) − 2.024 (CAR/HNF4a rs2501873/ rs3212198, 0 = TT/CT, TT, 1 = others) | 44 | 4 | 30 | 6 | - | - | High | - | - |
| 186 | Oztaner 2015^159^ | Ini-pgx | IWPC sites | PE, DVT, other | 2.0–3.5 | 2982 | - | - | - | - |  | Age in decades | Age, BSA, PE, DVT, amio, target INR, smoking | CYP2C9*2, *3; VKORC1−1639G>A | No explicit equation | 54 | - | - | - | - | - | High | - | - |
| 187 |  | Ini-pgx | Turkey | PE/DVT, other | 2.0–3.0 | 72 | - | - | - | 100 |  | Age in decades | Age, BSA, indications (DVT/PE/cardiac) | CYP2C9*2, *3; VKORC1−1639G>A; CYP4F2*3 | No explicit equation | 57 | 5 | 27 | 24 | - | - | High | - | - |
| 188 | Santos 2015^160^ (updates IWPC 2009) | Ini-pgx | Brazil | AF, CVA, TE, HV, other | 1.8–3.2 | 368 | 69 | - | 8 | 24 | 50 (of 832) | 63 ± 14 (for 832) | Age, gender, race, weight, height, amio, enzyme inducers | CYP2C9*2, *3; VKORC1-1639A | No explicit equation (no intercept) | 51 | - | - | - | - | Internally validated R2 = 40 | High | 1 | - |
| 189 | Alzubiedi 2016^161^ | Ini-pgx | USA (IWPC sites) | DVT, PE, AF, stroke, other | 2.0–3.0 | 163 | - | - | 100 | - | 39 | Age in decades | Age, weight, CHF, amio | CYP2C9*2, *3, *5; VKORC1 -1639G>A, CYP4F2*3, rs12777823 | √dose (mg/wk) = 5.97+ 0.02 (weight) − 0.25 (age) − 0.49 (rs12777823) − 0.74 (VKORC1) − 0.92 (amio) + 0.62 (CYP4F2) + 0.69 (CYP2C9*1*1) − 0.51 (CHF) | 38 | - | - | - | 1.54 | 52% patients with ideal dose | High | 1 | - |
| 190 |  | Ini-pgx | USA (IWPC sites) | DVT, PE, AF, stroke, other | 2.0–3.0 | 163 | - | - | 100 | - | 39 | Age in decades | Age, weight, CHF, amio | CYP2C9*2, *3, *5; VKORC1 -1639A, CYP4F2*3, CYP2C rs12777823 | No explicit equation (artificial neural network) | 38 |  |  |  | 1.56 | 48% patients with ideal dose | High | - | - |

**Table S5. Continued**

| **#** | **Algorithm** | **Sub-type** | **Country** | **Indication** | **Target INR** | **N** | **Ethnicity (%)^a^** | | | | **Male %** | **Age, yrs (mean ± SD or median, IQR)** | **Clinical parameters** | **Genetic parameters** | **Equation^b^** | **R2^c^** | | | | **MAE (mg/d)** | **Other** | **ROB** | **# EV** | **# CUA** |
| --- | --- | --- | --- | --- | --- | --- | --- | --- | --- | --- | --- | --- | --- | --- | --- | --- | --- | --- | --- | --- | --- | --- | --- | --- |
|  |  |  |  |  |  |  | **W** | **A** | **B** | **M** |  |  |  |  |  | **All** | **Cl** | **VKORC1** | **CYP2C9** |  |  |  |  |  |
| 191 | Cho 2016^162^ | Ini-pgx | Korea | Stroke | 1.5–3.0 | 101 | - | 100 | - | - | 63 | 64 ± 13 | Age, weight | CYP2C9*3, VKORC1 1173C>T | Ln dose (mg/d) = 1.756 – 0.015 (age) + 0.006 (weight) – 0.284 (CYP2C9) + 0.407 (VKORC1) | 51 | 33 | 17 | 1 | - | Ideal dose = 53%; M%PE = 3% | High | 1 | - |
| 192 | Eriksson 2016^163^ (updates IWPC 2009) | Ini-pgx | 44 (RE­LY sub-study) | AF | 2.0–3.0 | 951 | 87 | 2 | 1 | 10 | 66 (of 956) | 72 (68–77) (for 956) | Age, height, weight, race, enzyme inducers, amio | CYP2C9*2, *3; VKORC1-1639A; CYP4F2*3; DDHD1 rs17126068; NEDD4 rs2288344 | IWPC 2009 + 0.178 (CYP4F2*3) – 0.809 (DDHD1) + 0.191 (NEDD4) | 56 | - | - | - | - | 58% with ideal dose | High | - | - |
| 193 | Jiang 2016^164^ | Ini-pgx | China | AF | 1.8–3.0 | 122 | - | 100 | - | - | 62 | median 59 | Age, height | CYP2C9*3; VKORC1 -1639A | Dose (mg/d) = 0.438 – 0.0143 (age) + 0.0149 (height) – 0.324 (CYP2C9*3) + 0.209 (VKORC1) | 56 | 30 | 17 | 19 | - | - | High | 1 | 1 |
| 194 | Li 2016 (from Xie 2020^141^) | Ini-pgx | China | HV, AF, PE | 1.8–3.0 | 384 | 0 | 100 | 0 | 0 | - | - | Age, weight, height, amio | VKORC1, CYP2C9 | No explicit equation | 68 | - | - | - | - | - | Unclear | 1 | - |
| 195 | Liu 2016^165^ | Ini-pgx | China | HV | 1.6–2.5 | 186 | - | 100 | - | - | 50 | 56 ± 10 | Age, BSA, INR-increasing drugs | APOE ε2, ε3, ε4 | √dose (mg/d) = 1.515 – 0.006 (age) + 0.325 (BSA) + 0.113 (ε2/ε3) – 0.018 (ε3/ε4) – 0.130 (# INR-increasing drugs) | 31 | - | - | - | - | - | High | - | - |
| 196 | Pavani 2016^166^ | Ini-pgx | India | HV, other | 2.0–3.5 | 157 | - | 100 | - | - | 54 | 38 ± 15 | Age, gender, BMI, plasma vitamin K levels, thyroid status | CYP2C9*2, *3, *8, *13; VKORC1 3730 G>A, 6009C>T, -1639G>A, D36Y; CYP4F2 *3, GGCX 8016G>A | No explicit equation (artificial neural network) | 94/  96 | - | - | - | - | MPE = -0.28 | High | - | 1 |
| 197 | Rouleau-Mailloux 2016^167^ | Ini-pgx | Canada | Mitral stenosis, AF, HV, other | 2.0–3.5 | 969 | 97 | <1 | 1 | 2 | 61 | 71 (range 18–95) | Age, height, weight, target INR, RPA | CYP2C9 *2, *3; VKORC1 -1639A | Ln dose (mg/wk) = 2.735 + 0.053 (RPA) – 0.215 (CYP2C9*2) – 0.427 (CYP2C9*3) – 0.315 (VKORC1) + 0.129 (target INR) – 0.008 (age) + 0.518 (height) + 0.003 (weight) | 52 | 15 | 36 | | - | - | High | - | - |
| 198 |  | Ini-pgx | Canada | - | 2.0–3.5 | 618 | >99 | - | - | <1 | 65 | 69 (range 27–90) | Age, height, weight, target INR, RPA | CYP2C9*2, *3 | No explicit equation | 34 | - | - | - | - | - | High | - | - |
| 199 | Shahabi 2016^168^ (based on Gage 2008) | Ini-pgx | USA | AF, DVT, PE, HV, other | 2.0–3.0 | 73 | 93 | - | 4 | 3 | 67 | 66 ± 13 | Age, gender | CYP2C9*2, *3, *5, *6, *8, *11, *14; VKORC1 -1639G>A; CYP4F2*3 | Doses available in the Coriell Personalized Medicine Collaborative Warfarin Dosing table | - | - | - | - | 1.3 | 51% within 1 mg/d | High | - | - |
|  |  |  | Canada | AF, HV, mitral stenosis, other | 2.0–3.5 | 973 | 96 | <1 | 1 | 2 | 61 | 70 ± 12 |  |  |  | - | - | - | - | 1.2 (of 769) | 52% within 1mg/d (of 769) |  |  |  |
| 200 | Wakamiya 2016^169^ (***children***) | Ini-pgx | Japan | HV, FP, Kawasaki disease, other | 1.5–2.5 | 45 | - | 100 | - | - | 84 | 8 (range 0–19) | Height | VKORC1 -1639A | √dose (mg/d) = 0.235 + 0.011 (height) – 0.3 (VKORC1 TT) | 78 | 51 | 27 | - | - | 89% within 1mg/d | High | - | - |

**Table S5. Continued**

| **#** | **Algorithm** | **Sub-type** | **Country** | **Indication** | **Target INR** | **N** | **Ethnicity (%)^a^** | | | | **Male %** | **Age, yrs (mean ± SD or median, IQR)** | **Clinical parameters** | **Genetic parameters** | **Equation^b^** | **R2^c^** | | | | **MAE (mg/d)** | **Other** | **ROB** | **# EV** | **# CUA** |
| --- | --- | --- | --- | --- | --- | --- | --- | --- | --- | --- | --- | --- | --- | --- | --- | --- | --- | --- | --- | --- | --- | --- | --- | --- |
|  |  |  |  |  |  |  | **W** | **A** | **B** | **M** |  |  |  |  |  | **All** | **Cl** | **VKORC1** | **CYP2C9** |  |  |  |  |  |
| 201 | Zeng 2016^170^ | Ini-pgx | China | AF, DVT, HV, other | 1.8–2.5 | 408 | - | 100 | - | - | 41 | 50 ± 12 | Age, gender, BSA, aspirin | CYP2C9*3; VKORC1 -1639C>T; CYP4F2*3; POR 831–35C>T | √dose (mg/d) = 5.131 − 0.010 (age) + 0.0609 (BSA) − 0.211 (gender) − 0.143 (aspirin) − 1.636 (CYP2C9*3) + 0.667 (CYP4F2) − 2.721 (VKORC1) + 0.312 (POR) | 42 | - | 23 | 17 | - | - | High | - | - |
| 202 |  | Ini-pgx | China | AF, DVT, HV, other | 1.8–2.5 | 408 | - | 100 | - | - | 41 | 50 ± 12 | Age, gender, BSA | CYP2C9*3; VKORC1 -1639C>T; CYP4F2*3; POR 831–35C> | √dose (mg/d) = 4.991 − 0.011 (age) + 0.0613 (BSA) − 0.171 (gender) −1.687 (CYP2C9*3) + 0.625 (CYP4F2*3) − 2.631 (VKORC1) + 0.359 (POR) | 41 | - | - | - | - | - | High | - | - |
| 203 |  | Ini-pgx | China | AF, DVT, HV, other | 1.8–2.5 | 408 | - | 100 | - | - | 41 | 50 ± 12 | Age, gender, BSA | CYP2C9*3; VKORC1 -1639C>T; CYP4F2*3 | √dose (mg/d) = 5.383 − 0.010 (age) + 0.0703 (BSA) − 0.199 (gender) − 1.694 (CYP2C9) + 0.698 (CYP4F2) − 2.749 (VKORC1) | 37 | - | - | - | - | - | High | 1 | - |
| 204 | Claudio-Campos 2017^171^ | Ini-pgx | Puerto Rico | AF, DVT, PE, HV, stroke, other | 2.0–3.0 for most | 115 | 90 | - | 10 | - | >99 (of 255) | 72 ± 10 | Age, DM, HTN | CYP2C9*2, *3, rs1856908; VKORC1 -1639G>A; ABCB1 rs10276036; CES2 rs4783745 | Dose (mg/d) = 7.272 − 1.710 (VKORC1) + 0.723 (rs1856908) + 0.786 (ABCB1) + 0.591 (CES2) − 0.936 (CYP2C9*2) − 0.032 (age) + 0.598 (DM) − 0.638 (HTN) − 0.526 (CYP2C9*3) | 58 | 5 | 31 | 15 | - | - | High | - | - |
| 205 |  | Ini-pgx | Puerto Rico | AF, DVT, PE, HV, stroke, other | 2.0–3.0 for most | 115 | 90 | - | 10 | - | >99 (of 255) | 72 ± 10 | Age, DM, HTN | CYP2C9 rs2860905, rs1856908; VKORC1 -1639G>A; ABCB1 rs10276036; CES2 rs4783745 | Dose (mg/d) = 7.017 – 1.639 (VKORC1) −0.902 (rs2860905) − 0.039 (age) + 0.686 (rs1856908) + 0.710 (ABCB1) + 0.506 (CES2) + 0.648 (DM) − 0.656 (HTN) | 60 | 10 | 31 | 17 | 1.07 | mean standard error = 0.38 mg/d | High | - | - |
| 206 | Jiang 2017^172^ | Ini-pgx | China | AF | IQR 2.2–3.2 | 215 | - | 100 | - | - | 63 (or 37) | 56 (47–66) | Age, height | CYP2C9*3; VKORC1 -1639C>T; GGCX rs699664 | Dose (mg/d) = 1.726 – 0.008 (age) + 0.009 (height) – 0.620 (CYP2C9) + 0.439 (VKORC1) + 0.257 (GGCX) | 41 | 4 | 17 | 7 | - | Likely IVC (n=60), r = 0.660 | High | - | - |
| 207 | Liu 2017^173^ | Ini-pgx | China | HV | 1.6–2.5 | 183 | - | 100 | - | - | 51 | 56 ± 10 | Age, BSA, | CYP2C9*3; VKORC1 -1639G>A, 497T>G; CYP4F2*3; CYP1A2 rs2069514; CYP3A4 rs28371759; APOE rs7412 | √dose (mg/d) = 1.226 + 0.203 (VKORC1 -1639G>A) − 0.234 (CYP2C9*3) + 0.386 (BSA) + 0.110 (CYP1A2) − 0.004 (age) +  0.178 (CYP3A4) + 0.063 (CYP4F2*3)  +  0.091 (APOE) − 0.093 (VKORC1 497T>G) | 44 | 9 | 16 | 10 | - | - | High | 1 | - |

**Table S5. Continued**

| **#** | **Algorithm** | **Sub-type** | **Country** | **Indication** | **Target INR** | **N** | **Ethnicity (%)^a^** | | | | **Male %** | **Age, yrs (mean ± SD or median, IQR)** | **Clinical parameters** | **Genetic parameters** | **Equation^b^** | **R2^c^** | | | | **MAE (mg/d)** | **Other** | **ROB** | **# EV** | **# CUA** |
| --- | --- | --- | --- | --- | --- | --- | --- | --- | --- | --- | --- | --- | --- | --- | --- | --- | --- | --- | --- | --- | --- | --- | --- | --- |
|  |  |  |  |  |  |  | **W** | **A** | **B** | **M** |  |  |  |  |  | **All** | **Cl** | **VKORC1** | **CYP2C9** |  |  |  |  |  |
| 208 | Luo 2017^174^ | Ini-pgx | China | HV | 2.0–3.0 | 420 | - | 100 | - | - | 58 | 48 ± 12 | Age, BSA, gender, HV, HTN, DM, amio | CYP2C9*3, VKORC1 -1639G>A; rs2304429; rs3826041; rs72800847; rs10517; CYP4F2*3 | Dose = 0.38 + 0.3 (female) − 0.01 (age) + 0.72 (HV type) − 0.35 (HTN) + 0.61 (DM) − 0.48 (amio) + 1.14 (BSA) + 0.38 (rs2304429 AA) + 0.69 (CYP2C9 *1*1) + 0.22 (rs3826041 CC) − 0.96 (rs72800847 GA) − 0.76 (rs72800847 AA) + 1.38 (VKORC1 GA) + 1.78 (VKORC1 AA) + 0.26 (rs10517 TT) + 0.29 (CYP4F2*1*3). | 56 | - | - | - | - | - | High | - | - |
| 209 | Sohrabi 2017^175^ | Ini-pgx | Iran | - | - | 533 | - | Y | - | - | 47 | 59 ± 13 | Age, gender, height, weight, bleeding, DM, hyperlipidemia, HTN, smoking, thyroid, HV, other drugs | CYP2C9*2*3; VKORC1 -1639G>A Factor7Msp1, NQO1, exon3EcorV, EPHX4Rsa1 | No explicit equation | - | - | - | - | 0.11 | MSE=0.01, RMSE=0.10 | High | - | - |
| 210 |  | Ini-pgx | Iran | - | - | 533 | - | Y | - | - | 47 | 59 ± 13 | Age, gender, height, weight, bleeding, DM, hyperlipidemia, HTN, smoking, thyroid, HV, other drugs | CYP2C9*2*3; VKORC1 -1639G>A Factor7Msp1, NQO1, exon3EcorV, EPHX4Rsa1 | No explicit equation | - | - | - | - | 0.11 | MSE=0.02, RMSE=0.14 | High | - | - |
| 211 | Tang 2017^176^ | Ini-pgx | China | HV | 1.6–3.3 (based on the targets) | 231 | - | 100 | - | - | 27 | 46 ± 10 | Age, BSA | CYP2C9*3; VKORC1 -1639G>A; CYP4F2*3 | √dose (mg/d) = 2.095 − 0.304 (VKORC1) − 0.369 (CYP2C9*3) − 0.005 (age) + 0.295 (BSA) + 0.087 (CYP4F2) | 44 | 10 | 25 | 12 | - | - | High | - | - |
| 212 | Lee 2017^177^ | Ini-pgx | Korea | HV | 2.0–3.0 | 204 | - | 100 | - | - | 32 | 58 ± 10 | Age, weight | CYP2C9*3; VKORC1 1173C>T; CYP4F2*3; VDR rs7975232/rs2228571 | Dose (mg/d) = 10.424 − 2.334 (VKORC1) − 1.502 (CYP2C9*3) − 0.030 (age) − 0.567 (VDR, 0 = GT,TT/CT,CC, 1 = others) + 0.799 (CYP4F2*3*3) + 0.022 (weight) | 48 | 4 | 35 | 5 | - | 42% with ideal dose | High | - | - |
| 213 |  | Ini-pgx | Korea | HV | 2.0–3.0 | 204 | - | 100 | - | - | 32 | 58 ± 10 | Age, weight | CYP2C9*3; VKORC1 1173C>T; CYP4F2*3 | No explicit equation | 46 | - | - | - | - | 40% with ideal dose | High | - | - |
| 214 | Wattanachai 2017^178^ | Ini-pgx | Thailand | AF, DVT, PE, HV, other | 2.0–3.0 | 250 | - | 100 | - | - | 52 | 61 ± 13 | Age, BMI, smoking, amio, antiplatelet drugs | CYP2C9*3; VKORC1 −1639G>A; CYP4F2*3 | Dose (mg/wk) = 45.110 −18.109 (VKORC1 AA) − 0.250 (age) − 9.745 (VKORC1 GA) − 25.921 (CYP2C9*3*3) − 7.245 (amio) − 7.919 (CYP2C9*1*3) + 0.345 (BMI) + 4.149 (CYP4F2*3*3) − 3.359 (antiplatelet drugs use) + 3.968 (current smoker). | 51 | 18 | 26 | 6 | - | - | High | - | - |

**Table S5. Continued**

| **#** | **Algorithm** | **Sub-type** | **Country** | **Indication** | **Target INR** | **N** | **Ethnicity (%)^a^** | | | | **Male %** | **Age, yrs (mean ± SD or median, IQR)** | **Clinical parameters** | **Genetic parameters** | **Equation^b^** | **R2^c^** | | | | **MAE (mg/d)** | **Other** | **ROB** | **# EV** | **# CUA** |
| --- | --- | --- | --- | --- | --- | --- | --- | --- | --- | --- | --- | --- | --- | --- | --- | --- | --- | --- | --- | --- | --- | --- | --- | --- |
|  |  |  |  |  |  |  | **W** | **A** | **B** | **M** |  |  |  |  |  | **All** | **Cl** | **VKORC1** | **CYP2C9** |  |  |  |  |  |
| 215 | Wiley 2017^179^ | Ini-pgx | USA | DVT, PE, AF | 1.9–3.2 | 2181 | 88 | - | 12 | - | 58 | 66 ± 15 | Age, BSA, smoking, amio, enzyme inducers | CYP2C9*2, *3, *5, *6, *8, *11, VKORC1 -1639 G>A; rs12777823; other CYP2C9/ VKORC1 SNPs – either alone, combined or as haplotypes | No equation (Limited Genetic, Unadjusted) | 52 | 20 | - | - | 1.33 | - | High | - | - |
| 216 |  | Ini-pgx | USA | DVT, PE, AF | 1.9–3.2 | 2181 | 88 | - | 12 | - | 58 | 66 ± 15 |  |  | Limited Genetic - Race Adjusted | 52 | 22 | - | - | 1.33 | - | High | - | - |
| 217 |  | Ini-pgx | USA | DVT, PE, AF | 1.9–3.2 | 2181 | 88 | - | 12 | - | 58 | 66 ± 15 |  |  | Limited Genetic - % Ancestry Adjusted | 50 | 24 | - | - | 1.36 | - | High | - | - |
| 218 |  | Ini-pgx | USA | DVT, PE, AF | 1.9–3.2 | 2181 | 88 | - | 12 | - | 58 | 66 ± 15 |  |  | Expanded Genetic - Unadjusted | 54 | 20 | - | - | 1.29 | - | High | - | - |
| 219 |  | Ini-pgx | USA | DVT, PE, AF | 1.9–3.2 | 2181 | 88 | - | 12 | - | 58 | 66 ± 15 |  |  | Expanded Genetic - Race Adjusted | 54 | 22 | - | - | 1.29 | - | High | - | - |
| 220 |  | Ini-pgx | USA | DVT, PE, AF | 1.9–3.2 | 2181 | 88 | - | 12 | - | 58 | 66 ± 15 |  |  | Expanded Genetic - % Ancestry Adjusted | 53 | 24 | - | - | 1.31 | - | High | - | - |
| 221 |  | Ini-pgx | USA | DVT, PE, AF | 1.9–3.2 | 2181 | 88 | - | 12 | - | 58 | 66 ± 15 |  |  | Combined SNP - Unadjusted | 44 | 20 | - | - | 1.41 | - | High | - | - |
| 222 |  | Ini-pgx | USA | DVT, PE, AF | 1.9–3.2 | 2181 | 88 | - | 12 | - | 58 | 66 ± 15 |  |  | Combined SNP - Race Adjusted | 44 | 22 | - | - | 1.41 | - | High | - | - |
| 223 |  | Ini-pgx | USA | DVT, PE, AF | 1.9–3.2 | 2181 | 88 | - | 12 | - | 58 | 66 ± 15 |  |  | Combined SNP - % Ancestry Adjusted | 44 | 24 | - | - | 1.43 | - | High | - | - |
| 224 |  | Ini-pgx | USA | DVT, PE, AF | 1.9–3.2 | 2181 | 88 | - | 12 | - | 58 | 66 ± 15 |  |  | Haplotype - Unadjusted | 54 | 20 | - | - | 1.29 | - | High | - | - |
| 225 |  | Ini-pgx | USA | DVT, PE, AF | 1.9–3.2 | 2181 | 88 | - | 12 | - | 58 | 66 ± 15 |  |  | Haplotype - Race Adjusted | 54 | 22 | - | - | 1.29 | - | High | - | - |
| 226 |  | Ini-pgx | USA | DVT, PE, AF | 1.9–3.2 | 2181 | 88 | - | 12 | - | 58 | 66 ± 15 |  |  | Haplotype - % Ancestry Adjusted | 53 | 24 | - | - | 1.31 | - | High | - | - |
| 227 |  | Ini-pgx | USA | DVT, PE, AF | 1.9–3.2 | 1928 | 100 | - | - | - | 59 | 66 ± 15 |  |  | Limited Genetic - Whites only | 54 | 20 | - | - | 1.26 | - | High | - | - |
| 228 |  | Ini-pgx | USA | DVT, PE, AF | 1.9–3.2 | 1928 | 100 | - | - | - | 59 | 66 ± 15 |  |  | Expanded Genetic - Whites only | 56 | 20 | - | - | 1.23 | - | High | - | - |
| 229 |  | Ini-pgx | USA | DVT, PE, AF | 1.9–3.2 | 1928 | 100 | - | - | - | 59 | 66 ± 15 |  |  | Combined SNP - Whites only | 45 | 20 | - | - | 1.37 | - | High | - | - |
| 230 |  | Ini-pgx | USA | DVT, PE, AF | 1.9–3.2 | 1928 | 100 | - | - | - | 59 | 66 ± 15 |  |  | Haplotype - Whites only | 56 | 20 | - | - | 1.23 | - | High | - | - |
| 231 |  | Ini-pgx | USA | DVT, PE, AF | 1.9–3.2 | 253 | - | - | 100 | - | 50 | 60 ± 16 |  |  | Limited Genetic - Blacks only | 31 | 22 | - | - | 1.81 | - | High | - | - |
| 232 |  | Ini-pgx | USA | DVT, PE, AF | 1.9–3.2 | 253 | - | - | 100 | - | 50 | 60 ± 16 |  |  | Expanded Genetic - Blacks only | 40 | 22 | - | - | 1.7 | - | High | - | - |
| 233 |  | Ini-pgx | USA | DVT, PE, AF | 1.9–3.2 | 253 | - | - | 100 | - | 50 | 60 ± 16 |  |  | Combined SNP - Blacks only | 34 | 22 | - | - | 1.79 | - | High | - | - |
| 234 |  | Ini-pgx | USA | DVT, PE, AF | 1.9–3.2 | 253 | - | - | 100 | - | 50 | 60 ± 16 |  |  | Haplotype - Blacks only | 40 | 22 | - | - | 1.71 | - | High | - | - |
| 235 | Zhu 2017^180^ (based on Pop-PK model) | Rev-pgx | China | HV | 1.8–2.5 | 144 | - | Y | - | - | 42 (of 242) | 55 ± 12 (for 242) | Age, apparent S-warfarin clearance, target INR | VKORC1 -1639G>A | Dose = - 0.023 (age) + 1.834 (VKORC1) + 0.952 (target INR) + 2.156 (S-warfarin clearance) [says observed INR in methods) | 67 |  |  |  |  | IVC (n=42) R2 = 72%, MPE = -0.20; RMSE = 0.24 | High | - | - |
| 236 | Chung 2018^181^ | Ini-pgx | Korea | HV | 2.0–3.0 | 201 | - | 100 | - | - | 32 | 58 ± 10 | Age, weight, ACE inhibitors or ARB | CYP2C9*3; VKORC1 1173 C>T; CYP4F2 *3; VKORC1L1 rs4072879; EPHX1 rs1877724 | Dose (mg/d) = 8.509 − 2.050 (VKORC1) − 1.592 (rs1057910) −0.026 (age) + 0.793 (CYP4F2) + 0.025 (weight) − 0.519 (ACE inhibitors or ARB) + 0.467 (VKORC1L1 GA/GG) + 0.446 (EPHX1 CT/TT) | 47 | 6 | 29 | 6 | - | 35% with ideal dose | High | - | - |
| 237 | Gaikwad 2018^182^ | Ini-pgx | India | AF, DVT, HV, other | - | 300 | - | 100 | - | - | 59 | 37 (range 19–80) | Age, diet (vegetarian) | CYP2C9*2, *3; VKORC1 -1639 G>A | √dose (mg/d) = 2.61 − 0.41 (VKORCI) - 0.21 (*1*2) − 0.58 (*1*3) − 0.86 (*2*3) − 0.86 (*3*3) − 0.002 (age) − 0.08 (diet) | 67 | 2 | 23 | 42 | - | - | High | - | - |
| 238 | Galvez 2018^183^ | Ini-pgx | Colombia | HV, DVT, PE, other | 2.0–3.0 | 152 | - | - | - | 100 | 56 | 63 ± 15 | Age, gender | CYP2C9 *2, *3; VKORC1 -1639G>A | √dose (mg/wk) = 9.672 – 0.02 (age) – 0.404 (gender) – 0.794 (VKORC1) – 0.607 (CYP2C9) | 44 | - | 26 | 4 | - | - | High | 1 | - |
| 239 | Kabalak 2018^184^ | Ini-pgx | Turkey | DVT/PE | 2.0–3.0 | 75 | - | - | - | 100 | 52 | 55 ± 16 | Age, COPD | VKORC1 -1639 G>A/1173C>T; FV Leiden-1691 G>A; Factor II -20210 G>A; MTHFR 677C>T | Dose (mg/wk) = 37.4 + 3.6 (FV Leiden) + 0.98 (Factor II) − 4 (VKORC1) + 0.73 (COPD) + 3.1 (MTHFR) – 2 (age) | 17 | - | 6 | - | - | - | High | - | - |

**Table S5. Continued**

| **#** | **Algorithm** | **Sub-type** | **Country** | **Indication** | **Target INR** | **N** | **Ethnicity (%)^a^** | | | | **Male %** | **Age, yrs (mean ± SD or median, IQR)** | **Clinical parameters** | **Genetic parameters** | **Equation^b^** | **R2^c^** | | | | **MAE (mg/d)** | **Other** | **ROB** | **# EV** | **# CUA** |
| --- | --- | --- | --- | --- | --- | --- | --- | --- | --- | --- | --- | --- | --- | --- | --- | --- | --- | --- | --- | --- | --- | --- | --- | --- |
|  |  |  |  |  |  |  | **W** | **A** | **B** | **M** |  |  |  |  |  | **All** | **Cl** | **VKORC1** | **CYP2C9** |  |  |  |  |  |
| 240 | Ma 2018^185^ | Ini-pgx | 9 (IWPC sites) | AF, DVT, PE, HV, stroke, other | 1.7–3.3 | 4594 | 54 | 26 | 12 | 8 | - | Age in decades | Age, height, weight, race, enzyme inducer, amio | CYP2C9 *2, *3; VKORC1 -1639G>A | No explicit equation (linear regression) | - | - | - | - | - | IVC (n= 1149): MAE/ideal dose =1.22/ 46% (1.26/ 48%, 0.95/ 42%, 1.71/ 44% for Whites, Asians, and Blacks) | High | - | - |
| 241 |  | Ini-pgx | 9 (IWPC data) | AF, DVT, PE, HV, stroke, other | 1.7–3.3 | 4594 | 54 | 26 | 12 | 8 | - | Age in decades | Age, height, weight, race, enzyme inducer, amio | CYP2C9 *2, *3; VKORC1 -1639G>A | No explicit equation (support vector regression) | - | - | - | - | - | IVC (n= 1149): MAE/ideal dose =1.22/47% | High | - | - |
| 242 |  | Ini-pgx | 9 (IWPC data) | AF, DVT, PE, HV, stroke, other | 1.7–3.3 | 4594 | 54 | 26 | 12 | 8 | - | Age in decades | Age, height, weight, race, enzyme inducer, amio | CYP2C9 *2, *3; VKORC1 -1639G>A | No explicit equation (ridge regression) | - | - | - | - | - | IVC (n= 1149): MAE/ideal dose =1.22/46% | High | - | - |
| 243 |  | Ini-pgx | 9 (IWPC data) | AF, DVT, PE, HV, stroke, other | 1.7–3.3 | 4594 | 54 | 26 | 12 | 8 | - | Age in decades | Age, height, weight, race, enzyme inducer, amio | CYP2C9 *2, *3; VKORC1 -1639G>A | No explicit equation (neural network) | - | - | - | - | - | IVC (n= 1149): MAE/ideal dose =1.26/44% | High | - | - |
| 244 |  | Ini-pgx | 9 (IWPC data) | AF, DVT, PE, HV, stroke, other | 1.7–3.3 | 4594 | 54 | 26 | 12 | 8 | - | Age in decades | Age, height, weight, race, enzyme inducer, amio | CYP2C9 *2, *3; VKORC1 -1639G>A | No explicit equation (gradient boosting trees) | - | - | - | - | - | IVC (n= 1149): MAE/ideal dose =1.26/45% | High | - | - |
| 245 |  | Ini-pgx | 9 (IWPC data) | AF, DVT, PE, HV, stroke, other | 1.7–3.3 | 4594 | 54 | 26 | 12 | 8 | - | Age in decades | Age, height, weight, race, enzyme inducer, amio | CYP2C9 *2, *3; VKORC1 -1639G>A | No explicit equation (random forest) | - | - | - | - | - | IVC (n= 1149): MAE/ideal dose =1.33/43% | High | - | - |
| 246 |  | Ini-pgx | 9 (IWPC data) | AF, DVT, PE, HV, stroke, other | 1.7–3.3 | 4594 | 54 | 26 | 12 | 8 | - | Age in decades | Age, height, weight, race, enzyme inducer, amio | CYP2C9 *2, *3; VKORC1 -1639G>A | No explicit equation (extremely randomized trees) | - | - | - | - | - | IVC (n= 1149): MAE/ideal dose =1.45/39% | High | - | - |
| 247 |  | Ini-pgx | 9 (IWPC data) | AF, DVT, PE, HV, stroke, other | 1.7–3.3 | 4594 | 54 | 26 | 12 | 8 | - | Age in decades | Age, height, weight, race, enzyme inducer, amio | CYP2C9 *2, *3; VKORC1 -1639G>A | No explicit equation (k-nearest neighbors) | - | - | - | - | - | IVC (n= 1149): MAE/ideal dose =1.55/36% | High | - | - |
| 248 |  | Ini-pgx | 9 (IWPC data) | AF, DVT, PE, HV, stroke, other | 1.7–3.3 | 4594 | 54 | 26 | 12 | 8 | - | Age in decades | Age, height, weight, race, enzyme inducer, amio | CYP2C9 *2, *3; VKORC1 -1639G>A | No explicit equation (Stacked generalization framework - stacks 1 and 2 were similar) | - | - | - | - | - | IVC (n= 1149): MAE/ideal dose =1.19/48% (1.24/48%, 0.88/48%, 1.70/45% for Whites, Asians and Blacks) | High | - | - |

**Table S5. Continued**

| **#** | **Algorithm** | **Sub-type** | **Country** | **Indication** | **Target INR** | **N** | **Ethnicity (%)^a^** | | | | **Male %** | **Age, yrs (mean ± SD or median, IQR)** | **Clinical parameters** | **Genetic parameters** | **Equation^b^** | **R2^c^** | | | | **MAE (mg/d)** | **Other** | **ROB** | **# EV** | **# CUA** |
| --- | --- | --- | --- | --- | --- | --- | --- | --- | --- | --- | --- | --- | --- | --- | --- | --- | --- | --- | --- | --- | --- | --- | --- | --- |
|  |  |  |  |  |  |  | **W** | **A** | **B** | **M** |  |  |  |  |  | **All** | **Cl** | **VKORC1** | **CYP2C9** |  |  |  |  |  |
| 249 | Pei 2018^186^ | Ini-pgx | China | HV | 2.0–3.0 | 247 | - | 100 | - | - | 49 | 59 (49–67) | Age, BSA | VKORC1 -1639G>A; CYP4F2*3; CYP2C19 rs3814637 | Dose (mg/d) = 1.787 − 0.023 (Age) + 1.151 (BSA) + 0.917 (VKORC1 AG) + 4.619 (VKORC1 GG) + 0.595 (CYP4F2 *3*3) + 0.707 (CYP2C19 CC). | 58 | 10 | 43 | - | - | In entire cohort (+ temporal EV), R2 =57%, MAE = 0.74, MAPE = 27%, r=0.757, 61% with ideal dose | High | 2 | - |
| 250 | Selim 2018^187^ | Ini-pgx | Egypt | AF, DVT, PE, HV | 2.0–3.2 | 100 | - | - | - | 100 | 46 | 45 ± 13 | Age, BMI, weight, height, gender | CYP2C9 *2, *3, VKORC1 1639G>A | No explicit equation | 48 | 10 | 30 | 8 | - | - | High | - | - |
| 251 | Tavares 2018^188^ | Ini-pgx | Brazil | AF, CVA, TE, HV, other | 1.8–3.2 | 309 | 75 | - | 7 | 18 | 50 | 64 ± 14 | Age, gender, BMI, amio, race | CYP2C9 *2, *3, VKORC1 -1639G>A, CYP4F2 c.3435C>T | Dose (mg/wk) = 38.113 + 0.897 (CYP4F2) − 2.534 (CYP2C9) − 6.403 (VKORC1) − 0.190 (age) + 1.227 (male) + 0.302 (BMI) − 7.374 (amio) + 0.801 (self-declared race/color) | - | - | - | - | - | - | High | - | - |
| 252 |  | Ini-pgx | Brazil | AF, CVA, TE, HV, other | 1.8–3.2 | 309 | 75 | - | 7 | 18 | 50 | 64 ± 14 | Age, gender, BMI, amio, race | CYP2C9 *2, *3, VKORC1 -1639G>A, ABCB1 c.3435C>T | Dose (mg/wk) = 40.547 − 2.285 (ABCB1) − 2.596 (CYP2C9) − 6.394 (VKORC1) − 0.189 (age) + 1.028 (male) + 0.318 (BMI) − 7.823 (amio) + 0.649 (self-declared race/color) | - | - | - | - | - | - | High | - | - |
| 253 |  | Ini-pgx | Brazil | AF, CVA, TE, HV, other | 1.8–3.2 | 233 | 100 | - | - | - | - | - | Age, gender, BMI, amio | CYP2C9 *2, *3, VKORC1 -1639 G>A, CYP4F2 c.3435C>T | Dose (mg/wk) = 32.668 + 1.099 (CYP4F2) − 4.159 (CYP2C9) − 7.448 (VKORC1) − 0.119 (age) + 0.575 (male) + 0.460 (BMI) − 5.877 (amio) | - | - | - | - | - | - | High | - | - |
| 254 |  | Ini-pgx | Brazil | AF, CVA, TE, HV, other | 1.8–3.2 | 233 | 100 | - | - | - | - | - | Age, gender, BMI, amio | CYP2C9 *2, *3, VKORC1 -1639 G>A, ABCB1 c.3435C>T | Dose (mg/wk) = 34.833 − 1.606 (ABCB1) − 4.344 (CYP2C9) − 7.371 (VKORC1) − 0.121 (age) + 0.455 (male) + 0.473 (BMI) – 6.277 (amio) | - | - | - | - | - | - | High | - | - |
| 255 |  | Ini-pgx | Brazil | AF, CVA, TE, HV, other | 1.8–3.2 | 76 | - | - | 29 | 71 | - | - | Age, gender, BMI, amio | CYP2C9 *2, *3, VKORC1 -1639 G>A, CYP4F2 c.3435C>T | Dose (mg/wk) = 44.703 + 1.185 (CYP4F2) + 1.771 (CYP2C9) − 3.062 (VKORC1) − 0.325 (age) + 4.091 (male) + 0.120 (BMI) − 10.926 (amio) | - | - | - | - | - | - | High | - | - |
| 256 |  | Ini-pgx | Brazil | AF, CVA, TE, HV, other | 1.8–3.2 | 76 | - | - | 29 | 71 | - | - | Age, gender, BMI, amio | CYP2C9 *2, *3, VKORC1 -1639 G>A, ABCB1 c.3435C>T | Dose (mg/wk) = 46.859 − 4.762 (ABCB1) + 2.849 (CYP2C9) − 3.353 (VKORC1) − 0.312 (age) + 3.209 (male) + 0.156 (BMI) − 11.424 (amio) | - | - | - | - | - | - | High | - | - |
| 257 | Tavares 2018^189^ | Ini-pgx | Brazil | AF, CVA, TE, HV, other | 1.8–3.2 | 309 | 75 | - | 7 | 18 | 50 | 64 ± 14 | Age, gender, weight, height, race, amio, enzyme inducers | CYP2C9*2, *3; VKORC1 -1639G>A | Dose (mg/wk) = 24.55 + 0.41 (self-declared race) −7.37 (amio) − 1.70 (male) − 1.59 (age) + 0.16 (weight) + 0.07 (height) + 22.37 (inducers) − 2.96 (CYP2C9) − 7.00 (VKORC1) | 39 | 20 | 21 | 2 | - | mean ‘relative dose error’ = 0.08 mg/wk | High | 1 | - |

**Table S5. Continued**

| **#** | **Algorithm** | **Sub-type** | **Country** | **Indication** | **Target INR** | **N** | **Ethnicity (%)^a^** | | | | **Male %** | **Age, yrs (mean ± SD or median, IQR)** | **Clinical parameters** | **Genetic parameters** | **Equation^b^** | **R2^c^** | | | | **MAE (mg/d)** | **Other** | **ROB** | **# EV** | **# CUA** |
| --- | --- | --- | --- | --- | --- | --- | --- | --- | --- | --- | --- | --- | --- | --- | --- | --- | --- | --- | --- | --- | --- | --- | --- | --- |
|  |  |  |  |  |  |  | **W** | **A** | **B** | **M** |  |  |  |  |  | **All** | **Cl** | **VKORC1** | **CYP2C9** |  |  |  |  |  |
| 258 | Tavares 2018^189^ | Ini-pgx | Brazil | AF, CVA, TE, HV, other | 1.8–3.2 | 309 | 75 | - | 7 | 18 | 50 | 64 ± 14 | Age, gender, weight, height, race, amio, enzyme inducers | CYP2C9*2, *3; VKORC1 -1639G>A; CYP4F2*3; ABCB1 3435C>T | Dose (mg/wk) = 22.47 + 0.77 (self-declared race) − 7.31 (amio) − 1.92 (gender) − 1.53 (age) + 0.16 (weight) + 0.08 (height) + 21.75 (inducers) − 3.11 (CYP2C9) − 7.00 (VKORC1) + 1.85 (CYP4F2) − 1.68 (ABCB1) | 41 | 18 | 22 | 3 | - | mean ‘relative dose error’ = -0.03 mg/wk | High | 1 | - |
| 259 | Wang 2018^190^ (***children***) | Ini-pgx | China | Kawasaki disease | 1.5–2.5 | 47 | - | Y | - | - | 79 | 4 ± 4 | Weight | VKORC1 -1639G>A | Dose (mg/kg/d) = 0.133 − 0.002 (weight) + 0.026 (VKORC1 GG/GA) | 44 | 33 | 11 | - | - | - | High | - | - |
| 260 | Danese 2019^191^ | Ini-pgx | 17 (31  studies) | AF, CMP, orthopedic, other | 1.5–4.0 | 3016 | 100 | - | - | - | 54 (of 8806) | 62 ± 16 (for 8806) | Age, BMI, gender, indication | CYP2C9 *2, *3; VKORC1 -1639G>A; CYP4F2*3 | Log dose (mg/wk) = 3.981 − 0.009 (age) + 0.010 (BMI) + 0.123 (male) − 0.043 (AF/ CMP/orthopedic) − 0.231 (CYP2C9*1*2) − 0.513 (CYP2C9*2*2) − 0.387 (CYP2C9*1*3) − 1.316 (CYP2C9*3*3) − 0.266 (VKORC1 AG) − 0.666 (VKORC1 AA) + 0.073 (CYP4F2 CT) + 0.191 (CYP4F2 TT) | 51 | - | - | - | - | IVC (n = 1532), R2 = 52 | High | - | - |
| 261 |  | Ini-pgx | 17 (31  studies) | AF, CMP, orthopedic, other | 1.5–4.0 | 534 | - | - | 100 | - | - | - | Age, BMI, gender, indication | CYP2C9 *2, *3, *5; VKORC1 -1639G>A; CYP4F2*3 | Log dose (mg/wk) = 3.875 − 0.009 (age) + 0.010 (BMI) + 0.152 (male) − 0.090 (AF/ CMP/orthopedic) − 0.007 (CYP2C9*1*2) − 0.469 (CYP2C9*1*3) − 0.436 (CYP2C9*1 *5) − 0.284 (VKORC1 AG) − 0.281 (VKORC1 AA) − 0.0382 (CYP4F2 CT) + 0.300 (CYP4F2 TT) | 30 | - | - | - | - | IVC (n = 288), R2 = 22 | High | - | - |
| 262 |  | Ini-pgx | 17 (31  studies) | AF, CMP, orthopedic, other | 1.5–4.0 | 292 | - | 100 | - | - | - | - | Age, BMI, gender, indication | CYP2C9 *2, *3; VKORC1 -1639G>A; CYP4F2*3 | Log dose (mg/wk) = 3.484 − 0.005 (age) + 0.014 (BMI) + 0.058 (male) − 0.027 (AF/ CMP/orthopedic) − 0.114 (CYP2C9*1*2) − 0.224 (CYP2C9*1*3) − 1.065 (CYP2C9*3 *3) − 0.422 (VKORC1 AG) − 0.827 (VKORC1 AA) + 0.117 (CYP4F2 CT) + 0.124 (CYP4F2 TT) | 45 | - | - | - | - | IVC (n = 146), R2 = 42 | High | - | - |
| 263 | Roche-Lima 2019^192^ | Ini-Pgx | Puerto Rico | DVT, PE, AF, HV, stroke | 2.0–3.0 | 154 | - | - | - | 100 |  | Categorized | Age, weight, height, ancestry, smoking, DM, indication, CM (aspirin, statin, azoles, clopidogrel), HTN | FMO2 rs2020870; ABCB1 rs10276036; SLCO1B3 rs3764006; CYP2C9 rs1856908; VKORC1 1173C> T/-1639G>A;  CYP2C9*2; CYP4F2*3; NQO1*2 | No explicit equation (recursive partitioning) | - | - | - | - | 0.9 (IVC) | IVC (n=36), Ideal dose = 72% | High | - | - |
| 264 |  | Ini-Pgx | Puerto Rico | DVT, PE, AF, HV, stroke | 2.0–3.0 | 154 | - | - | - | 100 |  | Categorized |  |  | No explicit equation (multivariate  adaptive regression splines) | - | - | - | - | 1.22 (IVC) | IVC (n=36), Ideal dose = 56% | High | - | - |
| 265 |  | Ini-Pgx | Puerto Rico | DVT, PE, AF, HV, stroke | 2.0–3.0 | 154 | - | - | - | 100 |  | Categorized |  |  | No explicit equation (random forest regression) | - | - | - | - | 0.68 (IVC) | IVC (n=36), Ideal dose = 81% | High | - | - |
| 266 |  | Ini-Pgx | Puerto Rico | DVT, PE, AF, HV, stroke | 2.0–3.0 | 154 | - | - | - | 100 |  | Categorized |  |  | No explicit equation (artificial neural networks) | - | - | - | - | 1.39 (IVC) | IVC (n=36), Ideal dose = 58% | High | - | - |

**Table S5. Continued**

| **#** | **Algorithm** | **Sub-type** | **Country** | **Indication** | **Target INR** | **N** | **Ethnicity (%)^a^** | | | | **Male %** | **Age, yrs (mean ± SD or median, IQR)** | **Clinical parameters** | **Genetic parameters** | **Equation^b^** | **R2^c^** | | | | **MAE (mg/d)** | **Other** | **ROB** | **# EV** | **# CUA** |
| --- | --- | --- | --- | --- | --- | --- | --- | --- | --- | --- | --- | --- | --- | --- | --- | --- | --- | --- | --- | --- | --- | --- | --- | --- |
|  |  |  |  |  |  |  | **W** | **A** | **B** | **M** |  |  |  |  |  | **All** | **Cl** | **VKORC1** | **CYP2C9** |  |  |  |  |  |
| 267 | Roche-Lima 2019^192^ | Ini-Pgx | Puerto Rico | DVT, PE, AF, HV, stroke | 2.0–3.0 | 154 | - | - | - | 100 |  | Categorized | Age, weight, height, ance-stry, smoking, DM, indication, CM (aspirin, statin, azoles, clopidogrel), HTN | FMO2 rs2020870; ABCB1; rs10276036, SLCO1B3 rs3764006; CYP2C9 rs1856908 | No explicit equation (support vector regression) | - | - | - | - | 0.98 (IVC) | IVC (n=36), Ideal dose = 61% | High | - | - |
| 268 |  | Ini-Pgx | Puerto Rico | DVT, PE, AF, HV, stroke | 2.0–3.0 | 154 | - | - | - | 100 |  | Categorized | Age, weight, height, ancestry, smoking, DM, indication, CM (aspirin, statin, azoles, clopidogrel), HTN | FMO2 rs2020870; ABCB1 rs10276036; SLCO1B3 rs3764006; CYP2C9 rs1856908; VKORC1 1173C> T/-1639G>A;  CYP2C9*2; CYP4F2*3; NQO1*2 | No explicit equation (K-nearest neighbour, K = 1) | - | - | - | - | 0.97 (IVC) | IVC (n=36), Ideal dose = 67% | High | - | - |
| 269 |  | Ini-Pgx | Puerto Rico | DVT, PE, AF, HV, stroke | 2.0–3.0 | 154 | - | - | - | 100 |  | Categorized |  |  | No explicit equation (K-nearest neighbour, K = 2) | - | - | - | - | 0.9 (IVC) | IVC (n=36), Ideal dose = 69% | High | - | - |
| 270 |  | Ini-Pgx | Puerto Rico | DVT, PE, AF, HV, stroke | 2.0–3.0 | 154 | - | - | - | 100 |  | Categorized |  |  | No explicit equation (K-nearest neighbour, K = 3) | - | - | - | - | 0.88 (IVC) | IVC (n=36), Ideal dose = 72% | High | - | - |
| 271 |  | Ini-Pgx | Puerto Rico | DVT, PE, AF, HV, stroke | 2.0–3.0 | 154 | - | - | - | 100 |  | Categorized | Age, weight, height, ancestry, smoking, DM, indication, CM (aspirin, statin, azoles, clopidogrel), HTN | FMO2 rs2020870; ABCB1; rs10276036, SLCO1B3 rs3764006; CYP2C9 rs1856908 | No explicit equation (reduces error pruning tree classifier) | - | - | - | - | 1.41 (IVC) | IVC (n=36), Ideal dose = 47% | High | - | - |
| 272 | Shahabi 2019^193^ | Ini-pgx | Canada | AF, HV, mitral stenosis | 2.0–3.5 | 906 | 100 | - | - | - | 61 | 70 ± 12 | Age, gender, vitamin K intake, target INR | CYP2C9*2, *3; VKORC1 -1639G>A, 3730 G>A, 6009C>T | Ln dose (mg/d) = 2.68 − 0.0115 (age) 0.1335 (female) + 0.0133 (vitamin K intake) − 0.0524 (target INR 2-3) – 0.2512 (CYP2C9) − 0.3126 (VKORC1). | 47 | - | 24 | 9 | - | - | High | - | - |
| 273 | Tao 2019^194^ | Ini-pgx | China | AF, HV, DVT, PE, other | 2.0–3.0 | 517 | - | 100 | - | - | 57 | - | Age, height, weight, gender, amio, target INR | CYP2C9*3; VKORC1 -1639G>A | No explicit equation (ensemble learning method [evolutionary fuzzy c-mean clustering] with support vector regression - Used to develop a warfarin dosage predictive tool called “WarfarinSeer”) | 44 | 38 | - | - | 0.68 | 46% patients with ideal dose | High | - | - |
| 274 |  | Ini-pgx | China | AF, HV, DVT, PE, other | 2.0–3.0 | 517 | - | 100 | - | - | 57 | - |  |  | No explicit equation (convolution neural network) | 36 | - | - | - | - | - | High | - | - |
| 275 |  | Ini-pgx | China | AF, HV, DVT, PE, other | 2.0–3.0 | 517 | - | 100 | - | - | 57 | - |  |  | No explicit equation (back propagation network) | - | - | - | - | - | - | High | - | - |

**Table S5. Continued**

| **#** | **Algorithm** | **Sub-type** | **Country** | **Indication** | **Target INR** | **N** | **Ethnicity (%)^a^** | | | | **Male %** | **Age, yrs (mean ± SD or median, IQR)** | **Clinical parameters** | **Genetic parameters** | **Equation^b^** | **R2^c^** | | | | **MAE (mg/d)** | **Other** | **ROB** | **# EV** | **# CUA** |
| --- | --- | --- | --- | --- | --- | --- | --- | --- | --- | --- | --- | --- | --- | --- | --- | --- | --- | --- | --- | --- | --- | --- | --- | --- |
|  |  |  |  |  |  |  | **W** | **A** | **B** | **M** |  |  |  |  |  | **All** | **Cl** | **VKORC1** | **CYP2C9** |  |  |  |  |  |
| 276 | Tao 2019^194^ | Ini-pgx | China | AF, HV, DVT, PE, other | 2.0–3.0 | 517 | - | 100 | - | - | 57 | - | Age, height, weight, gender, amio, target INR | CYP2C9*3; VKORC1 -1639G>A | No explicit equation (general regression neural network) | 23 | - | - | - | - | - | High | - | - |
| 277 |  | Ini-pgx | China | AF, HV, DVT, PE, other | 2.0–3.0 | 517 | - | 100 | - | - | 57 | - |  |  | No explicit equation (support vector regression) | - | - | - | - | 0.71 | - | High | - | - |
| 278 |  | Ini-pgx | China | AF, HV, DVT, PE, other | 2.0–3.0 | 517 | - | 100 | - | - | 57 | - |  |  | No explicit equation (evolutionary ensemble model) | 30 | - | - | - | 0.77 | - | High | - | - |
| 279 |  | Ini-pgx | China | AF, HV, DVT, PE, other | 2.0–3.0 | 517 | - | 100 | - | - | 57 | - |  |  | No explicit equation (boosted binary regression trees) | 30 | - | - | - | 0.76 | With ideal dose= 37% | High | - | - |
| 280 |  | Ini-pgx | China | AF, HV, DVT, PE, other | 2.0–3.0 | 517 | - | 100 | - | - | 57 | - |  |  | No explicit equation (random forest) | - | - | - | - | - | - | High | - | - |
| 281  -  285 | Tao 2019^195^ | Ini-pgx | China | - | 2.0–3.0 | 229 | - | 100 | - | - | 58 (of 247) | Age categorized | Combinations of age, height, weight, gender, amio, drinking, ALT, serum creatinine | CYP2C9*3, VKORC1-1639G>A | No explicit equation (genetic programming) | 35-44 | - | - | - | - | Ideal dose = 51-53%  IVC (n=60): R2=40-44%, ideal dose = 46-53% | High | - | - |
| 286  -  290 |  | Ini-pgx | China | - | 2.0–3.0 | 229 | - | 100 | - | - | 58 (of 247) | Age categorized | Combinations of age, height, weight, gender, amio, drinking, ALT, serum creatinine | CYP2C9*3, VKORC1-1639G>A | No explicit equation (ε-support vector regression) | 37-50 | - | - | - | - | Ideal dose = 52-53%  IVC (n=60): R2=37-43%, ideal dose = 47-48% | High | - | - |
| 291  -  295 |  | Ini-pgx | China | - | 2.0–3.0 | 229 | - | 100 | - | - | 58 (of 247) | Age categorized | Combinations of age, height, weight, gender, amio, drinking, ALT, serum creatinine | CYP2C9*3, VKORC1-1639G>A | No explicit equation (v-support vector regression) | 32-49 | - | - | - | - | Ideal dose = 49-53%  IVC (n=60): R2=41-44%, ideal dose = 47-50% | High | - | - |
| 296  -  300 |  | Ini-pgx | China | - | 2.0–3.0 | 229 | - | 100 | - | - | 58 (of 247) | Age categorized | Combinations of age, height, weight, gender, amio, drinking, ALT, serum creatinine | CYP2C9*3, VKORC1-1639G>A | No explicit equation (back propagation neural network) | 35-43 | - | - | - | - | Ideal dose = 45-56%  IVC (n=60): R2=11-21%, ideal dose = 30-41% | High | - | - |
| 301  -  305 |  | Ini-pgx | China | - | 2.0–3.0 | 229 | - | 100 | - | - | 58 (of 247) | Age categorized | Combinations of age, height, weight, gender, amio, drinking, ALT, serum creatinine | CYP2C9*3, VKORC1-1639G>A | No explicit equation (general regression neural network) | 76-84 | - | - | - | - | Ideal dose = 75-98%  IVC (n=60): R2=13-18%, ideal dose = 35-40% | High | - | - |
| 306  -  311 |  | Ini-pgx | China | - | 2.0–3.1 | 229 | - | 100 | - | - | 58 (of 247) | Age categorized | Age, weight, amiodarone | CYP2C9*3, VKORC1-1639G>A | No explicit equation (evolutionary ensemble modelling) | 42-50 | - | - | - | - | - | High | - | - |

**Table S5. Continued**

| **#** | **Algorithm** | **Sub-type** | **Country** | **Indication** | **Target INR** | **N** | **Ethnicity (%)^a^** | | | | **Male %** | **Age, yrs (mean ± SD or median, IQR)** | **Clinical parameters** | **Genetic parameters** | **Equation^b^** | **R2^c^** | | | | **MAE (mg/d)** | **Other** | **ROB** | **# EV** | **# CUA** |
| --- | --- | --- | --- | --- | --- | --- | --- | --- | --- | --- | --- | --- | --- | --- | --- | --- | --- | --- | --- | --- | --- | --- | --- | --- |
|  |  |  |  |  |  |  | **W** | **A** | **B** | **M** |  |  |  |  |  | **All** | **Cl** | **VKORC1** | **CYP2C9** |  |  |  |  |  |
| 312 | Yang 2019^196^ (***children***) | Ini-pgx | China | Kawasaki disease | 1.0–2.5 | 44 | - | 100 | - | - | 77 | 4 ± 4 | Height | CYP2C9*3; VKORC1 1173C>T | Dose (mg/day) = -0.018 + 0.023 (height) − 0.653 (VKORC1 TT) − 0.46 (CYP2C9 *1*3) | 73 | 61 | 8 | 4 | - | - | High | - | - |
| 313 | Bader 2020^197^ | Ini-Pgx | Qatar | AF, HV, VTE, other | 2.0–3.5 for most | 104 | - | - | - | 100 | 37 | 62 ± 13 | Smoking, HTN, heart failure (HF) | CYP2C9*2, *3; VKORC1 −1639G>A | Log10 dose (mg/wk) = 1.674 − 0.178 (HTN) − 0.176 (HF) + 0.251 (smoking) −0.382 (VKORC1 per variant allele) − 0.33 (CYP2C9*2/*3) | 39 | - | 15 | 11 | - | IVC (n=45), r = 0.711, MAPE = 54% | High | - | - |
| 314 | Cho 2020^198^ | Ini-pgx | Korea | AF, cerebral infarction, DVT, PE, HV | - | 109 | - | 100 | - | - | 62 | 70 (range 29–91) | Age, BSA, gender | CYP2C9*3, VKORC1 1173C>T | Ln dose (mg/wk) = 3.223 − 0.009 (age) + 0.577 (BSA) + 0.178 (female) − 0.481 (CYP2C9*1*3) + 0.227 (VKORC1 CC). | 44 | - | - | - | - | r=0.641, MPE = -0.19, RMSE = 1.44, ideal dose = 61% | High | - | - |
| 315 | Li 2020^199^ | Ini-Pgx | China | AF, PE, DVT, other | 1.6–2.8 | 214 | - | 100 | - | - | 53 | 73 ± 11 | Age, BMI, Amio | CYP2C9*3, VKORC1 -1629>A, ApoE rs7412 | Dose (mg/d) = 8.284 – 1.117 (VKORC1 per variant allele) – 0.030 (Age) – 0.477 (Amio) – 0.648 (CYP2C9*3) – 0.356 (ApoE per variant allele) + 0.041 (BMI) | 37 | 14 | 18 | 3 | 0.53 | r = 0.716 | High | - | - |
| 316 | Dobrzanski 1983^200^ | Ini-cl | UK | - | 2.0–3.0 | 100 | Y | - | - | - | - | 69 ± 11 | Age, weight | - | Dose (mg/d) = 4.04 + 0.053 (weight) − 0.057 (age) | 53 | - | - | - | - | - | High | - | - |
| 317 | Wynne 1995^201^ | Ini-cl | UK | TE | - | 39 | Y | - | - | - | 54 | range 50–87 | Age, liver volume | - | Dose (mg/wk) = 36.4 − 0.37 (age) + 0.012 (liver volume) | 34 | - | - | - | - | - | High | - | - |
| 318 | Tabrizi 2002^29^ | Ini-cl | USA | AF, DVT, HV, CAD, other | 1.8–3.5 | 153 | 78 | - | - | 22 | 56 | 59 ± 15 | Age, weight | - | No explicit equation | 14 | - | - | - | - | - | High | - | - |
| 319 | Shine 2003^202^ | Ini-cl | USA | DVT, AF, PE, other | 2.0–3.0 | 101 | Y | - | - | - | - | - | Age, weight, serum albumin, malignancy | - | Dose (mg/d) = 1/3 [11.23 – age (0.10) + weight (0.09) + serum albumin (7.75) − 8.39 (active malignancy) | 25 | - | - | - | - | - | High | - | - |
| 320 |  | Ini-cl | USA | DVT, AF, PE, other | 2.0–3.0 | 127 | Y | - | - | - | - | - | Age, weight, serum albumin, malignancy | - | Dose (mg/d) = 1/3 [14.05 – age (0.25) + weight (0.07) + serum albumin (3.0) –1.15 (active malignancy) | 30 | - | - | - | - | - | High | - | 1 |
| 321 | Caldwell 2008^46^ | Ini-cl | USA | AF, DVT, PE, HV, other | 2.0–3.5 | 1051 | Y | - | - | - | - | - | Sex, age, BSA, HV | - | Ln dose (mg/wk) = 3.458 + 0.085 (male) + 0.633 (HV) – 0.009 (age) + 0.258 (BSA) – 0.007 (age × HV) (from Burmester 2011^47^) | - | - | - | - | - | - | High | 1 | 1 |
| 322 | Gage 2008^48^ | Ini-cl | USA | AF, DVT, PE, HV | 1.5–3.2 for most | 1015 | 83 | - | 15 | 2 | 64 | 65 ± 14 | Age, BSA, amio, target INR, smoking, race, DVT/PE | - | Ln dose (mg/d) = 0.613 + 0.425 (BSA) – 0.0075 (age) + 0.156 (Blacks) + 0.216 (target INR) – 0.257 (amio) + 0.108 (smoker) + 0.0784 (DVT/PE) | 22 | - | - | - | - | - | High | 14 | 5 |
| 323 | IWPC 2009^3^ | Ini-cl | 9 (21 sites) | AF, DVT, PE, heart valves, stroke, other | 2.0–3.0 | 4043 | 55 | 30 | 9 | 6 | - | Age in decades | Age, height, weight, race, enzyme inducers, amio | - | √dose (mg/wk) = 4.0376 – 0.2546 (age in decades) + 0.0118 (height) + 0.0134 (weight) – 0.6752(Asians) + 0.4060 (Blacks) + 0.0443 (missing or mixed race) + 1.2799 (inducers) – 0.5695 (amio) | 27 | - | - | - | 1.43 | IVC (n = 1009), R2 = 26%, MAE = 1.41; ideal dose = 39%; plus 1 outlier, MAE =1.44, R2=21% | High | 13 | - |

**Table S5. Continued**

| **#** | **Algorithm** | **Sub-type** | **Country** | **Indication** | **Target INR** | **N** | **Ethnicity (%)^a^** | | | | **Male %** | **Age, yrs (mean ± SD or median, IQR)** | **Clinical parameters** | **Genetic parameters** | **Equation^b^** | **R2^c^** | | | | **MAE (mg/d)** | **Other** | **ROB** | **# EV** | **# CUA** |
| --- | --- | --- | --- | --- | --- | --- | --- | --- | --- | --- | --- | --- | --- | --- | --- | --- | --- | --- | --- | --- | --- | --- | --- | --- |
|  |  |  |  |  |  |  | **W** | **A** | **B** | **M** |  |  |  |  |  | **All** | **Cl** | **VKORC1** | **CYP2C9** |  |  |  |  |  |
| 324 | Cen 2010^66^ | Ini-cl | China | HV | 1.5–3.0 | 222 | - | 100 | - | - | 47 | 45 ± 12 | age, BSA, gender, amio | - | Ln dose (mg/d) = 0.24015 + 0.02227 (gender) – 0.00096 (age) + 0.15350 (BSA) – 0.23511 (amio) (from Xu 2018^67^) | 14 | - | - | - | - | - | High | 1 | 1 |
| 325 | Zambon 2011^21^ | Ini-cl | Italy | AF, DVT, other | 2.0–3.0 | 274 | 100 | - | - | - | 65 | 74 (range 39–92) | Age, BSA, ALT | - | √dose (mg/wk) = 2.92410 – 0.01943 (age) + 1.94651 (BSA) + 0.01988 (ALT) | 13 | - | - | - | - | Total cohort (n=371) ideal dose=31%, IVC (n=97) R2 = 6%, | High | - | - |
| 326 | Bress 2012^101^ | Ini-cl | USA | DVT, PE, AF, HV, stroke/TIA | 2.0–3.5 | 258 | - | - | 100 | - | 26 (of 260) | 55 ± 16 (for 260) | Age, BSA, HTN | - | Log dose (mg/wk) = 3.60 – 0.006 (age) + 0.32 (BSA) – 0.19 (HTN) | 23 | - | - | - | - | - | High | - | - |
| 327 |  | Ini-cl | USA | DVT, PE, AF, HV, stroke/TIA | 2.0–3.5 | 53 | 100 | - | - | - | 38 | 58 ± 15 | Age, BSA, AF | - | Log dose (mg/wk) = 2.24 – 0.009 (age) + 0.94 (BSA) – 0.36 (AF) | 36 | - | - | - | - | - | High | - | - |
| 328 | Hu 2012^203^ | Ini-cl | Taiwan | - | 1.0–3.0 | 587 | - | 100 | - | - | 49 | 72 ± 13 | Age, gender, weight, serum creatinine, CHF, ALT, concomitant medications | - | No explicit equation (k-nearest neighbor) | - | - | - | - | 0.22 | - | High | - | - |
| 329 |  | Ini-cl | Taiwan | - | 1.0–3.0 | 587 | - | 100 | - | - | 49 | 72 ± 13 |  | - | No explicit equation (support vector regression) | - | - | - | - | 0.21 | - | High | - | - |
| 330 |  | Ini-cl | Taiwan | - | 1.0–3.0 | 587 | - | 100 | - | - | 49 | 72 ± 13 |  | - | No explicit equation (model tree) | - | - | - | - | 0.22 | - | High | - | - |
| 331 |  | Ini-cl | Taiwan | - | 1.0–3.0 | 587 | - | 100 | - | - | 49 | 72 ± 13 |  | - | No equation (multilayer perceptron) | - | - | - | - | 0.23 | - | High | - | - |
| 332 |  | Ini-cl | Taiwan | - | 1.0–3.0 | 587 | - | 100 | - | - | 49 | 72 ± 13 |  | - | No explicit equation (bagged k-nearest neighbour) | - | - | - | - | 0.22 | - | High | - | - |
| 333 |  | Ini-cl | Taiwan | - | 1.0–3.0 | 587 | - | 100 | - | - | 49 | 72 ± 13 |  | - | No explicit equation (bagged support vector regression) | - | - | - | - | 0.21 | - | High | - | - |
| 334 |  | Ini-cl | Taiwan | - | 1.0–3.0 | 587 | - | 100 | - | - | 49 | 72 ± 13 |  | - | No explicit equation (bagged model tree) | - | - | - | - | 0.22 | - | High | - | - |
| 335 |  | Ini-cl | Taiwan | - | 1.0–3.0 | 587 | - | 100 | - | - | 49 | 72 ± 13 |  | - | No explicit equation (bagged multilayer perceptron) | - | - | - | - | 0.22 | - | High | - | - |
| 336 |  | Ini-cl | Taiwan | - | 1.0–3.0 | 587 | - | 100 | - | - | 49 | 72 ± 13 |  | - | No equation (voting with 4 classifiers) | - | - | - | - | 0.21 | - | High | - | - |
| 337 |  | Ini-cl | Taiwan | - | 1.0–3.0 | 587 | - | 100 | - | - | 49 | 72 ± 13 |  | - | No explicit equation (bagged voting with 4 classifiers) | - | - | - | - | 0.21 | - | High | - | - |
| 338 | Ramirez 2012^114^ | Ini-cl | USA | DVT, PE, AF, stroke, orthopedic, other | 2.0–3.0 | 1167 | 88 | - | 12 | - | 55 | 66 (95% CI 35–87) | Age, gender, BSA, smoking, AF, DVT, PE, amio, race | - | No explicit equation | 25 (White 23, Black 24) | - | - | - | 1.73 (White1.69, Black 1.91) | - | High | - | - |
| 339 | Tatarunas 2012^116^ | Ini-cl | Lithuania | HV | 2.0–3.5 | 189 | 100 | - | - | - | 57 | 66 (range 18–85) | Age, weight, hepatic function | - | Dose (mg/d) = 8.415 – 0.067 (age) + 0.024 (weight) – 0.843 (hepatic function) | 12 | - | - | - | - | - | High | - | - |
| 340 |  | Ini-cl | Lithuania | HV | 2.0–3.5 | 189 | 100 | - | - | - | 57 | 66 (range 18–85) | Age, weight, hepatic malfunction, cephalosporin, amio, diuretics, ibuprofen or diclofenac, omeprazole, β-blockers | - | Dose (mg/d) = 9.623 – 0.052 (age) + 0.025 (weight) – 0.994 (hepatic function) + 3.021 (cephalosporin) – 2.243 (amio) – 1.485 (ibubrofen/diclofenac) – 1.349 (omeprazole) – 1.216 (loop diuretic) – 0.965 (β-blocker) | 32 | - | - | - | - | - | High | - | - |

**Table S5. Continued**

| **#** | **Algorithm** | **Sub-type** | **Country** | **Indication** | **Target INR** | **N** | **Ethnicity (%)^a^** | | | | **Male %** | **Age, yrs (mean ± SD or median, IQR)** | **Clinical parameters** | **Genetic parameters** | **Equation^b^** | **R2^c^** | | | | **MAE (mg/d)** | **Other** | **ROB** | **# EV** | **# CUA** |
| --- | --- | --- | --- | --- | --- | --- | --- | --- | --- | --- | --- | --- | --- | --- | --- | --- | --- | --- | --- | --- | --- | --- | --- | --- |
|  |  |  |  |  |  |  | **W** | **A** | **B** | **M** |  |  |  |  |  | **All** | **Cl** | **VKORC1** | **CYP2C9** |  |  |  |  |  |
| 341 | Sharabiani 2013^204^ | Ini-cl | USA | DVT, PE, other | 2.0–3.0 | ~235 | - | - | 100 | - | - | - | Age, BSA, smoking, HTN, DM, amio | - | Log dose (mg/wk) = 3.778 + 0.117 (BSA) – 0.004 (age) + 0.066 (smoker) – 0.068 (HTN) + 0.051 (DM) – 0.192 (amio) | - | - | - | - | - | IVC (n~59) MAE = 1.74, RMSE = 2.07 | High | - | - |
| 342 |  | Ini-cl | USA | DVT/PE, other | 2.0–3.0 | ~235 | - | - | 100 | - | - | - | Age, gender, BSA, amio, HTN, smoking, cancer, DVT/PE, DM, stable INR | - | No explicit equation (artificial neural network) | - | - | - | - | - | IVC (n~59) MAE = 2.89, RMSE = 3.09 | High | - | - |
| 343 |  | Ini-cl | USA | DVT/PE, other | 2.0–3.0 | ~235 | - | - | 100 | - | - | - | Age, gender, BSA, amio, HTN, smoking, cancer, DVT/PE, DM, stable INR | - | No explicit equation (support vector regression) | - | - | - | - | - | IVC (n~59) MAE = 2.30, RMSE = 2.47 | High | 1 | - |
| 344 | Tatarunas 2014^145^ | Ini-cl | Lithuania | HV | 2.0–3.5 | 189 | 100 | - | - | - | 62 | 68 (range 27–87) | Age, weight, target INR, TSH, amio, CCB, benzodiazepine | - | Dose (mg/d) = 7.121 – 0.055 (age) + 0.032 (weight) – 0.200 (INR) – 0.593 (TSH) – 1.086 (amio) – 1.157 (benzodiazepines) – 1.667 (CCB) | 26 | - | - | - | - | - | High | - | - |
| 345 | Krishna Kumar 2014^139^ | Ini-cl | India | RHD, IHD, DVT, dilated CMP, stroke, other | 2.0–3.5 | 240 | - | 100 | - | - | 37 | 43 ± 11 | Age, weight, indication | - | No explicit equation | - | - | - | - | - | 36% within 1 mg/d | High | - | - |
| 346 | Sharabiani 2015^205^ | Ini-cl | 9 (IWPC sites) | DVT, PE, HV, CHF, other | 1.8–3.5 | 2119 | 63 | 22 | 15 | - | 43 (of 4237) | Age categorized | High dose class: race, age, DVT, PE, DM, HV, lovastatin, BSA, amio, target INR, enzyme Low dose class: race, age, DVT, PE, aspirin, amio, azoles, smoking, Enzyme, BSA | - | High dose class: Ln dose (mg/wk) = 2.85332 − 0.07370 (race) − 0.06513 (age) + 0.10246 (DVT/PE) + 0.05766 (DM) + 0.03742 (HV)− 0.08763 (lovastatin) − 0.12542 (amio) + 0.13207 (target INR) + 0.12403 (enzyme) + 0.34487 (BSA)  Low dose class: Ln dose (mg/wk) = 3.44056 − 0.03649 (race) − 0.04820 (age) + 0.05059 (DVT/PE) − 0.03060 (aspirin) − 0.06150 (amio) − 0.20356 (afungal azoles) + 0.05744 (smoker) +0.10923 (enzyme) + 0.24601 (BSA) | - | - | - | - | - | IVC (n=2118) MAE = 1.2, RMSE=1.66 [high and low dose equations considered as one model] | High | - | - |
| 347 | Rouleau-Mailloux 2016^167^ | Ini-cl | Canada | Mitral stenosis, AF, HV, other | 2.0–3.5 | 969 | 97 | <1 | 1 | 2 | 61 | 71 (range 18–95) | Age, height, weight, target INR, RPA | - | Ln dose (mg/wk) = 3.193 − 0.012 (age) + 0.510 (height) + 0.002 (weight) | 15 | - | - | - | - | - | High | - | - |
| 348 | Wiley 2017^179^ | Ini-cl | USA | DVT, PE, AF | 1.9–3.2 | 2181 | 88 | - | 12 | - | 58 | 66 ± 15 | Age, BSA, smoking, amio, enzyme inducers | - | No explicit equation (Unadjusted) | 20 | - | - | - | 1.71 | - | High | - | - |
| 349 |  | Ini-cl | USA | DVT, PE, AF | 1.9–3.2 | 2181 | 88 | - | 12 | - | 58 | 66 ± 15 |  |  | No explicit equation (Race Adjusted) | 22 | - | - | - | 1.7 | - | High | - | - |
| 350 |  | Ini-cl | USA | DVT, PE, AF | 1.9–3.2 | 2181 | 88 | - | 12 | - | 58 | 66 ± 15 |  |  | No explicit equation (% Ancestry Adjusted) | 24 | - | - | - | 1.64 | - | High | - | - |
| 351 |  | Ini-cl | USA | DVT, PE, AF | 1.9–3.2 | 1928 | 100 | - | - | - | 59 | 66 ± 15 |  | - | No explicit equation (Whites only) | 20 | - | - | - | 1.67 | - | High | - | - |
| 352 |  | Ini-cl | USA | DVT, PE, AF | 1.9–3.2 | 253 | - | - | 100 | - | 50 | 60 ± 16 |  | - | No explicit equation (Blacks only) | 22 | - | - | - | 1.91 | - | High | - | - |

**Table S5. Continued**

| **#** | **Algorithm** | **Sub-type** | **Country** | **Indication** | **Target INR** | **N** | **Ethnicity (%)^a^** | | | | **Male %** | **Age, yrs (mean ± SD or median, IQR)** | **Clinical parameters** | **Genetic parameters** | **Equation^b^** | **R2^c^** | | | | **MAE (mg/d)** | **Other** | **ROB** | **# EV** | **# CUA** |
| --- | --- | --- | --- | --- | --- | --- | --- | --- | --- | --- | --- | --- | --- | --- | --- | --- | --- | --- | --- | --- | --- | --- | --- | --- |
|  |  |  |  |  |  |  | **W** | **A** | **B** | **M** |  |  |  |  |  | **All** | **Cl** | **VKORC1** | **CYP2C9** |  |  |  |  |  |
| 353  -  356   - 356 | Tao 2019^195^ | Ini-cl | China | - | 2.0–3.0 | 229 | - | 100 | - | - | 58 (of 247) | Age categorized | Combinations of age, height, weight, gender, amio, drinking, ALT, serum creatinine | - | No explicit equation (genetic programming) | 30-33 | - | - | - | - | Ideal dose = 45-47%  IVC (n=60): R2=33-37%, ideal dose = 41-42% | High | - | - |
| 357  -  360 |  | Ini-cl | China | - | 2.0–3.0 | 229 | - | 100 | - | - | 58 (of 247) | Age categorized | Combinations of age, height, weight, gender, amio, drinking, ALT, serum creatinine | - | No explicit equation (ε-support vector regression) | 25-26 | - | - | - | - | Ideal dose = 47-50%  IVC (n=60): R2=17-20%, ideal dose = 40-42% | High | - | - |
| 361  -  364 |  | Ini-cl | China | - | 2.0–3.0 | 229 | - | 100 | - | - | 58 (of 247) | Age categorized | Combinations of age, height, weight, gender, amio, drinking, ALT, serum creatinine | - | No explicit equation (v-support vector regression) | 23-27 | - | - | - | - | Ideal dose = 48%  IVC (n=60): R2=18-24%, ideal dose = 41-43% | High | - | - |
| 365  -  368 |  | Ini-cl | China | - | 2.0–3.0 | 229 | - | 100 | - | - | 58 (of 247) | Age categorized | Combinations of age, height, weight, gender, amio, drinking, ALT, serum creatinine | - | No explicit equation (back propagation neural network) | 40-40 | - | - | - | - | Ideal dose = 54-56%  IVC (n=60): R2=17-22%, ideal dose = 39-43% | High | - | - |
| 369  -  372 |  | Ini-cl | China | - | 2.0–3.0 | 229 | - | 100 | - | - | 58 (of 247) | Age categorized | Combinations of age, height, weight, gender, amio, drinking, ALT, serum creatinine | - | No explicit equation (general regression neural network) | 79-83 | - | - | - | - | Ideal dose = 80-87%  IVC (n=60): R2=2-9%, ideal dose = 38-43% | High | - | - |
| 373 | Asiimwe 2020 | Ini-cl | South Africa and Uganda | AF, DVT, PE, HV | 2.0–3.5 | 364 | - | - | 100 | - | 27 | 46 ± 15 | Age, weight, target INR, HIV status | - | Dose (mg/wk) = 20.2832 – 0.0656 (age) + 0.2178 (weight) + 7.3190 (Target INR 2.5 to 3.5)) + 8.7973 (HIV positive) + 3.4054 (HIV unknown) | 15 |  |  |  | 1.66 | Ideal dose =41%; unbiased MAPE=34% | Low | 1 | - |
| 374 | Li 2020^206^ | Ini-cl | China | HV | 1.5–2.5 | 9000 | - | 100 | - | - | 46 | 50 ± 11 | Age, NYHA classification, BSA, right atrial diameter, creat -inine, APPT, radiofrequency ablation, warfarin origin, anticoagulation starting time, target INR | - | No explicit equation (back propagation neural network) | - | - | - | - | 0.69 (IVC, n=3000) | IVC, MSE = 0.58, RMSE = 0.761, ideal dose = 63% | High | 1 | - |

**Table S5. Continued**

| **#** | **Algorithm** | **Sub-type** | **Country** | **Indication** | **Target INR** | **N** | **Ethnicity (%)^a^** | | | | **Male %** | **Age, yrs (mean ± SD or median, IQR)** | **Clinical parameters** | **Genetic parameters** | **Equation^b^** | **R2^c^** | | | | **MAE (mg/d)** | **Other** | **ROB** | **# EV** | **# CUA** |
| --- | --- | --- | --- | --- | --- | --- | --- | --- | --- | --- | --- | --- | --- | --- | --- | --- | --- | --- | --- | --- | --- | --- | --- | --- |
|  |  |  |  |  |  |  | **W** | **A** | **B** | **M** |  |  |  |  |  | **All** | **Cl** | **VKORC1** | **CYP2C9** |  |  |  |  |  |
| 375 | Li 2020^206^ | Ini-cl | China | HV | 1.5–2.5 | 9000 | - | 100 | - | - | 56 | 50 ± 11 | Age, NYHA classification, BSA, right atrial diameter, creat -inine, APPT, radiofrequency ablation, warfarin origin, anticoagulation starting time, target INR | - | Dose (mg/d) = 2.511 − 0.008 (age) − 0.041 (NYHA classification) + 0.820 (BSA)− 0.002 (RAD) − 0.151 (radiofrequency ablation) − 0.295 (warfarin origin) − 0.036 (starting time of anticoagulation) + 0.007 (preoperative APTT) − 0.002 (creatinine) + 0.050 (target INR) | - | - | - | - | 0.70 (IVC, n=3000) | IVC, MSE = 0.58, RMSE = 0.758, ideal dose = 63% | High | 1 | - |
| 376 | Millican 2007^207^ (incorporated in warfarindosing.com) | Rev-pgx | USA | Total knee or hip arthroplasty | 1.7–3.0 | 92 | 86 | - | 14 | - | 52 | 58 ± 16 | Doses 1 and 2, INR 3, EBL, target INR, smoking | CYP2C9*2, *3; VKORC1 haplotypes AB | Ln dose (mg/day) = 1.0138 – 2.5047 (ln[INR3]) + 0.0690 (dose 1) + 0.0385 (dose 2) + 0.2474 (ln[EBL] × ln[INR3]) – 0.1912 (CYP2C9*2) – 0.4793 (CYP2C9*3) – 0.1835 (smokes) – 0.1132 (# VKORC1 A haplotypes) + 0.2724 (target INR) | 79 | - | 1 | 17 | - | - | High | - | 1 |
| 377 |  | Rev-pgx | USA | Total knee or hip arthroplasty | 1.7–3.0 | 92 | 86 | - | 14 | - | 52 | 58 ± 16 | Doses 1 and 2, INR 3, EBL, target INR, smoking, history of liver disease | CYP2C9*2, *3 | Ln dose (mg/day) = 1.2091 – 0.1575 (CYP2C9*2) – 0.4814 (CYP2C9*3) – 0.3610 (liver disease history) + 0.1939 (smokes) + 0.1084 (target INR) – 2.5682 (ln[INR3]) + 0.0906 (dose 1) + 0.0405 (dose 2) + 0.2452 (ln[INR3] × ln[EBL]) | 79 | - | - | - | - | - | High | - | - |
| 378 | Lenzini 2008^208^ (incorporated in warfarindosing.com) | Rev-pgx | USA | Total knee or hip arthroplasty | 1.5–2.7  (for most) | 86 | 86 | - | 13 | 1 | 49 | 60 ± 14 | Doses 1-3, smoking, INR 4, EBL, liver disease | CYP2C9*2, *3 | Ln dose (mg/day) = 1.403 + 0.082 (dose 1) + 0.037 (dose 2) + 0.037 (dose 3) − 0.130 (CYP2C9*2) + 0.199 (smokes) − 1.989 (ln[INR4]) + 0.140 (ln[EBL] × ln[INR4]) − 0.385 (CYP2C9*3) − 0.463 (liver disease) | 82 | - | - | - | - | - | High | 1 | 1 |
| 379 |  | Rev-pgx | USA | Total knee or hip arthroplasty | 1.5–2.7  (for most) | 232 | Y | - | Y | - | - | - | Doses 1-3, smoking, INR 4, EBL, statin | CYP2C9*2, *3; VKORC1 -1639G>A | Ln dose (mg/day) = .098 + 0.048 (dose 1) + 0.048 (dose 2) + 0.048 (dose 3) + 0.055 (ln[EBL]) − 0.145 (statin) − 0.100 (# VKORC1 A alleles) − 0.102 (CYP2C9*2) − 0.315 (CYP2C9*3) + 0.128 (smokes) − 0.888 (ln[INR4]) | 77 | - | - | - | 0.68 (median) | - | High | - | - |
| 380 | Michaud 2008^209^ | Rev-pgx | Canada | AF, HV, DVT, PE, stroke/TIA, other | 2.0–3.5 | 132 | 99 | - | - | <1 | 57 | 62 ± 13 | Age, BSA, S: R-warfarin ratio at 14h, day 4 INR | CYP2C9*2, *3; VKORC1 -1639G>A; 3730G>A | Dose (mg/day) = 3.394 − 0.046 (age) + 2.473 (BSA) − 3.174 (14h S:R-warfarin ratio) − 0.256 (CYP2C9*1*x) − 1.575 (CYP2C9*x*x) + 0.523 (VKORC1 3730 GA) + 1.889 (VKORC1 3730 AA) − 0.237 (-1639 GA/AA) + 0.196 (day 4 INR) | 51 | 28 | 21 | 3 | - | - | High | - | - |
| 381 |  | Rev-pgx | Canada | AF, HV, DVT, PE, stroke/TIA, other | 2.0–3.5 | 132 | 99 | - | - | <1 | 57 | 62 ± 13 | Age, BSA, day 4 INR/dose, day 4 INR | CYP2C9*2, *3; VKORC1 -1639G>A; 3730G>A | Dose (mg/day) = 4.438 − 0.046 (age) + 1.400 (BSA) − 3.223 (day 4 INR/dose) − 0.599 (CYP2C9*1*x) − 1.443 (CYP2C9*x *x) + 0.282 (VKORC1 3730 GA) + 1.284 (VKORC1 3730 AA) - 0.107 (-1639 GA/AA) + 0.279 (INR) | 52 | 43 | 7 | - | - | - | High | 1 | - |

**Table S5. Continued**

| **#** | **Algorithm** | **Sub-type** | **Country** | **Indication** | **Target INR** | **N** | **Ethnicity (%)^a^** | | | | **Male %** | **Age, yrs (mean ± SD or median, IQR)** | **Clinical parameters** | **Genetic parameters** | **Equation^b^** | **R2^c^** | | | | **MAE (mg/d)** | **Other** | **ROB** | **# EV** | **# CUA** |
| --- | --- | --- | --- | --- | --- | --- | --- | --- | --- | --- | --- | --- | --- | --- | --- | --- | --- | --- | --- | --- | --- | --- | --- | --- |
|  |  |  |  |  |  |  | **W** | **A** | **B** | **M** |  |  |  |  |  | **All** | **Cl** | **VKORC1** | **CYP2C9** |  |  |  |  |  |
| 382 | Lenzini 2010^210^ | Rev-pgx | USA, UK, SwedenThailand | AF, DVT or PE, HV, stroke, orthopedic, other | Target INR 2.5 | 969 | 84 | 3 | 10 | 3 | 56 | 62 ± 14 | Age, BSA, DM, stroke, amio, fluvastatin, target INR, log INR, day 2–4 doses, race | CYP2C9*2, *3; VKORC1-1639A | The algorithm developed after pooling derivation and internal validation cohorts (n=1213): Ln dose (mg/wk) = 3.10894 − 0.00767 (age) − 0.51611 (ln[INR]) − 0.23032 (VKORC1) − 0.14745 (CYP2C9*2) − 0.3077 (CYP2C9*3) + 0.24597 (BSA) + 0.26729 (target INR) − 0.09644 (Blacks) − 0.2059 (stroke) − 0.11216 (DM) − 0.1035 (amio) − 0.19275 (fluvastatin) + 0.0169 (dose−2) + 0.02018 (dose−3) + 0.01065 (dose−4) | 63 | 48 | 23 | 7 | 0.79 (median) | IVC (n=204), R2 = 58%, median AE = 0.7  IVC subset (n=105), R2 =  60%, median AE = 0.9 | High | 8 | 4 |
| 383 | Moreau 2011^88^ | Rev-pgx | France | ATE, DVT, PE | 2.0–3.0 | 115 | 100 | - | - | - | 26 | 86 ± 6 | Age, indication, INR3 | CYP2C9*2, *3 | Dose (mg/d) = 8.85 − 0.0406 (age) − 0.489 (ATE) − 1.51 (INR3 value) − 0.396 (# variant CYP2C9 alleles) | 55 | - | - | - | - | 85% within 1 mg/d | High | - | - |
| 384 | Horne 2012^211^ | Rev-pgx | Sweden, South Korea, Thailand, UK, USA | Orthopedic (total knee or hip replacement), DVT or PE, AF, stroke, HV, other | Various | 1684 | 83 | 11 | 5 | 2 | 55 | 59 ± 14 | Age, BSA, stroke, target INR, INR values, therapy day, amio, sim, Fluvastatin. inducers, treatment response index [ln(INR/effective dose)] | CYP2C9*2, *3; VKORC1 -1639A | Ln dose (mg/wk) = 2.59853 − 0.47578 (treatment response index) − 0.17132 (VKORC1) − 0.23385 (CYP2C9*3) − 0.10696 (CYP2C9*2) − 0.00549 (age) + 0.16491 (BSA) − 0.09091 (sim) − 0.251 (Fluvastatin) − 0.11994 (amio) + 0.3319 (inducers) + 0.08796 (target INR) − 0.13902 (stroke) + 0.01028 (day of Therapy) | 72 | 65 | 3 | 3 | 0.67 (median) |  | High | 3 | - |
| 385 | Ramos 2012^212^ | Rev-pgx | Puerto Rico | AF, PTE, other | 2.0–3.5 | 163 | 90 | - | 10 | - | 99 | 68 ± 9 | Age, prior DVT, PE, amio, dose-adjusted INR at day 3 | CYP2C9*2, *3, *5; VKORC1 -1639G>A | Ln dose (mg/day) = 2.602 − 0.569 (INR/dose) − 0.463 (VKORC1 AA) − 0.153 (VKORC1 GA) − 0.174 (CYP2C9) − 0.272 (PE) − 0.276 (amio) − 0.0086 (age) | 68 | 34 | 25 | 10 | 0.79 | MAPE = 18%, SE of estimate = 0.23, MSE = 0.054 | High | 1 | - |
| 386 | Xu 2012^22^ | Rev-pgx | China | AF, DVT, PE, HV, CMP, other | 1.6–3.0 | 207 | - | 100 | - | - | 54 | 57 ± 16 | Age, BSA, target INR, day 4 INR | CYP2C9*3, VKORC1 -1639G>A, CYP4F2*3 | √dose (mg/d) = 1.66 − 0.004 (age) − 0.13 (target INR) − 0.34 (Ln[day 4 INR]) + 0.27 (BSA) − 0.92 (CYP2C9*3*3) − 0.22 (CYP2C9*1*3) + 0.16 (VKORC1 AG) + 0.50 (VKORC1 GG) + 0.25 (CYP4F2*3*3) | 54 | - | - | - | - | IVC (n=103) MAE= 0.66, R2 = 44% Entire cohort (n=310), ideal dose = 60% | High | - | - |
| 387 | Bosch 2014^134^ | Rev-pgx | Puerto Rico | AF, DVT, PE | 2.0–3.5 | 131 | - | - | - | 100 | 100 | 68 ± 9 (of 138) | Age, day 1-3 doses, day 4 INR4, admixture index, amio | CYP2C9*2, *3, *4, *6; VKORC1 -1639G>A | Ln dose (mg/d) = 1.56 − 0.00546 (age) + 0.0671 (day 1 dose) − 0.0478 (day 2 dose) + 0.156 (day 3 dose) − 0.323 (Ln[INR4]) − 0.146 (# CYP2C9 variant alleles) − 0.147 (VKORC1 AA) − 0.333 (VKORC1 GA) – 0.0593 (Taino) – 0.111 (African) – 0.108 (Mixed) − 0.132 (amio) | 77 |  | 5 | 3 | 0.63 | 72% within ideal dose, MSE =0.04 | High | - | - |

**Table S5. Continued**

| **#** | **Algorithm** | **Sub-type** | **Country** | **Indication** | **Target INR** | **N** | **Ethnicity (%)^a^** | | | | **Male %** | **Age, yrs (mean ± SD or median, IQR)** | **Clinical parameters** | **Genetic parameters** | **Equation^b^** | **R2^c^** | | | | **MAE (mg/d)** | **Other** | **ROB** | **# EV** | **# CUA** |
| --- | --- | --- | --- | --- | --- | --- | --- | --- | --- | --- | --- | --- | --- | --- | --- | --- | --- | --- | --- | --- | --- | --- | --- | --- |
|  |  |  |  |  |  |  | **W** | **A** | **B** | **M** |  |  |  |  |  | **All** | **Cl** | **VKORC1** | **CYP2C9** |  |  |  |  |  |
| 388 | Duconge 2016^213^ | Rev-pgx | Puerto Rico | AF, DVT, PE and other | 2.0–3.5 | 255 | 89 | - | 11 | - | 99 | 68 ± 10 | Age, day 1 dose, target INR, day 3 dose-adjusted INR, amio, admixture index | CYP2C9*2, *3, *8; VKORC1 -1639G>A; CYP4F2*3; NQO1*2 | Dose (mg day) = 3.2463 − 0.0181 (age) + 0.4246 (day 1 dose) + 0.5157 (target INR) − 1.419 (INR/day 3 dose) − 0.2450 (CYP2C9*2) − 0.5442 (CYP2C9*3) − 1.00 (CYP2C9*8) − 0.3793 (VKORC1 GA) − 0.8958 (VKORC1 AA) + 0.5602 (CYP4F2*3) + 0.3457 (NQO1*2) − 0.7948 (amio) − 0.0932 (admixture) | 70 | 60 | 2 | 5 | 0.72 | Mean SE of estimate = 0.99, MAPE, ideal dose in dose categories | High | 1 | - |
| 389-  392 | Byrne 2000^214^ | Rev-cl | Ireland | AF, other | - | 103 | Y | - | - | - | - | mean 57 | From age, weight, height, gender, marital status, reason for anticoagulation, target INR range, measured INR, previous and subsequent dose of warfarin, liver function tests, duration of therapy, number of hours since last warfarin dose, number of adverse drug reactions, concomitant medication, smoking, alcohol and compliance) | - | 4 models based on 17 and 23 predictors (2 artificial neural networks and 2 ensembles, each comprising 5 neural nets) | - | - | - | - | - | Within 6.1% of expert clinician doses | High | - | - |
| 393 | Solomon 2004^215^ | Rev-cl | Israel | - | Various | 108 | - | - | - | 100 | 58 | 58 ± 16 | Age, amio, total loading dose divided by INR | - | Dose (mg/d) = 3.26 − 0.31 (amio) + 0.28 (total loading dose/INR at end of loading) − 0.032 (age). | - | - | - | - | - | IVC (n=40), r = 0.800 | High | 2 | - |
| 394 |  | Rev-cl | Israel | - | Various | 108 | - | - | - | 100 | 58 | 58 ± 16 |  | - | No explicit equation (artificial neural network) | - | - | - | - | - | IVC (n=40), r = 0.823 | High | - | - |

**Table S5. Continued**

| **#** | **Algorithm** | **Sub-type** | **Country** | **Indication** | **Target INR** | **N** | **Ethnicity (%)^a^** | | | | **Male %** | **Age, yrs (mean ± SD or median, IQR)** | **Clinical parameters** | **Genetic parameters** | **Equation^b^** | **R2^c^** | | | | **MAE (mg/d)** | **Other** | **ROB** | **# EV** | **# CUA** |
| --- | --- | --- | --- | --- | --- | --- | --- | --- | --- | --- | --- | --- | --- | --- | --- | --- | --- | --- | --- | --- | --- | --- | --- | --- |
|  |  |  |  |  |  |  | **W** | **A** | **B** | **M** |  |  |  |  |  | **All** | **Cl** | **VKORC1** | **CYP2C9** |  |  |  |  |  |
| 395 | Lenzini 2007^216^ (incorporated at warfarindosing.com) | Rev-cl | USA | Total knee or hip arthroplasty | 1.7–3.0 | 271 | 85 | <1 | 13 | <1 | 45 | 60 ± 14 | Dose 1, INR after dose 2, EBL, statin | - | Ln dose (mg/d) = 1.312 − 2.861 (Ln[INR2]) + 0.257 (Ln[EBL] × Ln[INR2]) − 0.169 (statin) + 0.111 (dose 1) | 32 | - | - | - | - | - | High | 1 | 1 |
| 396 |  | Rev-cl | USA | Total knee or hip arthroplasty | 1.7–3.0 | 271 | 85 | <1 | 13 | <1 | 45 | 60 ± 14 | Doses 1-2, INR after doses 2-3, EBL, statin | - | Ln dose (mg/d) = 1.453 − 1.657 (ln[INR3]) + 0.093 (Ln[EBL] × ln[INR3]) − 0.162 (statin) + 0.070 (dose 1) + 0.061 (dose 2) | 53 | - | - | - | - | - | High | 1 | - |
| 397 | Lenzini 2008^208^ (warfarindosing.com) | Rev-cl | USA | Total knee or hip arthroplasty | 1.5–2.7 (for most) | 531 | Y | - | Y | - | - | - | Doses 1-3, smoking, INR 5, EBL, statin | - | Ln dose (mg/d) = 0.927 + 0.035 (Ln[EBL]) + 0.062 (dose 1) − 0.08830 (statin) + 0.062 (dose 2) + 0.062 (dose 3) − 1.029 (Ln[INR4]) + 0.076 (smokes) | 61 | - | - | - | 0.74 (median) | - | High | 1 | 1 |
| 398 | Lenzini 2010^210^ | Rev-cl | USA, UK, SwedenThailand | AF, DVT or PE, HV, stroke, orthopedic, others | Target INR 2.5 | 969 | 84 | 3 | 10 | 3 | 56 | 62 ± 14 | Age, BSA, DM, stroke, amio, Fluvastatin, target INR, log INR, day 2–4 doses, race | - | The algorithm developed after pooling derivation and internal validation cohorts (n=1213): Ln dose (mg/wk) = 2.81602 − 0.76679 (Ln[INR]) − 0.0059 (age) + 0.27815 (target INR) − 0.16759 (DM) + 0.17675 (BSA) − 0.22844 (stroke) − 0.25487 (Fluvastatin) + 0.07123 (Blacks) − 0.11137 (amio) + 0.03471 (dose 2) + 0.03047 (dose 3) + 0.01929 (dose 4) | 48 | - | - | - | 1.0 (median) | IVC (n=204), R2 = 43%, median AE = 0.87;  IVC subset (n=105), R2 = 44%, median AE = 1.06 | High | 4 | 1 |
| 399 | Moreau 2011^88^ | Rev-cl | France | ATE, DVT, PE | 2.0–3.0 | 115 | 100 | - | - | - | 26 | 86 ± 6 | Age, indication, INR3 | - | Dose (mg/d) = 9.61 – 0.0410 (age) – 0.482 (ATE) − 1.56 (INR3) | 52 | - | - | - | - | 87% within 1 mg/d | High | 1 | - |
| 400 | Horne 2012^211^ | Rev-cl | Sweden, South Korea, Thailand, UK, USA | Orthopedic, DVT/ PE, AF, stroke, HV, other | Various | 1684 | 83 | 11 | 5 | 2 | 55 | 59 ± 14 | Age, BSA, stroke, target INR, day of therapy, amio, sim, Fluvastatin inducers, treatment response index | - | Ln dose (mg/week) = 2.19023 − 0.66327 (treatment response index) − 0.00379 (age) + 0.1095 (BSA) − 0.06548 (sim) − 0.2809 (Fluvastatin) − 0.08761 (amio) + 0.2612 (inducer) + 0.04189 (target INR) − 0.13717 (stroke) + 0.01292 (day of therapy) | 65 | - | - | - | 0.73 (median) | - | High | 2 | - |
| 401 | Ramos 2012^212^ | Rev-cl | Puerto Rico | AF, PTE, other | 2.0–3.5 | 163 | 90 | - | 10 | - | 99 | 69 ± 9 | Age, prior DVT/ PE, amio, dose-adjusted INR at day 3 | - | Ln dose (mg/day) = 2.602 − 0.569 (INR/dose) − 0.272 (PE) − 0.276 (amio) − 0.0086 (age) | - | - | - | - | 1.22 | - | High | - | - |

**Table S5. Continued**

| **#** | **Algorithm** | **Sub-type** | **Country** | **Indication** | **Target INR** | **N** | **Ethnicity (%)^a^** | | | | **Male %** | **Age, yrs (mean ± SD or median, IQR)** | **Clinical parameters** | **Genetic parameters** | **Equation^b^** | **R2^c^** | | | | **MAE (mg/d)** | **Other** | **ROB** | **# EV** | **# CUA** |
| --- | --- | --- | --- | --- | --- | --- | --- | --- | --- | --- | --- | --- | --- | --- | --- | --- | --- | --- | --- | --- | --- | --- | --- | --- |
|  |  |  |  |  |  |  | **W** | **A** | **B** | **M** |  |  |  |  |  | **All** | **Cl** | **VKORC1** | **CYP2C9** |  |  |  |  |  |
| 402  -  405 | Zhou 2014^217^ | Rev-cl | China | HV | 1.5–2.5 | 820 | - | 100 | - | - | 43 | 49 ± 11 | Age, BSA, weight, HV, embolism history, anticoagulant drugs, operation history, urea nitrogen, creatinine, ALT, AST, starting dose, NYHA classification, left atrial appendage occlusion method, albumin | - | 4 models with 8, 11, 16 and 19 parameters (generalized linear model) | - | - | - | - | - | MPE = 0.01-0.60 mg/d; IVC (n=273) 45-66% with ideal dose | High | - | - |
| 406  -  409 |  | Rev-cl | China | HV | 1.5–2.5 | 820 | - | 100 | - | - | 43 | 49 ± 11 |  | - | 4 models with 8, 11, 16 and 19 parameters (artificial neural network) | - | - | - | - | - | MPE = 0.01-0.03 mg/d; IVC (n=273) 66-69% with ideal dose | High | - | - |
| 410 | Duconge 2016^213^ | Rev-cl | Puerto Rico | AF, DVT, PE, other | 2.0–3.5 | 255 | 89 | - | 11 | - | >99 | 68 ± 10 | Age, day 1 dose, target INR, day 3 dose-adjusted INR, amio | - | As Rev-pgx equation but excluding genotypes and admixture | 60 | - | - | - | 0.99 | Mean SE = 1.26, MAPE, ideal dose in dose categories | High | 1 | - |
| 411 | Li 2018^19^ | Rev-cl | China | HV | 1.5–2.5 | 10673 | - | 100 | - | - | 46 | 50 ± 11 | Age, ejection fraction, left ventricular diastolic diameter, operation history, albumin, urea nitrogen, creatinine, preoperative APTT, first anticoagulant timing, warfarin origin, height, weight | - | No explicit equation (back propagation neural network) | - | - | - | - | - | IVC (n=3558), MAE=0.38, RMSE=0.66, ideal dose = 59% | High | 1 | - |

**Table S5. Continued**

| **#** | **Algorithm** | **Sub-type** | **Country** | **Indication** | **Target INR** | **N** | **Ethnicity (%)^a^** | | | | **Male %** | **Age, yrs (mean ± SD or median, IQR)** | **Clinical parameters** | **Genetic parameters** | **Equation^b^** | **R2^c^** | | | | **MAE (mg/d)** | **Other** | **ROB** | **# EV** | **# CUA** |
| --- | --- | --- | --- | --- | --- | --- | --- | --- | --- | --- | --- | --- | --- | --- | --- | --- | --- | --- | --- | --- | --- | --- | --- | --- |
|  |  |  |  |  |  |  | **W** | **A** | **B** | **M** |  |  |  |  |  | **All** | **Cl** | **VKORC1** | **CYP2C9** |  |  |  |  |  |
| 412 | Sharabiani 2018^218^ | Rev-cl | USA | AF, DVT, PE, TKA/THA, HV, CVA, other | 1.8–3.5 | ~68 | 35 | 3 | 53 | 10 | 45 (of 150) | 54 ± 17 (for 150) | Age, weight, gender, IDP, indication, race, smoking, sim, lovastatin, atorvastatin, HTN, albumin, AST, asthma, alcohol, DM, dyslipidemia, creatinine | - | Dose (mg/d) = 0.396 (lovastatin) + 0.309 (CVA) + 0.293 (other race) + 0.268 (Hispanic) + 0.21 (sim) − 0.172 (PE) + 0.16 (ex-smoker) + 0.159 (female) − 0.153 (White) + 0.12 (atorvastatin) + 0.105 (IDP) − 0.089 (no HTN) − 0.073 + 0.069 (album in) − 0.064 (no Asthma) − 0.052 (DVT) − 0.036 (other indication) − 0.031 (No alcoh ol) + 0.022 (no DM) + 0.009 (no dyslipide -mia) − 0.003 (weight) − 0.001 (age) + 0.001 (creatinine clearance) + 0.001 (AST) | - | - | - | - | - | IVC (n~45), RMSE=2.38 | High | - | - |
| 413 | Tao 2018^219^ | Rev-cl | China | HV | 1.5–2.5  (for most) | 9000 | - | 100 | - | - | 46 | 50 ± 11 | Age, NYHA classification, BSA, right atrial diameter), creatinine, APPT, radiofrequency ablation, warfarin origin, anticoagulation starting time | - | No explicit equation (adaptive neural-fuzzy inference system) | - | - | - | - | - | IVC (n=3000), MAE = 0.58, MSE = 0.69, ideal dose = 64%, r = 0.313 | High | 1 | - |
| 414 |  | Rev-cl | China | HV | 1.5–2.5  (for most) | 3192 (subset of the above) | - | 100 | - | - | 45 | 50 ± 11 |  | - | No explicit equation (adaptive neural-fuzzy inference system) | - | - | - | - | - | IVC (n=3000), MAE = 0.62, MSE = 0.74, ideal dose = 60%, r = 0.309 | High | 1 | - |
| 415 | Lubetsky 1992^220^ | Clinical | Israel | Angina, DVT, PE, ATE, HV, AF | 2.0–3.0 | 18 | - | - | - | 100 | 67 | 59 ± 16 | Age, gender, weight, smoki ng, interacting drugs, INR and dose feedback | - | Computer program (pharmacokinetic-pharmacodynamic model) | Up to 58 | - | - | - | - | Prediction error 0.95-2.84 mg/d | High | - | - |
| 416 | Hamberg 2007^221^ | Pgx | Italy | AF, DVT, HV, other | 2.0–3.0 | 150 | 100 | - | - | - | 66 | 71 (range 22–87) | Age | CYP2C9*2, *3; VKORC1-1639G>A | Table provided (population pharmaco kinetic-pharmacodynamic model) | - | - | - | - | - | VPCs for six typical individuals | High | - | - |
| 417  -  422 | Caraco 2008^222^ | Pgx | Israel | - | - | - | - | - | - | Y | - | - | S-warfarin clearance, amio | CYP2C9*2*3 | Six algorithms. The first (*1*1) had doses on average 25% higher than a computerized INR-based system (Ageno 2000) or a 27% increase in S-warfarin clearance. The other five (for *1*2, *1*3, *2*2, *2*2 and *3*3) had lower doses that respectively corresponded to S-warfarin decreases of 20, 40, 50, 60, and 85%. Doses in patients taking amiod- arone were on average reduced by 25%. | - | - | - | - | - | - | Unclear | - | 1 |

**Table S5. Continued**

| **#** | **Algorithm** | **Sub-type** | **Country** | **Indication** | **Target INR** | **N** | **Ethnicity (%)^a^** | | | | **Male %** | **Age, yrs (mean ± SD or median, IQR)** | **Clinical parameters** | **Genetic parameters** | **Equation^b^** | **R2^c^** | | | | **MAE (mg/d)** | **Other** | **ROB** | **# EV** | **# CUA** |
| --- | --- | --- | --- | --- | --- | --- | --- | --- | --- | --- | --- | --- | --- | --- | --- | --- | --- | --- | --- | --- | --- | --- | --- | --- |
|  |  |  |  |  |  |  | **W** | **A** | **B** | **M** |  |  |  |  |  | **All** | **Cl** | **VKORC1** | **CYP2C9** |  |  |  |  |  |
| 423 | Linder 2009^223^ | Pgx | USA | - | 2.0–3.0 | 137 | Y | - | - | - | 58 | 75 (67–80) | Age, gender, weight | CYP2C9*2, *3; VKORC1 -1639G>A | Personalized medicine interface (PerMIT) tool | - | - | - | - | - | 2 case studies | High | - | 1 |
| 424 | Hamberg 2010^224^ (updates Hamberg 2007) | Pgx | Italy and  Sweden | AF, DVT, PE, HV, other | 2.0–3.0 | 1426 (final parameters | Y | - | - | - | 64 | 68 (range 18–92) | Age | CYP2C9*2, *3; VKORC1 -1639G>A | Dosing table provided (kinetic-pharmacodynamic model) | - | - | - | - | - | VPCs | High | 1 | - |
| 425 | Gong 2011^86^ | Pgx | Canada | AF, DVT, PE, other | 2.0–3.0 | 167 | 95 | 2 | 2 | 1 | 44 | 60 ± 17 | Age, gender, weight, height, amio, days 3-8 INRs | CYP2C9*2, *3; VKORC1 -1639G>A; CYP4F2*3 | Warfarin Regimen using A  Pharmacogenetics-guided Initiation Dosing (WRAPID) automated dose calculator (pharmacokinetic-pharmacodynamic model) | 70 | - | - | - | 1.21 | - | High | - | - |
| 426 | Perlstein 2012^225^ | Pgx | USA | Non-Orthopedic | 1.8–3.2 | 147 | 85 | - | - | - | 57 | 58 ± 17 | Age, height | CYP2C9*2, *3; VKORC1 haplotype | Dosing table provided (pharmacokinetic-pharmacodynamic model) | - | - | - | - | In figure | - | High | - | - |
| 427 |  | Pgx | USA | Non-Orthopedic | 1.8–3.2 | 79 | 91 | - | - | - | 52 | 57 ± 16 | - |  |  | - | - | - | - | In figure | - | High | - | - |
| 428 | Lala 2013^226^ (scales Perlstein 2012)  (***Children***) | Pgx | USA | HV, FP, Kawasaki disease, CMP | 1.5–3.5 | 26 | 88 | - | 8 | 4 | 61 | Mean 4 (range <1–18) | Weight | CYP2C9*2, *3, *5; VKORC1 -1639A | Dosing table provided (scaled pharmacokinetic-pharmacodynamic model) | - | - | - | - | - | VPCs | High | 2 | - |
| 429 | Lu 2013^227^ | Pgx | China | - | 1.5–2.8 | 183 | - | 100 | - | - | 42 (for 197) | 47 (range 18–76) for 197 | Age, gender, weight | CYP2C9*3; VKORC1 -1639G>A | In vitro-in vivo extrapolation with a pharmacokinetic-pharmacodynamic model | 42 | - | - | - | - | VPCs | High | - | - |
| 430 | Hamberg 2013^109^ (updates Hamberg 2007, 2010)  (***Children***) | Pgx | See Hamberg 2007 and 2010 – uses allometric scaling i.e. no data from warfarin-treated children was used during development | | - | - | Y | - | - | - | See Hamberg 2007 and 2010 | | Age, weight, baseline and target INR, previous doses and INR observations | CYP2C9*2, *3; VKORC1 –1639G>A | A set of differential equations (kinetic-pharmacodynamic model) | - | - | - | - | - | VPCs | High | 4 | 2 |
| 431 | Hamberg 2014^228^ (updates Hamberg 2010; 2013) | Pgx | Canada, Sweden, UK | TP, HV, dilated CMP, coronary aneurysm, Other | 2.0–3.5 (for 93% of participants) | 163 | 77 | 7 | 5 | 11 | 64 | 6 (range <1-19) | Age, weight, baseline and target INR, and time since thera py initiation | CYP2C9*2, *3; VKORC1 -1639G>A | Dosing table for 54 typical children aged 2, 8 and 14 years old (kinetic-pharmacodynamic model) | - | - | - | - | - | VPCs | High | - | - |
| 432 | Arwood 2017^229^ (based on Hamberg 2010) | Pgx | USA | VTE, AF, atrial or ventricular thrombus, stroke, other | 2.0–3.0 | 257 | 33 | 2 | 55 | 10 | 51 | 51 ± 16 | Age | CYP2C9*2, *3, *8; VKORC1 -1639G>A | Dosing table/nomogram (kinetic-pharmacodynamic model) | - | - | - | - | - | VPCs | High | - | - |

**Table S5. Continued**

| **#** | **Algorithm** | **Sub-type** | **Country** | **Indication** | **Target INR** | **N** | **Ethnicity (%)^a^** | | | | **Male %** | **Age, yrs (mean ± SD or median, IQR)** | **Clinical parameters** | **Genetic parameters** | **Equation^b^** | **R2^c^** | | | | **MAE (mg/d)** | **Other** | **ROB** | **# EV** | **# CUA** |
| --- | --- | --- | --- | --- | --- | --- | --- | --- | --- | --- | --- | --- | --- | --- | --- | --- | --- | --- | --- | --- | --- | --- | --- | --- |
|  |  |  |  |  |  |  | **W** | **A** | **B** | **M** |  |  |  |  |  | **All** | **Cl** | **VKORC1** | **CYP2C9** |  |  |  |  |  |
| 433 | Zhu 2017^180^ | Pgx | China | HV | 1.8–2.5 | 242 | - | Y | - | - | 42 | 55 ± 12 | BSA, gender, clearance; distribution volume, S- and R- warfarin concentrations | CYP2C9*3 | Population pharmacokinetic-pharmacodynamic model | - | - | - | - | - | VPCs | High | - | - |

^a^W, A, B and M stand for White, Asian, Black, and Mixed/Other respectively. Populations with multiple ethnicities (e.g. Turkish, Egyptians, Sudanese, Puerto Ricans – unless stated) were classified under Mixed/Other. Hispanics, if present and not classified as Black, included with Whites. Y implies that the population is mainly a given category (used where the exact proportion was not provided). ^b^Refer to original/referenced publications for units and explanations on how to code for the variables. ^c^Adjusted R^2^ used if reported. Abbreviations: #, number of; ABCB1, ATP Binding Cassette Subfamily B Member 1; ACE, angiotensin converting enzyme; AF, atrial fibrillation (and/or flutter); ALT, alanine aminotransferase; AMI, acute myocardial infarction; amio, amiodarone; APOE, Apolipoprotein E; APPT = activated partial thromboplastin time; ARB, angiotensin II receptor blocker; AST, aspartate aminotransferase; ATE, arterial thromboembolic disease; BMI, body mass index; BSA, body surface area; CAD, coronary artery disease; CALU, calumenin; CAR, constitutive androstane receptor; CCB, calcium channel blockers; CES2, Carboxylesterase 2; CHF, congestive heart failure; CKD, chronic kidney disease; Cl, clinical; CMP, cardiomyopathy; COPD, Chronic obstructive pulmonary disease; CUA, clinical utility assessment; CVA, cerebrovascular accident; CYP2C9, cytochrome P450, family 2, subfamily C, polypeptide 9; CYP4F2, cytochrome P450, family 4, subfamily F, polypeptide 2; DDHD1, DDHD Domain Containing 1; DM, diabetes mellitus; DVT, deep vein thrombosis; EBL, estimated blood loss; eCrCl, estimated creatinine clearance; eGFR, estimated glomerular filtration rate; GGCX, γ-glutamyl carboxylase; EPHX1, epoxide hydrolase 1; EV, external validations; FMO2, flavin containing dimethylaniline monoxygenase 2; FP, Fontan procedure; HIV, human immunodeficiency virus; HNF4a = hepatocyte nuclear factor 4a; HV, heart valve; HTN, hypertension; IDP, Initial Dose Prescribed by the Physician; IHD, ischaemic heart disease; Ini, dose-initiation; INR, international normalized ratio; IVC, internal validation cohort; IWPC, international warfarin pharmacogenetics consortium; LASSO, least absolute shrinkage and selection operator; LOOCV, leave-out-one cross validation; Ln, natural logarithm; M%PE, mean percentage prediction error; MAE, mean absolute error; MAPE, mean absolute percentage error; MDR1, multidrug resistance 1; MPE, mean prediction error; MTHFR, Methylene tetrahydrofolate reductase; MYC, MYC Proto-Oncogene; N, sample size; NEDD4, NEDD4 E3 Ubiquitin Protein Ligase; NQO1, NAD(P)H dehydrogenase [quinone] 1; NYHA, New York Heart Association; PE, pulmonary embolism; Pgx, pharmacogenetic; POR = P450 oxidoreductase; PXR, pregnane X receptor; PZ = Protein Z; R^2^, coefficient of determination; Rev, dose-revision; RHD, rheumatic heart disease; RMSE, root mean square error; RMSPE, root mean square percentage error; RPA, regular physical activity; sim = simvastatin; SLCO1B3, solute carrier organic anion transporter family member 1B3; TIA, transient ischaemic attack; TE, thromboembolism; TSH, thyroid stimulating hormone; UGT1A1, UDP glucuronosyltransferase family 1 member A1; VDR, vitamin D receptor; VKORC1, vitamin K epoxide reductase complex subunit 1; VKORC1L1, vitamin K epoxide reductase complex 1 like protein 1; VPC, visual predictive checks; WBC, white blood cell). Dashes imply information not available or not applicable while Underscored R^2^ values are reported as univariate analyses.

**Table S6. Quality assessment (algorithm development)**

| **#** | **Algorithm** | **Sub-type** | **N** | **CPP** | **PCV** | **Predictor selection** | **Dose transformation** | **Reason** | **Risk groups** | **Cut-offs** | **Validation (as report**  **ed in study)** | **Performance measures reported** | **Excluded participants** | **Missing data handling** | **PROBAST signaling questions** | | | | | | | | **ROB** | **Stable dose defined** | **Other key ROB concerns**  **(domains 1-3)** |
| --- | --- | --- | --- | --- | --- | --- | --- | --- | --- | --- | --- | --- | --- | --- | --- | --- | --- | --- | --- | --- | --- | --- | --- | --- | --- |
|  |  |  |  |  |  |  |  |  |  |  |  |  |  |  | **4.1** | **4.2** | **4.3** | **4.4** | **4.5** | **4.7** | **4.8** | **4.9** |  |  |  |
| 1 | Tabrizi 2002^29^ | MLR | 153 | ≥8 | 10_20 | Backward stepwise (univariate first) | Log | Normalize dose | None | NA | None | R2 (log scale) | None | NI | NC | NI | PY | NI | N | N | N | NI | High | Y | - |
| 2 | Gage 2004^26^ | MLR | 369 | ≥19 | 10_20 | Stepwise (univariate first) | Log | Normalize dose | None | NA | None | R2; MAE; within 1mg/d | None | Single imputation | NC | NI | PY | N | N | Y | N | Y | High | Y | - |
| 3 | Hillman 2004^30^ | MLR | 453 | <20 | >20 | Modiﬁed step-down approach | Log | Clinical consideration (proportional scale); variance and normality | None | NA | None | R2 (log scale) | With rare genotypes; on CYP2C9 inducers | NI | Y | NI | N | NI | PY | N | N | NI | High | N | - |
| 4 | Kamali 2004^31^ | MLR | 119 | ~8 | 10_20 | NI | None | NA | None | NA | None | R2 | With rare genotypes; missing data | Excluded | NC | NI | N | N | PN | N | N | NI | High | Y | - |
| 5 | Herman 2005^32^ | MLR | 187 | <10 | >20 | Forward stepwise (univariate first) | Log | Normalize dose | None | NA | None | R2 | Missing data or technicalities | Excluded | Y | NI | N | N | N | N | N | Y | High | Y | - |
| 6 | Sconce 2005^33^ | MLR | 297 | ~12 | >20 | Stepwise (univariate first) | Square root | Normalize dose | None | NA | External | R2 | None | NI | Y | NI | PY | NI | N | N | N | NI | High | Y | - |
| 7 | Wadelius 2005^34^ | MLR | 201 | >30 | <10 | NI (univariate first) | None | NA | None | NA | None | R2 | None | NI (missing genotypes) | N | NI | PY | N | N | N | N | NI | High | N | - |
| 8 | Aquilante 2006^36^ | MLR | 350 | ~27 | 10_20 | Backward stepwise (univariate first) | None | NA | None | NA | None | R2 | None | Excluded | NC | NI | Y | N | N | N | N | NI | High | Y | Mean INR in predictors, vit K assessment may have some [recall] bias. |
| 9 | Carlquist 2006^37^ | MLR | 213 | ~10 | >20 | NI (univariate first) | Log | Normalize dose | None | NA | None | R2 | None | NI | Y | NI | PY | NI | N | N | N | NI | High | Y | - |
| 10 | Herman 2006^39^ | MLR | 165 | ~27 | <10 | Stepwise (univariate first) | Log | Normalize dose | None | NA | None | R2 | None | NI | N | NI | PY | NI | N | N | N | Y | High | Y | - |
| 11 | Takahashi 2006^40^ | MLR | 179 | ~10 | 10_20 | Stepwise (univariate first) | None | NA | None | NA | None | R2 | Blacks with no genotype data | Excluded | NC | NI | N | N | N | N | N | NI | High | N | - |
| 12 | Tham 2006^41^ | MLR | 107 | 11 | <10 | NI (univariate first) | Log | Normalize dose | None | NA | Random split | R2, MPE, within -1 to 0.5 mg/d | Missing data | Excluded | N | NI | N | N | N | N | N | Y | High | Y | - |
| 13 | Anderson 2007^38^ | MLR | 175 | 8 | >20 | Based on Carlquist 2006 | Log | Carlquist 2006 | None | NA | None | R2 | None | NI | Y | NI | PY | NI | Y | N | N | NI | High | Y | Some doses estimated |
| 14 | Caldwell 2007^42^ | MLR | 431 | <20 | >20 | Modified step-down approach | Log | Clinical consideration; variance and normality | None | NA | None | R2 (log scale) | Missing genotype data; outliers | Excluded | Y | NI | N | N | PY | N | N | NI | High | Y (for some) | - |
| 15 | Miao 2007^43^ | MLR | 178 | ~14 | 10_20 | NI | None | NA | None | NA | None | R2 | None | NI | NC | NI | Y | NI | PN | N | N | NI | High | Y | - |
| 16 | Rieder 2007^44^ | MLR | 186 | ~12 | 10_20 | NI (likely no selection) | Log | NI | None | NA | None | R2 | None | NI | NC | NI | PY | NI | PY | N | N | NI | High | Y | - |
| 17 |  | MLR | 186 | ~12 | 10_20 | NI (likely no selection) | Log | NI | None | NA | None | - | None | NI | NC | NI | PY | NI | PY | N | N | NI | High | Y | - |
| 18 | Zhu 2007^45^ | MLR | 65 | ≥7 | <10 | NI (univariate conducted) | Log | Normalize dose | None | NA | None | R2, SE of the dose estimate | None | NI | N | NI | PY | NI | PN | N | N | NI | High | Y | - |
| 19 | Caldwell 2008^46^ | MLR | 1051 | >1228 | <10 | Modified step-down approach (univariate first) | Log | NI | None | NA | None | R2 | Missing genotype data; outliers | Excluded | N | NI | N | N | N | N | N | NI | High | Y (for some) | - |
| 20 | Gage 2008^48^ | MLR | 1015 | ~20 | >20 | Stepwise (univariate first) | Log | Heteroscedascity and model fit | None | NA | External | R2 | None | NI | Y | NI | PY | NI | N | N | PN | NI | High | Y | - |

**Table S6. Continued**

| **#** | **Algorithm** | **Sub-type** | **N** | **CPP** | **PCV** | **Predictor selection** | **Dose transformation** | **Reason** | **Risk groups** | **Cut-offs** | **Validation (as report**  **ed in study** | **Performance measures reported^a^** | **Excluded participants** | **Missing data handling** | **PROBAST signaling questions** | | | | | | | | **ROB** | **Stable dose defined** | **Other key ROB concerns**  **(domains 1-3)** |
| --- | --- | --- | --- | --- | --- | --- | --- | --- | --- | --- | --- | --- | --- | --- | --- | --- | --- | --- | --- | --- | --- | --- | --- | --- | --- |
|  |  |  |  |  |  |  |  |  |  |  |  |  |  |  | **4.1** | **4.2** | **4.3** | **4.4** | **4.5** | **4.7** | **4.8** | **4.9** |  |  |  |
| 21 | Haug 2008^49^ | MLR | 105 | ~7 | 10_20 | Stepwise (univariate first) | None | NA | None | NA | None | R2 | None | NI | NC | NI | PY | NI | PN | N | N | Y | High | N | - |
| 22 | Kimmel 2008^50^ | MLR | 232 | ~30 | <10 | NI (univariate first) | Square root | Normalize dose | None | NA | None | R2 | None | NI | N | N | PY | NI | N | N | N | NI | High | Y | - |
| 23 |  | MLR | 232 | ~30 | <10 | NI (univariate first) | Square root | Normalize dose | None | NA | None | R2 | Rare genotypes | NI | N | N | N | NI | N | N | N | NI | High | Y | - |
| 24 | Meckley 2008^51^ | MLR | 122 | ≥20 | <10 | Backwards stepwise | None | NA | None | NA | None | R2 | Missing data | Excluded | N | NI | N | N | Y | N | N | Y | High | Y |  |
| 25 | Oner Ozgon 2008^52^ | MLR | 205 | 14 | 10_20 | Backward (univariate conducted) | Log | Heteroscedascity and model fit | None | NA | None | R2 | None | NI | NC | NI | PY | NI | NI | N | N | NI | High | Y | Subjective stability assessment |
| 26 | Perini 2008^53^ | MLR | 390 | ~40 | <10 | NI (univariate first) | Square root | Model fit and data-driven (tested several) | None | NA | None | R2, MAE, M%PE, MAPE | None | NI | N | NI | PY | NI | N | Y | N | Y | High | Y | - |
| 27 | Sasaki 2009^54^ | NONMEN; MLR | 45 | ≥5 | <10 | Based on structural model | None | NA | None | NA | 10-fold CV | R2, MPE, RMSE | None | NI | N | NI | PY | NI | PY | Y | PN | Y | High | N | - |
| 28 | Schelleman 2008^55^ | MLR | 259 | ~20 | 10_20 | Backward elimination (univariate first) | Log | Normalize dose | None | NA | LOOCV | R2, within 1 mg/d | Missing data | Excluded | NC | NI | N | N | N | N | N | Y | High | Y | - |
| 29 |  | MLR | 147 | ~20 | <10 | Backward elimination (univariate first) | Log | Normalize dose | None | NA | LOOCV | R2, within 1 mg/d | Missing data | Excluded | N | NI | N | N | N | N | N | Y | High | Y | - |
| 30 |  | MLR | 112 | ~20 | <10 | Backward elimination (univariate first) | Log | Normalize dose | None | NA | LOOCV | R2, within 1 mg/d | Missing data | Excluded | N | NI | N | N | N | N | N | Y | High | Y | - |
| 31 | Wen 2008^56^ | MLR | 108 | ≥11 | <10 | Backward | None | NA | None | NA | None | R2 | None | NI | N | NI | PY | NI | PY | N | N | NI | High | Y | - |
| 32 |  | MLR | 75 | ≥11 | <10 | Backward | None | NA | None | NA | None | R2 | None | NI | N | NI | PY | NI | PY | N | N | NI | High | Y | - |
| 33 | Wu 2008^57^ | MLR | 92 | ≥10 | <10 | NI | Log | Normalize dose | None | NA | External | R2 | Missing data | Excluded | N | NI | N | N | NI | N | N | NI | High | Y | - |
| 34 | Huang 2009^58^ | MLR | 266 | ~12 | >20 | Stepwise (univariate first) | Log | Normalize dose | None | NA | External | R2 | None | NI | Y | NI | PY | NI | N | N | N | Y | High | Y | - |
| 35 | IWPC 2009^3^ | MLR and others^b^ | 4043 | NI | >20 | NI | Square root | Normalize dose; data-driven (tested log) | Bracketing | 21, 49 | 10-fold CV; random  split | R2^c^, MAE, ideal dose, as diagnostics | None | Likely single imputation | Y | NI | PY | PN | NI | Y | Y | NI | High | Y | - |
| 36 | Kim 2009^59^ | MLR | 239 | ~24 | <10 | Stepwise (univariate first) | Square root | Normalize dose | None | NA | Random split | R2, r | None | NI | N | NI | PY | NI | N | N | N | Y | High | Y | - |
| 37 | Ohno 2009^60^ | MLR | 125 | ≥15 | <10 | Stepwise (univariate first) | None | NA | None | NA | Random split, 1000 repeats | R2, MPE, RMSE, ideal dose | None | NI | N | NI | PY | NI | N | Y | N | NI | High | Y | - |
| 38 | Sandanaraj 2009^62^ | MLR | 107 | ≥15 | <10 | NI (univariate first) | None | NA | None | NA | None | R2 | None | NI | N | NI | PY | NI | N | N | N | NI | High | Y | - |
| 39 | Suarez-Kurtz 2009^63^ | MLR | 260 | ~40 | <10 | NI (univariate first) | Square root | Perini 2008 | None | NA | Random split | R2, MAE | None | NI | N | NI | PY | NI | N | Y | N | Y | High | Y | - |
| 40 | Wadelius 2009^64^ | MLR | 1324 | >183 | <10 | NI (univariate first) | Square root | NI | None | NA | External | R2 | Missing genotype data | Excluded | N | NI | N | N | N | N | N | Y | High | Y | - |
| 41 | Yoshizawa 2009^65^ | MLR | 93 | ≥20 | <10 | NI (univariate first) | None | NA | None | NA | None | R2 | Analyzed ≠ enrolled | NI | N | NI | N | PN | N | N | N | Y | High | Y | - |
| 42 | Cen 2010^66^ | MLR | 222 | ~11 | >20 | Stepwise (univariate first) | Log | Normalize dose | None | NA | None | R2 | None | NI | Y | NI | PY | NI | PN | N | N | NI | High | Y | - |
| 43 | Cha 2010^24^ | MLR | 440 | ~28 | 10_20 | NI (univariate first) | None | NA | Bracketing | 7, 28 | None | R2 | Missing genotype data | Excluded | NC | NI | N | N | N | N | N | NI | High | Y | - |
| 44 | Du 2010 | NI | NI | NI | NI | NI | NI | NI | NI | NI | NI | R2 | NI | NI | NI | NI | NI | NI | NI | NI | NI | NI | Unclear | NI | - |
| 45 | Harada 2010^69^ | MLR | 97 | 20 | <10 | Stepwise (univariate first) | None | NA | None | NA | None | R2 | Missing data | Excluded | N | NI | N | N | N | N | N | Y | High | N | - |
| 46 | King 2010^70^ | MLR | 985 | ~14 | >20 | Based on Gage 2008 | Log (errors) | Proportionality | None | NA | None | R2 | None | NI | Y | NI | PY | NI | Y | N | N | NI | High | Y | - |

**Table S6. Continued**

| **#** | **Algorithm** | **Sub-type** | **N** | **CPP** | **PCV** | **Predictor selection** | **Dose transformation** | **Reason** | **Risk groups** | **Cut-offs** | **Validation (as report**  **ed in study** | **Performance measures reported** | **Excluded participants** | **Missing data handling** | **PROBAST signaling questions** | | | | | | | | **ROB** | **Stable dose defined** | **Other key ROB concerns**  **(domains 1-3)** |
| --- | --- | --- | --- | --- | --- | --- | --- | --- | --- | --- | --- | --- | --- | --- | --- | --- | --- | --- | --- | --- | --- | --- | --- | --- | --- |
|  |  |  |  |  |  |  |  |  |  |  |  |  |  |  | **4.1** | **4.2** | **4.3** | **4.4** | **4.5** | **4.7** | **4.8** | **4.9** |  |  |  |
| 47 | McMillin 2010^71^ | MLR | 115 | ~7 | 10_20 | NI (univariate conducted) | None | NA | None | NA | External | - | None | NI | NC | NI | PY | NI | PN | N | N | NI | High | Y | - |
| 48 | Namazi 2010^72^ | MLR | 55 | ≥8 | <10 | Backward (univariate first) | None | NA | None | NA | None | R2 | Missing data | Excluded | N | NI | N | N | N | N | N | NI | High | Y | - |
| 49 | Nowak-Gottl 2010^73^ | MLR | 34 | ≥7 | <10 | NI (univariate first) | Square root | Normalize dose | None | NA | None | R2 | Missing data | Excluded | N | NI | N | N | N | N | N | NI | High | Y | - |
| 50 | Ozer 2010^74^ | MLR | 100 | ~9 | 10_20 | NI (univariate first) | None | NA | None | NA | None | R2 | None | NI | NC | NI | PY | NI | N | N | N | NI | High | Y | - |
| 51 | Pautas 2010^75^ | MLR | 283 | ≥36 | <10 | Stepwise (univariate first) | None | NA | None | NA | None | R2, plots inspection | None | NI | N | NI | PY | NI | N | N | N | Y | High | Y | - |
| 52 | Perini 2010^76^ | MLR | 370 | ~40 | <10 | Based on Perini 2008 | Square root | Model fit and data-driven (tested several) | None | NA | None | R2, MAE, M%PE | None | NI | N | NI | PY | NI | N | Y | N | Y | High | Y | - |
| 53 | Roper 2010^77^ | MLR | 121 | 6 | >20 | Stepwise | None | NA | Bracketing  (IWPC-based) | 21, 49 | External | R2, MAE, MAPE, ideal dose | Missing data; outliers | Excluded | Y | NI | N | N | PY | Y | N | Y | High | Y | - |
| 54 | Sagreiya 2010^78^ | MLR | 95 | ~18 | <10 | Based on IWPC | Square root | Based on IWPC | None | NA | NA | R2 | Missing data | Excluded | N | NI | N | N | PY | N | N | NI | High | Y | - |
| 55 | Sangviroon 2010^79^ | MLR | 89 | >10 | <10 | Forward stepwise (univariate first) | Log | NI | NI | 17.5, 35 | None | R2, within 15% of actual | None | NI | N | NI | PY | NI | N | N | N | Y | High | Y | - |
| 56 | Takeuchi 2010^23^ | MLR | 200 | ~11 | 10_20 | Based on IWPC | Square root | Based on IWPC | Bracketing | 10.5, 31.5 | None | R2 | None | NI | NC | N | PY | NI | Y | N | N | NI | High | N | - |
| 57 | Wells 2010^80^ | MLR | 246 | ≥29 | <10 | Stepwise backward | None | NA | None | NA | None | R2, VPCs | Missing data | Excluded | N | N | N | N | NI | N | N | N | High | Y | - |
| 58 | Zhang 2010 | MLR | 101 | NI | NI | NI | NI | NI | NI | NI | NI | R2 | NI | NI | NI | NI | NI | NI | NI | NI | NI | NI | Unclear | NI | - |
| 59 | Avery 2011^81^ | MLR | 671 | NI | >20 | Based on mechanistic model | None | NA | None | NA | None | r, VPCs | Missing data | Excluded | Y | NI | N | N | NI | N | N | NI | High | N | - |
| 60 | Botton 2011^82^ | MLR | 279 | >30 | <10 | NI (univariate first) | Log | Normalize dose | None | NA | None | R2, MAE | None | NI | N | NI | PY | NI | N | Y | N | Y | High | Y | - |
| 61 |  | MLR | 279 | >30 | <10 | NI (univariate first) | Log | Normalize dose | None | NA |  | R2, MAE | None | NI | N | NI | PY | NI | N | Y | N | Y | High | Y | - |
| 62 | Cavallari 2011^83^ | MLR | 50 | >5 | <10 | Stepwise (univariate first) | Log | Normalize dose | None | NA | None | R2 | None | NI | N | NI | PY | NI | N | N | N | NI | High | Y | - |
| 63 | Cho 2011^25^ | MLR | 130 | ≥20 | <10 | Stepwise (univariate first) | Log | Normalize dose | NI | 21, 49 | External | R2 | None | NI | N | NI | PY | NI | N | N | N | NI | High | Y | - |
| 64 | Choi 2011^84^ | MLR | 564 | ~27 | >20 | NI (univariate first) | None | NA | None | NA | None | R2 | None | NI | Y | NI | PY | NI | PN | N | N | N | High | Y | 27 patients had INR >3.0 |
| 65 | Cosgun 2011^85^ | RFR | 290 | >200 | <10 | Top SNP sets (univariate-based) | None | NA | None | NA | 5-fold CV | R2 | High identity-by-state (n = 4) | Likely single imputation | N | PY | Y | PN | N | N | Y | NA | High | N | - |
| 66 |  | BRT | 290 | >200 | <10 |  | None | NA | None | NA | 5-fold CV | R2 |  |  | N | PY | Y | PN | N | N | Y | NA | High | N | - |
| 67 |  | SVR | 290 | >200 | <10 |  | None | NA | None | NA | 5-fold CV | R2 |  |  | N | PY | Y | PN | N | N | Y | NA | High | N | - |
| 68 | Gong 2011^86^ | NONMEN; MLR | 167 | ~10 | 10_20 | NI | None | NA | None | NA | None | R2, MAE | Missing/wrong data | Excluded | NC | NI | N | N | NI | Y | N | NI | High | Y | - |
| 69 | Moon 2011^87^ | MLR | 58 | >10 | <10 | Stepwise (univariate first) | Log | NI | None | NA | None | R2 | None | NI | N | NI | PY | NI | N | N | N | NI | High | Y | - |
| 70 | Moreau 2011^88^ | MLR | 115 | ≥18 | <10 | Stepwise (univariate first) | None | NA | None | NA | None | R2, within 1 mg/d, plots inspection | None | NI | N | NI | PY | NI | N | N | N | Y | High | Y | - |
| 71 | Perera 2011^89^ | MLR | 330 | >40 | <10 | Forward stepwise (univariate first) | Log | Normalize dose | None | NA | None | R2 | Missing genotype data | Excluded | N | NI | N | N | N | N | N | NI | High | Y | - |
| 72 | Sarapakdi 2011 | NI | 197 | NI | NI | NI | NI | NI | NI | NI | NI | R2 | NI | NI | NI | NI | NI | NI | NI | NI | NI | NI | Unclear | NI | - |
| 73 | Shahin 2011^92^ | MLR | 195 | ≥30 | <10 | Stepwise (univariate first) | Square root | Heteroscedascity and model fit | None | NA | None | R2 | Missing genotype data | Excluded | N | NI | N | N | N | N | N | NI | High | Y | - |
| 74 | Shrif 2011^93^ | MLR | 157 | >30 | <10 | Backward elimination (univariate first) | Log | Normalize dose | None | NA | None | R2 | Missing data | Excluded | N | NI | N | N | N | N | N | NI | High | Y | - |

**Table S6. Continued**

| **#** | **Algorithm** | **Sub-type** | **N** | **CPP** | **PCV** | **Predictor selection** | **Dose transformation** | **Reason** | **Risk groups** | **Cut-offs** | **Validation (as report**  **ed in study** | **Performance measures reported** | **Excluded participants** | **Missing data handling** | **PROBAST signaling questions** | | | | | | | | **ROB** | **Stable dose defined** | **Other key ROB concerns**  **(domains 1-3)** |
| --- | --- | --- | --- | --- | --- | --- | --- | --- | --- | --- | --- | --- | --- | --- | --- | --- | --- | --- | --- | --- | --- | --- | --- | --- | --- |
|  |  |  |  |  |  |  |  |  |  |  |  |  |  |  | **4.1** | **4.2** | **4.3** | **4.4** | **4.5** | **4.7** | **4.8** | **4.9** |  |  |  |
| 75 | Singh 2011^94^ | MLR | 76 | ~10 | <10 | NI (univariate first) | None | NA | None | NA | None | R2 | None | NI | N | NI | PY | NI | N | N | N | NI | High | Y | - |
| 76 | Suriapranata 2011^95^ | MLR | 85 | ≥26 | <10 | NI (univariate first) | Log | Normalize dose | None | NA | None | R2 | Missing data | Excluded | N | NI | N | N | N | N | N | NI | High | Y | - |
| 77 | Yang 2011^96^ | MLR | 178 | ~8 | >20 | NI | None | NA | None | NA | None | R2 | None | NI | Y | NI | Y | NI | PN | N | N | NI | High | Y | - |
| 78 | You 2011^97^ | MLR | 80 | >8 | <10 | Stepwise (univariate first) | Square root | NI | None | NA | Random split | R2, r, ideal dose, mean difference | None | NI | N | N | PY | NI | N | Y | N | NI | High | Y | - |
| 79 | Zambon 2011^21^ | MLR | 274 | ≤21 | >20 | Backward (univariate conducted) | Square root | NI | Bracketing | 25, 45 | Random split | R2, MAE, ideal dose, Bland-Altman | Missing data | Excluded | Y | NI | N | N | N | Y | N | Y | High | Y | - |
| 80 | Zhang 2011^98^ | MLR | 122 | ~20 | <10 | Backward elimination (univariate first) | Square root | Normalize dose | None | NA | None | R2 | 2 females | NI | N | NI | N | NI | N | N | N | Y | High | Y | - |
| 81 |  | MLR | 122 | ~15 | <10 | Backward elimination (univariate first) | Square root | Normalize dose | None | NA | None | R2 | 2 females | NI | N | NI | N | NI | N | N | N | Y | High | Y | - |
| 82 |  | MLR | 122 | ~7 | 10_20 | Backward elimination (univariate first) | Square root | Normalize dose | None | NA | None | R2 | 2 females | NI | NC | NI | N | NI | N | N | N | Y | High | Y | - |
| 83 | Anderson 2012^99^ | MLR | See IWPC/Gage | | >20 | NI | Square root | Based on IWPC | None | NA | None | None | None | NI | Y | NI | PY | NI | Y | N | N | NI | High | Y | - |
| 84 | Biss 2012^100^ | MLR | 120 | ≥14 | <10 | Stepwise (univariate first) | Square root | Normalize dose | None | NA | None | R2 | None | NI | N | N | PY | NI | N | N | N | NI | High | Y | - |
| 85-  88 | Bress 2012^101^ | MLR | 252/258 | ~23 | 10_20 | NI (univariate first) | Log | Normalize residuals | None | NA | None | R2 | Missing genotype data | Excluded | NC | NI | N | N | N | N | N | NI | High | Y | - |
| 89-  92 |  | MLR | 50/ 53 | ~23 | <10 | NI (univariate first) | Log |  | None | NA | None | R2 |  | Excluded | N | NI | N | N | N | N | N | NI | High | Y | - |
| 93 | Cini 2012^102^ | MLR | 55 | ~18 | <10 | All variables (from univariate) | Square root | NS | Bracketing | 25, 45 | External | - | Missing clinical | Excluded | N | NI | N | N | N | N | N | Y | High | Y | - |
| 94 | Chan 2012^103^ | MLR | 248 | ~8 | >20 | Based on previous studies | Log | NI | None | NA | Random split, 100 repeats | R2, ideal dose | None | NI | Y | NI | PY | NI | PY | N | PN | NI | High | Y | - |
| 95 | El Din 2012^104^ | MLR | 46 | ≥8 | <10 | NI (univariate conducted) | None | NA | None | NA | None | R2 | None | NI | N | NI | PY | NI | N | N | N | NI | High | Y | - |
| 96 | Kurnik 2012^105^ | MLR | 210 | ~18 | 10_20 | Based on IWPC | Square root | Based on IWPC | None | NA | None | R2^c^ (on √ scale), MAE | None | NI | NC | NI | PY | NI | Y | Y | N | NI | High | Y | - |
| 97 | Lee 2012^106^ | MLR | 191 | ≥20 | <10 | NI (univariate first) | None | NA | None | NA | None | R2 | None | NI | N | NI | PY | NI | N | N | N | Y | High | Y | - |
| 98 | Liang 2012^107^ | MLR | 115 | ~10 | 10_20 | Stepwise (univariate first) | Square root | Normalize dose | None | NA | None | R2, MAE | None | NI | NC | NI | Y | Y | N | Y | N | NI | High | Y | - |
| 99 | Lou 2012 | NI | 488 | NI | NI | NI | NI | NI | NI | NI | NI | R2 | NI | NI | NI | NI | NI | NI | NI | NI | NI | NI | Unclear | NI | - |
| 100 | Moreau 2012^108^ | MLR | 83 | ≥9 | <10 | Backward stepwise (univariate first) | None | NA | None | NA | None | R2, within 1 mg/d | None | NI | N | NI | PY | NI | N | N | N | Y | High | Y | - |
| 101 | Pathare 2012^111^ | MLR | 142 | ≥20 | <10 | NI (univariate first) | Log | Normalize dose,  constant variance | None | NA | Random split | R2, MPE | Outliers | NI | N | NI | N | NI | N | N | N | Y | High | Y | - |
| 102 | Pavani 2012^112^ | MLR | 125 | ~10 | 10_20 | Stepwise (univariate done) | None | NA | NI | 21, 70 | None | r | None | NI | NC | NI | PY | NI | NI | N | N | NI | High | N | - |
| 103 | Pavani 2012^113^ | MLR | 160 | ≥12 | 10_20 | NI (univariate conducted) | None | NA | NI | 21, 49 | None | R2, diagnostic, Bland-Altman | None | NI | NC | NI | PY | NI | PN | N | N | NI | High | Y | - |
| 104 | Ramirez 2012^114^ | MLR | 1167 | <30 | >20 | NI (univariate first) | Log | NI | None | NA | Bootstrap | R2, MAE, as diagnostics | Missing data | Excluded | Y | NI | N | N | PN | Y | PY | Y | High | Y | - |
| 105 |  | MLR | 1167 | <30 | >20 | NI (univariate first) | Log | NI | None | NA | Bootstrap |  | Missing data | Excluded | Y | NI | N | N | PN | Y | PY | Y | High | Y | - |
| 106 | Tan 2012^115^ | MLR | 321 | ~20 | 10_20 | Stepwise (univariate first) | Square root | Normalize dose | NI | 13.2, 30.7 | Random split | R2, MPE, r, ideal dose | None | NI | NC | NI | PY | NI | N | N | N | Y | High | Y | - |

**Table S6. Continued**

| **#** | **Algorithm** | **Sub-type** | **N** | **CPP** | **PCV** | **Predictor selection** | **Dose transformation** | **Reason** | **Risk groups** | **Cut-offs** | **Validation (as report**  **ed in study** | **Performance measures reported** | **Excluded participants** | **Missing data handling** | **PROBAST signaling questions** | | | | | | | | **ROB** | **Stable dose defined** | **Other key ROB concerns**  **(domains 1-3)** |
| --- | --- | --- | --- | --- | --- | --- | --- | --- | --- | --- | --- | --- | --- | --- | --- | --- | --- | --- | --- | --- | --- | --- | --- | --- | --- |
|  |  |  |  |  |  |  |  |  |  |  |  |  |  |  | **4.1** | **4.2** | **4.3** | **4.4** | **4.5** | **4.7** | **4.8** | **4.9** |  |  |  |
| 107 | Tatarunas 2012^116^ | MLR | 189 | >20 | <10 | Stepwise (univariate first) | None | NA | None | NA | None | R2 | None | NI | N | N | PY | NI | N | N | N | NI | High | Y | - |
| 108 | Teh 2012^117^ | MLR | 86 | ~8 | 10_20 | NI | None | NA | None | NA | External | R2 | None | NI | NC | NI | PY | NI | NI | N | N | NI | High | Y | - |
| 109 | Wei 2012^118^ | MLR | 260 | ≥21 | 10_20 | Backward stepwise (univariate first) | None | NA | None | NA | Random split | R2, r | None | NI | NC | NI | PY | NI | N | N | N | Y | High | Y | - |
| 110 | Xu 2012^22^ | MLR | 207 | ~21 | <10 | Stepwise (univariate first) | Square root | NI | Bracketing | 14, 28 | Random split | R2, MAE, ideal dose | None | NI | N | NI | PY | NI | N | Y | N | NI | High | Y | - |
| 111 | Zhang 2012^119^ | MLR | 297 | 20 | 10_20 | Stepwise (univariate first) | None | NA | None | NA | Random split | R2, within 1 mg/d | None | NI | NC | NI | PY | NI | N | N | N | NI | High | Y | - |
| 112 | Zhong 2012^120^ | MLR | 591 | ~30 | 10_20 | Forward stepwise (univariate first) | Square root | Normalize dose | None | NA | Random split | R2, r | Missing data | Excluded | NC | NI | N | N | N | N | N | NI | High | Y | - |
| 113 | Zhu 2012^121^ | MLR | 322 | ~7 | >20 | Stepwise | None | NA | None | NA | None | R2 | None | NI | Y | NI | PY | NI | NI | N | N | NI | High | Y | - |
| 114 | Daneshjou 2013^122^ | Pathway analysis | 188 | ≥49 | <10 | Based on IWPC equation and pathway analysis | Square root | IWPC-based | None | NA | None | R2 | Missing data | Excluded | N | NI | N | N | PY | N | N | NA | High | Y | - |
| 115 |  |  | 233 | ≥49 | <10 |  | Square root | IWPC-based | None | NA | None | - | Missing data | Excluded | N | NI | N | N | PY | N | N | NA | High | Y | - |
| 116 |  |  | 302 | ≥49 | <10 |  | Square root | IWPC-based | None | NA | None | R2 | Missing data | Excluded | N | NI | N | N | PY | N | N | NA | High | Y | - |
| 117 | Ekladious 2013^123^ | MLR | 50 | 5 | 10_20 | Stepwise | None | NA | None | NA | External | R2 | Missing data | Excluded | NC | NI | N | N | PY | N | N | NI | High | Y | - |
| 118 | Kabagambe 2013^124^ | MLR | 172 | 16 | 10_20 | Forward (univariate first) | Square root | Normalize dose | None | NA | None | R2 | Missing data, 1 Asian, outliers | Excluded | NC | N | N | N | N | N | N | NI | High | Y (for some) | - |
| 119 |  | MLR | 172 | 16 | 10_20 | Forward (univariate first) | Square root | Normalize dose | None | NA | None | R2 | Missing data, 1 Asian, outliers | Excluded | NC | N | N | N | N | N | N | NI | High | Y (for some) | - |
| 120 | Mazzaccara 2013^125^ | MLR | 266 | ~25 | 10_20 | NI (univariate first) | Log | NI | None | NA | LOOCV | R2 | None | NI | NC | N | PY | NI | N | N | N | NI | High | N | - |
| 121 | Natarajan 2013^126^ | MLR | 103 | ~6 | 10_20 | NI (univariate first) | None | NA | None | NA | None | R2 | None | NI | NC | NI | PY | NI | N | N | N | NI | High | N | Out-of-range INRs included |
| 122 | Nguyen 2013^127^ | MLR | 37 | ≥6 | <10 | NI (univariate first) | None | NA | None | NA | None | R2 | None | NI | N | NI | PY | NI | PN | N | N | NI | High | Y | - |
| 123 | Ozer 2013^128^ | MLR | 107 | ≥12 | <10 | Forward stepwise (univariate first) | None | NA | None | NA | None | R2 | None | NI | N | NI | PY | NI | N | N | N | Y | High | Y | - |
| 124 | Park 2013^129^ | MLR | 204 | 34 | <10 | NI (univariate first) | Log | Normalize dose | None | NA | None | R2 | None | NI | N | NI | PY | NI | N | N | N | NI | High | Y | - |
| 125 | Perera 2013^130^ | MLR | 504 | >500 | <10 | Based on IWPC and GWAS | Log | Normalize dose | None | NA | External | R2 | Missing clinical data | Excluded | N | NI | N | N | N | N | N | NI | High | Y | - |
| 126 | Shahin 2013^131^ | MLR | 195 | ≥30 | <10 | Stepwise (univariate first) | Square root | Heteroscedascity and model fit | None | NA | None | R2 | Missing genotype data | Excluded | N | NI | N | N | N | N | N | NI | High | Y | - |
| 127 | Tan 2013^132^ | MLR | 317 | ~9 | >20 | Stepwise (univariate first) | Square root | Normalize dose | None | NA | None | R2 | None | NI | Y | NI | PY | NI | N | N | N | NI | High | Y | - |
| 128 | Bazan 2014^133^ | MLR | 63 | >10 | <10 | Stepwise (univariate first) | None | NA | None | NA | None | R2 | None | NI | N | NI | PY | NI | N | N | N | NI | High | Y | - |
| 129 | Bosch 2014^134^ | MLR | 121 | ≥14 | <10 | Stepwise (univariate first) | Log | NS | Bracketing | 21, 49 | None | R2, MSE | Missing data | Excluded | N | NI | N | N | N | Y | N | N | High | Y | - |
| 130 | Chen 2014^135^ | MLR | 551 | ~40 | 10_20 | Stepwise (univariate first) | None | NA | Bracketing | 14, 28 | External | R2, r, ideal dose | Missing genotype data | Excluded | NC | NI | N | N | N | N | N | NI | High | Y | - |
| 131 | Daneshjou 2014^136^ | MLR | 476 | ~7 | >20 | Based on IWPC and discovery cohort | Log | NI | NI | 35, 49 | None | - | None | NI | Y | NI | PY | NI | N | N | N | NI | High | N | - |
| 132 | Grossi 2014^137^ | ANN | 377 | 48 | <10 | TWIST system | None | NA | NI | 21, 49 | Random split, 10 repeats | R2, MAE, ideal dose | None | NI | N | PY | PY | NI | PY | Y | PN | NA | High | Y | Out-of-range patients included |
| 133 | Issac 2014^138^ | MLR | 84 | ~10 | <10 | Stepwise (univariate conducted) | None | NA | None | NA | None | R2 | Missing clinical data | Excluded | N | NI | N | N | N | N | N | NI | High | Y | - |
| 134 |  | MLR | 84 | ~10 | <10 |  | None | NA | None | NA | None | R2 |  | Excluded | N | NI | N | N | N | N | N | NI | High | Y | - |
| 135 | Krishna Kumar  2014^139^ | MLR | 240 | 19 | 10_20 | Stepwise (univariate first) | Log | NI | NI | 17.5, 49 | Bootstrap | R2, within 1 mg/d | Missing data | Excluded | NC | NI | N | N | N | N | N | Y | High | Y | - |

**Table S6. Continued**

| **#** | **Algorithm** | **Sub-type** | **N** | **CPP** | **PCV** | **Predictor selection** | **Dose transformation** | **Reason** | **Risk groups** | **Cut-offs** | **Validation (as report**  **ed in study** | **Performance measures reported** | **Excluded participants** | **Missing data handling** | **PROBAST signaling questions** | | | | | | | | **ROB** | **Stable dose defined** | **Other key ROB concerns**  **(domains 1-3)** |
| --- | --- | --- | --- | --- | --- | --- | --- | --- | --- | --- | --- | --- | --- | --- | --- | --- | --- | --- | --- | --- | --- | --- | --- | --- | --- |
|  |  |  |  |  |  |  |  |  |  |  |  |  |  |  | **4.1** | **4.2** | **4.3** | **4.4** | **4.5** | **4.7** | **4.8** | **4.9** |  |  |  |
| 136 | Lou 2014 | MLR | NI | NI | NI | NI | NI | NI | NI | NI | NI | NI | NI | NI | NI | NI | NI | NI | NI | NI | NI | NI | Unclear | NI | - |
| 137 | Pavani 2014^142^ | Polynomial | 125 | 18 | <10 | Stepwise (univariate conducted) | None | NA | None | NA | None | Bland-Altman, r, diagnostics (AUC, ROC) | None | NI | N | PY | PY | NI | N | N | N | NI | High | Y | - |
| 138 |  | MLR | 125 | 11 | 10_20 | Stepwise (univariate done) | None | NA | None | NA | None |  | None | NI | NC | NI | PY | NI | N | N | N | NI | High | Y | - |
| 139 | Saleh 2014^143^ | ANN | 3415 | ~13 | >20 | Backwards selection | NI | NA | NI | 21, 49 | 10-fold CV; random split | R2, MAE, ideal dose | None | Likely excluded | Y | PY | PY | N | Y | Y | PY | NA | High | Y | - |
| 140 | Shaw 2014^144^ | MLR | 77 | ≥25 | <10 | Stepwise (univariate first) | Square root | Normalize dose | None | NA | None | R2, within 1 mg/d | None | NI | N | N | PY | NI | N | N | N | NI | High | Y | - |
| 141 | Tatarunas 2014^145^ | MLR | 189 | >20 | <10 | Stepwise (univariate first) | None | NA | None | NA | None | R2 | None | NI | N | NI | PY | NI | N | N | N | NI | High | N | - |
| 142 | Vear 2014^146^ | MLR | 100 | ≥13 | <10 | Backwards (univariate conducted) | Log | NI | None | NA | None | R2 | Missing genotype data | Excluded | N | NI | N | N | N | N | N | NI | High | Y | - |
| 143 | Ye 2014^147^ | MLR | 101 | ~7 | 10_20 | Stepwise (univariate first) | None | NA | None | NA | None | R2 | None | NI | NC | NI | PY | NI | PN | N | N | NI | High | Y | - |
| 144 | An 2015^148^ | MLR | 191 | >31 | <10 | Stepwise (univariate first) | None | NA | None | NA | None | R2 | 15 (based on Chung 2015) | Probably excluded | N | NI | N | N | N | N | N | NI | High | Y | - |
| 145 | Chung 2015^149^ | MLR | 206 | >30 | <10 | Stepwise (univariate first) | None | NA | None | NA | None | R2 | None | NI | N | NI | PY | NI | N | N | N | NI | High | Y | - |
| 146 | Drozda 2015^150^ | MLR | 274 | ~15 | >20 | Based on Gage 2008 | Log | Gage 2008 | None | NA | None | Bland-Altman | None | NI | Y | NI | PY | NI | PY | N | N | NI | High | Y | - |
| 147 | Ghozlan 2015^151^ | MLR | 80 | ~7 | 10_20 | Stepwise (univariate first) | None | NA | None | NA | None | R2 | None | NI | NC | NI | PY | NI | N | N | N | NI | High | Y | - |
| 148 | Ichihara 2015^152^ | MLR | 137 | >15 | <10 | Forward stepwise (univariate first) | None | NA | None | NA | None | R2 | None | NI | N | NI | PY | NI | N | N | N | NI | High | Y | - |
| 149 |  | MLR | 137 | >15 | <10 | Forward stepwise (univariate first) | None | NA | None | NA | None | R2 | None | NI | N | NI | PY | NI | N | N | N | NI | High | Y | - |
| 150 | Jeong 2015^153^ | MLR | 201 | >30 | <10 | Stepwise (univariate first) | None | NA | None | NA | None | R2 | See Chung 2015 | NI | N | N | N | N | N | N | N | NI | High | Y | - |
| 151 | Karaca 2015^154^ | MLR | 97 | 22 | <10 | Stepwise (univariate first) | Log | NI | None | NA | External | R2, MAE, ideal dose, AIC | None | NI | N | NI | PY | NI | N | Y | N | NI | High | N | - |
| 152 | Lee 2015^155^ | MLR | 201 | >30 | <10 | Stepwise (univariate first) | None | NA | None | NA | None | R2 | See Chung 2015 | NI | N | N | N | N | N | N | N | NI | High | Y | - |
| 153 | Li 2015^18^ | MLR | 1036 | NI | >20 | Stepwise regression | Square root | Normalize dose | Quantiles  (30^th^, 70^th^) | 13.2, 30.7 | External | MAE, ideal dose | Missing data; rare genotypes | Excluded | Y | NI | N | N | NI | Y | N | NI | High | Y | - |
| 154 |  | SVR | 1036 | NI | >20 | Stepwise regression | Square root | Normalize dose |  |  | External |  |  | Excluded | Y | PY | N | N | NI | Y | N | NA | High | Y | - |
| 155 |  | ANN | 1036 | NI | >20 | Stepwise regression | Square root | Normalize dose |  |  | External |  |  | Excluded | Y | PY | N | N | NI | Y | N | NA | High | Y | - |
| 156 |  | RT | 1036 | NI | >20 | Stepwise regression | Square root | Normalize dose |  |  | External |  |  | Excluded | Y | PY | N | N | NI | Y | N | NA | High | Y | - |
| 157 |  | RFR | 1036 | NI | >20 | Stepwise regression | Square root | Normalize dose |  |  | External |  |  | Excluded | Y | PY | N | N | NI | Y | N | NA | High | Y | - |
| 158 |  | BRT | 1036 | NI | >20 | Stepwise regression | Square root | Normalize dose |  |  | External |  |  | Excluded | Y | PY | N | N | NI | Y | N | NA | High | Y | - |
| 159 |  | MARS | 1036 | NI | >20 | Stepwise regression | Square root | Normalize dose |  |  | External |  |  | Excluded | Y | PY | N | N | NI | Y | N | NA | High | Y | - |
| 160 | Li 2015^156^ | MLR | 1297 | ~10 | >20 | Stepwise | Square root | Based on Li 2015 | None | NA | Random split | R2, MAE, ideal dose | Missing genotype data | Excluded | Y | NI | N | N | NI | Y | N | NI | High | Y | - |
| 161-  164 |  | MLR | 1297 | ~10 | >20 | Stepwise | Square root |  | None | NA |  |  |  | Excluded | Y | NI | N | N | NI | Y | N | NI | High | Y | - |
| 165-  168 |  | MLR | 2155 | <20 | >20 | Stepwise | Square root |  | None | NA |  |  |  | Excluded | Y | NI | N | N | NI | Y | N | NI | High | Y | - |
| 169 |  | MLR | 2155 | <20 | >20 | Stepwise | Square root |  | None | NA |  |  |  | Excluded | Y | NI | N | N | NI | Y | N | NI | High | Y | - |
| 170 | Limdi 2015^157^ | MLR | 1357 | 17 | >20 | Based on Gage (2008) | Log | Normalize dose | None | NA | None | R2 | Missing genotype data | Excluded | Y | NI | N | N | Y | N | N | NI | High | Y | - |
| 171 |  | MLR | 1357 | 39 | >20 | Expert knowledge and NI (univariate) | Log | Normalize dose | None | NA | None | R2 | Missing genotype data | Excluded | Y | NI | N | N | N | N | N | NI | High | Y | - |

**Table S6. Continued**

| **#** | **Algorithm** | **Sub-type** | **N** | **CPP** | **PCV** | **Predictor selection** | **Dose transformation** | **Reason** | **Risk groups** | **Cut-offs** | **Validation (as report**  **ed in study** | **Performance measures reported** | **Excluded participants** | **Missing data handling** | **PROBAST signaling questions** | | | | | | | | **ROB** | **Stable dose defined** | **Other key ROB concerns**  **(domains 1-3)** |
| --- | --- | --- | --- | --- | --- | --- | --- | --- | --- | --- | --- | --- | --- | --- | --- | --- | --- | --- | --- | --- | --- | --- | --- | --- | --- |
|  |  |  |  |  |  |  |  |  |  |  |  |  |  |  | **4.1** | **4.2** | **4.3** | **4.4** | **4.5** | **4.7** | **4.8** | **4.9** |  |  |  |
| 172 | Limdi 2015^157^ | MLR | 762 | 8 | >20 | Based on Gage (2008) | Log | Normalize dose | None | NA | None | R2 | Missing genotype data | Excluded | Y | NI | N | N | Y | N | N | NI | High | Y | - |
| 173 |  | MLR | 762 | 29 | >20 | Expert knowledge and NI (univariate) | Log | Normalize dose | None | NA | None | R2 | Missing genotype data | Excluded | Y | NI | N | N | N | N | N | NI | High | Y | - |
| 174 |  | MLR | 595 | 8 | >20 | Based on Gage (2008) | Log | Normalize dose | None | NA | None | R2 | Missing genotype data | Excluded | Y | NI | N | N | Y | N | N | NI | High | Y | - |
| 175 |  | MLR | 595 | 29 | >20 | Expert knowledge and NI (univariate) | Log | Normalize dose | None | NA | None | R2 | Missing genotype data | Excluded | Y | NI | N | N | N | N | N | NI | High | Y | - |
| 176 | Liu 2015^20^ | MLR | 3838 | ~10 | >20 | Stepwise regression | Square root | Normalize dose | Quantiles | Asians:  14, 26.3  Whites:  22,42.5  Blacks:  30,52.2 Mixed: 22.5, 40 | Random split | MAE, ideal dose | Missing data | Excluded | Y | NI | N | N | NI | Y | N | NI | High | Y | - |
| 177 |  | SVR | 3838 | ~10 | >20 | Stepwise regression | Square root | Normalize dose | Quantiles |  |  |  | Missing data | Excluded | Y | PY | N | N | NI | Y | N | NA | High | Y | - |
| 178 |  | ANN | 3838 | ~10 | >20 | Stepwise regression | Square root | Normalize dose | Quantiles |  |  |  | Missing data | Excluded | Y | PY | N | N | NI | Y | N | NA | High | Y | - |
| 179 |  | RT | 3838 | ~10 | >20 | Stepwise regression | Square root | Normalize dose | Quantiles |  |  |  | Missing data | Excluded | Y | PY | N | N | NI | Y | N | NA | High | Y | - |
| 180 |  | RFR | 3838 | ~10 | >20 | Stepwise regression | Square root | Normalize dose | Quantiles |  |  |  | Missing data | Excluded | Y | PY | N | N | NI | Y | N | NA | High | Y | - |
| 181 |  | BRT | 3838 | ~10 | >20 | Stepwise regression | Square root | Normalize dose | Quantiles |  |  |  | Missing data | Excluded | Y | PY | N | N | NI | Y | N | NA | High | Y | - |
| 182 |  | MARS | 3838 | ~10 | >20 | Stepwise regression | Square root | Normalize dose | Quantiles |  |  |  | Missing data | Excluded | Y | PY | N | N | NI | Y | N | NA | High | Y | - |
| 183 |  | LASSO | 3838 | ~10 | >20 | Stepwise regression | Square root | Normalize dose | Quantiles |  |  |  | Missing data | Excluded | Y | PY | N | N | NI | Y | NA | NA | High | Y | - |
| 184 |  | BART | 3838 | ~10 | >20 | Stepwise regression | Square root | Normalize dose | Quantiles |  |  |  | Missing data | Excluded | Y | PY | N | N | NI | Y | N | NA | High | Y | - |
| 185 | Moon 2015^158^ | MLR | 201 | >30 | <10 | Stepwise (univariate first) | None | NA | None | NA | None | R2 | See Chung 2015 | NI | N | N | N | N | N | N | N | NI | High | Y | - |
| 186 | Oztaner 2015^159^ | BSE-MLE | 2982 | 9 | >20 | Expert knowledge and literature review | Log | Normalize dose | None | NA | CV, random split | R2 | With extensive missing data | Excluded, multiple imputation | Y | PY | N | N | Y | N | N | NA | High | Y | - |
| 187 |  | BSE-MLE | 72 | 8 | <10 | Complete eligible set of variables | Log | Normalize dose | None | NA | CV, random split | R2 | None | NA | N | PY | Y | Y | Y | N | N | NA | High | Y | - |
| 188 | Santos 2015^160^ | MLR | 368 | ~15 | >20 | Based on IWPC 2009 | Square root | IWPC 2009 | None | NA | Random split (10,000 repeats), external | R2 | None | NI | Y | NI | PY | NI | Y | N | N | NI | High | Y | - |
| 189 | Alzubiedi 2016^161^ | MLR | 163 | ≥20 | <10 | Stepwise (univariate first) | Square root | NI | Bracketing | 21, 49 | 10-fold CV | R2, MAE, ideal dose | None | NI | N | NI | PY | NI | N | Y | N | Y | High | Y | - |
| 190 |  | ANN | 163 | ≥20 | <10 | Stepwise (univariate first) | Square root | NI | Bracketing | 21, 49 | 10-fold CV | R2, MAE, ideal dose | None | NI | N | PY | PY | NI | N | Y | N | NA | High | Y | - |
| 191 | Cho 2016^162^ | MLR | 101 | ~20 | <10 | Stepwise (univariate first) | Log | Normalize dose | NI | 21, 49 | None | R2, M%PE, ideal dose | None | NI | N | NI | PY | NI | N | N | N | NI | High | Y | - |
| 192 | Eriksson 2016^163^ | MLR | 951 | >554725 | <10 | Based on IWPC and GWAS | Square root | IWPC-based | NI | 21, 49 | CV, random split | R2, ideal dose | None | NI | N | NI | PY | NI | N | N | N | NI | High | Y | - |
| 193 | Jiang 2016^164^ | MLR | 122 | ~8 | 10_20 | NI | None | NA | None | NA | External | R2 | Recruited ≠ analyzed | NI | NC | NI | N | N | NI | N | N | NI | High | Y | - |
| 194 | Li 2016 | NI | 384 | NI | NI | NI | NI | NI | NI | NI | NI | R2 | NI | NI | NI | NI | NI | NI | NI | NI | NI | NI | Unclear | NI | - |
| 195 | Liu 2016^165^ | MLR | 186 | ~17 | 10_20 | NI | Square root | Normalize dose | None | NA | None | R2 | Missing data, rare genotypes | Excluded | NC | NI | N | N | NI | N | N | NI | High | Y | - |
| 196 | Pavani 2016^166^ | ANN | 157 | ≥17 | <10 | Probably all existing included | None | NA | Unclear | 28, 49 | None | R2, MPE | None | NI | N | PY | NI | NI | PY | N | N | NA | High | N | - |
| 197 | Rouleau-Mailloux 2016^167^ | MLR | 969 | <20 | >20 | Stepwise (univariate first) | Log | Normalize dose | None | NA | None | R2 | Missing data and outliers | Excluded | Y | PN | N | N | N | N | N | Y | High | Y | Sub-optimal definition |
| 198 |  | MLR | 618 | <20 | >20 | Stepwise (univariate first) | Log | Normalize dose | None | NA | None | R2 | Missing data | Excluded | Y | PN | N | N | N | N | N | Y | High | Y | Sub-optimal definition |

**Table S6. Continued**

| **#** | **Algorithm** | **Sub-type** | **N** | **CPP** | **PCV** | **Predictor selection** | **Dose transformation** | **Reason** | **Risk groups** | **Cut-offs** | **Validation (as report**  **ed in study** | **Performance measures reported** | **Excluded participants** | **Missing data handling** | **PROBAST signaling questions** | | | | | | | | **ROB** | **Stable dose defined** | **Other key ROB concerns**  **(domains 1-3)** |
| --- | --- | --- | --- | --- | --- | --- | --- | --- | --- | --- | --- | --- | --- | --- | --- | --- | --- | --- | --- | --- | --- | --- | --- | --- | --- |
|  |  |  |  |  |  |  |  |  |  |  |  |  |  |  | **4.1** | **4.2** | **4.3** | **4.4** | **4.5** | **4.7** | **4.8** | **4.9** |  |  |  |
| 199 | Shahabi 2016^168^ | MLR | 1046 | See Gage 2008 | | | | | None | NA | NA | MAE, within 1 mg/d | Missing data | Excluded | Y | NI | N | N | N | Y | PN | NI | High | Y | Sub-optimal definitions |
| 200 | Wakamiya 2016^169^ | MLR | 45 | >15 | <10 | Stepwise (univariate first) | Square root | Normalize dose | None | NA | None | R2, within 1 mg/d | None | NI | N | NI | PY | NI | N | N | N | NI | High | Y | - |
| 201 |  | MLR | 408 | <20 | >20 | Backward stepwise (univariate first) | Square root | NI | None | NA | None | R2 | None | NI | Y | NI | PY | NI | N | N | N | Y | High | Y | - |
| 202 | Zeng 2016^170^ | MLR | 408 | <20 | >20 | Backward stepwise (univariate first) | Square root | NI | None | NA | None | R2 | None | NI | Y | NI | PY | NI | N | N | N | Y | High | Y | - |
| 203 |  | MLR | 408 | <20 | >20 | Backward stepwise (univariate first) | Square root | NI | None | NA | None | R2 | None | NI | Y | NI | PY | NI | N | N | N | Y | High | Y | - |
| 204 | Claudio-Campos 2017^171^ | MLR | 115 | ≥207 | <10 | Stepwise (univariate first) | None | NA | Quantiles | 28, 42 | None | R2 | Missing data | Excluded | N | NI | N | N | N | N | N | NI | High | Y | - |
| 205 |  | MLR | 115 | ≥207 | <10 | Stepwise (univariate first) | None | NA | Quantiles | 28, 42 | None | R2, MAE, mean SE | Missing data | Excluded | N | NI | N | N | N | Y | N | NI | High | Y | - |
| 206 | Jiang 2017^172^ | MLR | 215 | <10 | >20 | NI | None | NA | None | NA | Likely random split | R2, r | None | NI | Y | NI | PY | NI | PN | N | N | Y | High | N | - |
| 207 | Liu 2017^173^ | MLR | 183 | >100 | <10 | Stepwise (univariate first) | Square root | Normalize dose | Bracketing | 14, 28 | None | R2 | Missing data | Excluded | N | NI | N | N | N | N | N | Y | High | Y | - |
| 208 | Luo 2017^174^ | MLR | 420 | >50 | <10 | NI (univariate first) | None | NA | None | NA | None | R2 | Missing data | Excluded | N | NI | N | N | N | N | N | NI | High | Y | - |
| 209 | Sohrabi 2017^175^ | MOPSO | 533 | 19 | >20 | Best k-variable subsets | None | NA | None | NA | None | MAE, MSE, RMSE | None | NI | PY | PY | PY | NI | PY | Y | N | NA | High | Y | - |
| 210 |  | NSGA-II | 533 | 19 | >20 | Best k-variable subsets | None | NA | None | NA | None |  | None | NI | PY | PY | PY | NI | PY | Y | N | NA | High | Y | - |
| 211 | Tang 2017^176^ | MLR | 231 | ~9 | >20 | Stepwise (univariate first) | Square root | Normalize dose | None | NA | None | R2 | None | NI | Y | NI | PY | NI | N | N | N | NI | High | Y | - |
| 212 | Lee 2017^177^ | MLR | 204 | >30 | <10 | Stepwise (univariate first) | None | NA | None | NA | None | R2, ideal dose, | 2 (based on Chung 2015) | Probably excluded | N | NI | PN | N | N | N | N | NI | High | Y | - |
| 213 |  | MLR | 204 | >30 | <10 | Stepwise (univariate first) | None | NA | None | NA | None |  |  |  | N | NI | PN | N | N | N | N | NI | High | Y | - |
| 214 | Wattanachai 2017^178^ | MLR | 250 | ≥30 | <10 | Stepwise (univariate first) | None | NA | None | NA | None | R2 | None | NI | N | NI | PY | NI | N | N | N | NI | High | Y | - |
| 215-  226 | Wiley 2017^179^ | MLR | 2181 | ≤26 | >20 | Selected subsets | None | NA | NI | 21, 49 | Bootstrap | R2, MAE | Missing data | Excluded | Y | NI | N | N | PY | Y | NI | NI | High | Y | - |
| 227-230 |  | MLR | 1928 | ≤26 | >20 | Selected subsets | None | NA | NI | 21, 49 | Bootstrap | R2, MAE | Missing data | Excluded | Y | NI | N | N | PY | Y | NI | NI | High | Y | - |
| 231-  234 |  | MLR | 253 | ≤26 | <10 | Selected subsets | None | NA | NI | 21, 49 | Bootstrap | R2, MAE | Missing data | Excluded | N | NI | N | N | PY | Y | NI | NI | High | Y | - |
| 235 | Zhu 2017^180^ | NONME  M/MLR | 144 | ~21 | 10_20 | Forward then backward (univariate first) | None | NA | None | NA | None | R2, MPE, RMSE | None | NI | NC | NI | PY | NI | N | Y | N | NI | High | N | - |
| 236 | Chung 2018^181^ | MLR | 201 | >30 | <10 | Stepwise (univariate first) | None | NA | None | NA | None | R2, ideal dose | 5 missing (Chung 2015) | Excluded | N | NI | N | N | N | N | N | NI | High | Y | - |
| 237 | Gaikwad 2018^182^ | MLR | 300 | ≥18 | 10_20 | Stepwise (univariate first) | Square root | Normalize dose, equalize variance | None | NA | None | R2 | None | NI | NC | N | PY | NI | N | N | N | Y | High | N | - |
| 238 | Galvez 2018^183^ | MLR | 152 | ~8 | 10_20 | NI (univariate first) | Square root | NI | None | NA | External | R2 | None | NI | NC | NI | PY | NI | PN | N | N | NI | High | Y | - |
| 239 | Kabalak 2018^184^ | MLR | 75 | ≥10 | <10 | NI (univariate conducted) | None | NA | None | NA | None | R2 | None | NI | N | NI | PY | NI | N | N | N | NI | High | Y | - |
| 240 | Ma 2018^185^ | MLR | 4594 | ~20 | >20 | Based on IWPC and previous models | Square root | Normalize dose | Quantiles | Asians:  15, 28 Blacks:  30, 52.2 | 5-fold CV and random split | MAE, ideal dose | Rare genotypes and 1 outlier | Likely single imputation | Y | N | PN | PN | Y | Y | PY | NI | High | Y | - |
| 241 |  | SVR | 4594 | ~20 | >20 |  | Square root | Normalize dose | Quantiles |  |  |  |  |  | Y | N | PN | PN | Y | Y | PY | NA | High | Y | - |
| 242 |  | RR | 4594 | ~20 | >200 |  | Square root | Normalize dose | Quantiles |  |  |  |  |  | Y | N | PN | PN | Y | Y | PY | NA | High | Y | - |
| 243 |  | ANN | 4594 | ~20 | >200 |  | Square root | Normalize dose | Quantiles |  |  |  |  |  | Y | N | PN | PN | Y | Y | PY | NA | High | Y | - |
| 244 |  | GBT | 4594 | ~20 | >200 |  | Square root | Normalize dose | Quantiles |  |  |  |  |  | Y | N | PN | PN | Y | Y | PY | NA | High | Y | - |

**Table S6. Continued**

| **#** | **Algorithm** | **Sub-type** | **N** | **CPP** | **PCV** | **Predictor selection** | **Dose transformation** | **Reason** | **Risk groups** | **Cut-offs** | **Validation (as report**  **ed in study** | **Performance measures reported** | **Excluded participants** | **Missing data handling** | **PROBAST signaling questions** | | | | | | | | **ROB** | **Stable dose defined** | **Other key ROB concerns**  **(domains 1-3)** |
| --- | --- | --- | --- | --- | --- | --- | --- | --- | --- | --- | --- | --- | --- | --- | --- | --- | --- | --- | --- | --- | --- | --- | --- | --- | --- |
|  |  |  |  |  |  |  |  |  |  |  |  |  |  |  | **4.1** | **4.2** | **4.3** | **4.4** | **4.5** | **4.7** | **4.8** | **4.9** |  |  |  |
| 245 | Ma 2018^185^ | RFR | 4594 | ~20 | >200 | Continued from previous page | Square root | Normalize dose | Quantiles | Whites: 22, 42.5  Mixed:  22, 40 |  |  |  |  | Y | N | PN | PN | Y | Y | PY | NA | High | Y | - |
| 246 |  | ERT | 4594 | ~20 | >200 |  | Square root | Normalize dose | Quantiles |  |  |  |  |  | Y | N | PN | PN | Y | Y | PY | NA | High | Y | - |
| 247 |  | KNN | 4594 | ~20 | >200 |  | Square root | Normalize dose | Quantiles |  |  |  |  |  | Y | N | PN | PN | Y | Y | PY | NA | High | Y | - |
| 248 |  | SGF | 4594 | ~20 | >200 |  | Square root | Normalize dose | Quantiles |  |  |  |  |  | Y | N | PN | PN | Y | Y | PY | NA | High | Y | - |
| 249 | Pei 2018^186^ | MLR | 247 | >40 | <10 | Stepwise (univariate first) | None | NA | NI | 14, 28 | External | R2, MAE, r, MAPE, ideal dose | Missing genotype data | Excluded | N | NI | N | N | N | Y | N | NI | High | Y | - |
| 250 | Selim 2018^187^ | MLR | 100 | ≥11 | <10 | NI (univariate first) | None | NA | NI | 21, 49 | External | R2 | None | NI | N | NI | PY | NI | N | N | N | NI | High | Y | - |
| 251 | Tavares 2018^188^ | MLR | 309 | ~9 | >20 | NI | None | NA | None | NA | None | - | None | NI | Y | NI | PY | NI | NI | N | N | NI | High | Y | - |
| 252 |  | MLR | 309 | ~9 | >20 | NI | None | NA | None | NA | None | - | None | NI | Y | NI | PY | NI | NI | N | N | NI | High | Y | - |
| 253 |  | MLR | 233 | ~9 | >20 | NI | None | NA | None | NA | None | - | None | NI | Y | NI | PY | NI | NI | N | N | NI | High | Y | - |
| 254 |  | MLR | 233 | ~9 | >20 | NI | None | NA | None | NA | None | - | None | NI | Y | NI | PY | NI | NI | N | N | NI | High | Y | - |
| 255 |  | MLR | 76 | ~9 | <10 | NI | None | NA | None | NA | None | - | None | NI | N | NI | PY | NI | NI | N | N | NI | High | Y | - |
| 256 |  | MLR | 76 | ~9 | <10 | NI | None | NA | None | NA | None | - | None | NI | N | NI | PY | NI | NI | N | N | NI | High | Y | - |
| 257 | Tavares 2018^189^ | MLR | 309 | ~12 | >20 | NI (likely no selection) | None | NA | None | NA | random split (15,000 repeats); external | R2, MRDE | Missing data | Excluded | Y | NI | N | N | PY | N | PN | NI | High | Y | - |
| 258 |  | MLR | 309 | ~12 | >20 | NI (likely no selection) | None | NA | None | NA |  | R2, MRDE | Missing data | Excluded | Y | NI | N | N | PY | N | PN | NI | High | Y | - |
| 259 | Wang 2018^190^ | MLR | 47 | ≥6 | <10 | Stepwise (univariate first) | None | NA | None | NA | None | R2 | None | NI | N | NI | PY | NI | N | N | N | NI | High | Y | - |
| 260 | Danese 2019^191^ | MLR | 3016 | ~15 | >20 | Based on previous studies | Log | NI | None | NA | random split | R2 | None | NI | Y | NI | PY | NI | Y | N | N | NI | High | Y | - |
| 261 |  | MLR | 534 | ~15 | >20 |  | Log | NI | None | NA | random split | R2 | None | NI | Y | NI | PY | NI | Y | N | N | NI | High | Y | - |
| 262 |  | MLR | 292 | ~15 | >20 |  | Log | NI | None | NA | random split | R2 | None | NI | Y | NI | PY | NI | Y | N | N | NI | High | Y |  |
| 263 | Roche-Lima 2019^192^ | RPART | 154 | >20 | <10 | NI | None | NA | NI | 21, 49 | Random sample split | MAE, ideal dose | Missing data | Excluded | N | NI | N | N | NI | Y | N | PY | High | Y | Doses for secondary cohort imputed |
| 264 |  | MARS | 154 | >20 | <10 | NI | None | NA | NI | 21, 49 |  |  | Missing data | Excluded | N | NI | N | N | NI | Y | N | PY | High | Y |  |
| 265 |  | RFR | 154 | >20 | <10 | NI | None | NA | NI | 21, 49 |  |  | Missing data | Excluded | N | NI | N | N | NI | Y | N | PY | High | Y |  |
| 266 |  | ANN | 154 | >20 | <10 | NI | None | NA | NI | 21, 49 |  |  | Missing data | Excluded | N | NI | N | N | NI | Y | N | PY | High | Y |  |
| 267 |  | SVR# | 154 | >20 | <10 | NI | None | NA | NI | 21, 49 |  |  | Missing data | Excluded | N | NI | N | N | NI | Y | N | PY | High | Y |  |
| 268 |  | KNN-1 | 154 | >20 | <10 | NI | None | NA | NI | 21, 49 |  |  | Missing data | Excluded | N | NI | N | N | NI | Y | N | PY | High | Y |  |
| 269 |  | KNN-2 | 154 | >20 | <10 | NI | None | NA | NI | 21, 49 |  |  | Missing data | Excluded | N | NI | N | N | NI | Y | N | PY | High | Y |  |
| 270 |  | KNN-3 | 154 | >20 | <10 | NI | None | NA | NI | 21, 49 |  |  | Missing data | Excluded | N | NI | N | N | NI | Y | N | PY | High | Y |  |
| 271 |  | REPT | 154 | >20 | <10 | NI | None | NA | NI | 21, 49 |  |  | Missing data | Excluded | N | NI | N | N | NI | Y | N | PY | High | Y |  |
| 272 | Shahabi 2019^193^ | MLR | 906 | >2423 | <10 | Stepwise (univariate first) | Log | NI | None | NA | None | R2 | Missing data and 1 outlier | Excluded | N | NI | N | N | N | N | N | NI | High | Y | Some may not yet be stable at 3 months |
| 273 | Tao 2019^194^ | Ensemble (EFCM + SVR) | 517 | ~11 | >20 | Expert opinion and literature review | None | NA | None | NA | 5-fold CV | R2, MAE, MSE, RMSE, ideal dose, 15% within actual dose | None | NI | Y | NI | PY | NI | Y | Y | PY | NA | Unclear | Y | - |
| 274 |  | CNN | 517 | ~11 | >20 |  | None | NA | None | NA | 5-fold CV |  | None | NI | Y | NI | PY | NI | Y | N | PY | NA | High | Y | - |
| 275 |  | BPN | 517 | ~11 | >20 |  | None | NA | None | NA | 5-fold CV |  | None | NI | Y | NI | PY | NI | Y | N | PY | NA | High | Y | - |
| 276 |  | GRNN | 517 | ~11 | >20 |  | None | NA | None | NA | 5-fold CV |  | None | NI | Y | NI | PY | NI | Y | N | PY | NA | High | Y | - |
| 277 |  | SVR | 517 | ~11 | >20 |  | None | NA | None | NA | 5-fold CV |  | None | NI | Y | NI | PY | NI | Y | Y | PY | NA | Unclear | Y | - |
| 278 |  | EEM | 517 | ~11 | >20 |  | None | NA | None | NA | 5-fold CV |  | None | NI | Y | NI | PY | NI | Y | Y | PY | NA | Unclear | Y | - |
| 279 |  | BRT | 517 | ~11 | >20 |  | None | NA | None | NA | 5-fold CV |  | None | NI | Y | NI | PY | NI | Y | Y | PY | NA | Unclear | Y | - |
| 280 |  | RFR | 517 | ~11 | >20 |  | None | NA | None | NA | 5-fold CV |  | None | NI | Y | NI | PY | NI | Y | N | PY | NA | High | Y | - |

**Table S6. Continued**

| **#** | **Algorithm** | **Sub-type** | **N** | **CPP** | **PCV** | **Predictor selection** | **Dose transformation** | **Reason** | **Risk groups** | **Cut-offs** | **Validation (as report**  **ed in study** | **Performance measures reported** | **Excluded participants** | **Missing data handling** | **PROBAST signaling questions** | | | | | | | | **ROB** | **Stable dose defined** | **Other key ROB concerns**  **(domains 1-3)** |
| --- | --- | --- | --- | --- | --- | --- | --- | --- | --- | --- | --- | --- | --- | --- | --- | --- | --- | --- | --- | --- | --- | --- | --- | --- | --- |
|  |  |  |  |  |  |  |  |  |  |  |  |  |  |  | **4.1** | **4.2** | **4.3** | **4.4** | **4.5** | **4.7** | **4.8** | **4.9** |  |  |  |
| 281  -  311 | Tao 2019^195^ | EEM-GP | 229 | ~10 | >20 | Expert grouping | None | NA | None | NA | 5-fold; random split | R2^c^, MAE, MSE, RMSE, ideal dose | None | NI | Y | N | PY | NI | Y | Y | PY | NA | High | Y | - |
|  |  | ε-SVR | 229 | ~10 | >20 | Expert grouping | None | NA | None | NA |  |  | None | NI | Y | N | PY | NI | Y | Y | PY | NA | High | Y | - |
|  |  | v-SVR | 229 | ~10 | >20 | Expert grouping | None | NA | None | NA |  |  | None | NI | Y | N | PY | NI | Y | Y | PY | NA | High | Y | - |
|  |  | BP | 229 | ~10 | >20 | Expert grouping | None | NA | None | NA |  |  | None | NI | Y | N | PY | NI | Y | Y | PY | NA | High | Y | - |
|  |  | GRNN | 229 | ~10 | >20 | Expert grouping | None | NA | None | NA |  |  | None | NI | Y | N | PY | NI | Y | Y | PY | NA | High | Y | - |
|  |  | EEM | 229 | ~10 | >20 | Expert grouping | None | NA | None | NA |  |  | None | NI | Y | N | PY | NI | Y | Y | PY | NA | High | Y | - |
| 312 | Yang 2019^196^ | MLR | 44 | ≥10 | <10 | Stepwise (univariate first) | None | NA | None | NA | None | R2 | Missing data | Excluded | N | NI | N | N | N | N | N | Y | High | N | - |
| 313 | Bader 2020^197^ | MLR | 104 | ≥15 | <10 | Backward elimination and stepwise (univariate first) | Log | Normalize dose | Quantiles | NI | Random sample split | R2, MAPE, r | Missing data | Excluded | N | NI | N | N | N | Y | N | Y | High | Y | - |
| 314 | Cho 2020^198^ | MLR | 109 | ≥11 | <10 | Backward | Log | Normalize dose, equalize variance | None | NA | LOOCV | R2, MPE, RMSE, r, ideal dose | None | NI | N | NI | PY | NI | NI | Y | N | Y | High | Y | - |
| 315 | Li 2020^199^ | MLR | 214 | ~25 | <10 | Stepwise (univariate first) | None | NA | None | NA | None | R2, MAE, r | NA | NI | N | NI | PY | NI | N | Y | N | NI | High | Y | - |
| 316 | Dobrzanski 1983^200^ | MLR | 100 | 2 | >20 | NI (univariate first) | None | NA | None | NA | None | R2 | None | NI | Y | NI | PY | NI | PN | N | N | NI | High | Y | - |
| 317 | Wynne 1995^201^ | MLR | 39 | ≥5 | <10 | NI (univariate first) | None | NA | None | NA | None | R2 | None | NI | N | NI | PY | NI | N | N | N | NI | High | Y | - |
| 318 | Tabrizi 2002^29^ | MLR | 153 | ≥8 | 10_20 | Backward stepwise (univariate first) | Log | Normalize dose | None | NA | None | R2 (log scale) | None | NI | NC | NI | PY | NI | N | N | N | NI | High | Y | - |
| 319 | Shine 2003^202^ | MLR | 101 | ≥30 | <10 | NI (univariate first) | None | NA | mean ± ≥3 SDs | NA | None | R2 | Missing data | Excluded | N | NI | N | N | N | N | N | N | High | Y | Possible that some patients did not achieve stable dose |
| 320 |  | MLR | 127 | ≥30 | <10 | NI (univariate first) | None | NA | mean ± ≥3 SDs | NA | None | R2 | Missing data | Excluded | N | NI | N | N | N | N | N | N | High | Y |  |
| 321 | Caldwell 2008^46^ | MLR | 1015 | ~20 | >20 | Stepwise (univariate first) | Log | Heteroscedascity, model fit | None | NA | External | R2 | None | NI | Y | NI | PY | NI | N | N | N | NI | High | Y | - |
| 322 | Gage 2008^48^ | MLR | 1051 | >1228 | <10 | Modified step-down approach | Log | NI | None | NA | None | - | Missing genotype data, outliers | Excluded | N | NI | N | N | N | N | N | NI | High | Y (for some) | - |
| 323 | IWPC 2009^3^ | MLR others | 4043 | NI | >20 | NI | Square root | Normalize dose; data-driven (tested log) | Bracketing | 21, 49 | 10-fold CV; random  split | R2, MAE, ideal dose, as diagnostics | None | Likely single imputation | Y | NI | PY | PN | NI | Y | Y | NI | High | Y | - |
| 324 | Cen 2010^66^ | MLR | 222 | ~11 | >20 | Stepwise (univariate first) | Log | Normalize dose | None | NA | None | R2 | None | NI | Y | NI | PY | NI | PN | N | N | NI | High | Y | - |
| 325 | Zambon 2011^21^ | MLR | 274 | ≤21 | >20 | Backward (univariate conducted) | Square root | NI | Bracketing) | 25, 45 | Random split | R2, ideal dose, Bland-Altman | Missing data | Excluded | Y | NI | N | N | N | N | N | Y | High | Y | - |
| 326 | Bress 2012^101^ | MLR | 258 | ~16 | 10_20 | NI (univariate first) | Log | Normalize residuals | None | NA | None | R2 | Missing data | Excluded | NC | NI | N | N | N | N | N | NI | High | Y | - |
| 327 |  | MLR | 53 | ~16 | <10 | NI (univariate first) | Log |  | None | NA | None | R2 | Missing data | Excluded | N | NI | N | N | N | N | N | NI | High | Y | - |
| 328 | Hu 2012^203^ | KNN | 587 | ~50 | 10_20 | Expert opinion and literature review | None | NA | None | NA | 10-fold CV | MAE, SD of errors | None | NI | NC | PY | PY | NI | PY | Y | PY | NA | Unclear | Y | - |
| 329 |  | SVR | 587 | ~50 | 10_20 |  | None | NA | None | NA | 10-fold CV |  | None | NI | NC | PY | PY | NI | PY | Y | PY | NA | Unclear | Y | - |
| 330 |  | MT | 587 | ~50 | 10_20 |  | None | NA | None | NA | 10-fold CV |  | None | NI | NC | PY | PY | NI | PY | Y | PY | NA | Unclear | Y | - |
| 331 |  | MLP | 587 | ~50 | 10_20 |  | None | NA | None | NA | 10-fold CV |  | None | NI | NC | PY | PY | NI | PY | Y | PY | NA | Unclear | Y | - |
| 332 |  | Bagged KNN/SVR  /MT | 587 | ~50 | 10_20 |  | None | NA | None | NA | 10-fold CV |  | None | NI | NC | PY | PY | NI | PY | Y | PY | NA | Unclear | Y | - |
| 333 |  |  | 587 | ~50 | 10_20 |  | None | NA | None | NA | 10-fold CV |  | None | NI | NC | PY | PY | NI | PY | Y | PY | NA | Unclear | Y | - |
| 334 |  |  | 587 | ~50 | 10_20 |  | None | NA | None | NA | 10-fold CV |  | None | NI | NC | PY | PY | NI | PY | Y | PY | NA | Unclear | Y | - |

**Table S6. Continued**

| **#** | **Algorithm** | **Sub-type** | **N** | **CPP** | **PCV** | **Predictor selection** | **Dose transformation** | **Reason** | **Risk groups** | **Cut-offs** | **Validation (as report**  **ed in study** | **Performance measures reported** | **Excluded participants** | **Missing data handling** | **PROBAST signaling questions** | | | | | | | | **ROB** | **Stable dose defined** | **Other key ROB concerns**  **(domains 1-3)** |
| --- | --- | --- | --- | --- | --- | --- | --- | --- | --- | --- | --- | --- | --- | --- | --- | --- | --- | --- | --- | --- | --- | --- | --- | --- | --- |
|  |  |  |  |  |  |  |  |  |  |  |  |  |  |  | **4.1** | **4.2** | **4.3** | **4.4** | **4.5** | **4.7** | **4.8** | **4.9** |  |  |  |
| 335 | Hu 2012^203^ | Bagged MLP | 587 | ~50 | 10_20 | Expert opinion and literature review | None | NA | None | NA | 10-fold CV | MAE, SD of errors | None | NI | NC | PY | PY | NI | PY | Y | PY | NA | Unclear | Y | - |
| 336 |  | Voting,4 classifiers | 587 | ~50 | 10_20 |  | None | NA | None | NA | 10-fold CV |  | None | NI | NC | PY | PY | NI | PY | Y | PY | NA | Unclear | Y | - |
| 337 |  | Bagged voting, 4 classifiers | 587 | ~50 | 10_20 | Expert opinion and literature review | None | NA | None | NA | 10-fold CV | MAE, SD of errors | None | NI | NC | PY | PY | NI | PY | Y | PY | NA | Unclear | Y | - |
| 338 | Ramirez 2012^114^ | MLR | 1167 | <30 | >20 | NI (univariate first) | Log | NI | None | NA | Bootstrap | R2, MAE, as diagnostics | Missing data | Excluded | Y | NI | N | N | PN | Y | PY | Y | High | Y | - |
| 339 | Tatarunas 2012^116^ | MLR | 189 | ~20 | <10 | Stepwise (univariate first) | None | NA | None | NA | None | R2 | None | NI | N | N | PY | NI | N | N | N | NI | High | Y | - |
| 340 |  | MLR | 189 | ~20 | <10 | Stepwise (univariate first) | None | NA | None | NA | None | R2 | None | NI | N | N | PY | NI | N | N | N | NI | High | Y | - |
| 341 | Sharabiani 2013^204^ | MLR | 235 | 11 | >20 | Stepwise (NI) | Log | Normalize dose | None | NA | Random split | MAE, RMSE, Bland-Altman | Outliers | Likely single imputation | Y | NI | N | PN | PY | Y | PN | NI | High | Y | - |
| 342 |  | ANN | 235 | 11 | >20 | All used | Log | Normalize dose | None | NA | Random split | MAE, RMSE, Bland-Altman | Outliers | Likely single imputation | Y | PY | N | PN | PY | Y | PN | NA | High | Y | - |
| 343 |  | SVR | 235 | 11 | >20 | All used | Log | Normalize dose | None | NA | Random split | MAE, RMSE, Bland-Altman | Outliers | Likely single imputation | Y | PY | N | PN | PY | Y | PN | NA | High | Y | - |
| 344 | Tatarunas 2014^145^ | MLR | 189 | ~20 | <10 | Stepwise (univariate first) | None | NA | None | NA | None | R2 | None | NI | N | NI | PY | NI | N | N | N | NI | High | N | Includes INR in predictors |
| 345 | Krishna Kumar 2014^139^ | MLR | 240 | 19 | 10_20 | Stepwise (univariate first) | Log | NI | NI | 17.5, 49 | Bootstrap | within 1 mg/d | Missing data | Excluded | NC | NI | N | N | N | N | N | Y | High | Y | - |
| 346 | Sharabiani 2015^205^ | SVM; MLR | 2119 | ~30 | >20 | Probably all variables | Log | NI | None | NA | Random split | MAE, RMSE | None | Likely single imputation | Y | NI | PY | NI | PY | Y | N | NI | High | Y | - |
| 347 | Rouleau-Mailloux 2016^167^ | MLR | 969 | <20 | >20 | Stepwise (univariate first) | Log | Normalize dose | None | NA | None | R2 | Missing data | Excluded | Y | N | N | N | N | N | N | Y | High | Y | Sub-optimal definition |
| 348- 350 | Wiley 2017^179^ | MLR | 2181 | 6 | >20 | Probably all variables | None | NA | NI | 21, 49 | Bootstrap | R2, MAE | Missing data | Excluded | Y | NI | N | N | PY | Y | NI | NI | High | Y | - |
| 351 |  | MLR | 1928 | 6 | >20 | Probably all variables | None | NA | NI | 21, 49 | Bootstrap | R2, MAE | Missing data | Excluded | Y | NI | N | N | PY | Y | NI | NI | High | Y | - |
| 352 |  | MLR | 253 | 6 | >20 | Probably all variables | None | NA | NI | 21, 49 | Bootstrap | R2, MAE | Missing data | Excluded | Y | NI | N | N | PY | Y | NI | NI | High | Y | - |
| 353-  372 | Tao 2019^195^ | EEM- GP | 229 | ~10 | >20 | Expert grouping | None | NA | None | NA | 5-fold CV; random split | R2^c^, MAE, MSE, RMSE, ideal dose | None | NI | Y | N | PY | NI | Y | Y | PY | NA | High | Y | - |
|  |  | ε-SVR | 229 | ~10 | >20 | Expert grouping | None | NA | None | NA |  |  | None | NI | Y | N | PY | NI | Y | Y | PY | NA | High | Y | - |
|  |  | v-SVR | 229 | ~10 | >20 | Expert grouping | None | NA | None | NA |  |  | None | NI | Y | N | PY | NI | Y | Y | PY | NA | High | Y | - |
|  |  | BP | 229 | ~10 | >20 | Expert grouping | None | NA | None | NA |  |  | None | NI | Y | N | PY | NI | Y | Y | PY | NA | High | Y | - |
|  |  | GRNN | 229 | ~10 | >20 | Expert grouping | None | NA | None | NA |  |  | None | NI | Y | N | PY | NI | Y | Y | PY | NA | High | Y | - |
| 373 | Asiimwe 2020 | NLS and others^d^ | 364 | 8 | >20 | Expert opinion, literature review and exhaustive search | None | Data-led (also tested log and square-root) | Bracketing, also based on a 40% difference from predicted dose | 21, 49 | Bootstrap; external | R2^c^, MAE, (exp(mean(log(predicted dose/actual dose))) – 1), unbiased MAPE, ideal dose, patients at risk of over- and under-anticoagulation | None | Multiple imputation | Y | Y | Y | Y | Y | Y | Y | Y | Low | Y | - |

**Table S6. Continued**

| **#** | **Algorithm** | **Sub-type** | **N** | **CPP** | **PCV** | **Predictor selection** | **Dose transformation** | **Reason** | **Risk groups** | **Cut-offs** | **Validation (as report**  **ed in study** | **Performance measures reported** | **Excluded participants** | **Missing data handling** | **PROBAST signaling questions** | | | | | | | | **ROB** | **Stable dose defined** | **Other key ROB concerns**  **(domains 1-3)** |
| --- | --- | --- | --- | --- | --- | --- | --- | --- | --- | --- | --- | --- | --- | --- | --- | --- | --- | --- | --- | --- | --- | --- | --- | --- | --- |
|  |  |  |  |  |  |  |  |  |  |  |  |  |  |  | **4.1** | **4.2** | **4.3** | **4.4** | **4.5** | **4.7** | **4.8** | **4.9** |  |  |  |
| 374 | Li 2020^206^ | BPNN | 9000 | 45 | >20 | Univariate | None | NA | Quantiles | 13.1, 21.9 | Random sample split, temporal | MAE, ideal dose, RMSE, MSE | None | NI | Y | PY | PY | NI | N | Y | N | NA | High | Y | - |
| 375 |  | MLR | 9000 | 45 | >20 | Forward stepwise (univariate first) | None | NA |  |  |  |  | None | NI | Y | NI | PY | NI | N | Y | N | NA | High | Y | - |
| 376 | Millican 2007^207^ | MLR | 92 | ≥10 | <10 | Stepwise (univariate first) | Log | NI | None | NA | None | R2 | None | Likely single imputation | N | NI | PY | PN | N | N | N | Y | High | Y | - |
| 377 |  | MLR | 92 | ≥10 | <10 | Stepwise (univariate first) | Log | NI | None | NA | None | R2 | None |  | N | NI | PY | PN | N | N | N | Y | High | Y | - |
| 378 | Lenzini 2008^208^ | MLR | 86 | ~17 | <10 | Backward stepwise (univariate first) | Log | Normalize dose | None | NA | External | R2 | Missing INR data | Excluded | N | NI | N | N | PN | N | N | NI | High | Y | - |
| 379 |  | MLR | 232 | ~17 | 10_20 |  | Log | Normalize dose | None | NA | External | R2, median AE |  | Excluded | NC | NI | N | N | PN | Y | N | NI | High | Y | - |
| 380 | Michaud 2008^209^ | MLR, robust regression | 132 | >20 | <10 | Backward stepwise, best subset (univariate first) | None | NA | NI | 17.5, 45.5 | None | R2 | Missing data | Excluded | N | NI | N | N | N | N | N | NI | High | Y | Possible that some did not achieve stable dose |
| 381 |  |  | 132 | >20 | <10 |  | None | NA | NI | 17.5, 45.5 | None | R2 | Missing data | Excluded | N | NI | N | N | N | N | N | NI | High | Y |  |
| 382 | Lenzini 2010^210^ | MLR | 969 | <30 | >20 | Stepwise (univariate first) | Log | To preserve linearity | None | NA | random split external | R2, median AE | Missing data | Excluded | Y | NI | N | N | PN | Y | N | NI | High | Y | - |
| 383 | Moreau 2011^88^ | MLR | 115 | ≥18 | <10 | Stepwise (univariate first) | None | NA | None | NA | None | R2, within 1 mg/d, plots inspection | None | NI | N | NI | PY | NI | N | N | N | Y | High | Y | - |
| 384 | Horne 2012^211^ | MLR | 1684 | >50 | >20 | Stepwise (univariate first) | Log | To preserve linearity | None | NA | random split  external | R2, median AE | Missing data | Excluded | Y | NI | N | N | PN | Y | PN | Y | High | Y | - |
| 385 | Ramos 2012^212^ | MLR | 163 | ~15 | 10_20 | Stepwise (univariate first) | Log | heteroscedasticity | Bracketing | 21, 49 | External | R2, MAE, MAPE, SE of estimate | Missing data, outliers | Excluded | NC | NI | N | N | N | Y | N | Y | High | Y | - |
| 386 | Xu 2012^22^ | MLR | 207 | ~21 | <10 | Stepwise (univariate first) | Square root | NI | Bracketing | 14, 28 | Random split | R2, MAE, ideal dose | Missing data | Excluded | N | NI | N | N | N | Y | N | NI | High | Y | - |
| 387 | Bosch 2014^134^ | MLR | 131 | ≥14 | <10 | Stepwise (univariate first) | Log | NS | Bracketing | 21, 49 | No validation | R2, MAE, MSE, ideal dose, SE of estimate | Missing data | Excluded | N | NI | N | N | N | Y | N | N | High | Y | - |
| 388 | Duconge 2016^213^ | MLR | 255 | ~34 | <10 | Stepwise (univariate first) | None | NA | Bracketing | 21, 49 | External | R2, MAE, SE of estimate, M% PE, ideal dose | Missing data | Excluded | N | N | N | N | N | Y | N | Y | High | Y | - |
| 389-  392 | Byrne 2000^214^ | Rev-cl | 103 | 17-25 | <10 | Literature review | NI | NA | None | NA | 10-fold cross validation | generalisation error | None | NI | N | PY | PY | NI | PY | N | PY | NA | High | N | - |
| 393 | Solomon 2004^215^ | MLR | 108 | ~12 | <10 | NI (univariate first) | None | NA | Quantile | 22.5, 50.0 | Random split | r | None | NI | N | NI | PY | NI | N | N | N | NI | High | Y | - |
| 394 |  | ANN | 108 | ~12 | <10 | NI (univariate first) | None | NA | Quantile |  |  | r | None | NI | N | PY | PY | NI | N | N | N | NA | High | Y | - |
| 395 | Lenzini 2007^216^ | MLR | 271 | ~19 | 10_20 | Stepwise (univariate first) | Log | For linearity | None | NA | External | R2 | None | NI | NC | NI | PY | NI | N | N | N | NI | High | Y | - |
| 396 |  | MLR | 271 | ~19 | 10_20 | Stepwise (univariate first) | Log | For linearity | None | NA | External |  | None | NI | NC | NI | PY | NI | N | N | N | NI | High | Y | - |
| 397 | Lenzini 2008^208^ | MLR | 531 | ~17 | >20 | Backward stepwise (univariate first) | Log | Normalize dose | None | NA | External | R2, median AE | Missing INR data | Excluded | Y | NI | N | N | PN | Y | N | NI | High | Y | - |
| 398 | Lenzini 2010^210^ | MLR | 969 | <30 | >20 | Stepwise (univariate first) | Log | Preserve linearity | None | NA | random split external | R2, median AE | Missing data | Excluded | Y | NI | N | N | PN | Y | NI | NI | High | Y | - |
| 399 | Moreau 2011^88^ | MLR | 115 | ≥18 | <10 | Stepwise (univariate first) | None | NA | None | NA | External | R2, within 1 mg/d, plots inspection | None | NI | N | NI | PY | NI | N | N | N | Y | High | Y | - |
| 400 | Horne 2012^211^ | MLR | 1684 | <50 | >20 | Stepwise (univariate first) | Log | Preserve linearity | None | NA | random split  external | R2, median AE | Missing data | Excluded | Y | NI | N | N | PN | Y | PN | Y | High | Y | - |
| 401 | Ramos 2012^212^ | MLR | 163 | ~15 | 10_20 | Stepwise (univariate first) | Log | heteroscedasticity | Bracketing | 21, 49 | External | MAE | Missing data, outliers | Excluded | NC | NI | N | N | N | Y | N | Y | High | Y | - |

**Table S6. Continued**

| **#** | **Algorithm** | **Sub-type** | **N** | **CPP** | **PCV** | **Predictor selection** | **Dose transformation** | **Reason** | **Risk groups** | **Cut-offs** | **Validation (as report**  **ed in study** | **Performance measures reported** | **Excluded participants** | **Missing data handling** | **PROBAST signaling questions** | | | | | | | | **ROB** | **Stable dose defined** | **Other key ROB concerns**  **(domains 1-3)** |
| --- | --- | --- | --- | --- | --- | --- | --- | --- | --- | --- | --- | --- | --- | --- | --- | --- | --- | --- | --- | --- | --- | --- | --- | --- | --- |
|  |  |  |  |  |  |  |  |  |  |  |  |  |  |  | **4.1** | **4.2** | **4.3** | **4.4** | **4.5** | **4.7** | **4.8** | **4.9** |  |  |  |
| 402-  405 | Zhou 2014^217^ | MLR | 820 | ~40 | >20 | Forward stepwise (univariate first) | None | NA | NI | 13.2, 21.9 | Random split | MPE, ideal dose | None | NI | Y | NI | PY | NI | N | N | N | NI | High | Y | - |
| 406-  409 |  | ANN | 820 | ~40 | >20 | Forward stepwise (univariate first) | None | NA | NI | 13.2, 21.9 | Random split | MPE, ideal dose | None | NI | Y | NI | PY | NI | N | N | N | NI | High | Y | - |
| 410 | Duconge 2016^213^ | MLR | 255 | ~34 | <10 | Stepwise (univariate first) | None | NA | Bracketing | 21, 49 | External | R2, MAE, Mean SE, M%PE, ideal dose | Missing data | Excluded | N | N | N | N | N | Y | N | Y | High | Y | - |
| 411 | Li 2018^19^ | BP | 10673 | ~661 | 10_20 | Expert knowledge, univariate screening | None | NA | Quantiles | 17.5, 21 | External | MAE, RMSE, ideal dose | Missing data | Excluded | NC | PY | N | N | N | Y | N | NA | High | Y | - |
| 412 | Sharabiani 2018^218^ | LASSO | 68 | ~57 | <10 | LASSO | None | NA | None | NA | Random split | RMSE | None | Single imputation | PY | NI | PY | N | Y | Y | PY | NI | High | N | Only patients with common doses |
| 413 | Tao 2018^219^ | ANFIS | 9000 | ~50 | >20 | All variables from the univariate analyses | None | NA | Based on warfarin origin | 13.1, 21.9 | External | MAE, MSE, r, ideal dose | None | NI | Y | PY | PY | NI | N | Y | N | NA | High | N | - |
| 414 |  | ANFIS | 3192 | ~50 | >20 |  | None | NA |  |  | External |  | None | NI | Y | PY | PY | NI | N | Y | N | NA | High | N | - |
| 415 | Lubetsky 1992^220^ | NONMEN-PKPD | 18 | ≥7 | <10 | Based on mechanistic model | None | NA | None | NA | None | R2, MAE | None | NI | PN | PY | PY | NI | NI | N | N | NA | High | Y | Possibility of above range INR |
| 416 | Hamberg 2007^221^ | NONMEM Pop PKPD | 150 | ~10 | 10_20 | Based on mechanistic model, stepwise | Log | NS | None | NA | None | Prediction in typical indivi-duals, VPCs | Missing data | Excluded | NC | PY | N | N | PY | N | N | NA | High | N | - |
| 417-  422 | Caraco 2008^222^ | NI | NI | NI | NI | NI | NI | NI | NI | NI | NI | NI | NI | NI | NI | NI | NI | NI | NI | NI | NI | NA | Unclear | NI | - |
| 423 | Linder 2009^223^ | NONMEN  MLR  PKPD | 137 | ~7 | 10_20 | Structural and based on previous model (univariate analyses conducted) | None | NA | None | NA | None | 2 case studies | Outliers and rare genotypes | NI | NC | NI | PY | NI | NI | N | N | NI | High | Y | - |
| 424 | Hamberg 2010^224^ | NONMEM  KPD | 1426 | <10 | >20 | Based on mechanistic model, stepwise | Log | NS | None | NA | None | Prediction in typical indivi-duals, VPCs | Missing data | Excluded | Y | PY | N | N | PY | N | N | NA | High | N | - |
| 425 | Gong 2011^86^ | NONMEN  MLR  PKPD | 167 | ~10 | 10_20 | NI | None | NA | None | NA | None | R2, MAE | Missing data | Excluded | NC | PY | N | N | NI | Y | N | NI | High | Y | - |
| 426 | Perlstein 2012^225^ | NONMEM PKPD | 147 | ~10 | 10_20 | Based on mechanistic model | None | NA | None | NA | None | MAE | None | NI | NC | PY | PY | NI | PY | Y | N | NA | High | Y | - |
| 427 |  |  | 79 | ~10 | <10 | Based on mechanistic model | None | NA | None | NA | None | MAE | None | NI | N | PY | PY | NI | PY | Y | N | NA | High | Y | - |
| 428 | Lala 2013^226^ | NONMEM | 26 | ≥5 | <10 | Based on adult model | NI | NA | None | NA | None | VPCs | Missing data | Excluded | N | PY | N | N | PY | N | N | NA | High | N | - |
| 429 | Lu 2013^227^ | NONMEN  IVIVE | 183 | ~6 | >20 | Base on structural model | None | NA | None | NA | None | R2, M%PE, VPCs | None | NI | Y | PY | PY | NI | PY | N | N | NA | High | Y | - |
| 430 | Hamberg 2013^109^ | KPD | See Hamberg 2007 and 2010 | | | | | | | | | VPCs | Missing data | Excluded | NC | PY | N | N | NA | N | NA | NA | High | Y | - |
| 431 | Hamberg 2014^228^ | NONMEM PKPD | 163 | ~13 | 10_20 | Stepwise | Log | NI | None | NA | None | Prediction in typical indivi-duals, VPCs | Missing data | Excluded | NC | PY | N | N | NI | N | N | NI | High | Y | - |
| 432 | Arwood 2017^229^ | MLRK-PD | 257 | 20 | 10_20 | On previous model and stepwise | Log (errors) | NI | None | NA | None |  | None | NI | NC | PY | PY | NI | NI | N | N | NA | High | N | - |
| 433 | Zhu 2017^180^ | NONMEM  Pop PKPD | 242 | ~21 | 10_20 | Based on mechanistic model | None | NA | None | NA | None | VPCs | None | NI | NC | PY | PY | NI | PY | N | N | NA | High | N | - |

^a^See Table S4 for details about these performance measures. ^b^IWPC explored many ML methods but their results were not reported. They include, artificial neural networks, support vector regression with polynomial (including linear) and Gaussian kernels, regression trees, model trees, least angle regression, LASSO and multivariate adaptive regression splines, boosting and bagging to construct ensemble predictors. ^c^R2 equation provided. ^d^Also explored ordinary least squares regression, quantile regression and weighted quantile regression. Abbreviations: AE, absolute error; AIC, Akaike Information Criteria; ANFIS, adaptive neural-fuzzy inference system; ANN, artificial neural networks; AUC, area under curve; BART, Bayesian additive regression trees; BP, back propagation neural network; BRT, Boosted Regression Tree; BSE, Bayesian structural equation; CNN, convolution neural network; CPP= candidate predictor parameters; CV, cross validation; EFCM, evolutionary fuzzy c-mean; EEM, evolutionary ensemble model; ERT, extremely randomized trees; MLR, generalized linear model; GBT, Gradient boosting trees; GP, genetic programming; GRNN, general regression neural network; GWAS, genome-wide association study; IVIVE= in vitro-in vivo extrapolation; KNN, k-nearest neighbors; KPD, kinetic-pharmacodynamic; LASSO; least absolute shrinkage and selection operator; LOOCV, leave-out-one cross validation; M%PE, mean percentage prediction error; MAE, mean absolute error; MAPE, mean absolute percentage error; MARS, multivariate adaptive regression spines; MLE= maximum likelihood estimation; MLP, multilayer perceptron; MOPSO, Multi-Objective Particle Swarm Optimization; MPE, mean prediction error; MRDE, mean relative dose error; MSE, mean square error; MT, model tree; n, number of participants; N, no; NA, not applicable; NC, not calculated; NI, no information; NLS, non-linear least squares regression; NSGA-II, Non-dominated Sorting Genetic Algorithm-II; PCV, participants per candidate predictor; PKPD, pharmacokinetic-pharmacodynamic; PN, probably no; Pop, population; PY, probably yes; r, Pearson’s or Spearman’s rank correlation coefficient; R2, R-squared (the coefficient of determination); ROC, receiver operating curve; RFR, random forest regression; RMSE, root mean square error; RR, Ridge regression; RT, regression trees; SE, standard error; SD, standard deviation; SGF, stacked generalization framework; SVR, support vector regression; VPC (visual predictive checks); Y, ‘yes’.

**Table S7. External validations**

| **#** | **Algorithm** | **Sub-type** | **Validation study** | **Country** | **Indication** | **Target INR** | **N** | **Ethnicity (%)** | | | | **Male %** | **Age, yrs (mean ± SD or median, IQR)** | **R2** | **MAE (mg/d)** | **Other** | **Excluded participants** | **Missing data** | **PROBAST** | | | | | **ROB** | **Stable dose defined** | **Other key ROB concerns**  **(domains 1-3)** |
| --- | --- | --- | --- | --- | --- | --- | --- | --- | --- | --- | --- | --- | --- | --- | --- | --- | --- | --- | --- | --- | --- | --- | --- | --- | --- | --- |
|  |  |  |  |  |  |  |  | **W** | **A** | **B** | **M** |  |  |  |  |  |  |  | **4.1** | **4.2** | **4.3** | **4.4** | **4.7** |  |  |  |
| 1 | Gage 2004^26^ | Ini-pgx | Voora 2005^230^ | USA | Orthopedic (total hip or knee arthroplasty) | 1.8–3.2 | 36 | 83 | - | 10 | 6 | 60 (of 48) | 61 ± 14  (for 48) | 42 | - | MPE= -0.6 mg/d | None | NI | N | PY | Y | NI | N | High | Y | - |
| 2 |  |  | Schelleman 2008^55^ | USA | AF, DVT, PE, stroke/TIA, CMP, other | 2.0–3.0 | 147 | 100 | - | - | - | 71 | 63 ± 14 | 21 | - | 42% within 1 mg/d | Missing data | Excluded | Y | PY | N | N | N | High | Y | - |
| 3 |  |  |  | USA | AF, DVT, PE, stroke/TIA, CMP, other | 2.0–3.0 | 112 | - | - | 100 | - | 59 | 57 ± 15 | 28 | - | 29% within 1 mg/d | Missing data | Excluded | Y | PY | N | N | N | High | Y | - |
| 4 |  |  | Wu 2008^57^ | USA | AF, DVT, PE, HV, stroke, other | 2.0–3.0 | 92 | 48 | 16 | 34 | 2 | 70 | 57 ± 13 | - | - | r=0.68 | Missing data | Excluded | N | PY | N | N | N | High | Y | - |
| 5 |  |  | Shaw 2010^231^ | USA | AF, DVT, PE, other | 2.0–3.0 | 71 | 74 | - | 4 | 21 | 45 | 60 ± 15 | 43 | 2.20 | 31% within 1 mg/d | Missing data | Excluded | N | PY | N | N | Y | High | Y | - |
| 6 | Hillman 2004^30^ | Ini-pgx | Hillman 2005^232^ | USA | AF, DVT, PE, HV, other | 1.9–3.0 | 37 | 100 | - | - | - | 45 | 70 ± 12 | - | 1.47 | - | None | NI | N | PY | PY | NI | Y | High | Y | - |
| 7 |  |  | Schelleman 2008^55^ | USA | AF, DVT, PE, stroke/TIA, CMP, other | 2.0–3.0 | 147 | 100 | - | - | - | 71 | 63 ± 14 | 6 | - | 33% within 1 mg/d | Missing data | Excluded | Y | PY | N | N | N | High | Y | - |
| 8 |  |  |  | USA | AF, DVT, PE, stroke/TIA, CMP, other | 2.0–3.0 | 112 | - | - | 100 | - | 59 | 57 ± 15 | 18 | - | 38% within 1 mg/d | Missing data | Excluded | Y | PY | N | N | N | High | Y | - |
| 9 | Sconce 2005^33^ | Ini-pgx | Sconce 2005^33^ | UK | AF, DVT, PE, other | 2.0–3.0 | 38 | 100 | - | - | - | 50 | 72 (range 39–91) | - | - | r=0.80, MPE=-0.2 mg/d | None | NI | N | PY | PY | NI | N | High | Y | - |
| 10 |  |  | Hatch 2008^233^ | Sweden | AF, PE/DVT, other | 2.0–3.0 | 88 | 100 | - | - | - | 74 | range 48–86 | 44 | - | - | None | NI | N | PY | PY | NI | N | High | Y | - |
| 11 |  |  | Wu 2008^57^ | USA | AF, DVT, PE, HV, stroke, other | 2.0–3.0 | 92 | 48 | 16 | 34 | 2 | 70 | 57 ± 13 | - | - | r=0.55 | Missing data | Excluded | N | PY | N | N | N | High | Y | - |
| 12 |  |  | Perini 2008^53^ | Brazil | AF, HV, TE, other | 2.0–3.5 | 390 | 50 | - | 20 | 30 | 48 | 54 (range 18–91) | 36 | 1.21 | M%PE=8% | None | NI | Y | PY | PY | NI | Y | Unclear | Y | - |
| 13 |  |  | Schelleman 2008^55^ | USA | AF, DVT, PE, stroke/TIA, CMP, other | 2.0–3.0 | 145 | 100 | - | - | - | 71 (of 147) | 63 ± 14 (for 147) | 38 | - | 42% within 1 mg/d | Missing data | Excluded | Y | PY | N | N | N | High | Y | - |
| 14 |  |  |  | USA | AF, DVT, PE, stroke/TIA, CMP, other | 2.0–3.0 | 109 | - | - | 100 | - | 59 (of 112) | 57 ± 15 (for 112) | 23 | - | 41% within 1 mg/d | Missing data | Excluded | Y | PY | N | N | N | High | Y | - |
| 15 |  |  | Langley 2009^234^ | USA | - | - | 75 | 81 | - | 19 | - | 63 | - | 12 | - | R2=29% with 2 outliers excluded | Missing data | Excluded | N | PY | N | N | N | High | Y | - |
| 16 |  |  | Sasaki 2009^54^ | Japan | HV, IHF | 1.5–3.8 | 45 | - | 100 | - | - | 60 | 63 ± 10 | - | - | MPE=-0.95 mg/d, RMSE=1.52 mg/d | None | NI | N | PY | PY | NI | Y | High | N | - |
| 17 |  |  | Harada 2010^69^ | Japan | HV | 2.0–3.0 | 97 | - | 100 | - | - | 53 | 63 ± 9 | 27 | - | - | Missing data | Excluded | N | PY | N | N | N | High | N | - |
| 18 |  |  | Le Cam-Duchez 2010^235^ | France | - | 2.3–3.0 | 35 | 100 | - | - | - | 29 | 46 ± 14 | - | 3.16 | - | Non-adherence | Single imputation | N | PY | PY | N | Y | High | Y | Unstable INRs likely included |
| 19 |  |  |  | France | - | 1.5–2.0 | 35 | 100 | - | - | - | 46 | 46 ± 12 | - | 0.82 | - | Non-adherence | Single imputation | N | PY | PY | N | Y | High | Y | Unstable INRs likely included |
| 20 |  |  | Lubitz 2010^35^ | USA | AF, DVT, PE, Other | 2.0–3.0 | 145 | 71 | 10 | 19 | - | 62 | 67 ± 14 | 45 | - | - | Non-compliance | NI | Y | PY | PY | NI | N | High | Y | Different outcome determination |
| 21 |  |  | Roper 2010^77^ | USA | AF, PE, DVT, CVA, HV, other | 2.0–3.0 | 121 | 100 | - | - | - | 61 (of 125) | 70 ± 13 (for 125) | 60 | 1.35 | MAPE=28%, 31% with ideal dose | Missing data | Excluded | Y | PY | N | N | Y | High | Y | - |
| 22 |  |  |  | 9 (IWPC sites) | DVT, PE, AF, HV, stroke, other | - | 974 | - | - | - | Y | - | - | 38 | 1.36 | MAPE=35%, 36% with ideal dose | Missing data | Excluded | Y | PY | N | N | Y | High | Y | - |
| 23 |  |  | Sagreiya 2010^78^ | USA | - | 2.0–3.0 | 95 | 75 | 17 | 8 | - | 58 (of 104) | 67 (53–75) (for 104) | 47 | 1.28 | - | Missing data | Excluded | N | PY | N | N | Y | High | Y | - |
| 24 |  |  | Shaw 2010^231^ | USA | AF, DVT, PE, other | 2.0–3.0 | 71 | 74 | - | 4 | 21 | 45 | 60 ± 15 | 54 | 2.3 | 39% within 1 mg/d | Missing data | Excluded | N | PY | N | N | Y | High | Y | - |
| 25 |  |  | Botton 2011^82^ | Brazil | AF, TE, HV, CVA, thrombophilia, other | 2.0–3.5 | 279 | 100 | - | - | - | 56 | 63 ± 14 | 36 | - | - | None | NI | Y | PY | PY | NI | N | High | Y | - |
| 26 |  |  | Cho 2011^25^ | Korea | AF | 2.0–3.0 | 108 | - | 100 | - | - | 64 | 67 ± 10 | - | - | r=0.626, M%PE=-55% | None | NI | Y | PY | PY | NI | N | High | Y | - |
| 27 |  |  | Shin 2011^236^ | 9 (IWPC) | DVT, PE, AF, HV, stroke, other | 2.0–3.0 | 1940 | 64 | 20 | 16 | - | 60 | In decades | - | 1.46 | 39% with ideal dose | Missing data | Excluded | Y | PY | N | N | Y | High | Y | - |
| 28 |  |  | Cini 2012^102^ | Italy | DVT, HV, PE, other | 2.0–3.5 | 40 | 100 | - | - | - | 58 | 70 ± 16 | 45 | 1.48 | - | Missing data | Excluded | N | PY | N | N | Y | High | Y | - |
| 29 |  |  | Liu 2012^237^ | China | AF, HV, DVT, PE | 1.6–2.5 | 282 | - | 100 | - | - | 50 | 56 ± 16 | - | 0.96 | 40% with ideal dose | None | NI | Y | PY | PY | NI | Y | Unclear | Y | - |
| 30 |  |  | Shalia 2012^238^ | India | HV | - | 83 | - | 100 | - | - | 73 | 44 ± 1 | 52 | - | - | None | NI | N | PY | PY | NI | N | High | Y | - |

**Table S7. Continued.**

| **#** | **Algorithm** | **Sub-type** | **Validation study** | **Country** | **Indication** | **Target INR** | **N** | **Ethnicity (%)** | | | | **Male %** | **Age, yrs (mean ± SD or median, IQR)** | **R2** | **MAE (mg/d)** | **Other** | **Excluded participants** | **Missing data** | **PROBAST** | | | | | **ROB** | **Stable dose defined** | **Other key ROB concerns**  **(domains 1-3)** |
| --- | --- | --- | --- | --- | --- | --- | --- | --- | --- | --- | --- | --- | --- | --- | --- | --- | --- | --- | --- | --- | --- | --- | --- | --- | --- | --- |
|  |  |  |  |  |  |  |  | **W** | **A** | **B** | **M** |  |  |  |  |  |  |  | **4.1** | **4.2** | **4.3** | **4.4** | **4.7** |  |  |  |
| 31 | Sconce 2005^33^ | Ini-pgx | Tan 2012^115^ | China | HV | 1.7–3.0 | 320 | - | 100 | - | - | 39 | 47 ± 11 | - | - | MPE=0.44 mg/d, 48% with ideal dose. | None | NI | Y | PY | PY | NI | N | High | Y | - |
| 32 |  |  | Francis 2014^239^ | UK | AF, VTE | 2.0–3.0 | 508 | 100 | - | - | - | 55 | 68 | 24 | 1.32 | MAPE = 37% | None | NI | Y | PY | PY | NI | Y | Unclear | Y | - |
| 33 |  |  | Francis 2014^239^ | UK/Sweden | AF, VTE | 2.0–3.0 | 133 | 100 | - | - | - | 59 | 67 | 42 | 1.28 | MAPE = 33% | None | NI | Y | PY | PY | NI | Y | Unclear | Y | - |
| 34 |  |  | Peng 2015^240^ | China | HV | 2.0–3.0 | 586 | - | 100 | - | - | 45 | 52 ± 11 | 25 | 0.98 | 36% with ideal dose | None | NI | Y | PY | PY | NI | Y | Unclear | Y | - |
| 35 |  |  | Cho 2016^162^ | Korea | Stroke | 1.5–3.0 | 101 | - | 100 | - | - | 63 | 64 ± 13 | 38 | - | M%PE=-10%, 46% with ideal dose | None | NI | Y | PY | PY | NI | N | High | Y | - |
| 36 |  |  | Dilge Taskin 2016 ^241^ (***Children***) | Turkey | CVD, thrombophilia, HV, dilated CMP, other | 2.0–3.0 | 47 | - | - | - | Y | 53 (for 58) | 13 ± 5 (for 58) | - | - | r = 0.645 | Missing data | Excluded | N | PY | N | N | N | High | Y | - |
| 37 |  |  | Yang 2016^242^ | Korea | AF, CI | - | 310 | - | 100 | - | - | - | mean 69 (26–94) | - | - | r = 0.644, MPE=-1.07, 29% with ideal dose | Missing data | Excluded | Y | PY | N | N | N | High | N | - |
| 38 |  |  | Chumnumwat 2018^91^ | Thailand | HV, AF, VTE | 2.0–3.0 | 165 | - | 100 | - | - | 38 | 50 ± 11 | - | - | MPE= -0.02 mg/d, 40% with ideal dose | None | NI | Y | PY | PY | NI | N | High | Y | - |
| 39 |  |  | Galvez 2018^183^ | Colombia | HV, DVT, PE, CVD, other | 2.0–3.0 | 87 | - | - | - | 100 | 53 | 62 ± 12 | 37 | - | - | None | NI | N | PY | PY | NI | N | High | Y | - |
| 40 |  |  | Cho 2020^198^ | Korea | AF, CI, DVT, PE, HV | - | 109 | - | 100 | - | - | 62 | 70 (range 29–91) | - | - | r = 0.471, MPE=-1.8 mg/d, RMSE = 2.48, 26% with ideal dose | None | NA | Y | PY | PY | NI | Y | Unclear | Y | - |
| 41 | Wadelius 2005^34^ | Ini-pgx | Wu 2008^57^ | USA | AF, DVT, PE, HV, stroke, other | 2.0–3.0 | 92 | 48 | 16 | 34 | 2 | 70 | 57 ± 13 | - | - | r = 0.66 | Missing data | Excluded | N | PY | N | N | N | High | Y | - |
| 42 |  |  | Lubitz 2010^35^ | USA | AF, DVT, PE, Other | 2.0–3.0 | 145 | 71 | 10 | 19 | - | 62 | 67 ± 14 | 46 | - | - | Non-compliance | NI | Y | PY | PY | NI | N | High | Y | Different outcome determination |
| 43 | Carlquist 2006^37^ | Ini-pgx | Anderson 2007^38^ | USA | AF, DVT, PE, orthopedic, other | 2.0–3.0 | 92 | 94 | - | - | - | 50 (of 101) | 63 (range 25–86) (for 101) | - | 1.01 | - | None | NI | N | PY | PY | NI | Y | High | Y | Some doses estimated |
| 44 |  |  | Schelleman 2008^55^ | USA | AF, DVT, PE, stroke/TIA, CMP, other | 2.0–3.0 | 147 | 100 | - | - | - | 71 | 63 ± 14 | 39 | - | 46% within 1 mg/d | Missing data | Excluded | Y | PY | N | N | N | High | Y | - |
| 45 |  |  |  | USA | AF, DVT, PE, stroke/TIA, CMP, other | 2.0–3.0 | 112 | - | - | 100 | - | 59 | 57 ± 15 | 32 | - | 38% within 1 mg/d | Missing data | Excluded | Y | PY | N | N | N | High | Y | - |
| 46 |  |  | Lubitz 2010^35^ | USA | AF, DVT, PE, other | 2.0–3.0 | 145 | 71 | 10 | 19 | - | 62 | 67 ± 14 | 52 | - | - | Non-compliance | NI | Y | PY | PY | NI | N | High | Y | Different outcome determination |
| 47 |  |  | Roper 2010^77^ | USA | AF, PE, DVT, CVA, HV, other | 2.0–3.0 | 121 | 100 | - | - | - | 61 (of 125) | 70 ± 13 (for 125) | 65 | 1.06 | MAPE = 24%, 52% with ideal dose | Missing data | Excluded | Y | PY | N | N | Y | High | Y | - |
| 48 |  |  |  | 9 (IWPC sites) | DVT, PE, AF, HV, stroke, other | - | 974 | - | - | - | Y | - | - | 41 | 1.26 | MAPE = 38%, 44% with ideal dose | Missing data | Excluded | Y | PY | N | N | Y | High | Y | - |
| 49 |  |  | Shin 2011^236^ | 9 (IWPC) | DVT, PE, AF, HV, stroke, other | 2.0–3.0 | 1940 | 64 | 20 | 16 | - | 60 | In decades | - | 1.31 | 47% with ideal dose | Missing data | Excluded | Y | PY | N | N | Y | High | Y | - |
| 50 |  |  | Zambon 2011^21^ | Italy | AF, DVT, other | 2.0–3.0 | 274 | 100 | - | - | - | 65 | 74 (range 39-92) | 61 | 1.16 | - | Missing data | Excluded | Y | PY | N | N | Y | High | Y | - |
| 51 |  |  |  | Italy | AF, DVT, other | 2.0–3.0 | 97 | 100 | - | - | - | 55 | 74 (range 43-87) | 56 | 1.14 | - | Missing data | Excluded | Y | PY | N | N | Y | High | Y | - |
| 52 |  |  | Cini 2012^102^ | Italy | DVT, HV, PE, other | 2.0–3.5 | 40 | 100 | - | - | - | 58 | 70 ± 16 | 56 | 1.17 | - | Missing data | Excluded | N | PY | N | N | Y | High | Y | - |
| 53 |  |  | Lei 2012^243^ | China | AF, DVT, PE, HV | 1.6–2.5 | 368 | - | 100 | - | - | 47 | 46 ± 12 | 57 | 1.09 | 70% with ideal dose | None | NI | Y | PY | PY | NI | Y | Unclear | Y | - |
| 54 |  |  | Tan 2012^115^ | China | HV | 1.7–3.0 | 320 | - | 100 | - | - | 39 | 47 ± 11 | - | - | MPE=0.83 mg/d; 36% with ideal dose. | None | NI | Y | PY | PY | NI | N | High | Y | - |
| 55 |  |  | Francis 2014^239^ | UK | AF, VTE | 2.0–3.0 | 508 | 100 | - | - | - | 55 | 68 | 37 | 1.18 | MAPE = 40% | None | NI | Y | PY | PY | NI | Y | Unclear | Y | - |
| 56 |  |  | Francis 2014^239^ | UK/Sweden | AF, VTE | 2.0–3.0 | 133 | 100 | - | - | - | 59 | 67 | 29 | 1.38 | MAPE = 32% | None | NI | Y | PY | PY | NI | Y | Unclear | Y | - |
| 57 |  |  | Peng 2015^240^ | China | HV | 2.0–3.0 | 586 | - | 100 | - | - | 45 | 52 ± 11 | 31 | 1.08 | 31% with ideal dose | None | NI | Y | PY | PY | NI | Y | Unclear | Y | - |
| 58 |  |  | Cho 2016^162^ | Korea | Stroke | 1.5–3.0 | 101 | - | 100 | - | - | 63 | 64 ± 13 | 48 | - | M%PE=4%, 50% with ideal dose | None | NI | Y | PY | PY | NI | N | High | Y | - |
| 59 | Herman 2006^39^ | Ini-pgx | Wu 2008^57^ | USA | AF, DVT, PE, HV, stroke, other | 2.0–3.0 | 92 | 48 | 16 | 34 | 2 | 70 | 57 ± 13 | - | - | r = 0.64 | Missing data | Excluded | N | PY | N | N | N | High | Y | - |

**Table S7. Continued.**

| **#** | **Algorithm** | **Sub-type** | **Validation study** | **Country** | **Indication** | **Target INR** | **N** | **Ethnicity (%)** | | | | **Male %** | **Age, yrs (mean ± SD or median, IQR)** | **R2** | **MAE (mg/d)** | **Other** | **Excluded participants** | **Missing data** | **PROBAST** | | | | | **ROB** | **Stable dose defined** | **Other key ROB concerns**  **(domains 1-3)** |
| --- | --- | --- | --- | --- | --- | --- | --- | --- | --- | --- | --- | --- | --- | --- | --- | --- | --- | --- | --- | --- | --- | --- | --- | --- | --- | --- |
|  |  |  |  |  |  |  |  | **W** | **A** | **B** | **M** |  |  |  |  |  |  |  | **4.1** | **4.2** | **4.3** | **4.4** | **4.7** |  |  |  |
| 60 | Herman 2006^39^ | Ini-pgx | Lubitz 2010^35^ | USA | AF, DVT, PE, other | 2.0–3.0 | 145 | 71 | 10 | 19 | - | 62 | 67 ± 14 | 52 |  | - | Non-compliance | NI | Y | PY | PY | NI | N | High | Y | Different outcome determination |
| 61 |  |  | Sagreiya 2010^78^ | USA | - | 2.0–3.0 | 95 | 75 | 17 | 8 | - | 58 (for 104) | 67 (53–75) (for 104) | 47 | 1.39 | - | Missing data | Excluded | N | PY | N | N | Y | High | Y | - |
| 62 |  |  | Botton 2011^82^ | Brazil | AF, TE, HV, CVA, thrombophilia, other | 2.0–3.5 | 279 | 100 | - | - | - | 56 | 63 ± 14 | 46 | - | - | None | NI | Y | PY | PY | NI | N | High | Y | - |
| 63 | Takahashi 2006^40^ | Ini-pgx | Perini 2008^53^ | Brazil | AF, HV, TE, other | 2.0–3.5 | 390 | 50 | - | 20 | 30 | 48 | 54 (range 18–91) | 39 | 1.49 | M%PE=33% | None | NI | Y | PY | PY | NI | Y | Unclear | Y | - |
| 64 |  |  | Sasaki 2009^54^ | Japan | HV, IHF | 1.5–3.8 | 45 | - | 100 | - | - | 60 | 63 ± 10 | - | - | MPE=-0.27 mg/d, RMSE=1.19 mg/d | None | NI | N | PY | PY | NI | Y | High | N | - |
| 65 |  |  | Lubitz 2010^35^ | USA | AF, DVT, PE, other | 2.0–3.0 | 145 | 71 | 10 | 19 | - | 62 | 67 ± 14 | 38 | - | - | Non-compliance | NI | Y | PY | PY | NI | N | High | Y | Different outcome determination |
| 66 |  |  | Cho 2011^25^ | Korea | AF | 2.0–3.0 | 108 | - | 100 | - | - | 64 | 67 ± 10 | - | - | r=0.674, M%PE=-8%, 42% with ideal dose | None | NI | Y | PY | PY | NI | N | High | Y | - |
| 67 |  |  | Liu 2012^237^ | China | AF, HV, DVT, PE | 1.6–2.5 | 282 | - | 100 | - | - | 50 | 56 ± 16 | - | 1.17 | 29% with ideal dose | None | NI | Y | PY | PY | NI | Y | Unclear | Y | - |
| 68 |  |  | Tan 2012^115^ | China | HV | 1.7–3.0 | 320 | - | 100 | - | - | 39 | 47 ± 11 | - | - | MPE=0.69 mg/d, 41% with ideal dose. | None | NI | Y | PY | PY | NI | N | High | Y | - |
| 69 |  |  | Yang 2016^242^ | Korea | AF, CI | - | 310 | - | 100 | - | - | - | mean 69 (range 26–94) | - | - | r = 0.712, MPE=-0.34, 49% with ideal dose | None | NI | Y | PY | N | N | N | High | N | - |
| 70 |  |  | Zhu 2017^180^ | China | HV | 1.8–2.5 | 42 | - | Y | - | - | - | - | - | - | MPE=1.64 mg/d, RMSE=1.75 mg/d | None | NI | N | PY | PY | NI | Y | High | N | - |
| 71 |  |  | Cho 2020^198^ | Korea | AF, CI, DVT, PE, HV | - | 109 | - | 100 | - | - | 62 | 70 (range 29–91) | - | - | r=0.422, MPE=-0.61 mg/d, RMSE=1.81, 61% with ideal dose | None | NI | Y | PY | PY | NI | Y | Unclear | Y | - |
| 72 | Tham 2006^41^ | Ini-pgx | Sasaki 2009^54^ | Japan | HV, IHF | 1.5–3.8 | 45 | - | 100 | - | - | 60 | 63 ± 10 | - | - | MPE=-0.95 mg/d, RMSE=1.50 mg/d | None | NI | N | PY | PY | NI | Y | High | N | - |
| 73 |  |  | Lubitz 2010^35^ | USA | AF, DVT, PE, other | 2.0–3.0 | 145 | 71 | 10 | 19 | - | 62 | 67 ± 14 | 48 | - | - | Non-compliance | NI | Y | PY | PY | NI | N | High | Y | Different outcome determination |
| 74 |  |  | Sagreiya 2010^78^ | USA | - | 2.0–3.0 | 95 | 75 | 17 | 8 | - | 58 (for 104) | 67 (53–75) (for 104) | 33 | 1.39 | - | Missing data | Excluded | N | PY | N | N | Y | High | Y | - |
| 75 |  |  | Shin 2011^236^ | 9 (IWPC) | DVT, PE, AF, HV, stroke, other | 2.0–3.0 | 1940 | 64 | 20 | 16 | - | 60 | In decades | - | 2.29 | 19% with ideal dose | Missing data | Excluded | Y | PY | N | N | Y | High | Y | - |
| 76 |  |  | Lei 2012^243^ | China | AF, DVT, PE, HV | 1.6–2.5 | 368 | - | 100 | - | - | 47 | 46 ± 12 | 40 | 1.84 | 46% with ideal dose | None | NI | Y | PY | PY | NI | Y | Unclear | Y | - |
| 77 |  |  | Chumnumwat 2018^91^ | Thailand | HV, AF, VTE | 2.0–3.0 | 165 | - | 100 | - | - | 38 | 50 ± 11 |  |  | MPE=1.61 mg/d, 27% with ideal dose | None | NI | Y | PY | PY | NI | N | High | Y | - |
| 78 |  |  | Syn 2018^244^ | Singapore  Malaysia | AF, DVT, PE, stroke, other | 1.9–3.1 | 103 | - | 100 | - | - | 58 (of 322) | 59 ± 14 (of 322) | 42 | - | RMSE=1.10 mg/d, M%PE=-7%. | None | NI | Y | PY | PY | NI | Y | Unclear | Y | - |
| 79 | Anderson 2007^38^ | Ini-pgx | Shin 2011^236^ | 9 (IWPC) | DVT, PE, AF, HV, stroke, other | 2.0–3.0 | 1940 | 64 | 20 | 16 | - | 60 | In decades | - | 1.33 | 47% with ideal dose | Missing data | Excluded | Y | PY | N | N | Y | High | Y | - |
| 80 |  |  | Cho 2020^198^ | Korea | AF, CI, DVT, PE, HV | - | 109 | - | 100 | - | - | 62 | 70 (range 29–91) | - | - | r=0.574, MPE=-1.47 mg/d, RMSE=2.15, 43% with ideal dose | None | NI | Y | PY | PY | NI | Y | Unclear | Y | - |
| 81 | Miao 2007^43^ | Ini-pgx | Lubitz 2010^35^ | USA | AF, DVT, PE, other | 2.0–3.0 | 145 | 71 | 10 | 19 | - | 62 | 67 ± 14 | 37 | - | - | Non-compliance | NI | Y | PY | PY | NI | N | High | Y | Different outcome determination |
| 82 |  |  | Botton 2011^82^ | Brazil | AF, TE, HV, CVA, thrombophilia, other | 2.0–3.5 | 279 | 100 | - | - | - | 56 | 63 ± 14 | 36 | - | - | None | NI | Y | PY | PY | NI | N | High | Y | - |

**Table S7. Continued.**

| **#** | **Algorithm** | **Sub-type** | **Validation study** | **Country** | **Indication** | **Target INR** | **N** | **Ethnicity (%)** | | | | **Male %** | **Age, yrs (mean ± SD or median, IQR)** | **R2** | **MAE (mg/d)** | **Other** | **Excluded participants** | **Missing data** | **PROBAST** | | | | | **ROB** | **Stable dose defined** | **Other key ROB concerns**  **(domains 1-3)** |
| --- | --- | --- | --- | --- | --- | --- | --- | --- | --- | --- | --- | --- | --- | --- | --- | --- | --- | --- | --- | --- | --- | --- | --- | --- | --- | --- |
|  |  |  |  |  |  |  |  | **W** | **A** | **B** | **M** |  |  |  |  |  |  |  | **4.1** | **4.2** | **4.3** | **4.4** | **4.7** |  |  |  |
| 83 | Miao 2007^43^ | Ini-pgx | Shin 2011^236^ | 9 (IWPC) | DVT, PE, AF, HV, stroke, other | 2.0–3.0 | 1940 | 64 | 20 | 16 | - | 60 | In decades | - | 1.67 | 32% with ideal dose | Missing data | Excluded | Y | PY | N | N | Y | High | Y | - |
| 84 |  |  | Lei 2012^243^ | China | AF, DVT, PE, HV | 1.6–2.5 | 368 | - | 100 | - | - | 47 | 46 ± 12 | 38 | 1.89 | 26% with ideal dose | None | NI | Y | PY | PY | NI | Y | Unclear | Y | - |
| 85 |  |  | Tan 2012^115^ | China | HV | 1.7–3.0 | 320 | - | 100 | - | - | 39 | 47 ± 11 | - | - | MPE=-1.09 mg/d, 19% with ideal dose. | None | NI | Y | PY | PY | NI | N | High | Y | - |
| 86 |  |  | Zhao 2014^68^ | China | HV | 1.5–2.5 | 122 | - | 100 | - | - | 31 | 50 ± 10 | - | - | MPE=0.19 mg/d, 64% with ideal dose | None | NI | Y | PY | PY | NI | N | High | Y | - |
| 87 |  |  | Peng 2015^240^ | China | HV | 2.0–3.0 | 586 | - | 100 | - | - | 45 | 52 ± 11 | 30 | 0.92 | 31% with ideal dose | None | NI | Y | PY | PY | NI | Y | Unclear | Y | - |
| 88 |  |  | Xu 2015^245^ | China | HV | 1.8–2.5 | 59 | - | 100 | - | - | 47 (of 193) | 53 ± 12 (for 193) | - | 1.30 | 27% with ideal dose | Loss to follow-up | NI | N | PY | PY | NI | Y | High | Y | - |
| 89 |  |  | Lin 2016^27^ | China | HV | 1.5–2.5 | 208 | - | 100 | - | - | - | - | 34 | 1.09 | MAPE=33%, 51% within 1mg/d, 25% with ideal dose | None | NI | Y | PY | PY | NI | Y | Unclear | Y | - |
| 90 |  |  | Zhu 2017^180^ | China | HV | 1.8–2.5 | 42 | - | Y | - | - | - | - | - | - | MPE=-0.28 mg/d, RMSE=0.55 mg/d | None | NI | N | PY | PY | NI | Y | High | N | - |
| 91 |  |  | Chumnumwat 2018^91^ | Thailand | HV, AF, VTE | 2.0–3.0 | 165 | - | 100 | - | - | - | 38 | - | - | MPE=-1.16 mg/d, 27% with ideal dose | None | NI | Y | PY | PY | NI | N | High | Y | - |
| 92 |  |  | Dong 2019^246^ | China | AF, PE, DVT, other | 1.6–2.8 | 119 | - | 100 | - | - | 51 | 73 (range 34–90) | - | - | r=0.45, MPE=-0.80 mg/d, RMSE=1.30, 22% with ideal dose | Missing genotype data | Excluded | Y | PY | PN | PN | Y | High | Y | - |
| 93 |  |  | Tao 2019^194^ | China | AF, HV, DVT, PE, other | 2.0–3.0 | 517 | - | 100 | - | - | 57 | - | 33 | - | 29% patients with ideal dose | None | NI | Y | PY | PY | NI | N | High | Y | - |
| 94 |  |  | Cho 2020^198^ | Korea | AF, CI, DVT, PE, HV | - | 109 | - | 100 | - | - | 62 | 70 (range 29–91) | - | - | r=0.141, MPE=-1.97 mg/d, RMSE = 2.88, 17% with ideal dose | None | NI | Y | PY | PY | NI | Y | Unclear | Y | - |
| 95 |  |  | Xie 2020^141^ | China | HV, AF, other | 1.5–3.0. | 325 | - | 100 | - | - | 51 | 57 ± 13 | 41 | - | Ideal dose = 41%, MPE = -0.2 mg/d | NA | NI | Y | PY | PY | NI | N | High | Y | - |
| 96 | Zhu 2007^45^ | Ini-pgx | Perini 2008^53^ | Brazil | AF, HV, TE, other | 2.0–3.5 | 390 | 50 | - | 20 | 30 | 48 | 54 (range 18–91) | 36 | 1.31 | M%PE=22% | None | NI | Y | PY | PY | NI | Y | Unclear | Y | - |
| 97 |  |  | Wu 2008^57^ | USA | AF, DVT, PE, HV, stroke, other | 2.0–3.0 | 92 | 48 | 16 | 34 | 2 | 70 | 57 ± 13 | - | - | r = 0.66 | Missing data | Excluded | N | PY | N | N | N | High | Y | - |
| 98 |  |  | Langley 2009^234^ | USA | - | - | 75 | 81 | - | 19 | - | 63 | - | 9 | - | R2=31% with 2 outliers excluded | Missing data | Excluded | N | PY | N | N | N | High | Y | - |
| 99 |  |  | Linder 2009^223^ | USA | - | 2.0–3.0 | 137 | Y | - | - | - | 58 | 75 (67–80) | 58 | - | - | Outliers, rare genotypes | NI | Y | PY | PY | NI | N | High | Y | - |
| 100 |  |  | Sasaki 2009^54^ | Japan | HV, IHF | 1.5–3.8 | 45 | - | 100 | - | - | 60 | 63 ± 10 | - | - | MPE=-1.43 mg/d, RMSE=1.90 mg/d | None | NI | N | PY | PY | NI | Y | High | N | - |
| 101 |  |  | Lubitz 2010^35^ | USA | AF, DVT, PE, other | 2.0–3.0 | 145 | 71 | 10 | 19 | - | 62 | 67 ± 14 | 51 | - | - | Non-compliance | NI | Y | PY | PY | NI | N | High | Y | Different outcome determination |
| 102 |  |  | Sagreiya 2010^78^ | USA | - | 2.0–3.0 | 95 | 75 | 17 | 8 | - | 58 (of 104) | 67 (53–75) (for 104) | 39 | 1.28 | - | Missing data | Excluded | N | PY | N | N | Y | High | Y | - |
| 103 |  |  | Shaw 2010^231^ | USA | AF, DVT, PE, other | 2.0–3.0 | 71 | 74 | - | 4 | 21 | 45 | 60 ± 15 | 61 | 1.7 | 37% within 1 mg/d | Missing data | Excluded | N | PY | N | N | Y | High | Y | - |
| 104 |  |  | Botton 2011^82^ | Brazil | AF, TE, HV, CVA, thrombophilia, other | 2.0–3.5 | 279 | 100 | - | - | - | 56 | 63 ± 14 | 48 | - | - | None | NI | Y | PY | PY | NI | N | High | Y | - |
| 105 |  |  | Cho 2011^25^ | Korea | AF | 2.0–3.0 | 108 | - | 100 | - | - | 64 | 67 ± 10 | - | - | r=0.593, M%PE=-41% | None | NI | Y | PY | PY | NI | N | High | Y | - |
| 106 |  |  | Shin 2011^236^ | 9 (IWPC) | AF, DVT, PE, HV, stroke, other | 2.0–3.0 | 1940 | 64 | 20 | 16 | - | 60 | In decades | - | 1.43 | 45% with ideal dose | Missing data | Excluded | Y | PY | N | N | Y | High | Y | - |
| 107 |  |  | Cini 2012^102^ | Italy | DVT, HV, PE, other | 2.0–3.5 | 40 | 100 | - | - | - | 58 | 70 ± 16 | 49 | 2.46 | - | Missing data | Excluded | N | PY | N | N | Y | High | Y | - |

**Table S7. Continued.**

| **#** | **Algorithm** | **Sub-type** | **Validation study** | **Country** | **Indication** | **Target INR** | **N** | **Ethnicity (%)** | | | | **Male %** | **Age, yrs (mean ± SD or median, IQR)** | **R2** | **MAE (mg/d)** | **Other** | **Excluded participants** | **Missing data** | **PROBAST** | | | | | **ROB** | **Stable dose defined** | **Other key ROB concerns**  **(domains 1-3)** |
| --- | --- | --- | --- | --- | --- | --- | --- | --- | --- | --- | --- | --- | --- | --- | --- | --- | --- | --- | --- | --- | --- | --- | --- | --- | --- | --- |
|  |  |  |  |  |  |  |  | **W** | **A** | **B** | **M** |  |  |  |  |  |  |  | **4.1** | **4.2** | **4.3** | **4.4** | **4.7** |  |  |  |
| 108 | Zhu 2007^45^ | Ini-pgx | Tan 2012^115^ | China | HV | 1.7–3.0 | 320 | - | 100 | - | - | 39 | 47 ± 11 | - | - | MPE=0.2 mg/d; 54% with ideal dose. | None | NI | Y | PY | PY | NI | N | High | Y | - |
| 109 |  |  | Francis 2014^239^ | UK | AF, VTE | 2.0–3.0 | 508 | 100 | - | - | - | 55 | 68 | 37 | 1.29 | MAPE = 32% | None | NI | Y | PY | PY | NI | Y | Unclear | Y | - |
| 110 |  |  | Francis 2014^239^ | UK/Sweden | AF, VTE | 2.0–3.0 | 133 | 100 | - | - | - | 59 | 67 | 37 | 1.6 | MAPE = 30% | None | NI | Y | PY | PY | NI | Y | Unclear | Y | - |
| 111 |  |  | Peng 2015^240^ | China | HV | 2.0–3.0 | 586 | - | 100 | - | - | 45 | 52 ± 11 | 36 | 0.89 | 39% with ideal dose | None | NI | Y | PY | PY | NI | Y | Unclear | Y | - |
| 112 | Caldwell 2008^46^ | Ini-pgx | Burmester 2011^47^ | USA | AF, DVT, PE, HV | 2.0–3.5 | 91 | 100 | - | - | - | 57 (of 115) | 67 (range 40–90) (for 115) | - | 0.8 median | - | None | NI | N | PY | PY | NI | Y | High | Y | - |
| 113 | Gage 2008^a48^ | Ini-pgx | Gage 2008^48^ | USA | AF, VTE, HV | 1.5–3.2 | 292 | 83 | - | 15 | 2 | 48 | 57 ± 14 | 54 | 1.3 | - | None | NA | Y | PY | PY | NI | Y | Unclear | Y | - |
| 114 |  |  | Schelleman 2008^55^ | USA | AF, DVT, PE, stroke/TIA, CMP, other | 2.0–3.0 | 145 | 100 | - | - | - | 71 (of 147) | 63 ± 14 (for 147) | 42 | - | 48% within 1 mg | Missing data | Excluded | Y | PY | N | N | N | High | Y | - |
| 115 |  |  | Schelleman 2008^55^ | USA | AF, DVT, PE, stroke/TIA, CMP, other | 2.0–3.0 | 109 | - | - | 100 | - | 59 (of 112) | 57 ± 15 (for 112) | 34 | - | 34% within 1 mg | Missing data | Excluded | Y | PY | N | N | N | High | Y | - |
| 116 |  |  | Langley 2009^234^ | USA | - | - | 75 | 81 | - | 19 | - | 63 | - | 13 | - | R2 = 27% with 2 outliers excluded | Missing data | Excluded | N | PY | N | N | N | High | Y | - |
| 117 |  |  | Le Cam-Duchez 2010^235^ | France | - | 2.3–3.0 | 35 | 100 | - | - | - | 29 | 46 ± 14 | - | 2.47 | - | Non-adherence | Single imputation | N | PY | PY | N | Y | High | Y | Unstable INRs likely included |
| 118 |  |  | Le Cam-Duchez 2010^235^ | France | - | 2.3–3.0 | 35 | 100 | - | - | - | 46 | 46 ± 12 | - | 0.99 | - | Non-adherence | Single imputation | N | PY | PY | N | Y | High | Y | Unstable INRs likely included |
| 119 |  |  | Lubitz 2010^35^ | USA | AF, DVT, PE, other | 2.0–3.0 | 145 | 71 | 10 | 19 | - | 62 | 67 ± 14 | 54 | - | - | Non-compliance | NI | Y | PY | PY | NI | N | High | Y | Different outcome determination |
| 120 |  |  | Roper 2010^77^ | USA | AF, PE, DVT, CVA, HV, other | 2.0–3.0 | 121 | 100 | - | - | - | 61 (of 125) | 70 ± 13 (for 125) | 64 | 1.04 | MAPE = 24%, 54% with ideal dose | Missing data | Excluded | Y | PY | N | N | Y | High | Y | - |
| 121 |  |  | Roper 2010^77^ | 9 (IWPC sites) | AF, DVT, PE, HV, stroke, other | - | 974 | - | - | - | Y | - | - | 46 | 1.20 | MAPE=35%, 45% with ideal dose | Missing data | Excluded | Y | PY | N | N | Y | High | Y | - |
| 122 |  |  | Sagreiya 2010^78^ | USA | - | 2.0–3.0 | 95 | 75 | 17 | 8 | - | 58 (of 104) | 67 (53–75) (for 104) | 49 | 1.11 | - | Missing data | Excluded | N | PY | N | N | Y | High | Y | - |
| 123 |  |  | Shaw 2010^231^ | USA | AF, DVT, PE, other | 2.0–3.0 | 71 | 74 | - | 4 | 21 | 45 | 60 ± 15 | 66 | 1.8 | 38% within 1 mg/d | Missing data | Excluded | N | PY | N | N | Y | High | Y | - |
| 124 |  |  | Botton 2011^82^ | Brazil | AF, TE, HV, CVA, thrombophilia, other | 2.0–3.5 | 279 | 100 | - | - | - | 56 | 63 ± 14 | 42 | - | - | None | NI | Y | PY | PY | NI | N | High | Y | - |
| 125 |  |  | Cavallari 2011^83^ | USA | VTE, AF, stroke, HV, other | 2.0–3.5 | 50 | 100 | - | - | - | 36 | 57 ± 15 | 49 | 1.2 median | 40% within 1 mg/d | None | NI | N | PY | PY | NI | Y | High | Y | - |
| 126 |  |  | Cho 2011^25^ | Korea | AF | 2.0–3.0 | 108 | - | 100 | - | - | 64 | 67 ± 10 |  |  | r = 0.193, M%PE=48% | None | NI | Y | PY | PY | NI | N | High | Y | - |
| 127 |  |  | Finkelman 2011^247^ | 9 (IWPC sites) | AF, DVT, PE, HV, stroke, other | mainly 2.0–3.0 | 1378 | - | - | - | Y | - | - | - | - | 52% with ideal dose | Missing data | Excluded | Y | PY | N | N | N | High | Y | - |
| 128 |  |  | Schwartz 2011^248^ | USA | AF, DVT, PE, other | 2.0–3.0 | 69 | 83 | 14 | 3 | - | 46 | 81 ± 8 (all <65) | 50 | - | - | None | NI | N | PY | PY | NI | N | High | Y | - |
| 129 |  |  | Shin 2011^236^ | 9 (IWPC) | AF, DVT, PE, HV, stroke, other | 2.0–3.0 | 1940 | 64 | 20 | 16 | - | 60 | In decades | - | 1.21 | 51% with ideal dose | Missing data | Excluded | Y | PY | N | N | Y | High | Y | - |
| 130 |  |  | Bazan 2012^249^ | Egypt | AF, ATE, HV | 2.0–3.5 | 63 | - | - | - | 100 | 49 | 46 ± 13 | 42 | 3.30 | 27% within 1 mg/d | None | NI | N | PY | PY | NI | Y | High | Y | - |
| 131 |  |  | Cini 2012^102^ | Italy | DVT, HV, PE, other | 2.0–3.5 | 40 | 100 | - | - | - | 58 | 70 ± 16 | 54 | 1.10 | - | Missing data | Excluded | N | PY | N | N | Y | High | Y | - |
| 132 |  |  | Lei 2012^243^ | China | AF, DVT, PE, HV | 1.6–2.5 | 368 | - | 100 | - | - | 47 | 46 ± 12 | 58 | 1.01 | 69% with ideal dose | None | NI | Y | PY | PY | NI | Y | Unclear | Y | - |
| 133 |  |  | Liu 2012^237^ | China | AF, HV, DVT, PE | 1.6–2.5 | 282 | - | 100 | - | - | 50 | 56 ± 16 |  | 0.74 | 49% with ideal dose | None | NI | Y | PY | PY | NI | Y | Unclear | Y | - |
| 134 |  |  | Marin-Leblanc 2012^250^ | Canada | AF, HV | 2.0–3.5 | 605 | Y | - | - | - | 68 | 67 ± 11 | 44 | 1.43 | 42% with ideal dose | None | NI | Y | PY | PY | NI | Y | Unclear | Y | - |
| 135 |  |  | Tan 2012^115^ | China | HV | 1.7–3.0 | 320 | - | 100 | - | - | 39 | 47 ± 11 | - | - | MPE=0.07 mg/d; 57% with ideal dose. | None | NI | Y | PY | PY | NI | N | High | Y | - |
| 136 |  |  | Ekladious 2013^123^ | Egypt | AF, DVT, HV, coronary heart disease | 2.0–3.0 | 35 | - | - | - | 100 | - | - | - | - | r = 0.276 | Missing data | Excluded | N | PY | N | N | N | High | Y | - |
| 137 |  |  | Kimmel 2013^251^  French 2016^252^ | USA | AF, DVT, PE, other | 2.0–3.0 | 370 | 77 | - | 23 | - | 53 (of 514) | 59 (48–70) (for 514) | 48 | 1.3 | r=0.72, 53% within 1 mg/d | None | NI | Y | PY | PY | PY | Y | Low | Y | - |

**Table S7. Continued.**

| **#** | **Algorithm** | **Sub-type** | **Validation study** | **Country** | **Indication** | **Target INR** | **N** | **Ethnicity (%)** | | | | **Male %** | **Age, yrs (mean ± SD or median, IQR)** | **R2** | **MAE (mg/d)** | **Other** | **Excluded participants** | **Missing data** | **PROBAST** | | | | | **ROB** | **Stable dose defined** | **Other key ROB concerns**  **(domains 1-3)** |
| --- | --- | --- | --- | --- | --- | --- | --- | --- | --- | --- | --- | --- | --- | --- | --- | --- | --- | --- | --- | --- | --- | --- | --- | --- | --- | --- |
|  |  |  |  |  |  |  |  | **W** | **A** | **B** | **M** |  |  |  |  |  |  |  | **4.1** | **4.2** | **4.3** | **4.4** | **4.7** |  |  |  |
| 138 | Gage 2008^a48^ | Ini-pgx | Supe 2014^253^ | Croatia | Stroke | 2.0–3.0 | 106 | 100 | - | - | - | 43 | mean 68 (95% CI, 66–70) | - | - | r = 0.031 | None | NI | Y | PY | PY | NI | N | High | Y | - |
| 139 |  |  | Zhao 2014^68^ | China | HV | 1.5–2.5 | 122 | - | 100 | - | - | 31 | 50 ± 10 | - | - | MPE=0.38 mg/d, 53% with ideal dose | None | NI | Y | PY | PY | NI | N | High | Y | - |
| 140 |  |  | Drozda 2015^150^ | USA | AF, DVT, PE, stroke, HV, other | - | 274 | - | - | 100 | - | 28 | 55 ± 16 | - | - | Median prediction error of 0.5 and 2.2 in CYP2C9*5, *6, *8, *11 non-carriers and carriers respectively | None | NI | Y | PY | PY | NI | N | High | Y | - |
| 141 |  |  | Karaca 2015^154^ | Turkey | AF, HV, CVA, DVT, PE, other | - | 97 | - | - | - | 100 | 49 | 61 ± 13 | - | 2.25 | r = 0.558, 29% with ideal dose | None | NI | N | PY | PY | NI | Y | High | N | - |
| 142 |  |  | Karaca 2015^154^ | Turkey | AF, HV, CVA, DVT, PE, other | - | 92 | - | - | - | 100 | 51 | 67 ± 12 | - | 1.65 | r = 0.417, 36% with ideal dose | None | NI | N | PY | PY | NI | Y | High | N | - |
| 143 |  |  | Saffian 2015^254^ | New  Zealand | AF, DVT, PE | 2.0–3.0 | 46 | Y | - | - | - | 41 | 62 (range 29–87) | - | - | MPE=-0.64 mg/d, RMSE=1.6 mg/d | Missing geno-type data | Likely single imputation | N | PY | N | N | Y | High | Y | - |
| 144 |  |  | Xu 2015^245^ | China | HV | 1.8–2.5 | 59 | - | 100 | - | - | 47 (of 193) | 53 ± 12 (for 193) | - | 0.77 | 51% with ideal dose | Loss to follow-up | NI | N | PY | PY | NI | Y | High | Y | - |
| 145 |  |  | Cho 2016^162^ | Korea | Stroke | 1.5–3.0 | 101 | - | 100 | - | - | 63 | 64 ± 13 | 33 | - | M%PE=-7% | None | NI | Y | PY | PY | NI | N | High | Y | - |
| 146 |  |  | Wypasek 2016^255^ | Poland | HV | 2.0–3.0 | 200 | Y | - | - | - | 65 | 63 ± 12 | - | - | 49% with ideal dose | Missing data | Excluded | Y | PY | N | N | N | High | N | - |
| 147 |  |  | Yang 2016^242^ | Korea | AF, CI | NA | 310 | - | 100 | - | - | NA | mean 69 (26–94) | - | - | r=0.715, MPE=-0.73, 43% with ideal dose | Missing data | Excluded | Y | PY | N | N | N | High | N | - |
| 148 |  |  | Chumnumwat 2018^91^ | Thailand | HV, AF, VTE | 2.0–3.0 | 165 | - | 100 | - | - | 38 | 50 ± 11 | - | - | Prediction error of -0.04 mg/d; 50% with ideal dose | None | NI | Y | PY | PY | NI | N | High | Y | - |
| 149 |  |  | Selim 2018^187^ | Egypt | AF, DVT, PE, HV | 2.0–3.2 | 100 | - | - | - | 100 | 46 | 45 ± 13 | 50 | - | 65% within 1 mg/d | None | NI | Y | PY | PY | NI | N | High | Y | - |
| 150 |  |  | Bairova 2019^256^ | Russia | AF, CAD, thrombosis, other | 2.0–3.0 | 114 | 58 | 42 | - | - | 54 | 61 ± 12 | - | 1.25 | 42% with ideal dose | None | NI | Y | PY | PY | NI | Y | Unclear | Y | - |
| 151 |  |  | Danese 2019^191^ | 17 | AF, CMP, orthopedic, other | 1.5–4.0 | 938 | 100 | - | - | - | 54 (of 8806) | 62 ± 16 (for 8806) | 43 | - | - | None | NI | Y | PY | PY | NI | N | High | Y | - |
| 152 |  |  | Danese 2019^191^ | 17 | AF, CMP, orthopedic, other | 1.5–4.0 | 187 | - | - | 100 | - | - | - | 22 | - | - | None | NI | Y | PY | PY | NI | N | High | Y | - |
| 153 |  |  | Danese 2019^191^ | 17 | AF, CMP, orthopedic, other | 1.5–4.0 | 86 | - | 100 | - | - | - | - | 42 | - | - | None | NI | N | PY | PY | NI | N | High | Y | - |
| 154 |  |  | Dong 2019^246^ | China | AF, PE, DVT, other | 1.6–2.8 | 119 | - | 100 | - | - | 51 | 73 (range 34–90) | - | - | r=0.54, MPE=0.08 mg/d, RMSE = 0.91, 58% with ideal dose | Missing genotype data | Excluded | Y | PY | PN | PN | Y | High | Y | - |
| 155 |  |  | Helin 2019^257^ | Finland | Thrombosis, HV | 2.0–3.5 | 50 | Y | - | - | - | 32 | mean 47 (range 20-76) | 26 | 1.2 median) | - | None | NI | N | PY | PY | NI | Y | High | N | - |
| 156 |  |  | Makohusová 2019^258^ | Slovakia | AF, VTE | 2.0–3.0 | 47 | Y | - | - | - | 60 | 68 (range 25–84) | - | - | r = 0.743 | None | NI | N | PY | PY | NI | N | High | N | - |
| 157 |  |  | Tao 2019^194^ | China | AF, HV, DVT, PE, other | 2.0–3.0 | 517 | - | 100 | - | - | 57 | - | 14 | 0.83 | 36% patients with ideal dose | None | NI | Y | PY | PY | NI | Y | Unclear | Y | - |
| 158 |  |  | Cho 2020^198^ | Korea | AF, CI, DVT, PE, HV | - | 109 | - | 100 | - | - | 62 | 70 (range 29–91) | - | - | r=0.523, MPE = -1.34 mg/d, RMSE = 2.08, 47% with ideal dose | None | NA | Y | PY | PY | NI | Y | Unclear | Y | - |
| 159 | Haug 2008^49^ | Ini-pgx | Harada 2010^69^ | Japan | HV | 2.0–3.0 | 97 | - | 100 | - | - | 53 | 63 ± 9 | 15 | - | - | Missing data | Excluded | N | PY | N | N | N | High | N | - |

**Table S7. Continued.**

| **#** | **Algorithm** | **Sub-type** | **Validation study** | **Country** | **Indication** | **Target INR** | **N** | **Ethnicity (%)** | | | | **Male %** | **Age, yrs (mean ± SD or median, IQR)** | **R2** | **MAE (mg/d)** | **Other** | **Excluded participants** | **Missing data** | **PROBAST** | | | | | **ROB** | **Stable dose defined** | **Other key ROB concerns**  **(domains 1-3)** |
| --- | --- | --- | --- | --- | --- | --- | --- | --- | --- | --- | --- | --- | --- | --- | --- | --- | --- | --- | --- | --- | --- | --- | --- | --- | --- | --- |
|  |  |  |  |  |  |  |  | **W** | **A** | **B** | **M** |  |  |  |  |  |  |  | **4.1** | **4.2** | **4.3** | **4.4** | **4.7** |  |  |  |
| 160 | Oner Ozgon 2008^52^ | Ini-pgx | Shin 2011^236^ | 9 (IWPC) | DVT, PE, AF, HV, stroke, other | 2.0–3.0 | 1940 | 64 | 20 | 16 | - | 60 | In decades | - | 1.54 | 40% with ideal dose | Missing data | Excluded | Y | PY | N | N | Y | High | Y | - |
| 161 | Perini 2008^53^ | Ini-pgx | Botton 2011^82^ | Brazil | AF, TE, HV, CVA, thrombophilia, other | 2.0–3.5 | 279 | 100 | - | - | - | 56 | 63 ± 14 | 41 | - | - | None | NI | Y | PY | PY | NI | N | High | Y | - |
| 162 |  |  | Cho 2011^25^ | Korea | AF | 2.0–3.0 | 108 | - | 100 | - | - | 64 | 67 ± 10 | - | - | r=0.676, M%PE=-76% | None | NI | Y | PY | PY | NI | N | High | Y | - |
| 163 |  |  | Cini 2012^102^ | Italy | DVT, HV, PE, other | 2.0–3.5 | 40 | 100 | - | - | - | 58 | 70 ± 16 | 50 | 2.91 | - | Missing data | Excluded | N | PY | N | N | Y | High | Y | - |
| 164 |  |  | Santos 2015^160^,  Tavares 2018^189^ | Brazil | AF, CVA, TE, HV, other | 1.8–3.2 | 133 | 56 | - | 17 | 26 | 56 | 63 ± 17 | 49 | - | r = 0.4063 | None | NI | Y | PY | PY | NI | N | High | Y | - |
| 165 |  |  | Yang 2016^242^ | Korea | AF, CI | - | 310 | - | 100 | - | - | - | mean 69 (26–94) | - | - | r=0.686, MPE=-2.87, 2% with ideal dose | Missing data | Excluded | Y | PY | N | N | N | High | N | - |
| 166 |  |  | Galvez 2018^183^ | Colombia | HV, DVT, PE, CVD, other | 2.0–3.0 | 87 | - | - | - | 100 | 53 | 62 ± 12 | 40 | - | - | None | NI | N | PY | PY | NI | N | High | Y | - |
| 167 |  |  | Cho 2020^198^ | Korea | AF, CI, DVT, PE, HV | - | 109 | - | 100 | - | - | 62 | 70 (range 29–91) | - | - | r=0.437, MPE=-3.94 mg/d, RMSE = 4.29, 2% with ideal dose | None | NA | Y | PY | PY | NI | Y | Unclear | Y | - |
| 168 | Schelleman 2008^55^ | Ini-pgx | Cini 2012^102^ | Italy | DVT, HV, PE, other | 2.0–3.5 | 40 | 100 | - | - | - | 58 | 70 ± 16 | 43 | 1.14 | - | Missing data | Excluded | N | PY | N | N | Y | High | Y | - |
| 169 | Wen 2008^56^ | Ini-pgx | Shin 2011^236^ | 9 (IWPC) | DVT, PE, AF, HV, stroke, other | 2.0–3.0 | 1940 | 64 | 20 | 16 | - | 60 | In decades | - | 1.39 | 44% with ideal dose | Missing data | Excluded | Y | PY | N | N | Y | High | Y | - |
| 170 |  |  | Tan 2012^115^ | China | HV | 1.7–3.0 | 320 | - | 100 | - | - | 39 | 47 ± 11 |  |  | MPE = -0.15; 63% with ideal dose. | None | NI | Y | PY | PY | NI | N | High | Y | - |
| 171 |  |  | Cho 2011^25^ | Korea | AF | 2.0–3.0 | 108 | - | 100 | - | - | 64 | 67 ± 10 | - | - | r=0.665, M%PE=-34% | None | NI | Y | PY | PY | NI | N | High | Y | - |
| 172 |  |  | Peng 2015^240^ | China | HV | 2.0–3.0 | 586 | - | 100 | - | - | 45 | 52 ± 11 | 37 | 0.72 | 49% with ideal dose | None | NI | Y | PY | PY | NI | Y | Unclear | Y | - |
| 173 |  |  | Lin 2016^27^ | China | HV | 1.5–2.5 | 208 | - | 100 | - | - | - | - | 32 | 0.62 | MAPE=22%, 78% within 1mg/d, 53% with ideal dose | None | NI | Y | PY | PY | NI | Y | Unclear | Y | - |
| 174 |  |  | Wen 2017^259^ | Taiwan | AF, DVT, PE, stroke, other | 2.0–3.0 | 107 | - | 100 | - | - | 55 | 67 (range 30–92) | - | 0.92 | 40% within 0.5 mg/d | None | NI | Y | PY | PY | NI | Y | Unclear | Y | - |
| 175 |  |  | Xie 2020^141^ | China | HV, AF, other | 1.5–3.0. | 325 | - | 100 | - | - | 51 | 57 ± 13 | 32 | - | Ideal dose = 32%, MPE = 0.69 mg/d | NA | NI | Y | PY | PY | NI | N | High | Y | - |
| 176 | Wen 2008^56^ | Ini-pgx | Xu 2015^245^ | China | HV | 1.8–2.5 | 59 | - | 100 | - | - | 47 (of 193) | 53 ± 12 (for 193) | - | 0.94 | 49% with ideal dose | Loss to follow-up | NI | N | PY | PY | NI | Y | High | Y | - |
| 177 | Wu 2008^57^ | Ini-pgx | Wu 2008^57^ | USA | AF, DVT, PE, HV, stroke, other | 2.0–3.0 | 92 | 48 | 16 | 34 | 2 | 70 | 69 ± 13 | - | - | r = 0.67 | Missing data | Excluded | N | PY | N | N | N | High | Y | - |
| 178 |  |  | Langley 2009^234^ | USA | - | - | 75 | 81 | - | 19 | - | 63 | - | 11 | - | R2 = 38% with 2 outliers excluded | Missing data | Excluded | N | PY | N | N | N | High | Y | - |
| 179 |  |  | Lubitz 2010^35^ | USA | AF, DVT, PE, Other | 2.0–3.0 | 145 | 71 | 10 | 19 | - | 62 | 67 ± 14 | 55 | - | - | Non-compliance | NI | Y | PY | PY | NI | N | High | Y | Different outcome determination |
| 180 |  |  | Sagreiya 2010^78^ | USA | - | 2.0–3.0 | 95 | 75 | 17 | 8 | - | 58 (of 104) | 67 (53–75) (for 104) | 35 | 1.43 | - | Missing data | Excluded | N | PY | N | N | Y | High | Y | - |
| 181 |  |  | Cho 2011^25^ | Korea | AF | 2.0–3.0 | 108 | - | 100 | - | - | 64 | 67 ± 10 | - | - | r=0.571, M%PE=-41% | None | NI | Y | PY | PY | NI | N | High | Y | - |
| 182 |  |  | Lei 2012^243^ | China | AF, DVT, PE, HV | 1.6–2.5 | 368 | - | 100 | - | - | 47 | 46 ± 12 | 55 | 1.12 | 68% with ideal dose | None | NI | Y | PY | PY | NI | Y | Unclear | Y | - |
| 183 |  |  | Cho 2016^162^ | Korea | Stroke | 1.5–3.0 | 101 | - | 100 | - | - | 63 | 64 ± 13 | 13 | - | M%PE=46% | None | NI | Y | PY | PY | NI | N | High | Y | - |
| 184 | Huang 2009^58^ | Ini-pgx | Huang 2009^58^ | China | HV | 1.8–3.0 | 87 | - | 100 | - | - | 31 (of 121) | 42 ± 10 (for 121) | 45 | - | MPE=0.14 mg/d, 66% with predicted dose within -1 to 0.5 mg/d | None | NI | N | PY | PY | NI | N | High | Y | - |
| 185 |  |  | Cho 2011^25^ | Korea | AF | 2.0–3.0 | 108 | - | 100 | - | - | 64 | 67 ± 10 | - | - | r=0.674, M%PE=-31% | None | NI | Y | PY | PY | NI | N | High | Y | - |
| 186 |  |  | Shin 2011^236^ | 9 (IWPC) | DVT, PE, AF, HV, stroke, other | 2.0–3.0 | 1940 | 64 | 20 | 16 | - | 60 | In decades | - | 1.46 | 40% with ideal dose | Missing data | Excluded | Y | PY | N | N | Y | High | Y | - |

**Table S7. Continued.**

| **#** | **Algorithm** | **Sub-type** | **Validation study** | **Country** | **Indication** | **Target INR** | **N** | **Ethnicity (%)** | | | | **Male %** | **Age, yrs (mean ± SD or median, IQR)** | **R2** | **MAE (mg/d)** | **Other** | **Excluded participants** | **Missing data** | **PROBAST** | | | | | **ROB** | **Stable dose defined** | **Other key ROB concerns**  **(domains 1-3)** |
| --- | --- | --- | --- | --- | --- | --- | --- | --- | --- | --- | --- | --- | --- | --- | --- | --- | --- | --- | --- | --- | --- | --- | --- | --- | --- | --- |
|  |  |  |  |  |  |  |  | **W** | **A** | **B** | **M** |  |  |  |  |  |  |  | **4.1** | **4.2** | **4.3** | **4.4** | **4.7** |  |  |  |
| 187 | Huang 2009^58^ | Ini-pgx | Lei 2012^243^ | China | AF, DVT, PE, HV | 1.6–2.5 | 368 | - | 100 | - | - | 47 | 46 ± 12 | 50 | 1.17 | 62% with ideal dose | None | NI | Y | PY | PY | NI | Y | Unclear | Y | - |
| 188 |  |  | Liu 2012^237^ | China | AF, HV, DVT, PE | 1.6–2.5 | 282 | - | 100 | - | - | 50 | 56 ± 16 | - | 0.73 | 49% with ideal dose | None | NI | Y | PY | PY | NI | Y | Unclear | Y | - |
| 189 |  |  | Tan 2012^115^ | China | HV | 1.7–3.0 | 320 | - | 100 | - | - | 39 | 47 ± 11 | - | - | MPE=-0.30 mg/d, 60% with ideal dose. | None | NI | Y | PY | PY | NI | N | High | Y | - |
| 190 |  |  | Wang 2012^260^ | China | RHD after HV | 1.8–3.0 | 72 | - | 100 | - | - | 31 (of 101) | 42 ± 7 (for 101) | - | - | r=0.684 | None | NI | N | PY | PY | NI | N | High | Y | - |
| 191 |  |  | Zhao 2014^68^ | China | HV | 1.5–2.5 | 122 | - | 100 | - | - | 31 | 50 ± 10 |  |  | MPE=0.07 mg/d, 61% with ideal dose | None | NI | Y | PY | PY | NI | N | High | Y | - |
| 192 |  |  | Peng 2015^240^ | China | HV | 2.0–3.0 | 586 | - | 100 | - | - | 45 | 52 ± 11 | 37 | 0.72 | 50% with ideal dose | None | NI | Y | PY | PY | NI | Y | Unclear | Y | - |
| 193 |  |  | Xu 2015^245^ | China | HV | 1.8–2.5 | 59 | - | 100 | - | - | 47 (of 193) | 53 ± 12 (for 193) | - | 0.87 | 49% with ideal dose | Loss to follow-up | NI | N | PY | PY | NI | Y | High | Y | - |
| 194 |  |  | Cho 2016^162^ | Korea | Stroke | 1.5–3.0 | 101 | - | 100 | - | - | 63 | 64 ± 13 | 24 | - | M%PE=-17% | None | NI | Y | PY | PY | NI | N | High | Y | - |
| 195 |  |  | Lin 2016^27^ | China | HV | 1.5–2.5 | 208 | - | 100 | - | - | - | - | 33 | 0.63 | MAPE=22%, 83% within 1mg/d, 53% with ideal dose | None | NI | Y | PY | PY | NI | Y | Unclear | Y | - |
| 196 |  |  | Yang 2016^242^ | Korea | AF, CI | - | 310 | - | 100 | - | - | - | mean 69 (26–94) | - | - | r=0.726, MPE=-1.33 mg/d, 26% with ideal dose | Missing data | Excluded | Y | PY | N | N | N | High | N | - |
| 197 |  |  | Zhu 2017^180^ | China | HV | 1.8–2.5 | 42 | - | Y | - | - | - | - | - | - | MPE=0.48 mg/d, RMSE = 0.69 mg/d | None | NI | N | PY | PY | NI | Y | High | N | - |
| 198 |  |  | Chumnumwat 2018^91^ | Thailand | HV, AF, VTE | 2.0–3.0 | 165 | - | 100 | - | - | 38 | 50 ± 11 | - | - | MPE=-0.48 mg/d, 45% with ideal dose | None | NI | Y | PY | PY | NI | N | High | Y | - |
| 199 |  |  | Dong 2019^246^ | China | AF, PE, DVT, other | 1.6–2.8 | 119 | - | 100 | - | - | 51 | 73 (range 34–90) |  |  | r=0.54, MPE= -0.20 mg/d, RMSE=0.93, 56% with ideal dose | Missing genotype data | Excluded | Y | PY | PN | PN | Y | High | Y | - |
| 200 |  |  | Sasano 2019^61^ | Japan | AF, DVT, dilated CMP, PE, other | 1.5–3.0 | 125 | - | 100 | - | - | 60 | 73 ± 12 | - | 0.67 | 50% with ideal dose, MPE=-0.26, RMSE= 0.93, RMSPE=39% | None | NI | Y | PY | PY | NI | Y | Unclear | Y | - |
| 201 |  |  | Cho 2020^198^ | Korea | AF, CI, DVT, PE, HV | - | 109 | - | 100 | - | - | 62 | 70 (range 29–91) | - | - | r=0.53, MPE=-2.17 mg/d, RMSE = 2.69, 22% with ideal dose | None | NA | Y | PY | PY | NI | Y | Unclear | Y | - |
| 202 |  |  | Xie 2020^141^ | China | HV, AF, other | 1.5–3.0. | 325 | - | 100 | - | - | 51 | 57 ± 13 | 35 | - | Ideal dose = 35%, MPE = 0.62 mg/d | NA | NI | Y | PY | PY | NI | N | High | Y | - |
| 203 | IWPC 2009^3^ | Ini-pgx | Lubitz 2010^35^ | USA | AF, DVT, PE, Other | 2.0–3.0 | 145 | 71 | 10 | 19 | - | 62 | 67 ± 14 | 50 | - | - | Non-compliance | NI | Y | PY | PY | NI | N | High | Y | Different outcome determination |
| 204 |  |  | Roper 2010^77^ | USA | AF, PE, DVT, CVA, HV, other | 2.0–3.0 | 121 | 100 | - | - | - | 61 (of 125) | 70 ± 13 (for 125) | 65 | 1.02 | MAPE=22%, 53% with ideal dose | Missing data | Excluded | Y | PY | N | N | Y | High | Y | - |
| 205 |  |  | Sagreiya 2010^78^ | USA | - | 2.0–3.0 | 104 | 75 | 17 | 8 | - | 58 | 67 (53–75) | 46 | 1.16 | 63% within 1 mg/d | None | NI | Y | PY | PY | NI | Y | Unclear | Y | - |
| 206 |  |  | Shaw 2010^231^ | USA | AF, DVT, PE, other | 2.0–3.0 | 71 | 74 | - | 4 | 21 | 45 | 60 ± 15 | 65 | 1.8 | 41% within 1 mg/d | Missing data | Excluded | N | PY | N | N | Y | High | Y | - |
| 207 |  |  | Takeuchi 2010^23^ | Japan | AF, HV, DVT, PE, other | 1.6–2.6 | 200 | - | 100 | - | - | 68 | 67 ± 10 | 28 | - | 71% within 1mg/d | None | NI | Y | PY | PY | NI | N | High | N |  |
| 208 |  |  | Botton 2011^82^ | Brazil | AF, TE, HV, CVA, thrombophilia, other | 2.0–3.5 | 279 | 100 | - | - | - | 56 | 63 ± 14 | 46 | - | - | None | NI | Y | PY | PY | NI | N | High | Y | - |
| 209 |  |  | Cho 2011^25^ | Korea | AF | 2.0–3.0 | 108 | - | 100 | - | - | 64 | 67 ± 10 | - | - | r=0.698, M%PE=-22%, 40% with ideal dose | None | NI | Y | PY | PY | NI | N | High | Y | - |
| 210 |  |  | Cavallari 2011^83^ | USA | VTE, AF, stroke, HV, other | 2.0–3.5 | 50 | 100 | - | - | - | 36 | 57 ± 15 | 43 | 1.2 median | 50% within 1 mg/d | None | NI | N | PY | PY | NI | Y | High | Y | - |

**Table S7. Continued.**

| **#** | **Algorithm** | **Sub-type** | **Validation study** | **Country** | **Indication** | **Target INR** | **N** | **Ethnicity (%)** | | | | **Male %** | **Age, yrs (mean ± SD or median, IQR)** | **R2** | **MAE (mg/d)** | **Other** | **Excluded participants** | **Missing data** | **PROBAST** | | | | | **ROB** | **Stable dose defined** | **Other key ROB concerns**  **(domains 1-3)** |
| --- | --- | --- | --- | --- | --- | --- | --- | --- | --- | --- | --- | --- | --- | --- | --- | --- | --- | --- | --- | --- | --- | --- | --- | --- | --- | --- |
|  |  |  |  |  |  |  |  | **W** | **A** | **B** | **M** |  |  |  |  |  |  |  | **4.1** | **4.2** | **4.3** | **4.4** | **4.7** |  |  |  |
| 211 | IWPC 2009^3^ | Ini-pgx | Perera 2011^89^ | USA | AF, DVT, PE, HV | - | 330 | - | - | 100 | - | 28 | mean 57 | 26 | - | - | Missing data | Excluded | Y | PY | N | N | N | High | Y | - |
| 212 |  |  | Zambon 2011^21^ | Italy | AF, DVT, other | 2.0–3.0 | 274 | 100 | - | - | - | 65 | 74 (range 39–92) | 63 | 1.09 | - | Missing data | Excluded | Y | PY | N | N | Y | High | Y | - |
| 213 |  |  | Zambon 2011^21^ | Italy | AF, DVT, other | 2.0–3.0 | 97 | 100 | - | - | - | 55 | 74 (range 43–87) | 57 | 1.10 | - | Missing data | Excluded | N | PY | N | N | Y | High | Y | - |
| 214 |  |  | Anderson 2012^99^ | US | DVT, PE, AF, other | 1.5–3.5 | 488 | 95 | - | - | - | 47 (for 504) | mean 60.6 (range 18–90) (for 504) | - | 1.00 | - | None | NI | Y | PY | PY | NI | Y | Unclear | Y | Some stable doses estimated |
| 215 |  |  | Bazan 2012^249^ | Egypt | AF, arterial or venous thrombosis, HV | 2.0–3.5 | 63 | - | - | - | 100 | 49 | 46 ± 13 | 42 | 3.2 | 25% within 1 mg/d | None | NI | N | PY | PY | NI | Y | High | Y | - |
| 216 |  |  | Biss 2012^100^  (***Children***) | UK | FP, HV, aneurysm, dilated CMP, DVT, PE, pulmonary HT, stroke, other | 2.0–3.5 | 120 | 76 | 13 | 5 | 6 | 68 | 11 (range 1–18) | - | - | r = 0.76, MPE (or MAE) = 1.5 mg/d | None | NI | Y | PY | PY | NI | Y | Unclear | Y | - |
| 217 |  |  | Cini 2012^102^ | Italy | DVT, HV, PE, other | 2.0–3.5 | 40 | 100 | - | - | - | 58 | 70 ± 16 | 47 | 1.21 | - | Missing data | Excluded | N | PY | N | N | Y | High | Y | - |
| 218 |  |  | Kurnik 2012^105^ | Israel | - | 2.0–3.5 | 210 | 100 | - | - | - | 48 | 61 ± 16 | 27 | 2.06 | - | None | NI | Y | PY | PY | NI | Y | Unclear | Y | - |
| 219 |  |  | Lei 2012^243^ | China | AF, DVT, PE, HV | 1.6–2.5 | 368 | - | 100 | - | - | 47 | 46 ± 12 | 52 | 1.19 | 63% with ideal dose | None | NI | Y | PY | PY | NI | Y | Unclear | Y | - |
| 220 |  |  | Liang 2012^107^ | China | AF, HV, DVT/PE | 2.0–3.0 | 115 | - | 100 | - | - | 62 | 65 ± 13 | - | 0.70 | - | None | NI | Y | PY | PY | NI | Y | Unclear | Y | - |
| 221 |  |  | Liu 2012^237^ | China | AF, HV, DVT, PE | 1.6–2.5 | 282 | - | 100 | - | - | 50 | 56 ± 16 | - | 0.80 | 45% with ideal dose | None | NI | Y | PY | PY | NI | Y | Unclear | Y | - |
| 222 |  |  | Marin-Leblanc 2012^250^ | Canada | AF, HV | 2.0–3.5 | 605 | Y | - | - | - | 68 | 67 ± 11 | 43 | 1.52 | 46% with ideal dose | None | NI | Y | PY | PY | NI | Y | Unclear | Y | - |
| 223 |  |  | Pathare 2012^111^ | Oman | AF, ATE, HV, PE, other | 2.0–3.0 | 212 | - | - | - | 100 | 53 | 50 ± 18 | 34 | - | - | Outliers | NI | Y | PY | N | NI | N | High | Y | - |
| 224 |  |  | Ramirez 2012^114^ | USA | VTE, AF, stroke, orthopedic, other | 2.0–3.0 | 1167 | 88 | 1 | 12 | - | 55 | 66 (95% CI 35–87) | 50; 29 | 1.81 | - | Missing data | Excluded | Y | PY | N | N | Y | High | Y | - |
| 225 |  |  | Ramos 2012^212^ | Puerto Rico | AF, PTE, other | 2.0–3.5 | 163 | 90 | - | 10 | - | 99 | 68 ± 9 | 36 | 1.13 | MAPE=25% | Missing data | Excluded | Y | PY | N | N | Y | High | Y | - |
| 226 |  |  | Ramos 2012^212^,  Duconge 2016^213^ | USA | AF, PTE, other | 2.0–3.5 | 55 | 93 | - | 7 | - | 36 | 61 ± 14 | 21,  39 | 1.57,  1.60 | MAPE=34% | Missing data | Excluded | N | PY | N | N | Y | High | Y | - |
| 227 |  |  | Tan 2012^115^ | China | HV | 1.7–3.0 | 320 | - | 100 | - | - | 39 | 47 ± 11 | - | - | MPE = 0.28 mg/wk, 54% with ideal dose. | None | NI | Y | PY | PY | NI | N | High | Y | - |
| 228 |  |  | Valentin 2012^261^ | Puerto Rico | AF, DVT, PE, CVA, stroke, HV, other | 2.0–3.5 | 97 | - | - | - | 100 | 99 | 67 ± 8 | 13-45 | 1.54 | 34% with ideal dose | Missing data | Excluded | N | PY | N | N | Y | High | Y | - |
| 229 |  |  | Xu 2012^22^ | China | AF, DVT, PE, HV, CMP, other | 1.6–3.0 | 103 | - | 100 | - | - | 46 | 56 ± 15 | - | 0.81 | - | None | NI | Y | PY | PY | NI | Y | Unclear | Y | - |
| 230 |  |  | Ekladious 2013^123^ | Egypt | AF, DVT, HV, coronary heart disease | 2.0–3.0 | 35 | - | - | - | 100 | - | - | - | - | r=0.304 | Missing data | Excluded | N | PY | N | N | N | High | Y | - |
| 231 |  |  | Perera 2013^130^ | USA | AF, DVT, PE, HV, stroke, other | 2.0–3.0 | 432 | - | - | 100 | - | 39 | 59 ± 16 | 22 | - | - | Missing data | Excluded | Y | PY | N | N | N | High | Y | - |
| 232 |  |  | Sharabiani 2013^204^ | USA | DVT, PE, other | 2.0–3.0 | ~59 | - | - | 100 | - | - | - | - | 2.04 | RMSE=2.36 mg/d | Outliers | Single imputation | N | PY | N | PN | Y | High | Y | - |
| 233 |  |  | Bosch 2014^134^ | Puerto Rico | AF, DVT, PE | 2.0–3.5 | 131 | - | - | - | 100 | 100 | 68 ± 9.2 (for 138) | 1-2 | 1.60 | 27% patients with ideal dose | Missing data | Excluded | Y | PY | N | N | Y | High | Y | - |
| 234 |  |  | Daneshjou 2014^136^ | USA | VTE | - | 476 | - | - | 100 | - | 37 | mean 57 | 30 | - | - | None | NI | Y | PY | PY | NI | N | High | N | - |
| 235 |  |  | Hernandez 2014^90^ | USA | AF, DVT, PE, other | - | 149 | - | - | 100 | - | 53 | 59 ± 16 | 15 | 1.74 | 49% with ideal dose | Missing data | Excluded | Y | PY | N | N | Y | High | Y | - |
| 236 |  |  | Krishna Kumar 2014^139^ | India | RHD (HV, AF, mitral stenosis), IHD, DVT, dilated CMP, stroke, other | 2.0–3.5 | 240 | - | 100 | - | - | 37 | 43 ± 11 | - | - | 17% within 1 mg/d | Missing data | Excluded | Y | PY | N | N | N | High | Y | - |
| 237 |  |  | Pavani 2014^142^ | India | HV, DVT, AF | 2.0–3.5 | 125 | - | 100 | - | - | 53 | 38 ± 15 | - | - | r=0.29 | None | NI | Y | PY | PY | NI | N | High | N | - |
| 238 |  |  | Shaw 2014^144^  (***Children***) | Canada | FP, HV, DVT, PE, other | Various | 77 | 66 | 17 | - | 17 | 56 (of 93) | 5 (range <1–18) (for 93) | 57 | - | MPE=1.80 mg/d | None | NI | N | PY | PY | NI | N | High | Y | - |

**Table S7. Continued.**

| **#** | **Algorithm** | **Sub-type** | **Validation study** | **Country** | **Indication** | **Target INR** | **N** | **Ethnicity (%)** | | | | **Male %** | **Age, yrs (mean ± SD or median, IQR)** | **R2** | **MAE (mg/d)** | **Other** | **Excluded participants** | **Missing data** | **PROBAST** | | | | | **ROB** | **Stable dose defined** | **Other key ROB concerns**  **(domains 1-3)** |
| --- | --- | --- | --- | --- | --- | --- | --- | --- | --- | --- | --- | --- | --- | --- | --- | --- | --- | --- | --- | --- | --- | --- | --- | --- | --- | --- |
|  |  |  |  |  |  |  |  | **W** | **A** | **B** | **M** |  |  |  |  |  |  |  | **4.1** | **4.2** | **4.3** | **4.4** | **4.7** |  |  |  |
| 239 | IWPC 2009^3^ | Ini-pgx | Zhao 2014^68^ | China | HV | 1.5–2.5 | 122 | - | 100 | - | - | 31 | 50 ± 10 | - | - | MPE=0.53 mg/d, 49% with ideal dose | None | NI | Y | PY | PY | NI | N | High | Y | - |
| 240 |  |  | Drozda 2015^150^ | USA | AF, DVT, PE, stroke, HV, other | - | 274 | - | - | 100 | - | 28 | 55 ± 16 | - | - | Median PE of -0.5 and 0.7 in CYP2C9*5, *6, *8, *11 non-carriers & carriers respectively | None | NI | Y | PY | PY | NI | N | High | Y | - |
| 241 |  |  | Karaca 2015^154^ | Turkey | AF, HV, CVA, DVT, PE, other | - | 97 | - | - | - | 100 | 49 | 61 ± 13 | - | 1.70 | r=0.586, 33% with ideal dose | None | NI | N | PY | PY | NI | Y | High | N | - |
| 242 |  |  | Karaca 2015^154^ | Turkey | AF, HV, CVA, DVT, PE, other | - | 92 | - | - | - | 100 | 51 | 67 ± 12 | - | 1.26 | r = 0.552, 34% with ideal dose | None | NI | N | PY | PY | NI | Y | High | N | - |
| 243 |  |  | Peng 2015^240^ | China | HV | 2.0–3.0 | 586 | - | 100 | - | - | 45 | 52 ± 11 | 35 | 0.88 | 41% with ideal dose | None | NI | Y | PY | PY | NI | Y | Unclear | Y | - |
| 244 |  |  | Saffian 2015^254^ | New  Zealand | AF, DVT, PE | 2.0–3.0 | 46 | Y | - | - | - | 41 | 62 (range 29–87) | - | - | MPE= -0.72 mg/d, RMSE=1.62 mg/d | Missing data | Single imputation | N | PY | N | N | Y | High | Y | - |
| 245 |  |  | Santos 2015^160^,  Tavares 2018^189^ | Brazil | AF, CVA, TE, HV, other | 1.8–3.2 | 133 | 56 | - | 17 | 26 | 56 | 63 ± 17 | 51 | - | r = 0.5330 | None | NI | Y | PY | PY | NI | N | High | Y | - |
| 246 |  |  | Xu 2015^245^ | China | HV | 1.8–2.5 | 59 | - | 100 | - | - | 47 (of 193) | 53 ± 12 (for 193) | - | 0.77 | 56% with ideal dose | Loss to follow-up | NI | N | PY | PY | NI | Y | High | Y | - |
| 247 |  |  | Cho 2016^162^ | Korea | Stroke | 1.5–3.0 | 101 | - | 100 | - | - | 63 | 64 ± 13 | 37 | - | M%PE=-18% | None | NI | Y | PY | PY | NI | N | High | Y | - |
| 248 |  |  | Duconge 2016^213^ | Puerto Rico | AF, DVT, PE, other | 2.0–3.5 | 255 | 89 | - | 11 | - | 99 | 68 ± 10 | 2-23 | 0.97- 2.48 | 25% to 62% with ideal dose, MAPE=21-53% | Missing data | Excluded | Y | PY | N | N | Y | High | Y | - |
| 249 |  |  | Eriksson 2016^163^ | 44 (RE­LY  sub-study) | AF | 2.0–3.0 | 951 | 87 | 2 | 1 | 10 | 66 (of 956) | 72 (68–77) (for 956) | 53 | - | 57% with ideal dose | None | NI | Y | PY | PY | NI | N | High | Y | - |
| 250 |  |  | French 2016^252^ | USA | AF, DVT, PE, other | 2.0–3.0 | 370 | 77 | - | 23 | - | 53 (of 514) | 59 (48–70) (for 514) | 47 | 1.30 | r=0.70, 51% within 1 mg/d  (White 54%, Black 40%) | None | NI | Y | PY | PY | PY | Y | Low | Y | - |
| 251 |  |  | Lin 2016^27^ | China | HV | 1.5–2.5 | 208 | - | 100 | - | - | - | - | 27 | 0.74 | MAPE = 29%, 74% within 1mg/d, 49% with ideal dose | None | NI | Y | PY | PY | NI | Y | Unclear | Y | - |
| 252 |  |  | Stack 2016^262^ | USA | AF, DVT, PE, other | 2.0–3.0 | 146 | 75 | <1 | 6 | 19 | 100 | 73 ± 11 | - | 0.99 | 80% with ideal dose, 55% within 0.5 mg/d | Missing data | Excluded | Y | PY | N | N | Y | High | Y | - |
| 253 |  |  | Wakamiya 2016^169^  (***Children***) | Japan | HV, FD, Kawasaki disease, others | 1.5–2.5 | 45 | - | 100 | - | - | 84 | 8 (range 0–19) | 57 | - | 20% within 1mg/d | None | NI | N | PY | PY | NI | N | High | Y | - |
| 254 |  |  | Yang 2016^242^ | Korea | AF, CI | - | 310 | - | 100 | - | - | - | mean 69 (26–94) | - | - | r=0.717, MPE = -1.10, 33% with ideal dose | Missing data | Excluded | Y | PY | N | N | N | High | N | - |
| 255 |  |  | Huang 2017^263^ | China | AF | mean 2.1 | 362 | - | 100 | - | - | 50 | 60 ± 12 | - | - | 57% with ideal dose | Missing data | Excluded | Y | PY | N | N | N | High | N | - |
| 256 |  |  | Wen 2017^259^ | Taiwan | AF, DVT, PE, stroke, other | 2.0–3.0 | 107 | - | 100 | - | - | 54 | 67 (range 26–88) | - | 1.04 | 40% within 0.5 mg/d | None | NI | Y | PY | PY | NI | Y | Unclear | Y | - |
| 257 |  |  | Wiley 2017^179^ | USA | VTE, AF | 1.9–3.2 | 2181 | 88 | - | 12 | - | 58 | 66 ± 15 | 43 | 1.36 | - | Missing data | Excluded | Y | PY | N | N | Y | High | Y | - |
| 258 |  |  | Ayesh 2018^264^ | Gaza-  Palestine | VTE, HV, other | 2.0–3.5 | 47 | - | - | - | 100 | 43 (of 101) | 49 ± 14 (for 101) | 35 | 1.27 | 68% with ideal dose | None | NI | N | PY | PY | NI | Y | High | Y | - |
| 259 |  |  | Chumnumwat 2018^91^ | Thailand | HV, AF, VTE | 2.0–3.0 | 165 | - | 100 | - | - | 38 | 50 ± 11 | - | - | MPE=0.04 mg/d; 49% with ideal dose | None | NI | Y | PY | PY | NI | N | High | Y | - |
| 260 |  |  | Galvez 2018^183^ | Colombia | HV, DVT, PE, CVD, other | 2.0–3.0 | 87 | - | - | - | 100 | 53 | 62 ± 12 | 44 |  |  | None | NI | N | PY | PY | NI | N | High | Y | - |
| 261 |  |  | Pei 2018^186^ | China | HV | 2.0–3.0 | 358 | - | 100 | - | - | 51 | - | 46 | 0.85 | MAPE=29%, 54% with ideal dose | Missing data | Excluded | Y | PY | N | N | Y | High | Y | - |
| 262 |  |  | Selim 2018^187^ | Egypt | AF, DVT, PE, HV | 2.0–3.2 | 100 | - | - | - | 100 | 46 | 45 ± 13 | 42 |  | 58% within 1 mg/d | None | NI | Y | PY | PY | NI | N | High | Y | - |

**Table S7. Continued.**

| **#** | **Algorithm** | **Sub-type** | **Validation study** | **Country** | **Indication** | **Target INR** | **N** | **Ethnicity (%)** | | | | **Male %** | **Age, yrs (mean ± SD or median, IQR)** | **R2** | **MAE (mg/d)** | **Other** | **Excluded participants** | **Missing data** | **PROBAST** | | | | | **ROB** | **Stable dose defined** | **Other key ROB concerns**  **(domains 1-3)** |
| --- | --- | --- | --- | --- | --- | --- | --- | --- | --- | --- | --- | --- | --- | --- | --- | --- | --- | --- | --- | --- | --- | --- | --- | --- | --- | --- |
|  |  |  |  |  |  |  |  | **W** | **A** | **B** | **M** |  |  |  |  |  |  |  | **4.1** | **4.2** | **4.3** | **4.4** | **4.7** |  |  |  |
| 263 | IWPC 2009^3^ | Ini-pgx | Danese 2019^191^ | 17 | AF, CMP, orthopedic, other | 1.5–4.0 | 775 | 100 | - | - | - | - | - | 43 | - | - | None | NI | Y | PY | PY | NI | N | High | Y | - |
| 264 |  |  | Danese 2019^191^ | 17 | AF, CMP, orthopedic, other | 1.5–4.0 | 80 | - | - | 100 | - | - | - | 23 | - | - | None | NI | N | PY | PY | NI | N | High | Y | - |
| 265 |  |  | Danese 2019^191^ | 17 | AF, CMP, orthopedic, other | 1.5–4.0 | 34 | - | 100 | - | - | - | - | 11 | - | - | None | NI | N | PY | PY | NI | N | High | Y | - |
| 266 |  |  | Dong 2019^246^ | China | AF, PE, DVT, other | 1.6–2.8 | 119 | - | 100 | - | - | 51 | 73 (range 34–90) | - | - | r=0.59, MPE= -0.11 mg/d, RMSE=0.89, 60% with ideal dose | Missing genotype data | Excluded | Y | PY | PN | PN | Y | High | Y | - |
| 267 |  |  | Helin 2019^257^ | Finland | Thrombosis, HV | 2.0–3.5 | 50 | Y | - | - | - | 32 | mean 47 (range 20-76) | 29 | 1.4 median | - | None | NI | Y | PY | PY | NI | N | High | N | - |
| 268 |  |  | Filippelli 2019^265^ | Italy | Non-valvular AF, HV | 1.5–3.5 | 283 | 100 | - | - | - | 49 | 74 ± 6 | 31 | 1.09 | Ideal dose = 38%, MPE = 0.26 | None | NI | N | PY | PY | NI | Y | High | Y | - |
| 269 |  |  | Sasano 2019^61^ | Japan | AF, DVT, dilated CMP, PE, other | 1.5–3.0 | 125 | - | 100 | - | - | 60 | 73 ± 12 | - | 0.66 | 50% with ideal dose, MPE=-0.21, RMSE= 0.89, RMSPE=37% | None | NI | Y | PY | PY | NI | Y | Unclear | Y | - |
| 270 |  |  | Tao 2019^194^ | China | AF, HV, DVT, PE, other | 2.0–3.0 | 517 | - | 100 | - | - | 57 | - | 9 | 0.84 | 41% patients with ideal dose | None | NI | Y | PY | PY | NI | Y | Unclear | Y | - |
| 271 |  |  | Tao 2019^195^ | China | - | 2.0–3.0 | 289 | - | 100 | - | - | 58 (of 247) | Categorized | 12 | - | - | None | NI | Y | PY | PY | NI | N | High | Y | - |
| 272 |  |  | Yang 2019^196^  (***Children***) | China | Kawasaki disease, thrombogenesis | 1.0–2.5 | 44 | - | 100 | - | - | 77 | 4 ± 4 | - | - | r = 0.742 | Missing data | Excluded | N | PY | N | N | N | High | N | - |
| 273 |  |  | Cho 2020^198^ | Korea | AF, CI, DVT, PE, HV | - | 109 | - | 100 | - | - | 62 | 70 (range 29–91) | - | - | r=0.519, MPE=-1.73 mg/d, RMSE = 2.36, 27% with ideal dose | None | NI | Y | PY | PY | NI | Y | Unclear | Y | - |
| 274 |  |  | Xie 2020^141^ | China | HV, AF, other | 1.5–3.0. | 325 | - | 100 | - | - | 51 | 57 ± 13 | 23 | - | Ideal dose = 23%, MPE = 0.87 mg/d | NA | NI | Y | PY | PY | NI | N | High | Y | - |
| 275 | Kim 2009^59^ | Ini-pgx | Cho 2011^25^ | Korea | AF | 2.0–3.0 | 108 | - | 100 | - | - | 64 | 67 ± 10 | - | - | r=0.595, M%PE=-2%, 56% with ideal dose | None | NI | Y | PY | PY | NI | N | High | Y | - |
| 276 |  |  | Tan 2012^115^ | China | HV | 1.7–3.0 | 320 | - | 100 | - | - | 39 | 47 ± 11 | - | - | MPE=1.09 mg/d; 26% with ideal dose | None | NI | Y | PY | PY | NI | N | High | Y | - |
| 277 |  |  | Xu 2015^245^ | China | HV | 1.8–2.5 | 59 | - | 100 | - | - | 47 (of 193) | 53 ± 12 (for 193) | - | 1.04 | 46% with ideal dose | Loss to follow-up | NI | N | PY | PY | NI | Y | High | Y | - |
| 278 |  |  | Yang 2016^242^ | Korea | AF, CI | - | 310 | - | 100 | - | - | - | mean 69 (26–94) | - | - | r=0.654, MPE=-0.29, 47% with ideal dose | Missing data | Excluded | Y | PY | N | N | N | High | N | - |
| 279 |  |  | Cho 2020^198^ | Korea | AF, CI, DVT, PE, HV | - | 109 | - | 100 | - | - | 62 | 70 (range 29–91) | - | - | r=0.422, MPE=-0.72 mg/d, RMSE=1.86, 45% with ideal dose | None | NI | Y | PY | PY | NI | Y | Unclear | Y | - |
| 280 | Ohno 2009^60^ | Ini-pgx | Shin 2011^236^ | 9 (IWPC) | DVT, PE, AF, HV, stroke, other | 2.0–3.0 | 1940 | 64 | 20 | 16 | - | 60 | In decades | - | 1.49 | 42% with ideal dose | Missing data | Excluded | Y | PY | N | N | Y | High | Y | - |
| 281 |  |  | Lei 2012^243^ | China | AF, DVT, PE, HV | 1.6–2.5 | 368 | - | 100 | - | - | 47 | 46 ± 12 | 49 | 1.38 | 62% with ideal dose | None | NI | Y | PY | PY | NI | Y | Unclear | Y | - |
| 282 |  |  | Liu 2012^237^ | China | AF, HV, DVT, PE | 1.6–2.5 | 282 | - | 100 | - | - | 50 | 56 ± 16 | - | 0.76 | 50% with ideal dose | None | NI | Y | PY | PY | NI | Y | Unclear | Y | - |
| 283 |  |  | Tan 2012^115^ | China | HV | 1.7–3.0 | 320 | - | 100 | - | - | 39 | 47 ± 11 | - | - | MPE=0.13 mg/d, 55% with ideal dose. | None | NI | Y | PY | PY | NI | N | High | Y | - |
| 284 |  |  | Peng 2015^240^ | China | HV | 2.0–3.0 | 586 | - | 100 | - | - | 45 | 52 ± 11 | 32 | 0.82 | 42% with ideal dose | None | NI | Y | PY | PY | NI | Y | Unclear | Y | - |
| 285 |  |  | Xu 2015^245^ | China | HV | 1.8–2.5 | 59 | - | 100 | - | - | 47 (of 193) | 53 ± 12 (for 193) | - | 0.73 | 53% with ideal dose | Loss to follow-up | NI | N | PY | PY | NI | Y | High | Y | - |
| 286 |  |  | Cho 2016^162^ | Korea | Stroke | 1.5–3.0 | 101 | - | 100 | - | - | 63 | 64 ± 13 | 42 | - | M%PE=-10%, 46% with ideal dose | None | NI | Y | PY | PY | NI | N | High | Y | - |

**Table S7. Continued.**

| **#** | **Algorithm** | **Sub-type** | **Validation study** | **Country** | **Indication** | **Target INR** | **N** | **Ethnicity (%)** | | | | **Male %** | **Age, yrs (mean ± SD or median, IQR)** | **R2** | **MAE (mg/d)** | **Other** | **Excluded participants** | **Missing data** | **PROBAST** | | | | | **ROB** | **Stable dose defined** | **Other key ROB concerns**  **(domains 1-3)** |
| --- | --- | --- | --- | --- | --- | --- | --- | --- | --- | --- | --- | --- | --- | --- | --- | --- | --- | --- | --- | --- | --- | --- | --- | --- | --- | --- |
|  |  |  |  |  |  |  |  | **W** | **A** | **B** | **M** |  |  |  |  |  |  |  | **4.1** | **4.2** | **4.3** | **4.4** | **4.7** |  |  |  |
| 287 | Ohno 2009^60^ | Ini-pgx | Lin 2016^27^ | China | HV | 1.5–2.5 | 208 | - | 100 | - | - | - | - | 25 | 0.67 | MAPE=26%, 75% within 1mg/d, 58% with ideal dose | None | NI | Y | PY | PY | NI | Y | Unclear | Y | - |
| 288 |  |  | Yang 2016^242^ | Korea | AF, CI | - | 310 | - | 100 | - | - | - | mean 69 (26–94) | - | - | r = 0.711, MPE=-1.04, 37% with ideal dose | Missing data | Excluded | Y | PY | N | N | N | High | N | - |
| 289 |  |  | Zhu 2017^180^ | China | HV | 1.8–2.5 | 42 | - | Y | - | - | - | - | - | - | MPE=0.84 mg/d, RMSE=0.98 mg/d | None | NI | N | PY | PY | NI | Y | High | N | - |
| 290 |  |  | Chumnumwat 2018^91^ | Thailand | HV, AF, VTE | 2.0–3.0 | 165 | - | 100 | - | - | 38 | 50 ± 11 | - | - | MPE=-0.1 mg/d, 48% with ideal dose | None | NI | Y | PY | PY | NI | N | High | Y | - |
| 291 |  |  | Cho 2020^198^ | Korea | AF, CI, DVT, PE, HV | - | 109 | - | 100 | - | - | 62 | 70 (range 29–91) | - | - | r=0.495, MPE=-1.91 mg/d, RMSE=2.50, 28% with ideal dose | None | NA | Y | PY | PY | NI | Y | Unclear | Y | - |
| 292 | Wadelius 2009^64^ | Ini-pgx | Wadelius 2009^64^ | Sweden | AF, DVT, PE, HV, other | 2.0–3.0 | 181 | Y | - | - | - | 67 (of 201) | 67 (range 28–88) (for 201) | 53 | - | - | Missing data | Excluded | Y | PY | N | N | N | High | Y | - |
| 293 |  |  | Lubitz 2010^35^ | USA | AF, DVT, PE, other | 2.0–3.0 | 145 | 71 | 10 | 19 | - | 62 | 67 ± 14 | 46 | - | - | Non-compliance | NI | Y | PY | PY | NI | N | High | Y | Different outcome determination |
| 294 |  |  | Shaw 2010^231^ | USA | AF, DVT, PE, other | 2.0–3.0 | 71 | 74 | - | 4 | 21 | 45 | 60 ± 15 | 55 | 1.9 | 37% within 1 mg/d | Missing data | Excluded | N | PY | N | N | Y | High | Y | - |
| 295 |  |  | Botton 2011^82^ | Brazil | AF, TE, HV, CVA, thrombophilia, other | 2.0–3.5 | 279 | 100 | - | - | - | 56 | 63 ± 14 | 46 | - | - | None | NI | Y | PY | PY | NI | N | High | Y | - |
| 296 |  |  | Cho 2011^25^ | Korea | AF | 2.0–3.0 | 108 | - | 100 | - | - | 64 | 67 ± 10 | - | - | r=0.683, M%PE=-5%, 51% with ideal dose | None | NI | Y | PY | PY | NI | N | High | Y | - |
| 297 |  |  | Shin 2011^236^ | 9 (IWPC) | DVT, PE, AF, HV, stroke, other | 2.0–3.0 | 1940 | 64 | 20 | 16 | - | 60 | In decades | - | 1.34 | 47% with ideal dose | Missing data | Excluded | Y | PY | N | N | Y | High | Y | - |
| 298 |  |  | Zambon 2011^21^ | Italy | AF, DVT, other | 2.0–3.0 | 274 | 100 | - | - | - | 65 | 74 (range 39-92) | 61 | 1.08 | - | Missing data | Excluded | Y | PY | N | N | Y | High | Y | - |
| 299 |  |  | Zambon 2011^21^ | Italy | AF, DVT, other | 2.0–3.0 | 97 | 100 | - | - | - | 55 | 74 (range 43-87) | 59 | 0.98 | - | Missing data | Excluded | N | PY | N | N | Y | High | Y | - |
| 300 |  |  | Cini 2012^102^ | Italy | DVT, HV, PE, other | 2.0–3.5 | 40 | 100 | - | - | - | 58 | 70 ± 16 | 50 | 1.18 | - | Missing data | Excluded | N | PY | N | N | Y | High | Y | - |
| 301 |  |  | Marin-Leblanc 2012^250^ | Canada | AF, HV | 2.0–3.5 | 605 | 100 | - | - | - | 68 | 67 ± 11 | 41 | 1.36 | 39% with ideal dose | None | NI | Y | PY | PY | NI | Y | Unclear | Y | - |
| 302 |  |  | Tan 2012^115^ | China | HV | 1.7–3.0 | 320 | - | 100 | - | - | 39 | 47 ± 11 | - | - | MPE=0.001; 15% with ideal dose. | None | NI | Y | PY | PY | NI | N | High | Y | - |
| 303 |  |  | Francis 2014^239^ | UK | AF, VTE | 2.0–3.0 | 508 | 100 | - | - | - | 55 | 68 | 36 | 1.37 | MAPE = 47% | None | NI | Y | PY | PY | NI | Y | Unclear | Y | - |
| 304 |  |  | Francis 2014^239^ | UK/Sweden | AF, VTE | 2.0–3.0 | 133 | 100 | - | - | - | 59 | 67 | 41 | 1.33 | MAPE = 36% | None | NI | Y | PY | PY | NI | Y | Unclear | Y | - |
| 305 |  |  | Karaca 2015^154^ | Turkey | AF, HV, CVA, DVT, PE, other | - | 97 | - | - | - | 100 | 49 | 61 ± 13 | - | 1.69 | r=0.569, 34% with ideal dose | None | NI | N | PY | PY | NI | Y | High | N | - |
| 306 |  |  | Karaca 2015^154^ | Turkey | AF, HV, CVA, DVT, PE, other | - | 92 | - | - | - | 100 | 51 | 67 ± 12 | - | 1.11 | r = 0.559, 46% with ideal dose | None | NI | N | PY | PY | NI | Y | High | N | - |
| 307 |  |  | Xu 2015^245^ | China | HV | 1.8–2.5 | 59 | - | 100 | - | - | 47 (of 193) | 53 ± 12 (for 193) | - | 1.18 | 29% with ideal dose | Loss to follow-up | NI | N | PY | PY | NI | Y | High | Y | - |
| 308 |  |  | Cho 2016^162^ | Korea | Stroke | 1.5–3.0 | 101 | - | 100 | - | - | 63 | 64 ± 13 | 0.4 | - | M%PE = 51% | None | NI | Y | PY | PY | NI | N | High | Y | - |
| 309 |  |  | Yang 2016^242^ | Korea | AF, CI | - | 310 | - | 100 | - | - | - | mean 69 (26–94) | - | - | r=0.697, MPE=-0.24, 47% with ideal dose | Missing data | Excluded | Y | PY | N | N | N | High | N | - |
| 310 |  |  | Pei 2018^186^ | China | HV | 2.0–3.0 | 358 | - | 100 | - | - | 51 | - | 40 | 1.18 | MAPE=46%, 43% with ideal dose | Missing data | Excluded | Y | PY | N | N | Y | High | Y | - |
| 311 |  |  | Cho 2020^198^ | Korea | AF, CI, DVT, PE, HV | - | 109 | - | 100 | - | - | 62 | 70 (range 29–91) | - | - | r=0.494, MPE=-0.37 mg/d, RMSE = 1.69, 62% with ideal dose | None | NI | Y | PY | PY | NI | Y | Unclear | Y | - |

**Table S7. Continued.**

| **#** | **Algorithm** | **Sub-type** | **Validation study** | **Country** | **Indication** | **Target INR** | **N** | **Ethnicity (%)** | | | | **Male %** | **Age, yrs (mean ± SD or median, IQR)** | **R2** | **MAE (mg/d)** | **Other** | **Excluded participants** | **Missing data** | **PROBAST** | | | | | **ROB** | **Stable dose defined** | **Other key ROB concerns**  **(domains 1-3)** |
| --- | --- | --- | --- | --- | --- | --- | --- | --- | --- | --- | --- | --- | --- | --- | --- | --- | --- | --- | --- | --- | --- | --- | --- | --- | --- | --- |
|  |  |  |  |  |  |  |  | **W** | **A** | **B** | **M** |  |  |  |  |  |  |  | **4.1** | **4.2** | **4.3** | **4.4** | **4.7** |  |  |  |
| 312 | Yoshizawa 2009^65^ | Ini-pgx | Cho 2011^25^ | Korea | AF | 2.0–3.0 | 108 | - | 100 | - | - | 64 | 67 ± 10 |  |  | r=0.406, M%PE=-23% | None | NI | Y | PY | PY | NI | N | High | Y | - |
| 313 | Cen 2010^66^ | Ini-pgx | Xu 2018^67^ | China | HV | 1.8–2.5 | 100 | - | 100 | - | - | 42 | 48 ± 11 | 34 | 0.61 | 63% within 0.6 mg/d, 32% within 0.5 mg/d | None | NI | Y | PY | PY | NI | Y | Unclear | Y | - |
| 314 | Cha 2010^24^ | Ini-pgx | Sasano 2019^61^ | Japan | AF, DVT, dilated CMP, PE, other | 1.5–3.0 | 125 | - | 100 | - | - | 60 | 73 ± 12 | - | 0.72 | MPE=0.22, RMSE = 0.91, RMSPE= 62%, 44% with ideal dose | None | NI | Y | PY | PY | NI | Y | Unclear | Y | - |
| 315 | Du 2010 | Ini-pgx | Zhao 2014^68^ | China | HV | 1.5–2.5 | 122 | - | 100 | - | - | 31 | 50 ± 10 | - | - | MPE=0.27 mg/d, 53% with ideal dose | None | NI | Y | PY | PY | NI | N | High | Y | - |
| 316 | McMillin 2010^71^ | Ini-pgx | McMillin 2010^71^ | USA | Elective total hip or knee arthroplasty | 1.8–2.9 | 114 | 90 | - | - | - | 39 (of 229) | 59 ± 13 (for 229) | - | - | MPE = -0.07 mg/d | None | NA | Y | PY | PY | PY | N | High | Y | - |
| 317 | Nowak-Gottl 2010^73^ | Ini-pgx | Hamberg 2013^109^  (***Children***) | Sweden | FP, HV, dilated CMP, other | 2.0–3.0 for 90% | 49 | Y | Y | - | - | 53 | 7 (range <1–17) | 49 | 0.98 | MPE=-0.26 mg/d, RMSE= 1.05 mg/d, 35% with ideal dose | Missing data | Excluded | N | PY | N | N | Y | High | Y | - |
| 318 |  |  | Hamberg 2014^28^  (***Children***) | Sweden | Cardiac | 1.7–3.0 | 31 | 77 | 13 | 6 | 3 | 58 | 3 (range <1–16) | 59 | - | MPE=0.10 mg/d, RMSE=1.25 mg/d, 35% with ideal dose | None | NI | N | PY | PY | NI | Y | High | Y | - |
| 319 |  |  | Marek 2016^110^  (***Children***) | USA | Thrombosis, congenital heart disease, HV, other | 1.5–3.5 for 78% | 32 | 97 | - | - | 3 | 69 | 5 (range 1–17) | 52 | - | MPE = -0.03mg/d, 16% with ideal dose | None | NI | N | PY | PY | NI | N | High | Y | - |
| 320 | Roper 2010^77^ | Ini-pgx | Roper 2010^77^ | 9 (IWPC sites) | DVT, PE, AF, HV, stroke, other | - | 974 | - | - | - | Y | - | - | 37 | 1.39 | MAPE=44%, 40% with ideal dose | Missing data | Excluded | Y | PY | N | N | Y | High | Y | - |
| 321 | Sangviroon 2010^79^ | Ini-pgx | Chumnumwat 2018^91^ | Thailand | HV, AF, VTE | 2.0–3.0 | 165 | - | 100 | - | - | 38 | 50 ± 11 | - | - | MPE=-0.14 mg/d; 48% with ideal dose | None | NI | Y | PY | PY | NI | N | High | Y | - |
| 322 | Zhang 2010 | Ini-pgx | Zhao 2014^68^ | China | HV | 1.5–2.5 | 122 | - | 100 | - | - | 31 | 50 ± 10 | - | - | MPE=0.25 mg/d, 52% with ideal dose | None | NI | Y | PY | PY | NI | N | High | Y | - |
| 323 | Avery 2011^81^ | Ini-pgx | Yang 2016^242^ | Korea | AF, CI | - | 310 | - | 100 | - | - | - | mean 69 (26–94) | - | - | r=0.717, MPE=-0.97, 38% with ideal dose | Missing data | Excluded | Y | PY | N | N | N | High | N | - |
| 324 |  |  | Cho 2020^198^ | Korea | AF, CI, DVT, PE, HV | - | 109 | - | 100 | - | - | 62 | 70 (range 29–91) | - | - | r=0.519, MPE=-1.57, RMSE = 2.24, 35% with ideal dose | None | NI | Y | PY | PY | NI | Y | Unclear | Y | - |
| 325 | Botton 2011^82^ | Ini-pgx | Santos 2015^160^,  Tavares 2018^189^ | Brazil | AF, CVA, TE, HV, other | 1.8–3.2 | 133 | 56 | - | 17 | 26 | 56 | 63 ± 17 | 32 | - | r=0.3305 | None | NI | Y | PY | PY | NI | N | High | Y | - |
| 326 | Cho 2011^25^ | Ini-pgx | Cho 2011^25^ | Korea | AF | 2.0–3.0 | 108 | - | 100 | - | - | 64 | 67 ± 10 | 46 | - | r = 0.681, M%PE=-4%, 69% with ideal dose | None | NI | Y | PY | PY | NI | N | High | Y | - |
| 327 |  |  | Tan 2012^115^ | China | HV | 1.7–3.0 | 320 | - | 100 | - | - | 39 | 47 ± 11 | - | - | MPE = -1.35; 9% with ideal dose. | None | NI | Y | PY | PY | NI | N | High | Y | - |
| 328 |  |  | Cho 2016^162^ | Korea | Stroke | 1.5–3.0 | 101 | - | 100 | - | - | 63 | 64 ± 13 | 41 | - | M%PE=-50% | None | NI | Y | PY | PY | NI | N | High | Y | - |
| 329 |  |  | Yang 2016^242^ | Korea | AF, CI | - | 310 | - | 100 | - | - | - | mean 69 (26–94) | - | - | r=0.712, MPE= -0.43, 45% with ideal dose | Missing data | Excluded | Y | PY | N | N | N | High | N | - |
| 330 |  |  | Chumnumwat 2018^91^ | Thailand | HV, AF, VTE | 2.0–3.0 | 165 | - | 100 | - | - | 38 | 50 ± 11 | - | - | MPE=-1.73 mg/d, 13% with ideal dose | None | NI | Y | PY | PY | NI | N | High | Y | - |
| 331 |  |  | Cho 2020^198^ | Korea | AF, CI, DVT, PE, HV | - | 109 | - | 100 | - | - | 62 | 70 (range 29–91) | - | - | r=0.543, MPE=-0.73 mg/d, RMSE = 1.84, 49% with ideal dose | None | NI | Y | PY | PY | NI | Y | Unclear | Y | - |
| 332 | Choi 2011^84^ | Ini-pgx | Yang 2016^242^ | Korea | AF, CI | - | 310 | - | 100 | - | - | - | mean 69 (26–94) | - | - | r=0.687, MPE=-0.17, 46% with ideal dose | Missing data | Excluded | Y | PY | N | N | N | High | N | - |

**Table S7. Continued.**

| **#** | **Algorithm** | **Sub-type** | **Validation study** | **Country** | **Indication** | **Target INR** | **N** | **Ethnicity (%)** | | | | **Male %** | **Age, yrs (mean ± SD or median, IQR)** | **R2** | **MAE (mg/d)** | **Other** | **Excluded participants** | **Missing data** | **PROBAST** | | | | | **ROB** | **Stable dose defined** | **Other key ROB concerns**  **(domains 1-3)** |
| --- | --- | --- | --- | --- | --- | --- | --- | --- | --- | --- | --- | --- | --- | --- | --- | --- | --- | --- | --- | --- | --- | --- | --- | --- | --- | --- |
|  |  |  |  |  |  |  |  | **W** | **A** | **B** | **M** |  |  |  |  |  |  |  | **4.1** | **4.2** | **4.3** | **4.4** | **4.7** |  |  |  |
| 333 | Choi 2011^84^ | Ini-pgx | Pei 2018^186^ | China | HV | 2.0–3.0 | 358 | - | 100 | - | - | 51 | - | 38 | 1.24 | MAPE=52%, 34% with ideal dose | Missing data | Excluded | Y | PY | N | N | Y | High | Y | - |
| 334 |  |  | Cho 2020^198^ | Korea | AF, CI, DVT, PE, HV | - | 109 | - | 100 | - | - | 62 | 70 (range 29–91) | - | - | r=0.597, MPE=-1.3 mg/d, RMSE = 2.01, 51% with ideal dose | None | NI | Y | PY | PY | NI | Y | Unclear | Y | - |
| 335 | Perera 2011^89^ | Ini-pgx | Hernandez 2014^90^ | USA | AF, DVT, PE, other | - | 149 | - | - | 100 | - | 53 | 59 ± 16 | 27 | 1.56 | 59% with ideal dose, 11% accurate assigning | Missing data | Excluded | Y | PY | N | N | Y | High | Y | - |
| 336 |  |  | Wiley 2017^179^ | USA | VTE, AF | 1.9–3.2 | 2181 | 88 | - | 12 | - | 58 | 66 ± 15 | 37 | 1.49 | - | Missing data | Excluded | Y | PY | N | N | Y | High | Y | - |
| 337 | Sarapakdi 2011 | Ini-pgx | Chumnumwat 2018^91^ | Thailand | HV, AF, VTE | 2.0–3.0 | 165 | - | 100 | - | - | 38 | 50 ± 11 | - | - | MPE=0.19 mg/d, 48% with ideal dose | None | NI | Y | PY | PY | NI | N | High | Y | - |
| 338 | You 2011 ^97^ | Ini-pgx | Tan 2012^115^ | China | HV | 1.7–3.0 | 320 | - | 100 | - | - | 39 | 47 ± 11 | - | - | MPE = 0.26, 54% with ideal dose | None | NI | Y | PY | PY | NI | N | High | Y | - |
| 339 |  |  | Peng 2015^240^ | China | HV | 2.0–3.0 | 586 | - | 100 | - | - | 45 | 52 ± 11 | 33 | 1.09 | 32% with ideal dose | None | NI | Y | PY | PY | NI | Y | Unclear | Y | - |
| 340 |  |  | Xu 2015^245^ | China | HV | 1.8–2.5 | 59 | - | 100 | - | - | 47 (of 193) | 53 ± 12 (for 193) | - | 0.89 | 41% with ideal dose | Loss to follow-up | NI | N | PY | PY | NI | Y | High | Y | - |
| 341 |  |  | Yang 2016^242^ | Korea | AF, CI | - | 310 | - | 100 | - | - | - | mean 69 (26–94) |  |  | r=0.71, MPE=-1.44, 21% with ideal dose | Missing data | Excluded | Y | PY | N | N | N | High | N | - |
| 342 |  |  | Cho 2020^198^ | Korea | AF, CI, DVT, PE, HV | - | 109 | - | 100 | - | - | 62 | 70 (range 29–91) | - | - | r=0.503, MPE=-2.15 mg/d, RMSE=2.68, 25% with ideal dose | None | NI | Y | PY | PY | NI | Y | Unclear | Y | - |
| 343 |  |  | Xie 2020^141^ | China | HV, AF, other | 1.5–3.0. | 325 | - | 100 | - | - | 51 | 57 ± 13 | 30 | - | Ideal dose = 30%, MPE = 0.76 mg/d | NA | NI | Y | PY | PY | NI | N | High | Y | - |
| 344 | Zambon 2011^21^ | Ini-pgx | Liu 2012^237^ | China | AF, HV, DVT, PE | 1.6–2.5 | 282 | - | 100 | - | - | 50 | 56 ± 16 | - | 1.06 | 36% with ideal dose | None | NI | Y | PY | PY | NI | Y | Unclear | Y | - |
| 345 |  |  | Pengo 2015^266^ | Italy | Non-valvular AF | 2.0–3.0 | 75 | 100 | - | - | - | 66 (of 88) | 71 (range 39–84) (for 88) | - | 1.61 | - | None | NI | N | PY | PY | NI | Y | High | Y | - |
| 346 |  |  | Sasano 2019^61^ | Japan | AF, DVT, dilated CMP, PE, other | 1.5–3.0 | 125 | - | 100 | - | - | 60 | 73 ± 12 | - | 0.75 | MPE=0.31, RMSE = 0.94, RMSPE = 53%, 39% with ideal dose | None | NI | Y | PY | PY | NI | Y | Unclear | Y | - |
| 347 | Anderson 2012^99^ | Ini-pgx | Filippelli 2019^265^ | Italy | Non-valvular AF, HV | 1.5–3.5 | 283 | 100 | - | - | - | 49 | 74 ± 6 | 31 | 1.16 | Ideal dose = 39%, MPE = 1.16 | None | NI | Y | PY | PY | NI | N | High | Y | - |
| 348 | Biss 2012^100^ | Ini-pgx | Biss 2012^267^  (***Children***) | Sweden | FP, HV, CMP, other | 1.8–4.0 | 49 | 82 | 12 | - | 6 | 53 | range <1–18 | - | - | r=0.833 (square-root scale) | None | Single imputation | N | PY | PY | PN | N | High | Y | - |
| 349 |  |  | Hamberg 2013^109^  (***Children***) | Sweden | FP, HV, dilated CMP, other | 2.0–3.0 for 90% | 49 | Y | Y | - | - | 53 | 7 (range <1–17) | 66 | 0.9 | MPE=-0.03 mg/d, RMSE=0.90 mg/d, 41% had ideal dose | Missing data | Excluded | N | PY | N | N | Y | High | Y | - |
| 350 |  |  | Hamberg 2014^28^  (***Children***) | Sweden | Cardiac | 1.7–3.0 | 31 | 77 | 13 | 6 | 3 | 58 | 3 (range <1–16) | 73 | - | MPE=0.04 mg/d, RMSE = 1.04 mg/d, 39% with ideal dose | None | NI | N | PY | PY | NI | Y | High | Y | - |
| 351 |  |  | Shaw 2014^144^  (***Children***) | Canada | FP, HV, DVT, PE, other | Various | 77 | 66 | 17 | - | 17 | 56 (of 93) | 5 (range <1–18) (for 93) | 68 | - | MPE = -0.22 mg/d | None | NI | N | PY | PY | NI | N | High | Y | - |
| 352 |  |  | Marek 2016^110^  (***Children***) | USA | Thrombosis, congenital heart disease, HV, other | 1.5–3.5 for 78% | 32 | 97 | - | - | 3 | 69 | 5 (range 1–17) | 66 | - | MPE = -0.28mg/d, 37% with ideal dose | None | NI | N | PY | PY | NI | N | High | Y | - |
| 353 | Bress 2012^101^ | Ini-pgx | Sasano 2019^61^ | Japan | AF, DVT, dilated CMP, PE, other | 1.5–3.0 | 125 | - | 100 | - | - | 60 | 73 ± 12 | - | 1.18 | MPE = 0.98, RMSE = 1.34, RMSPE = 99%, 24% with ideal dose | None | NI | Y | PY | PY | NI | Y | Unclear | Y | - |

**Table S7. Continued.**

| **#** | **Algorithm** | **Sub-type** | **Validation study** | **Country** | **Indication** | **Target INR** | **N** | **Ethnicity (%)** | | | | **Male %** | **Age, yrs (mean ± SD or median, IQR)** | **R2** | **MAE (mg/d)** | **Other** | **Excluded participants** | **Missing data** | **PROBAST** | | | | | **ROB** | **Stable dose defined** | **Other key ROB concerns**  **(domains 1-3)** |
| --- | --- | --- | --- | --- | --- | --- | --- | --- | --- | --- | --- | --- | --- | --- | --- | --- | --- | --- | --- | --- | --- | --- | --- | --- | --- | --- |
|  |  |  |  |  |  |  |  | **W** | **A** | **B** | **M** |  |  |  |  |  |  |  | **4.1** | **4.2** | **4.3** | **4.4** | **4.7** |  |  |  |
| 354 | Cini 2012^102^ | Ini-pgx | Cini 2012^102^ | Italy | DVT, HV, PE, other | 2.0–3.5 | 40 | 100 | - | - | - | 58 | 70 ± 16 | 44 | 1.42 | - | Missing data | Excluded | N | PY | N | N | Y | High | Y | - |
| 355 | Lou 2012 | Ini-pgx | Zhao 2014^68^ | China | HV | 1.5–2.5 | 122 | - | 100 | - | - | 31 | 50 ± 10 | - | - | MPE = 0.59, 45% with ideal dose | None | NI | Y | PY | PY | NI | N | High | Y | - |
| 356 |  |  | Dong 2019^246^ | China | AF, PE, DVT, other | 1.6–2.8 | 119 | - | 100 | - | - | 51 | 73 (range 34–90) | - | - | r=0.50, MPE=0.61 mg/d, RMSE=1.27, 35% with ideal dose | Missing genotype data | Excluded | Y | PY | PN | PN | Y | High | Y | - |
| 357 | Moreau 2012^108^ | Ini-pgx | Hamberg 2013^109^  (***Children***) | Sweden | FP, HV, dilated CMP, other | 2.0–3.0 for 90% | 49 | Y | Y | - | - | 53 | 7 (range <1–17) | 65 | 1.06 | MPE=0.32 mg/d, RMSE=0.98 mg/d, 33% had ideal dose | Missing data | Excluded | N | PY | N | N | Y | High | Y | - |
| 358 |  |  | Hamberg 2014^28^  (***Children***) | Sweden | Cardiac | 1.7–3.0 | 31 | 77 | 13 | 6 | 3 | 58 | 3 (range <1–16) | 68 | - | MPE=0.23 mg/d, RMSE=1.11 mg/d, 26% with ideal dose | None | NI | N | PY | PY | NI | Y | High | Y | - |
| 359 |  |  | Marek 2016^110^  (***Children***) | USA | Thrombosis, congenital heart disease, HV, other | 1.5–3.5 for 78% | 32 | 97 | - | - | 3 | 69 | 5 (range 1–17) | 66 | - | MPE = -0.19mg/d, 47% with ideal dose | None | NI | N | PY | PY | NI | N | High | Y | - |
| 360 | Ramirez 2012^114^ | Ini-pgx | Wiley 2017^179^ | USA | VTE, AF | 1.9–3.2 | 2181 | 88 | - | 12 | - | 58 | 66 ± 15 | 48 | 1.31 | - | Missing data | Excluded | Y | PY | N | N | Y | High | Y | - |
| 361 | Tan 2012^115^ | Ini-pgx | Chen 2014^135^ | China | HV | 1.6–2.5 | 787 | - | 100 | - | - | 48 | 54 (44–60) | 56 (for 551) | - | r=0.569, 50% with ideal dose | Missing data | Excluded | Y | PY | N | N | N | High | Y | - |
| 362 |  |  | Xu 2015^245^ | China | HV | 1.8–2.5 | 59 | - | 100 | - | - | 47 (of 193) | 53 ± 12 (for 193) | - | 0.82 | 46% with ideal dose | Loss to follow-up | NI | N | PY | PY | NI | Y | High | Y | - |
| 363 |  |  | Yang 2016^242^ | Korea | AF, CI | - | 310 | - | 100 | - | - | - | mean 69 (26–94) |  |  | r=0.655, MPE=-1.54, 17% with ideal dose | Missing data | Excluded | Y | PY | N | N | N | High | N | - |
| 364 |  |  | Pei 2018^186^ | China | HV | 2.0–3.0 | 358 | - | 100 | - | - | 51 | - | 50 | 0.81 | MAPE=28%, 55% with ideal dose | Missing data | Excluded | Y | PY | N | N | Y | High | Y | - |
| 365 |  |  | Cho 2020^198^ | Korea | AF, CI, DVT, PE, HV | - | 109 | - | 100 | - | - | 62 | 70 (range 29–91) | - | - | r=0.432, MPE=-2.06 mg/d, RMSE = 2.67, 25% with ideal dose | None | NI | Y | PY | PY | NI | Y | Unclear | Y | - |
| 366 |  |  | Xie 2020^141^ | China | HV, AF, other | 1.5–3.0. | 325 | - | 100 | - | - | 51 | 57 ± 13 | 36 | - | Ideal dose = 36%, MPE = -1.63 mg/d | NA | NI | Y | PY | PY | NI | N | High | Y | - |
| 367 | Teh 2012^117^ | Ini-pgx | Teh 2012^117^ | Malaysia | VT, AF, HV | 2.0–4.0 | 28 | - | 100 | - | - | 43 | 60 ± 11 | - | - | r = 0.598 | None | NI | N | PY | PY | NI | N | High | Y | - |
| 368 | Wei 2012^118^ | Ini-pgx | Zhao 2014^68^ | China | HV | 1.5–2.5 | 122 | - | 100 | - | - | 31 | 50 ± 10 | - | - | MPE=0.02 mg/d, 67% with ideal dose | None | NI | Y | PY | PY | NI | N | High | Y | - |
| 369 |  |  | Yang 2016^242^ | Korea | AF, CI | - | 310 | - | 100 | - | - | - | mean 69 (26–94) | - | - | r=0.697, MPE=-1.54, 20% with ideal dose | Missing data | Excluded | Y | PY | N | N | N | High | N | - |
| 370 |  |  | Dong 2019^246^ | China | AF, PE, DVT, other | 1.6–2.8 | 119 | - | 100 | - | - | 51 | 73 (range 34–90) | - | - | r=0.45, MPE=-0.60 mg/d, RMSE=1.10, 45% with ideal dose | Missing genotype data | Excluded | Y | PY | PN | PN | Y | High | Y | - |
| 371 |  |  | Cho 2020^198^ | Korea | AF, CI, DVT, PE, HV | - | 109 | - | 100 | - | - | 62 | 70 (range 29–91) | - | - | r=0.602, MPE = -2.91, RMSE = 3.33, 0% with ideal dose | None | NI | Y | PY | PY | NI | Y | Unclear | Y | - |
| 372 |  |  | Xie 2020^141^ | China | HV, AF, other | 1.5–3.0. | 325 | - | 100 | - | - | 51 | 57 ± 13 | 39 | - | Ideal dose = 39%, MPE = 0.39 mg/d | NA | NI | Y | PY | PY | NI | N | High | Y | - |

**Table S7. Continued.**

| **#** | **Algorithm** | **Sub-type** | **Validation study** | **Country** | **Indication** | **Target INR** | **N** | **Ethnicity (%)** | | | | **Male %** | **Age, yrs (mean ± SD or median, IQR)** | **R2** | **MAE (mg/d)** | **Other** | **Excluded participants** | **Missing data** | **PROBAST** | | | | | **ROB** | **Stable dose defined** | **Other key ROB concerns**  **(domains 1-3)** |
| --- | --- | --- | --- | --- | --- | --- | --- | --- | --- | --- | --- | --- | --- | --- | --- | --- | --- | --- | --- | --- | --- | --- | --- | --- | --- | --- |
|  |  |  |  |  |  |  |  | **W** | **A** | **B** | **M** |  |  |  |  |  |  |  | **4.1** | **4.2** | **4.3** | **4.4** | **4.7** |  |  |  |
| 373 | Xu 2012^22^ | Ini-pgx | Xie 2020^141^ | China | HV, AF, other | 1.5–3.0. | 325 | - | 100 | - | - | 51 | 57 ± 13 | 17 | - | Ideal dose = 17%, MPE = -0.95 mg/d | NA | NI | Y | PY | PY | NI | N | High | Y | - |
| 374 | Zhang 2012^119^ | Ini-pgx | Xie 2020^141^ | China | HV, AF, other | 1.5–3.0. | 325 | - | 100 | - | - | 51 | 57 ± 13 | 12 | - | Ideal dose = 12%, MPE = 1.39 mg/d | NA | NI | Y | PY | PY | NI | N | High | Y | - |
| 375 | Zhong 2012^120^ | Ini-pgx | Chen 2014^135^ | China | HV | 1.6–2.5 | 787 | - | 100 | - | - | 48 | 54 (44–60) | 44 (for 551) | - | r=0.573, 55% with ideal dose | Missing data | Excluded | Y | PY | N | N | N | High | Y | - |
| 376 |  |  | Pei 2018^186^ | China | HV | 2.0–3.0 | 358 | - | 100 | - | - | 51 | - | 46 | 1.05 | MAPE=27%, 43% with ideal dose | Missing data | Excluded | Y | PY | N | N | Y | High | Y | - |
| 377 |  |  | Xie 2020^141^ | China | HV, AF, other | 1.5–3.0. | 325 | - | 100 | - | - | 51 | 57 ± 13 | 12 | - | Ideal dose = 12%, MPE = -1.06 mg/d | NA | NI | Y | PY | PY | NI | N | High | Y | - |
| 378 | Ekladious 2013^123^ | Ini-pgx | Ekladious 2013^123^ | Egypt | AF, DVT, HV, coronary heart diseases | 2.0–3.0 | 34 | - | - | - | 100 | 53 | 44 (34–53) | - | - | r=0.388 | Missing data | Excluded | N | PY | N | N | N | High | Y | - |
| 379 | Nguyen 2013^127^ | Ini-pgx | Hamberg 2014^28^  (***Children***) | Sweden | Cardiac | 1.7–3.0 | 31 | 77 | 13 | 6 | 3 | 58 | 3 (range <1–16) | 76 | - | MPE=0.27 mg/d, RMSE=2.21 mg/d, 26% with ideal dose | None | NI | N | PY | PY | NI | Y | High | Y | - |
| 380 |  |  | Marek 2016^110^  (***Children***) | USA | Thrombosis, congenital heart disease, HV, other | 1.5–3.5 for 78% | 32 | 97 | - | - | 3 | 69 | 5 (range 1–17) | 74 | - | MPE = -2.67mg/d; 19% with ideal dose | None | NI | N | PY | PY | NI | N | High | Y | - |
| 381 | Perera 2013^130^ | Ini-pgx | Perera 2013^130^ | USA | AF, DVT, PE, HV, stroke, other | 2.0–3.0 | 432 | - | - | 100 | - | 39 | 59 ± 16 | 27 | - | - | Missing data | Excluded | Y | PY | N | N | N | High | Y | - |
| 382 | Chen 2014^135^ | Ini-pgx | Chen 2014^135^ | China | HV | 1.6–2.5 | 236 | - | 100 | - | - | 48 | 54 (44–60) | - | - | r =0.609 | Missing data | Excluded | Y | PY | N | N | N | High | Y | - |
| 383 |  |  | Yang 2016^242^ | Korea | AF, CI | - | 310 | - | 100 | - | - | - | mean 69 (26–94) |  |  | r=0.516, MPE=-1.26, 31% with ideal dose | Missing data | Excluded | Y | PY | N | N | N | High | N | - |
| 384 |  |  | Cho 2020^198^ | Korea | AF, CI, DVT, PE, HV | - | 109 | - | 100 | - | - | 62 | 70 (range 29–91) | - | - | r=0.597, MPE=-2.87 mg/d, RMSE=3.28, 4% with ideal dose | None | NI | Y | PY | PY | NI | Y | Unclear | Y | - |
| 385 |  |  | Xie 2020^141^ | China | HV, AF, other | 1.5–3.0. | 325 | - | 100 | - | - | 51 | 57 ± 13 | 24 | - | Ideal dose = 24%, MPE = 0.92 mg/d | NA | NI | Y | PY | PY | NI | N | High | Y | - |
| 386 | Lou 2014 | Ini-pgx | Xie 2020^141^ | China | HV, AF, other | 1.5–3.0. | 325 | - | 100 | - | - | 51 | 57 ± 13 | 20 | - | Ideal dose = 20%, MPE = 1.1 mg/d | NA | NI | Y | PY | PY | NI | N | High | Y | - |
| 387 | Shaw 2014^144^ | Ini-pgx | Marek 2016^110^  (***Children***) | USA | Thrombosis, congenital heart disease, HV, other | 1.5–3.5 for 78% | 32 | 97 | - | - | 3 | 69 | 5 (range 1–17) | 58 | - | MPE=-0.48mg/d, 41% with ideal dose | None | NI | N | PY | PY | NI | N | High | Y | - |
| 388 | Vear 2014^146^ | Ini-pgx | Marek 2016^110^  (***Children***) | USA | Thrombosis, congenital heart disease, HV, other | 1.5–3.5 for 78% | 32 | 97 | - | - | 3 | 69 | 5 (range 1–17) | 35 | - | MPE=-0.63mg/d, 34% with ideal dose | None | NI | N | PY | PY | NI | N | High | Y | - |
| 389 | Karaca 2015^154^ | Ini-pgx | Karaca 2015^154^ | Turkey | AF, HV, CVA, DVT, PE, other | - | 92 | - | - | - | 100 | 51 | 67 ± 12 | - | 1.29 | r=0.426, 31% with ideal dose | None | NI | N | PY | PY | NI | Y | High | N | - |
| 390 | Li 2015^18^ | Ini-pgx | Li 2015^18^ | IWPC | AF, DVT, PE, HV, stroke, other | 2.0–3.0 | 216 | - | 100 | - | - | - | - | - | 0.61 | 56% with ideal dose | Missing data | Excluded | Y | PY | N | N | Y | High | Y | - |
| 391 |  |  |  | IWPC | AF, DVT, PE, HV, stroke, other | 2.0–3.0 | 216 | - | 100 | - | - | - | - | - | 0.67 | 53% with ideal dose | Missing data | Excluded | Y | PY | N | N | Y | High | Y | - |
| 392 |  |  |  | IWPC | AF, DVT, PE, HV, stroke, other | 2.0–3.0 | 216 | - | 100 | - | - | - | - | - | 0.67 | 53% with ideal dose | Missing data | Excluded | Y | PY | N | N | Y | High | Y | - |
| 393 |  |  |  | IWPC | AF, DVT, PE, HV, stroke, other | 2.0–3.0 | 216 | - | 100 | - | - | - | - | - | 0.69 | 52% with ideal dose | Missing data | Excluded | Y | PY | N | N | Y | High | Y | - |
| 394 |  |  |  | IWPC | AF, DVT, PE, HV, stroke, other | 2.0–3.0 | 216 | - | 100 | - | - | - | - | - | 0.65 | 55% with ideal dose | Missing data | Excluded | Y | PY | N | N | Y | High | Y | - |
| 395 |  |  |  | IWPC | AF, DVT, PE, HV, stroke, other | 2.0–3.0 | 216 | - | 100 | - | - | - | - | - | 0.65 | 54% with ideal dose | Missing data | Excluded | Y | PY | N | N | Y | High | Y | - |
| 396 |  |  |  | IWPC | AF, DVT, PE, HV, stroke, other | 2.0–3.0 | 216 | - | 100 | - | - | - | - | - | 0.63 | 55% with ideal dose | Missing data | Excluded | Y | PY | N | N | Y | High | Y | - |

**Table S7. Continued.**

| **#** | **Algorithm** | **Sub-type** | **Validation study** | **Country** | **Indication** | **Target INR** | **N** | **Ethnicity (%)** | | | | **Male %** | **Age, yrs (mean ± SD or median, IQR)** | **R2** | **MAE (mg/d)** | **Other** | **Excluded participants** | **Missing data** | **PROBAST** | | | | | **ROB** | **Stable dose defined** | **Other key ROB concerns**  **(domains 1-3)** |
| --- | --- | --- | --- | --- | --- | --- | --- | --- | --- | --- | --- | --- | --- | --- | --- | --- | --- | --- | --- | --- | --- | --- | --- | --- | --- | --- |
|  |  |  |  |  |  |  |  | **W** | **A** | **B** | **M** |  |  |  |  |  |  |  | **4.1** | **4.2** | **4.3** | **4.4** | **4.7** |  |  |  |
| 397 | Limdi 2015^157^ | Ini-pgx | Xie 2020^141^ | China | HV, AF, other | 1.5–3.0. | 325 | - | 100 | - | - | 51 | 57 ± 13 | 22 | - | Ideal dose = 22%, MPE = 1.01 mg/d | NA | NI | Y | PY | PY | NI | N | High | Y | - |
| 398 | Limdi 2015^157^ | Ini-pgx | Xie 2020^141^ | China | HV, AF, other | 1.5–3.0. | 325 | - | 100 | - | - | 51 | 57 ± 13 | 17 | - | Ideal dose = 17%, MPE = 1.22 mg/d | NA | NI | Y | PY | PY | NI | N | High | Y | - |
| 399 | Santos 2015^160^ | Ini-pgx | Santos 2015^160^ | Brazil | AF, CVA, TE, HV, other | 1.8–3.2 | 133 | 56 | - | 17 | 26 | 56 | 63 ± 17 | 61 | - | - | None | NI | Y | PY | PY | NI | N | High | Y | - |
| 400 | Alzubiedi 2016^161^ | Ini-pgx | Cho 2020^198^ | Korea | AF, CI, DVT, PE, HV | - | 109 | - | 100 | - | - | 62 | 70 (range 29–91) | - | - | r=0.57, MPE=-1.32, RMSE=2.12, 51% with ideal dose | None | NI | Y | PY | PY | NI | Y | Unclear | Y | - |
| 401 | Cho 2016^162^ | Ini-pgx | Cho 2020^198^ | Korea | AF, CI, DVT, PE, HV | - | 109 | - | 100 | - | - | 62 | 70 (range 29–91) | - | - | r=0.61, MPE = -0.88, RMSE = 1.72, 56% with ideal dose | None | NI | Y | PY | PY | NI | Y | Unclear | Y | - |
| 402 | Jiang 2016^164^ | Ini-pgx | Jiang 2016^164^ | China | AF | 1.8–3.0 | 45 | - | 100 | - | - | 58 (of 60) | 57 (for 60) | - | - | r=0.822 | Recruited vs analyzed don't match | NI | N | PY | PN | NI | N | High | Y | - |
| 403 | Li 2016 | Ini-pgx | Xie 2020^141^ | China | HV, AF, other | 1.5–3.0. | 325 | - | 100 | - | - | 51 | 57 ± 13 | 22 | - | Ideal dose = 22%, MPE = 0.72 mg/d | NA | NI | Y | PY | PY | NI | N | High | Y | - |
| 404 | Zeng 2016^170^ | Ini-pgx | Xie 2020^141^ | China | HV, AF, other | 1.5–3.0. | 325 | - | 100 | - | - | 51 | 57 ± 13 | 31 | - | Ideal dose = 31%, MPE = -0.59 mg/d | NA | NI | Y | PY | PY | NI | N | High | Y | - |
| 405 | Liu 2017^173^ | Ini-pgx | Xie 2020^141^ | China | HV, AF, other | 1.5–3.0. | 325 | - | 100 | - | - | 51 | 57 ± 13 | 20 | - | Ideal dose = 20%, MPE = -0.8 mg/d | NA | NI | Y | PY | PY | NI | N | High | Y | - |
| 406 | Galvez 2018^183^ | Ini-pgx | Galvez 2018^183^ | Colombia | HV, DVT, PE, CVD, other | 2.0–3.0 | 87 | - | - | - | 100 | 53 | 62 ± 12 | 46 | - | - | None | NI | N | PY | PY | NI | N | High | Y | - |
| 407 | Pei 2018^186^ | Ini-pgx | Pei 2018^186^ | China | HV | 2.0–3.0 | 111 | - | 100 | - | - | 51 | 69 (59–76) | - | - | r=0.722 | Missing data | Excluded | Y | PY | N | N | N | High | Y | - |
| 408 |  |  | Xie 2020^141^ | China | HV, AF, other | 1.5–3.0. | 325 | - | 100 | - | - | 51 | 57 ± 13 | 20 | - | Ideal dose = 20%, MPE = 1.1 mg/d | NA | NI | Y | PY | PY | NI | N | High | Y | - |
| 409 | Tavares 2018^189^ | Ini-pgx | Tavares 2018^189^ | Brazil | AF, CVA, TE, HV, other | 1.8–3.2 | 133 | 58 | - | 18 | 24 | 56 | 63 ± 17 | - | - | r=0.6075 | Missing data | Excluded | Y | PY | N | N | N | High | Y | - |
| 410 | Tavares 2018^189^ | Ini-pgx | Tavares 2018^189^ | Brazil | AF, CVA, TE, HV, other | 1.8–3.2 | 133 | 58 | - | 18 | 24 | 56 | 63 ± 17 | - | - | r=0.6078 | Missing data | Excluded | Y | PY | N | N | N | High | Y | - |
| 411 | Caldwell 2008^46^ | Ini-cl | Burmester 2011^47^ | USA | AF, DVT, PE, HV | 2.0–3.5 | 85 | 100 | - | - | - | 61 (of 115) | 69 (range 40–91) (for 115) | - | 1.32 median | - | None | NI | N | PY | PY | PY | Y | High | Y | - |
| 412 | Gage 2008^a^ | Ini-cl | Gage 2008^48^ | USA | AF, VTE, HV | 1.5–3.2 | 292 | 83 | - | 15 | 2 | 48 | 57 ± 14 | 17 | 1.8 | - | None | NA | Y | PY | PY | NI | Y | Unclear | Y | - |
| 413 |  |  | Finkelman 2011^247^ | 9 (IWPC sites) | AF, DVT, PE, HV, stroke, other | mainly 2.0–3.0 | 1378 | - | - | - | Y | - | - | - | - | 39% with ideal dose | Missing data | Excluded | Y | PY | N | N | N | High | Y |  |
| 414 |  |  | Schwartz 2011^248^ | USA | AF, DVT, PE, other | 2.0–3.0 | 69 | 83 | 14 | 3 | - | 46 | 81 ± 8 (all <65) | 12 | - | - | None | NI | N | PY | PY | NI | N | High | Y | - |
| 415 |  |  | Bazan 2012^249^ | Egypt | AF, A/VTE, HV | 2.0–3.5 | 63 | - | - | - | 100 | 49 | 46 ± 13 | 24 | 3.7 | 24% within 1 mg/d | None | NI | N | PY | PY | NI | Y | High | Y | - |
| 416 |  |  | Horne 2012^211^ | Sweden,  South Korea,  Thailand,  UK, USA | Orthopedic (total knee or hip replacement), DVT, PE, AF, stroke, HV, other | Various | 43 | 84 | - | 14 | 2 | 42 | 73 ± 6 | 24 | 1.07 median | - | Missing data | Excluded | N | PY | N | N | Y | High | Y | - |
| 417 |  |  | Kimmel 2013^251^,  French 2016^252^ | USA | AF, DVT, PE, other | 2.0–3.0 | 349 | 74 | - | 26 | - | 49 (of 501) | 57 (46–68) (for 501) | 27 | 1.50 | r=0.54, 42% within 1 mg/d | None | NI | Y | PY | PY | PY | Y | Low | Y | - |
| 418 |  |  | Sharabiani 2013^204^ | USA | DVT, PE, other | 2.0–3.0 | ~59 | - | - | 100 | - | - | - | - | 2.13 | RMSE=2.33 mg/d | Outliers | Single imputation | N | PY | N | PN | Y | High | Y | - |

**Table S7. Continued.**

| **#** | **Algorithm** | **Sub-type** | **Validation study** | **Country** | **Indication** | **Target INR** | **N** | **Ethnicity (%)** | | | | **Male %** | **Age, yrs (mean ± SD or median, IQR)** | **R2** | **MAE (mg/d)** | **Other** | **Excluded participants** | **Missing data** | **PROBAST** | | | | | **ROB** | **Stable dose defined** | **Other key ROB concerns**  **(domains 1-3)** |
| --- | --- | --- | --- | --- | --- | --- | --- | --- | --- | --- | --- | --- | --- | --- | --- | --- | --- | --- | --- | --- | --- | --- | --- | --- | --- | --- |
|  |  |  |  |  |  |  |  | **W** | **A** | **B** | **M** |  |  |  |  |  |  |  | **4.1** | **4.2** | **4.3** | **4.4** | **4.7** |  |  |  |
| 419 | Gage 2008^a48^ | Ini-cl | Sharabiani 2018^218^ | USA | AF, DVT, PE, HV, CVA, other | 1.8–3.5 | ~45 | Y | - | Y | - | 45 (for 150) | 54 ± 17 (for 150) | - | - | RMSE = 2.05 | None | Single imputation | N | PY | PY | N | Y | High | N | - |
| 420 |  |  | Saffian 2015^254^ | New  Zealand | AF, DVT, PE | 2.0–3.0 | 46 | Y | - | - | - | 41 | 62 (range 29–87) | - | - | MPE=-0.76 mg/d, RMSE=2.12 mg/d | Missing data | Single imputation | N | PY | N | N | Y | High | Y | - |
| 421 |  |  | Sharabiani 2015^205^ | 9 (IWPC) | AF, DVT, PE, HV, CHF, other | 1.8–3.5 | 2118 | 63 | 22 | 15 | - | 43 (of 4237) | Categorized | - | 1.41 | - | None | Single imputation | Y | PY | PY | N | Y | High | Y | - |
| 422 |  |  | Shahabi 2016^168^ | USA | AF, DVT, PE, HV, Other | 2.0–3.0 | 73 | 93 | - | 4 | 3 | 67 | 66 ± 13 | - | 1.5 | 38% within 1mg | Missing data | Excluded | N | PY | N | N | Y | High | Y | Sub-optimal definition |
| 423 |  |  | Shahabi 2016^168^ | Canada | AF, HV, mitral stenosis, Other | 2.0–3.5 | 769 | 97 | <1 | 1 | 3 | 59 | 73 ± 10 | - | 1.4 | 45% within 1mg | Missing data | Excluded | N | PY | N | N | Y | High | Y |  |
| 424 |  |  | Selim 2018^187^ | Egypt | AF, DVT, PE, HV | 2.0–3.2 | 100 | - | - | - | 100 | 46 | 45 ± 13 | 19 | - | 37% within 1 mg/d | None | NI | Y | PY | PY | NI | N | High | Y | - |
| 425 |  |  | Asiimwe 2020 | South Africa  Uganda | AF, VTE, HV | 2.0–3.5 | 364 | - | - | 100 | - | 27 | 46 ± 15 | 16 | 1.81 | Unbiased MAPE = 38%, 37% with ideal dose | None | Multiple imputation | Y | Y | Y | Y | Y | Low | Y | - |
| 426 | IWPC 2009^3^ | Ini-cl | Sagreiya 2010^78^ | USA | - | 2.0–3.0 | 104 | 75 | 17 | 8 | - | 58 | 67 (53–75) | 19 | 1.49 | 54% within 1 mg/d | None | NI | Y | PY | PY | NI | Y | Unclear | Y | - |
| 427 |  |  | Takeuchi 2010^23^ | Japan | AF, HV, DVT/PE, other | 1.6–2.6 | 200 | - | 100 | - | - | 68 | 67 ± 10 | - | - | 64% within 1mg/d | None | NI | Y | PY | PY | NI | N | High | N | - |
| 428 |  |  | Bazan 2012^249^ | Egypt | AF, AVT, HV | 2.0–3.5 | 63 | - | - | - | 100 | 49 | 46 ± 13 | 25 | 3.7 | 22% within 1 mg/d | None | NA | N | PY | PY | NI | Y | High | Y | - |
| 429 |  |  | Valentin 2012^261^ | Puerto Rico | AF, DVT, PE, CVA, stroke, HV, other | 2.0–3.5 | 97 | - | - | - | 100 | 99 | 67 ± 8 | 0-4 | 1.64 | 48% with ideal dose | Missing data | Excluded | N | PY | N | N | Y | High | Y | - |
| 430 |  |  | Sharabiani 2013^204^ | USA | DVT, PE, other | 2.0–3.0 | ~59 | - | - | 100 | - | - | - | - | 1.87 | RMSE = 2.27 | Outliers | Single imputation | N | PY | N | PN | Y | High | Y | - |
| 431 |  |  | Hernandez 2014^90^ | USA | AF, DVT, PE, others | - | 149 | - | - | 100 | - | 53 | 59 ± 16 | 16 | 2.56 | 26% with ideal dose | Missing data | Excluded | Y | PY | N | N | Y | High | Y | - |
| 432 |  |  | Saffian 2015^254^ | New  Zealand | AF, DVT, PE | 2.0–3.0 | 46 | Y | - | - | - | 41 | 62 (range 29–87) | - | - | MPE=-1.01 mg/d, RMSE=2.26 | Missing data | Single imputation | N | PY | N | N | Y | High | Y | - |
| 433 |  |  | Eriksson 2016^163^ | 44 (RE­LY  sub-study) | AF | 2.0–3.0 | 951 | 87 | 2 | 1 | 10 | 66 (of 956) | 72 (68–77) (for 956) | 13 | - | 43% with ideal dose | None | NI | Y | PY | PY | NI | N | High | Y | - |
| 434 |  |  | French 2016^252^ | USA | AF, DVT, PE, other | 2.0–3.0 | 349 | 74 | - | 26 | - | 49 (of 501) | 57 (46–68) (for 501) | 30 | 1.50 | r=0.54, 41% within 1 mg/d | None | NI | Y | PY | PY | PY | Y | Low | Y | - |
| 435 |  |  | Stack 2016^262^ | USA | AF, DVT, PE, other | 2.0–3.0 | 146 | 75 | <1 | 6 | 19 | 100 | 73 ± 11 | - | 1.58 | 48% with ideal dose, 27% within 0.5 mg/d | Missing data | Excluded | Y | PY | N | N | Y | High | Y | - |
| 436 |  |  | Ayesh 2018^264^ | Gaza-  Palestine | VTE, HV, other | 2.0–3.5 | 47 | - | - | - | 100 | 43 (of 101) | 49 ± 14 (for 101) | 11 | 1.49 | 53% with ideal dose | None | NI | N | PY | PY | NI | Y | High | Y | - |
| 437 |  |  | Selim 2018^187^ | Egypt | AF, DVT, PE, HV | 2.0–3.2 | 100 | - | - | - | 100 | 46 | 45 ± 13 | 9 |  | 36% within 1 mg/d | None | NI | Y | PY | PY | NI | N | High | Y | - |
| 438 |  |  | Asiimwe 2020 | South Africa  Uganda | AF, VTE, HV | 2.0–3.5 | 364 | - | - | 100 | - | 27 | 46 ± 15 | 13 | 1.79 | Unbiased MAPE = 37%, 38% with ideal dose | None | Multiple imputation | Y | Y | Y | Y | Y | Low | Y | - |
| 439 | Cen 2010^66^ | Ini-cl | Xu 2018^67^ | China | HV | 1.8–2.5 | 101 | - | 100 | - | - | 45 | 48 ± 10 | 10 | 0.73 | 51% within 0.6 mg/d, 35% within 0.5mg/d | None | NI | Y | PY | PY | NI | Y | Unclear | Y | - |
| 440 | Sharabiani 2013^204^ | Ini-cl | Sharabiani 2015^205^ | 9 (IWPC) | AF, DVT, PE, HV, CHF, other | 1.8–3.5 | 2118 | 63 | 22 | 15 | - | 43 (of 4237) | Categorized | - | 1.81 | - | None | Single imputation | Y | PY | PY | N | Y | High | Y | - |
| 441 | Asiimwe 2020 | Ini-cl | Asiimwe 2020 | 9 (IWPC) | AF, DVT, PE, HV, stroke, other | 2.0–3.5 | 690 | - | - | 100 | - | 43 | Categorized | 12 | 1.79 | Unbiased MAPE = 36%, 42% with ideal dose | None | Multiple imputation | Y | Y | Y | Y | Y | Low | Y | - |
| 442 | Li 2020^206^ | Ini-cl | Li 2020^206^ | China | HV | 1.5–2.5 | 1639 | - | 100 | - | - | 42 | 50 ± 11 | - | 0.74 | MSE = 0.599, RMSE = 0.774, ideal dose = 60% | None | NI | Y | PY | PY | NI | Y | Unclear | Y | - |

**Table S7. Continued.**

| **#** | **Algorithm** | **Sub-type** | **Validation study** | **Country** | **Indication** | **Target INR** | **N** | **Ethnicity (%)** | | | | **Male %** | **Age, yrs (mean ± SD or median, IQR)** | **R2** | **MAE (mg/d)** | **Other** | **Excluded participants** | **Missing data** | **PROBAST** | | | | | **ROB** | **Stable dose defined** | **Other key ROB concerns**  **(domains 1-3)** |
| --- | --- | --- | --- | --- | --- | --- | --- | --- | --- | --- | --- | --- | --- | --- | --- | --- | --- | --- | --- | --- | --- | --- | --- | --- | --- | --- |
|  |  |  |  |  |  |  |  | **W** | **A** | **B** | **M** |  |  |  |  |  |  |  | **4.1** | **4.2** | **4.3** | **4.4** | **4.7** |  |  |  |
| 443 | Li 2020^206^ | Ini-cl | Li 2020^206^ | China | HV | 1.5–2.5 | 1639 | - | 100 | - | - | 42 | 50 ± 11 | - | 0.75 | MSE = 0.601, RMSE = 0.775, ideal dose = 57% | None | NI | Y | PY | PY | NI | Y | Unclear | Y | - |
| 444 | Lenzini 2008^208^ | Rev-pgx | Lenzini 2008^208^ | USA | Orthopedic (total knee or hip replacement) | 1.5–2.7 for most | 146 | Y | - | Y | - | - | - | 70 | 0.73  median | 73% within 1 mg | Missing data | Excluded | Y | PY | N | N | Y | High | Y | - |
| 445 | Michaud 2008^209^ | Rev-pgx | Marin-Leblanc 2012^250^ | Canada | AF, HV | 2.0–3.5 | 454 | 100 | - | - | - | 68 | 67 ± 11 | 29 | 1.95 | 32% with ideal dose | None | NI | Y | PY | PY | NI | Y | Unclear | Y | - |
| 446 | Lenzini 2010^210^ | Rev-pgx | Lenzini 2010^210^ | USA, UK, South Korea | AF, DVT, PE, HV, stroke, orthopedic, other | Target 2.5 | 584 | Y | Y | Y | - | - | - | 40, 42 | 0.99, 0.96  median | - | Missing data | Excluded | Y | PY | N | N | Y | High | Y | - |
| 447 |  |  | Anderson 2012^99^ | US | DVT, PE, AF, other | 1.5–3.5 | 238 | 96 | - | - | - | 48 (of 247) | mean 60 (range 18–85) (for 247) | - | 0.99 | - | None | NA | Y | PY | Y | NI | Y | Unclear | Y | Some stable doses estimated |
| 448 |  |  | Liu 2012^237^ | China | AF, HV, DVT, PE | 1.6–2.5 | 282 | - | 100 | - | - | 50 | 56 ± 16 | - | 0.72 | 52.1% with ideal dose | None | NI | Y | PY | PY | NI | Y | Unclear | Y | - |
| 449 |  |  | Ramos 2012^212^ | Puerto Rico | AF, PTE, other | 2.0–3.5 | 163 | 90 | - | 10 | - | 99 | 68 ± 9 | 33 | 1.27 | MAPE=28% | Missing data | Excluded | Y | PY | N | N | Y | High | Y | - |
| 450 |  |  | Ramos 2012^212^,  Duconge 2016^213^ | USA | AF, PTE, other | 2.0–3.5 | 55 | 93 | - | 7 | - | 36 | 61 ± 14 | 27 | 1.77,  1.62 | MAPE=35%, 29% | Missing data | Excluded | N | PY | N | N | Y | High | Y | - |
| 451 |  |  | Saffian 2015^254^ | New  Zealand | AF, DVT, PE | 2.0–3.0 | 46 | Y | - | - | - | 41 | 62 (range 29–87) | - | - | MPE=-0.18 mg/d, RMSE = 1.19 mg/d | Missing data | Single imputation | N | PY | N | N | Y | High | Y | - |
| 452 |  |  | Duconge 2016^213^ | Puerto Rico | AF, DVT, PE, other | 2.0–3.5 | 255 | 89 | - | 11 | - | 99 | 68 ± 10 | 7-24 | 0.63- 3.20 | 14-45% with ideal dose, MAPE=26-40% | Missing data | Excluded | Y | PY | N | N | Y | High | Y | - |
| 453 |  |  | Cho 2020^198^ | Korea | AF, CI, DVT, PE, HV | - | 109 | - | 100 | - | - | 62 | 70 (range 29–91) | - | - | r=0.767, MPE=-0.2 mg/d, RMSE = 1.39, 60% with ideal dose | None | NI | Y | PY | PY | NI | Y | Unclear | Y | - |
| 454 | Horne 2012^211^ | Rev-pgx | Karaca 2015^154^ | Turkey | AF, HV, CVA, DVT, PE, other | - | 97 | - | - | - | 100 | 49 | 61 ± 13 | - | 2.94 | r = 0.604, 5% with ideal dose | None | NI | N | PY | PY | NI | Y | High | N | - |
| 455 |  |  | Karaca 2015^154^ | Turkey | AF, HV, CVA, DVT, PE, other | - | 92 | - | - | - | 100 | 51 | 67 ± 12 | - | 2.74 | r = 0.558, 2% with ideal dose | None | NI | N | PY | PY | NI | Y | High | N | - |
| 456 |  |  | Saffian 2015^254^ | New  Zealand | AF, DVT, PE | 2.0–3.0 | 46 | Y | - | - | - | 41 | 62 (range 29–87) | - | - | MPE=-0.52 mg/d, RMSE=1.16 mg/d | Missing data | Single imputation | N | PY | N | N | Y | High | Y | - |
| 457 | Ramos 2012^212^ | Rev-pgx | Ramos 2012^212^ | USA | AF, PTE, other | 2.0–3.5 | 55 | 93 | - | 7 | - | 36 | 61 ± 14 | 51 | 1.38 | MAPE=32% | Missing data | Excluded | N | PY | N | N | Y | High | Y | - |
| 458 | Duconge 2016^213^ | Rev-pgx | Duconge 2016^213^ | Puerto Rico | AF, DVT, PE and other | 2.0–3.5 | 55 | 93 | - | 7 | - | 36 | 61 ± 14 | 59 | 0.89 | MAPE=24% | Missing data | Excluded | N | PY | N | N | Y | High | Y | - |
| 459 | Solomon 2004^215^ | Rev-cl | Francis 2014^239^ | UK | AF, VTE | 2.0–3.0 | 508 | 100 | - | - | - | 55 | 68 | 34 | 1.21 | MAPE = 36% | None | NI | Y | PY | PY | NI | Y | Unclear | Y | - |
| 460 |  |  | Francis 2014^239^ | UK/Sweden | AF, VTE | 2.0–3.0 | 133 | 100 | - | - | - | 59 | 67 | 69 | 1 | MAPE = 20% | None | NI | Y | PY | PY | NI | Y | Unclear | Y | - |
| 461 | Lenzini 2007^216^ | Rev-cl | Lenzini 2007^216^ | USA | Total knee or hip arthroplasty | 1.7–3.0 | 105 | 88 | - | 12 | - | 50 | 56 ± 15 | 22 | 1.20  median | 44% within 1 mg/d | None | NI | Y | PY | PY | NI | Y | Unclear | Y | - |
| 462 |  |  |  | USA | Total knee or hip arthroplasty | 1.7–3.0 | 105 | 88 | - | 12 | - | 50 | 56 ± 15 | 42 | 0.9  median | 54% within 1 mg/d | None | NI | Y | PY | PY | NI | Y | Unclear | Y | - |
| 463 | Lenzini 2008^208^ | Rev-cl | Lenzini 2008^208^ | USA | Total knee or hip replacement | 1.5–2.7 for most | 146 | Y | - | Y | - | - | - | 48 | 0.74  median | 70% within 1 mg/d | Missing data | Excluded | Y | PY | N | N | Y | High | Y | - |
| 464 | Lenzini 2010^210^ | Rev-cl | Lenzini 2010^210^ | USA, UK, South Korea | AF, DVT, PE, HV, stroke, orthopedic, other | Target 2.5 | 584 | Y | Y | Y | - | - | - | 26-28 | 0.91-0.99  median | - | Missing data | Excluded | Y | PY | N | N | Y | High | Y | - |

**Table S7. Continued.**

| **#** | **Algorithm** | **Sub-type** | **Validation study** | **Country** | **Indication** | **Target INR** | **N** | **Ethnicity (%)** | | | | **Male %** | **Age, yrs (mean ± SD or median, IQR)** | **R2** | **MAE (mg/d)** | **Other** | **Excluded participants** | **Missing data** | **PROBAST** | | | | | **ROB** | **Stable dose defined** | **Other key ROB concerns**  **(domains 1-3)** |
| --- | --- | --- | --- | --- | --- | --- | --- | --- | --- | --- | --- | --- | --- | --- | --- | --- | --- | --- | --- | --- | --- | --- | --- | --- | --- | --- |
|  |  |  |  |  |  |  |  | **W** | **A** | **B** | **M** |  |  |  |  |  |  |  | **4.1** | **4.2** | **4.3** | **4.4** | **4.7** |  |  |  |
| 465 | Lenzini 2010^210^ | Rev-cl | Horne 2012^211^ | Sweden,  South Korea,  Thailand,  UK, USA | Orthopedic (total knee or hip replacement), DVT, PE, AF, stroke, HV, other | Various | 42 | 84 | - | 14 | 2 | 42 | 73 ± 6 | 39 | 1.04  median | - | Missing data | Excluded | N | PY | N | N | Y | High | Y | - |
| 466 |  |  | Kimmel 2013^251^ | USA | AF, DVT, PE, other | 2.0–3.0 | 349 | 74 | - | 26 | - | 49 (of 501) | 57 (46–68) (for 501) | 54 | 1.10 | - | None | NI | Y | PY | PY | PY | Y | Low | Y | - |
| 467 |  |  | Saffian 2015^254^ | New  Zealand | AF, DVT, PE | 2.0–3.0 | 46 | Y | - | - | - | 41 | 62 (range 29–87) | - | - | MPE=0.27 mg/d, RMSE=1.49 mg/d | Missing data | Single imputation | N | PY | N | N | Y | High | Y | - |
| 468 | Moreau 2011^88^ | Rev-cl | Moreau 2011^88^ | France | ATE, VTE | 2.0–3.0 | 55 | 100 | - | - | - | 31 | 84 ± 6 | 60 | - | - | None | NI | N | PY | PY | NI | N | High | Y | - |
| 469 | Horne 2012^211^ | Rev-cl | Horne 2012^211^ | Sweden,  South Korea,  Thailand,  UK, USA | Orthopedic (total knee or hip replacement), DVT, PE, AF, stroke, HV, other | Various | 26  /35 | 84 | - | 14 | 2 | 42 | 73 ± 6 | 58, 79 | 0.81, 0.38 | - | Missing data | Excluded | N | PY | N | N | Y | High | Y | - |
| 470 |  |  | Saffian 2015^254^ | New  Zealand | AF, DVT, PE | 2.0–3.0 | 46 | Y | - | - | - | 41 | 62 (range 29–87) | - | - | MPE=-0.42 mg/d, RMSE=1.22 mg/d | Missing data | Single imputation | N | PY | N | N | Y | High | Y | - |
| 471 | Duconge 2016^213^ | Rev-cl | Duconge 2016^213^ | Puerto Rico | AF, DVT, PE, other | 2.0–3.5 | 55 | 93 | - | 7 | - | 36 | 61 ± 14 | 51 | 1.34 | MAPE=34% | Missing data | Excluded | N | PY | N | N | Y | High | Y | - |
| 472 | Li 2018^19^ | Rev-cl | Li 2018^19^ | China | HV | 1.5–2.5 | 1463 | - | 100 | - | - | 44 | 49 ± 11 | - | 0.37 | RMSE=0.66 mg/d, 63% with ideal dose | Missing data | Excluded | Y | PY | N | N | Y | High | Y | - |
| 473 | Tao 2018^219^ | Rev-cl | Tao 2018^219^ | China | HV | 1.5–2.5 for most | 1639 | - | 100 | - | - | 52 | 50 ± 11 | - | 0.60 | r=0.066, MSE = 0.74, 60% with ideal dose | None | NI | Y | PY | PY | NI | Y | Unclear | N | - |
| 474 |  |  |  | China | HV | 1.5–2.5 for most | 1639 | - | 100 | - | - | 52 | 50 ± 11 | - | 0.62 | r = 0.101, MSE = 0.74, 57% with ideal dose | None | NI | Y | PY | PY | NI | Y | Unclear | N | - |
| 475 | Hamberg 2010^224^ | Pgx | Dong 2019^246^ | China | AF, PE, DVT, other | 1.6–2.8 | 119 | - | 100 | - | - | 51 | 73 (range 34–90) | - | - | A priori: r=0.54, MPE = 0.01, RMSE = 1.00, 56% with ideal dose  A posteriori: r=0.86, MPE=0.17, RMSE = 0.69, 75% ideal dose | Missing genotype data | Excluded | Y | PY | PN | PN | Y | High | Y | - |
| 476 | Lala 2013^226^ | Pgx | Hamberg 2014^28^  (***Children***) | Sweden | Cardiac | 1.7–3.0 | 31 | 77 | 13 | 6 | 3 | 58 | 3 (range <1–16) | 77 | - | PE= -1.18 mg/d, RMSE=1.58 mg/d, 13% with ideal dose | None | NI | N | PY | PY | NI | Y | High | Y | - |
| 477 |  |  | Marek 2016^110^  (***Children***) | USA | Thrombosis, congenital heart disease, HV, other | 1.5–3.5 for 78% | 32 | 97 | - | - | 3 | 69 | 5 (range 1–17) | 60 | - | MPE=0.85 mg/d; 19% with ideal dose | None | NI | N | PY | PY | NI | N | High | Y | - |
| 478 | Hamberg 2013^109^ | Pgx | Hamberg 2013^109^  (***Children***) | Sweden | FP, HV, dilated CMP, other | 2.0–3.0 for 90% | 49 | Y | Y | - | - | 53 | 7 (range <1–17) | 63,86 | 0.94, 0.57 | A priori: MPE=-0.13, RMSE= 0.94, 41% with ideal dose  A posteriori: MPE=-0.04, RMSE= 0.57, 70% with ideal dose | Missing data | Excluded | N | PY | N | N | Y | High | Y | - |

**Table S7. Continued.**

| **#** | **Algorithm** | **Sub-type** | **Validation study** | **Country** | **Indication** | **Target INR** | **N** | **Ethnicity (%)** | | | | **Male %** | **Age, yrs (mean ± SD or median, IQR)** | **R2** | **MAE (mg/d)** | **Other** | **Excluded participants** | **Missing data** | **PROBAST** | | | | | **ROB** | **Stable dose defined** | **Other key ROB concerns**  **(domains 1-3)** |
| --- | --- | --- | --- | --- | --- | --- | --- | --- | --- | --- | --- | --- | --- | --- | --- | --- | --- | --- | --- | --- | --- | --- | --- | --- | --- | --- |
|  |  |  |  |  |  |  |  | **W** | **A** | **B** | **M** |  |  |  |  |  |  |  | **4.1** | **4.2** | **4.3** | **4.4** | **4.7** |  |  |  |
| 479 | Hamberg 2013^109^ | Pgx | Hamberg 2014^28^  (***Children***) | Sweden | Cardiac | 1.7–3.0 | 31 | 77 | 13 | 6 | 3 | 58 | 3 (range <1–16) | 78 | - | MPE=-0.39 mg/d, RMSE=0.99 mg/d, 45% with ideal dose | None | NI | N | PY | PY | NI | Y | High | Y | - |
| 480 |  |  | Marek 2016^110^  (***Children***) | USA | Thrombosis, congenital heart disease, HV, other | 1.5–3.5 for 78% | 32 | 97 | - | - | 3 | 69 | 5 (range 1–17) | 78 | - | MPE=0.15mg/d; 38% with ideal dose | None | NI | N | PY | PY | NI | N | High | Y | - |
| 481 |  |  | Al-Metwali 2019^268^ (says children but range includes adults) | UK | FP, HV, other | 1.5–4.0 | 60 | 72 | 13 | - | 15 | 65 | 17 (range 8–67) | - | - | Ideal dose = 70%, MPE = -0.10, RMSE = 0.19 | None | NI | N | PY | PY | NI | Y | High | Y | - |

^a^Any external validation study that used warfarindosing.org to calculate dose was considered under Gage 2008^48^. Nevertheless, many other studies including Millican 2007^207^, Lenzini 2008^208^, IWPC 2009^3^, King 2010^70^ etc. have contributed to the data and algorithms available at warfarindosing.org. Abbreviations: N, no; NI, no information; PN, probably no; PY, probably yes; ROB, risk of bias; Y, yes; For other abbreviations, see Table S5.

**Table S8. Clinical utility assessments^a^**

| **#** | **Algorithm** | **Sub-type** | **Clinical utility study** | **Country** | **Study**  **type** | **Double-blind** | **Indication** | **Target INR** | **Analyzed**  **(I vs C)** | **Ethnicity**  **(Entire cohort or I vs C)** | **Male %**  **(I vs C)** | **Age, yrs (mean ± SD or median, IQR)**  **(I vs C)** | **Initial dosing (I vs C)** | **Subsequent dosing**  **(I vs C)** | **Follow-up time (days)** | **Main results** |
| --- | --- | --- | --- | --- | --- | --- | --- | --- | --- | --- | --- | --- | --- | --- | --- | --- |
|  |  |  |  |  |  |  |  |  |  |  |  |  |  |  |  |  |
| 1 | Gage 2004^26^ | Ini-pgx | Lenzini 2008^208^ | Included in initial dosing intervention arm – see Gage 2008^48^ Ini-pgx validation | | | | | | | | | | | | |
| 2 | Hillman 2004^30^ | Ini-pgx | Hillman 2005^232^ | USA | RCT | No | AF, DVT, PE, HV, other | 1.9–3.0 | 18 vs 20 | All White | 44 vs 45 | 69 ± 11 vs 71 ± 13 | Hillman equation vs 5 mg/d | Usual clinical practice (INR-based) | 28 | The mean PTTR was similar in both arms (42 ± 25 vs 41.5 ± 24.9). |
| 3 | Sconce 2005^33^ | Ini-pgx | McMillin 2010^71^ | USA | PC | No | Elective total hip or knee arthroplasty | 1.8–2.9 | 115 vs 114 | 90% White | 37 vs 40 | 58 ± 12 vs 59 ± 13 | First 2 days (Sconce vs 5mg/d) | Usual clinical practice (INR-based) | 90 | Similar average time to attain therapeutic INR (~4 d), similar time within therapeutic range and similar frequency of adverse clinical events. |
| 4 | Carlquist 2006^37^ | Ini-pgx | Anderson 2007^38^ | USA | RCT | No | AF, DVT, PE, orthopedic surgery, other | 2.0–3.0 | 101 vs 99 | 94% White vs 95% White | 50 vs 57 | 63 (range 25–86) vs 59 (range 18–82) | Carlquist equation vs Kovacs 2003 nomogram | Usual clinical practice (INR-based) | 90 | Percent out-of-range INRs (pharmacogenetic = 30.7%, standard = 33.1%) similar. |
| 5 | Tham 2006^41^ | Ini-pgx | Syn 2018^244^ | Singapore and  Malaysia | RCT | No | AF, DVT, PE, stroke, other | 1.9–3.1 | 133 vs 136 | 100% Asian | 63 (of 159) vs 54 (of 163) | 58 ± 14 (of 159) vs 59 ± 15 (of 163) | First 3 days  Tham equation vs 5, 5, 3 mg (if <75 yrs) of 5, 4, 3 mg (if ≥75 yrs) | Usual clinical practice (INR-based) | 90 | The PTTR (MD 2.9%, 95% CI -2.6% to 8.4%, P=0.29), median time (d) to stable INR (36 [IQR 20–74] vs 37 [IQR 22–76]) and safety profiles were similar. Fewer dose titrations in the pgx arm. |
| 6 | Caldwell 2008^46^ | Ini-pgx | Burmester 2011^47^ | USA | RCT | No | AF, DVT, PE, HV | 2.0–3.5 | 113 vs 112 | 100% White including Hispanics | 57 vs 61 (of 115) | 67.4 (range 40–90) vs 69.2 (40–91) (for 115 each) | Clinical model | Usual clinical practice (INR-based) | 60 | PTTR in the first 14 d (29 ± 16 vs 31 ± 18, P=0.564) and number of adverse effects (8 per arm) similar. |
| 7 | Gage 2008^b48^ | Ini-pgx | Lenzini 2008^208^ | USA | PC | No | Orthopedic (total knee or hip replacement) | 1.5–2.7 (for most) | 179 vs 233 | 89% White, 9% Black, 2% Other vs 87% White, 12% Black, 1% Other | 86 vs 109 | 57 ± 12 vs 59 ± 15 | Based on either Gage 2004^26^ or Gage 2008^48^ (first 3 doses) vs Gage clinical | Millican 2007^207^ (day 4), Lenzini 2008^208^ (day 5+) then INR-based vs Lenzini 2007^216^ (day 4), Lenzini 2008^208^ (day 5+) then INR-based | 1 month | The PTTR was 7% higher (95% CI: 2.7 to 11.7) in the pgx arm. The risk of an adverse event was also reduced (hazard ratio 0.54; 95% CI: 0.30-0.97). |
| 8 |  |  | Jonas 2013^269^ | USA | RCT | No | AF, DVT, PE, HV, other | 2.0–3.5 | 53 vs 53 | 80% White, 20% Black vs 65% White, 35% Black | 44 vs 50 | 59 ± 19 vs 55 ± 19 | Gage pharmacogenetic vs Gage clinical | Standard protocols at anti-coagulation clinics | 90 | PTTR lower in the genotype-guided (45 ± 27 vs 49 ± 27) although this was not statistically significant (P=0.59). |
| 9 |  |  | Kimmel 2013^251^ | USA | RCT | Yes | AF, DVT, PE, other | 2.0–3.0 | 484 vs 471 | 73% White (including Hispanics) and 27% Black in both groups | 51 | 59 (48–70) (for 514) vs 57 (IQR 46–68) (for 501) | First 3 days:  Modified Gage pgx equation (all treated as CYP2C9 *1/*1) vs Gage 2008^48^ clinical equation | Lenzini 2010^210^ pgx vs clinical equations (days 4-5) followed by INR-based adjustments | 180 | At 28 d, mean PTTR was similar in both arms (45 ± 27 vs 45 ± 26, P=0.91). Subgroup analysis indicated that genotype-guided dosing was associated with a worse PTTR in Blacks (35 ± 26 vs 44 ± 27, P=0.01). |
| 10 |  |  | Supe 2015^270^ | Croatia | PC | No | Acute ischemic stroke (mainly due to AF) | 2.0–3.0 | 106 vs 104 | All White | 44 vs 40 | 68 ± 14 vs 69 ± 12 | Warfarindosing.org vs 6 mg/d for days 2 to 5 | Usual clinical practice (INR-based) | 21 | The Pgx arm achieved target INR values earlier (4.2 [95% CI 4.1 to 4.7] vs 5.2 d [95% CI 4.7 to 6.4, 95% CI], p = 0.0009) and had a higher PTTR (76% [95% CI 75 to 79] vs. 67% [95% CI 65 to 70]). |
| 11 |  |  | Jin 2017^271^ | China | RCT | No | PE | 2.0–3.0 | 115 vs 123 | 100% Asian | 50 vs 51 (or 50 vs 49) | 69 ± 12 vs 68 ± 12 | First 3 days  WarfarinDosing.org vs 3 mg/d | Usual clinical practice (INR-based) | 84 | Shorter time (d) to reach target INR target range (10.1 ± 2.9 vs 12.6 ± 3.1, P<0.001) and achieve stable dose (20.9 ± 4.5 vs 23.9 ± 6.0, P<0.001) in the Pgx arm |
| 12 |  |  | Gage 2017^272^ | USA | RCT | No | DVT prevention in elective hip or knee arthroplasty | 1.5–3.0 | 808 vs 789 | 91% White 6% Black, 2% Asian, <1% other vs 91% White 6% Black, 2% Asian, <1% other | 36 vs 37 | 72 ± 5 vs 72 ± 6 | Days 1-11: Pgx-guided vs clinically guided strategies using warfarindosing.org | Usual clinical practice (INR-based) | 90 | Genotyping improved the PTTR over 28 days (MD 3.4, 95% CI 1.1 to 5.8, p=0.004), although not for Blacks (MD 0.2, 95% CI -8.9 to 9.4, P= 0.96). It also reduced the combined risk of major bleeding/INR≥4/VTE/death. |

**Table S8. Continued.**

| **#** | **Algorithm** | **Sub-type** | **Clinical utility study** | **Country** | **Study**  **type** | **Double-blind** | **Indication** | **Target INR** | **Analyzed**  **(I vs C)** | **Ethnicity**  **(Entire cohort or I vs C)** | **Male %**  **(I vs C)** | **Age, yrs (mean ± SD or median, IQR)**  **(I vs C)** | **Initial dosing (I vs C)** | **Subsequent dosing**  **(I vs C)** | **Follow-up time (days)** | **Main results** |
| --- | --- | --- | --- | --- | --- | --- | --- | --- | --- | --- | --- | --- | --- | --- | --- | --- |
|  |  |  |  |  |  |  |  |  |  |  |  |  |  |  |  |  |
| 13 | Gage 2008^b48^ | Ini-pgx | Makar-Ausperger 2018^273^ | Croatia | RCT | No | AF, DVT, PE | 2.0–3.0 | 106 vs 99 | Not stated (from majority White country) | 52 vs 43 | 70 (53–77) vs 73 (59–81) | Days 2-5: WarfarinDosing.org-based pgx strategy vs 6 mg/d | Standard clinical practice (INR-based) | 28 | No difference in PTTR (MD -2, 95% CI -7 to 4, P=0.513) and total number of side effects (OR=1.54; 95% CI 0.44 to 5.63; P=0.464) 5 d after admission. Sub-group analysis of 62 AF patients (30 vs 32) revealed benefit (PTTR MD =12, 95% CI 0 to 23, P=0.040). |
| 14 |  |  | Panchenko 2020^274^ | Russia | RCT | No | AF, VTE, HV, other | 2.0–3.0 | 127 vs 136 | All White | 47 vs 57 | 61 (53–70) vs 64 (57–71) | Gage equation (days 1-4) vs 5 mg/d (days 1-2) | INR-based adjustment | 180 | Pgx dosing decreased major bleedings (0 vs. 6, p = 0.031), time to target INR (11 [9 to 14] vs 17 [15 to 24] d, p = 0.046), and frequency of INRs ≥4.0 (11% vs. 30.9%, p = 0.002). |
| 15 | Wen 2008^56^ | Ini-pgx | Wen 2017^259^ | Taiwan  (China) | RCT | No | AF, DVT, PE, stroke, other | 2.0–3.0 | 107 (Wen 2008^56^) vs 107 (IWPC 2009^3^) vs 104 | All Asian (Han Chinese) | 55 vs 54 vs 61 | 67 (range 30–92) vs 67 (range 26–88) vs 66 (range 33–89) | Days 1-3  Wen equation vs IWPC 2009^3^ Pgx equation vs 5 mg/d | INR-based adjustment | 90 | PTTR was not different between the 3 arms (respectively 50 ± 35 vs 52 ± 34 vs 52 ± 32, P=0.84). There were also no differences between INRs ≥4.0 (19% vs 17% vs 17%) and other adverse events. |
| 16 | Huang 2009^58^ | Ini-pgx | Huang 2009^58^ | China | RCT | No | HV | 1.8–3.0 | 61 vs 60 | All Asian (Han Chinese) | 33 vs 30 | 42 ± 10 vs 43 ±11 | Huang equation (days 1-3) vs 2.5 mg/d | Usual clinical practice (INR-based) | 50 | The time in therapeutic INR range (d) was higher with genotyping (28 ± 10 vs 22 ± 10, P=0.001). The median time (d) to achieving stable dose (24 vs 35, P=0.001) also reduced. |
| 17 |  |  | Wang 2012^260^ | China | RCT | NI | RHD after HV | 1.8–3.0 | 50 vs 51 | All Asian (Han Chinese) | 30 vs 31 | 42 ± 6 vs 43 ± 9 | Huang equation (3.5 mg/d set as highest) vs 2.5 mg/d for 3 days | INR-based adjustments | 50 | 84% patients in the Pgx arm and 59% patients in the control arm received stable dose with Pgx dosing reducing time to stable dose (28 ± 2 vs 35 ± 2 d, p<0.001) |
| 18 | IWPC 2009^3^ | Ini-pgx | Anderson 2012^99^ | US | PC | No | DVT, PE, AF, other | 1.5–3.5 | 477 vs 1866 | 95% White (for 504) vs 91% White (for 1911) | 47 (for 504) vs 49 (for 1911) | mean 61 (range 18–90) (for 504) vs 57 (18–99) (for 1911) | Pgx1 (IWPC equation modified once) + Pgx2 (IWPC equation modified thrice) vs 5 mg/d | Standard INR-based dose-modification algorithm (after day 7) | 90 | Pgx dosing superior to the parallel controls (PTTR 69 vs 58 at 1 month, 71 vs 59 at 3 months, all P<0.001). Fewer serious adverse events at 3 months (5% vs 9%, P<0.001) with Pgx dosing. |
| 19 |  |  | Pirmohamed 2013^275^ | UK and Sweden | RCT | No | AF, VTE | 2.0–3.0 | 211 vs 216 | 98% White, 1% Black, <1% Asian vs 99% White, <1% Black, <1% Asian | 65 vs 59 | 68 ± 14 vs 67 ± 13 | Modified IWPC equation with loading (Avery 2011^81^, days 1-3) vs day 1 (10mg if ≤75 years and 5mg if >75 years) and days 2-3 (5 mg/d) | Modified Lenzini 2010^210^ equation (days 4-5) vs local practice, followed by local clinical practice for all | 90 | Genotyping improved mean PTTR (67 ± 18 vs 60 ± 22, P<0.001) and reduced the time (d) to therapeutic INR (median 21 [IQR 8 to 39] vs 29 [IQR 14 to 58], P<0.001). |
| 20 |  |  | Tabib 2015^276^ | Iran | PC | No | HV or single ventricular approaches (e.g. Glenn or Fontan operations) | 1.5–3.0 (based on target INRs) | 50 vs 150 | All Asians (Iranians) | 70 vs 61 | 11 ± 3 vs 11 ± 3 | IWPC-based table vs 0.2 mg/kg | Adjusted according to the patient’s height, weight, and BSA vs INR-based | 90 | A shorter time to Pgx-guided dosing (P <0.001) although no statistically significant difference in time to reach the first therapeutic INR, time with over-anticoagulation and bleeding episodes between the two arms. |
| 21 |  |  | Wen 2017^259^ | Included as second pgx arm – see Wen 2008^56^ Ini-pgx validation | | | | | | | | | | | | |
| 22 |  |  | Hao 2019^277^ | China | RCT | No | HV | 2.0–3.0 | 1126 vs 1119 | All Asian (Chinese) | 46 (of 1134) vs 43 (of 1130) | 53 ±11 (for 1134) vs 53 ± 10 (for 1130) | IWPC equation vs 3 mg/d in the first 3 days | INR-based | 90 | Shorter time (3.8 ± 2.0 vs 4.4 ± 2.0 d, P<0.001) to reach therapeutic INR in Pgx arm. No reduction in major bleeding or thrombotic events. |

**Table S8. Continued.**

| **#** | **Algorithm** | **Sub-type** | **Clinical utility study** | **Country** | **Study**  **type** | **Double-blind** | **Indication** | **Target INR** | **Analyzed**  **(I vs C)** | **Ethnicity**  **(Entire cohort or I vs C)** | **Male %**  **(I vs C)** | **Age, yrs (mean ± SD or median, IQR)**  **(I vs C)** | **Initial dosing (I vs C)** | **Subsequent dosing**  **(I vs C)** | **Follow-up time (days)** | **Main results** |
| --- | --- | --- | --- | --- | --- | --- | --- | --- | --- | --- | --- | --- | --- | --- | --- | --- |
|
| 23 | IWPC 2009^3^ | Ini-pgx | Jorgensen 2019^278^ | UK | Matched cohort | No | AF, DVT, PE | 1.8–3.5 | 119 vs 93 | All White | 53 vs 55 | 72 ± 11 vs 70 ± 14 | Modified IWPC equation (days 1-3) vs C: standard clinical practice. | Modified Lenzini 2010^210^ equation (days 4-5) vs local practice, then local clinical practice for all | 90 | MD in PTTR was 8 (95% CI 2 to 14) higher in Pgx group (p=0.006) (7 [95% CI 3 to 12%] higher [p = 0.0004] when dashboard data (n=640) is added to the control group). |
| 24 | Zhang 2020^279^ | China | RC | No | AF, VTE, HV | 1.7–3.0 | 413 vs 431 | All Asian (Chinese) | 43 vs 41 | 57 ± 11 vs 56 ± 12 | IWPC equation vs 2.5 mg/d | Standard clinical practice | 90 | Shorter time to therapeutic INR (10 ± 5 vs 14 ± 8 d, P < 0.001) and higher PTTR (59 ± 11 vs. 53 ± 14, P =0.007) in the pgx group. |
| 25 | Cen 2010^66^ | Ini-pgx | Xu 2018^67^ | China | RCT | Yes | HV | 1.8–2.5 | 100 vs 101 | All Asian (Han Chinese) | 42 vs 45 | 48 ± 11 vs 48 ± 10 | Cen Pgx vs Cen clinical equations for the first 3 days | Standard clinical practice. | 90 | Shorter time to stable dose in Pgx arm (42 ± 24 vs 34 ± 20 d). Similar PTTR at day 30 (47 ± 20 vs 48 ±19, P=0.941) and safety. |
| 26 | Avery 2011^81^ | Ini-pgx | Pirmohamed 2013^275^ | See IWPC 2009^3^ validation | | | | | | | | | | | | |
| 27 | Zambon 2011^21^ | Ini-pgx | Pengo 2015^266^ | Italy | RCT | No | Non-Valvular AF | 2.0–3.0 | 88 vs 92 | All White | 66 vs 65 | 71 (range 39–84) vs 75 (rage 48–88) | Loading dose (max 10 mg) followed by Zambon equation (days 2-6) vs 5 mg/d (days 1-4) and a pharmacodynamic prediction model based on day 5 INR (days 5-6) | PARMA v5.7 software-based dosing | 30 | At 19 d, mean PTTR similar in both arms (52% [95% CI 48 to 56] vs 53% [95% CI 49 to 57], P=0.71) although the percent time spent at INR>4.0 was significantly lower in the Pgx arm (0.7 [95% CI 0.4 to 1.4] vs 1.8 [95% CI 0.4 to 3.3], P=0.02). |
| 28 | Lee 2012^106^ | Ini-pgx | Lee 2020^280^ | Korea | RCT | No | HV | 2.0–3.0 | 42 vs 49 | All Korean | 69 vs 63 | 55 ± 15 vs 57 ± 17 | Lee 2012 vs 5 mg/d (says 2.5 mg on trial registry) | INR-based | 90 | After 7 days, Pgx dosing did not offer a significant clinical advantage (PTTR = 56 vs 47, p = 0.059), but a possible benefit in patients with aortic valve replacement (PTTR = 64 vs. 45, p=0.012) was suggested. |
[truncated: 113,590 more chars]
